# Supplementary material for: BRD4 isoforms have distinct roles in tumour progression and metastasis in rhabdomyosarcoma
Source: EMBO Rep. 2024 Jan 8;25(2):832–52. doi: 10.1038/s44319-023-00033-1 (PMC10897194; doi:10.1038/s44319-023-00033-1)
Supplement: Supplementary file 1 — Appendix [file 44319_2023_33_MOESM1_ESM.pdf]

Appendix Figures and Tables

BRD4 isoforms have distinct roles in tumor progression and metastasis in embryonal rhabdomyosarcoma

Das et al

CONTENT

1. Appendix Figure S1……………………………………………………………..2
2. Appendix Figure S2 ……………………………………………………………3
3. Appendix Figure S3 …………………………………………………………4-5
4. Appendix Figure S4……………………………………………………………6
5. Appendix Figure S5……………………………………………………………7
6. Appendix Table S1…………………………………………………………...8
7. Appendix Table S2…………………………………………………………...9

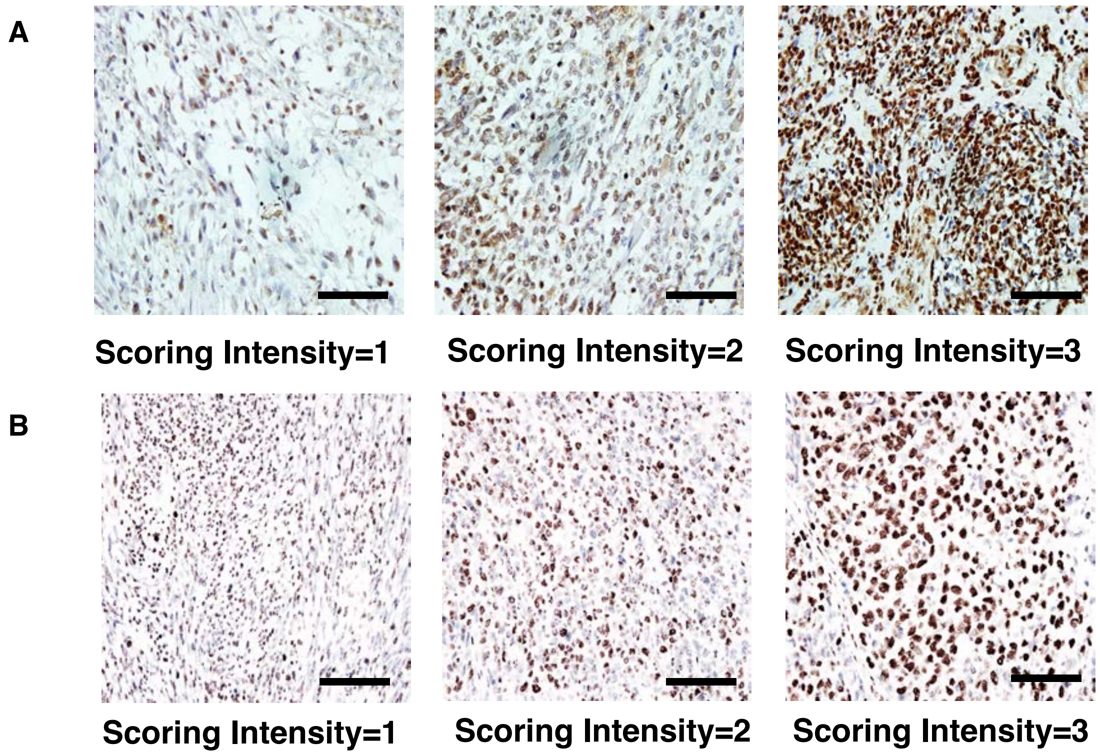

Appendix Figure S1: BRD4 scoring intensity
Representative images showing BRD4 staining intensities in
A TMA sections for ERMS
B ERMS patient tumors
Weak staining intensity was given a score of 1, and moderate and strong intensity staining was given a score of 2 and 3 respectively. Scale bar: 100µm.

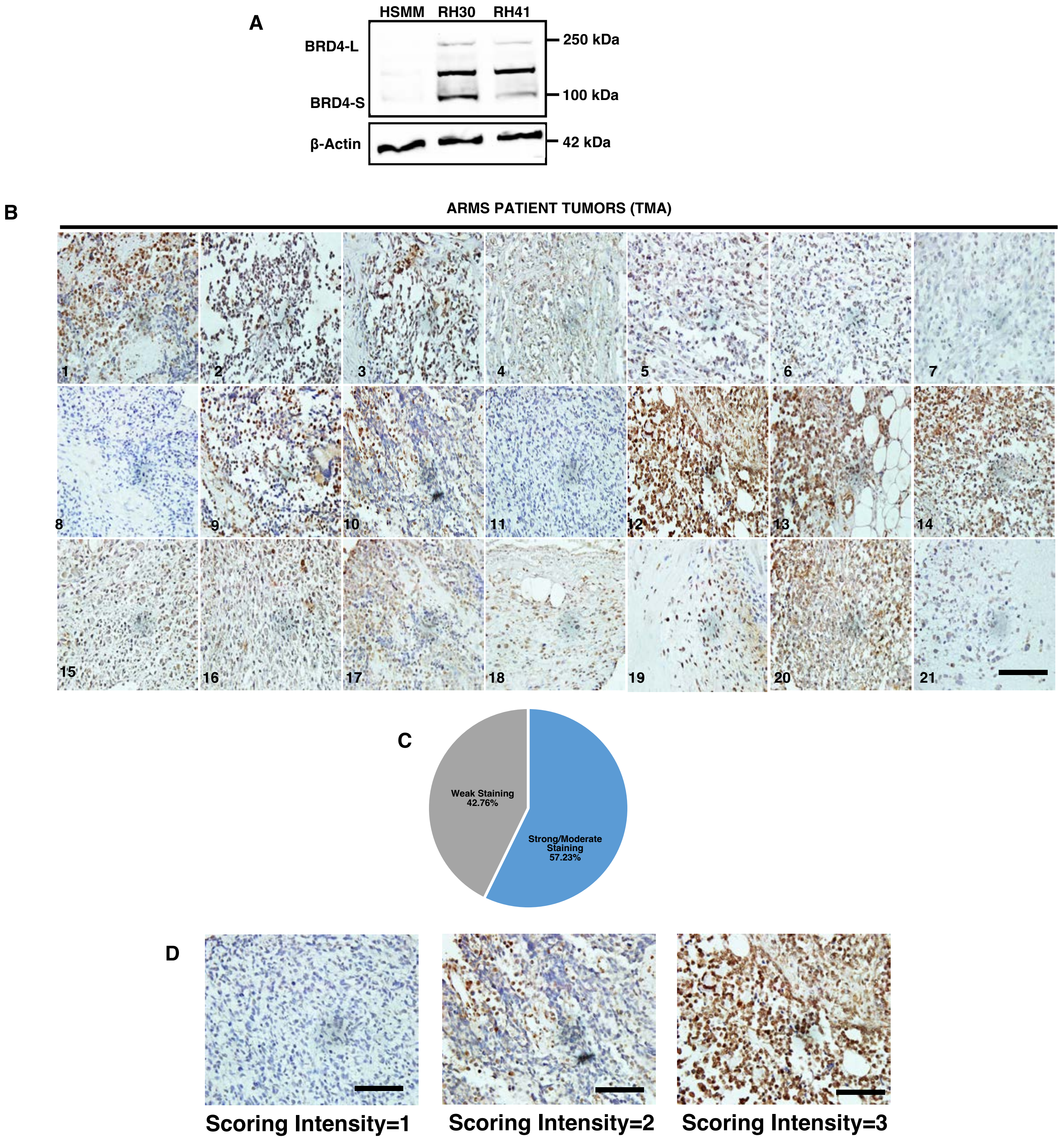

Appendix Figure S2: BRD4 is overexpressed in ARMS

A Western blot analysis showing expression of BRD4-L and BRD4-S isoforms in HSMM, RH30 and RH41 cell lines. β-Actin was used as loading control. Images are representative of at least three biological replicates.

B TMA consisting of 21 ARMS patient tumors was analyzed by IHC using anti-BRD4 antibody. Images were taken at 40X magnification. Scale bar: 100µm.

C Pie chart illustrating the distribution of staining intensities in the samples, with weak staining (intensity = 1) and moderate/strong staining (intensity = 2/3).

D TMA sections for ARMS samples with scoring intensity of 1 (weak staining), 2 (medium staining), and 3 (strong staining) respectively. Scale bar: 100µm.

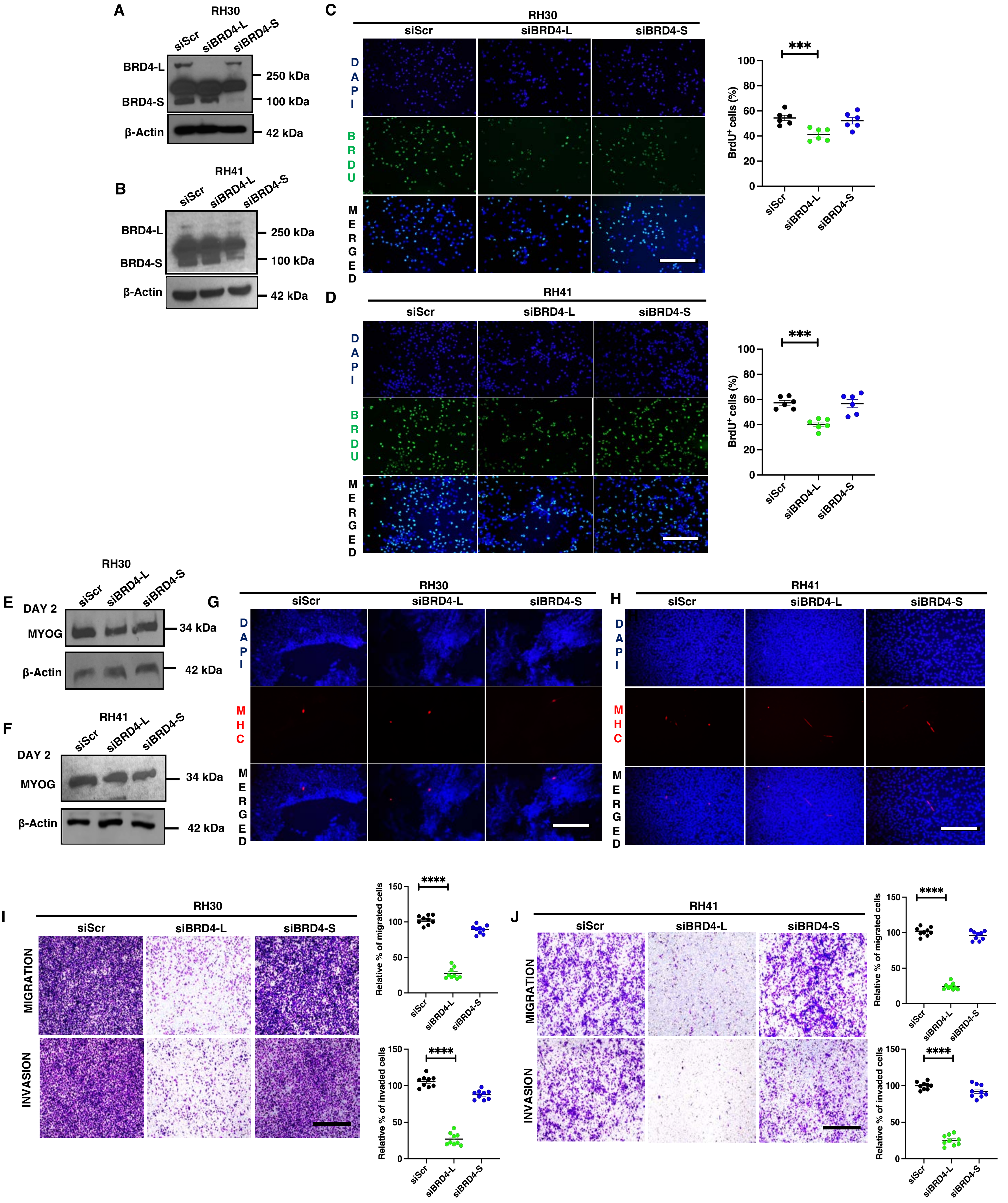
A
RH30
siScr
siBRD4-L
siBRD4-S
BRD4-L
BRD4-S
β-Actin
250 kDa
100 kDa
42 kDa
B
RH41
C
RH30
DAPI
BRDU
MERGED
BrdU⁺ cells (%)
***
D
RH41
E
DAY 2
MYOG
34 kDa
F
G
H
MHC
I
MIGRATION
INVASION
Relative % of migrated cells
Relative % of invaded cells
****
J

Appendix Figure S3: BRD4-L has unrestrained oncogenic role in ARMS

A-B Western blot analysis showing transient BRD4 knockdown efficiency in siBRD4-L and siBRD4-S RH30 and RH41 cells as indicated. β-Actin was used as the loading control. Images are representative of at least three biological replicates.

C-D Proliferation was analysed by BrdU assay in control (siScr), siBRD4-L and siBRD4-S RH30 and RH41 cells as indicated. Images are representative of two biological replicates. Scale bar: 100µm. Scatter plot showing the percentage of BrdU+ cells in siScr, siBRD4-L and siBRD4-S cells. Values correspond to the average ± SEM (n=2 biological replicates with 3 technical replicates shown). Two-tailed non-parametric unpaired t test was performed for statistical analysis. ***$p \leq 0.001$.

E-F Western blot analysis of MYOG at Day 2 in siBRD4-L and siBRD4-S compared to siScr after culturing RH30 and RH41 cells in differentiation media.

G-H Differentiation was analysed by immunofluorescence in siScr, siBRD4-L and siBRD4-S in RH30 and RH41 cells as indicated. 48 hr post-transfection, cells were cultured in differentiation media for 3 days and MHC+ cells were analysed using anti-MHC antibody. Images are representative of at least three biological replicates. Nuclei were stained with DAPI (blue). Scale bar: 100µm.

I-J Migration and invasion was analysed by transwell assays in siScr, siBRD4-L and siBRD4-S RH30 and RH41 cells as indicated. The inserts were stained with crystal violet. Images are representative of at least three biological replicates. Scale bar: 100µm.Scatter plots indicating the percentage of migration and invasion is shown. Values correspond to the average ± SEM (n=3 biological replicates with 3 technical replicates shown). Two-tailed non-parametric unpaired t test was performed for statistical analysis. ****$p \leq 0.0001$.

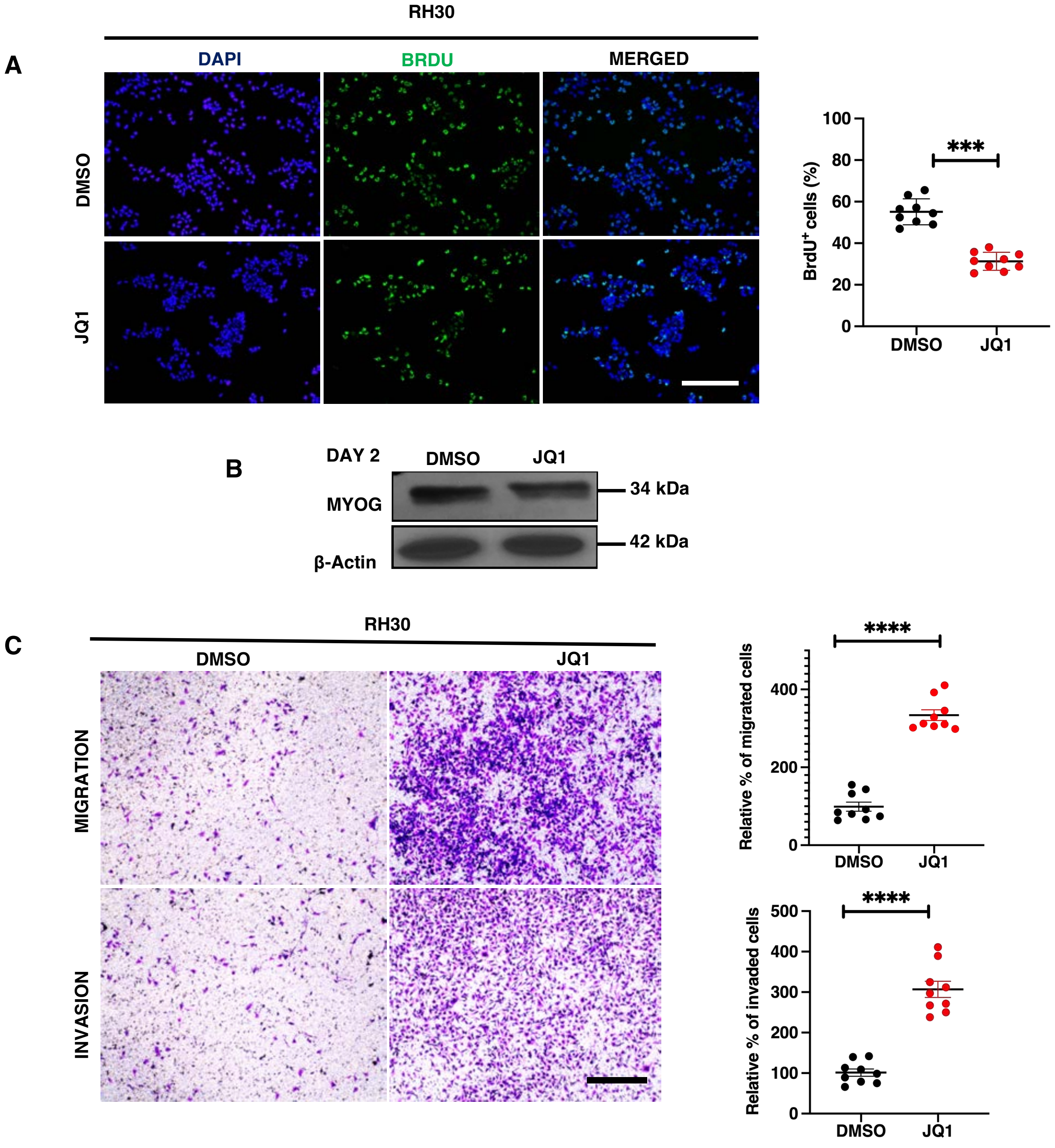

Appendix Figure S4: Effect of JQ1 on proliferation, differentiation and cell motility in RH30 cells

A RH30 cells were treated with DMSO (vehicle) or 50 nM of JQ1 for 48 hr and proliferation was assessed using BrdU assay by immunofluorescence using anti-BrdU antibody. Nuclei were stained with DAPI (blue). Images are representative of at least three biological replicates. Scale bar: 100µm. Scatter plot representing the percentage of BrdU+ cells in RH30 cells treated with DMSO or JQ1. Values correspond to the average ± SEM (n=3 biological replicates with 3 technical replicates shown). Two-tailed non-parametric unpaired t test was performed for statistical analysis. ***$p \leq 0.001$.

B Western blot analysis using anti-MYOG antibody in RH30 cells treated with DMSO or JQ1 in differentiation media for 2 days. β-Actin was used as loading control.

C Migratory and invasive capacity of RH30 cells treated with 50 nM of JQ1 for 48 hr was assessed using transwell assays followed by crystal violet staining of the inserts. Images are representative of at least three biological replicates. Scale bar: 100 µm. Scatter plot representing the percentage of migration and invasion of RH30 cells treated with DMSO or JQ1. The values correspond to average ± SEM (n=3 biological replicates with 3 technical replicates shown). Two-tailed non-parametric unpaired t test was performed for statistical analysis. ****$p \leq 0.0001$.

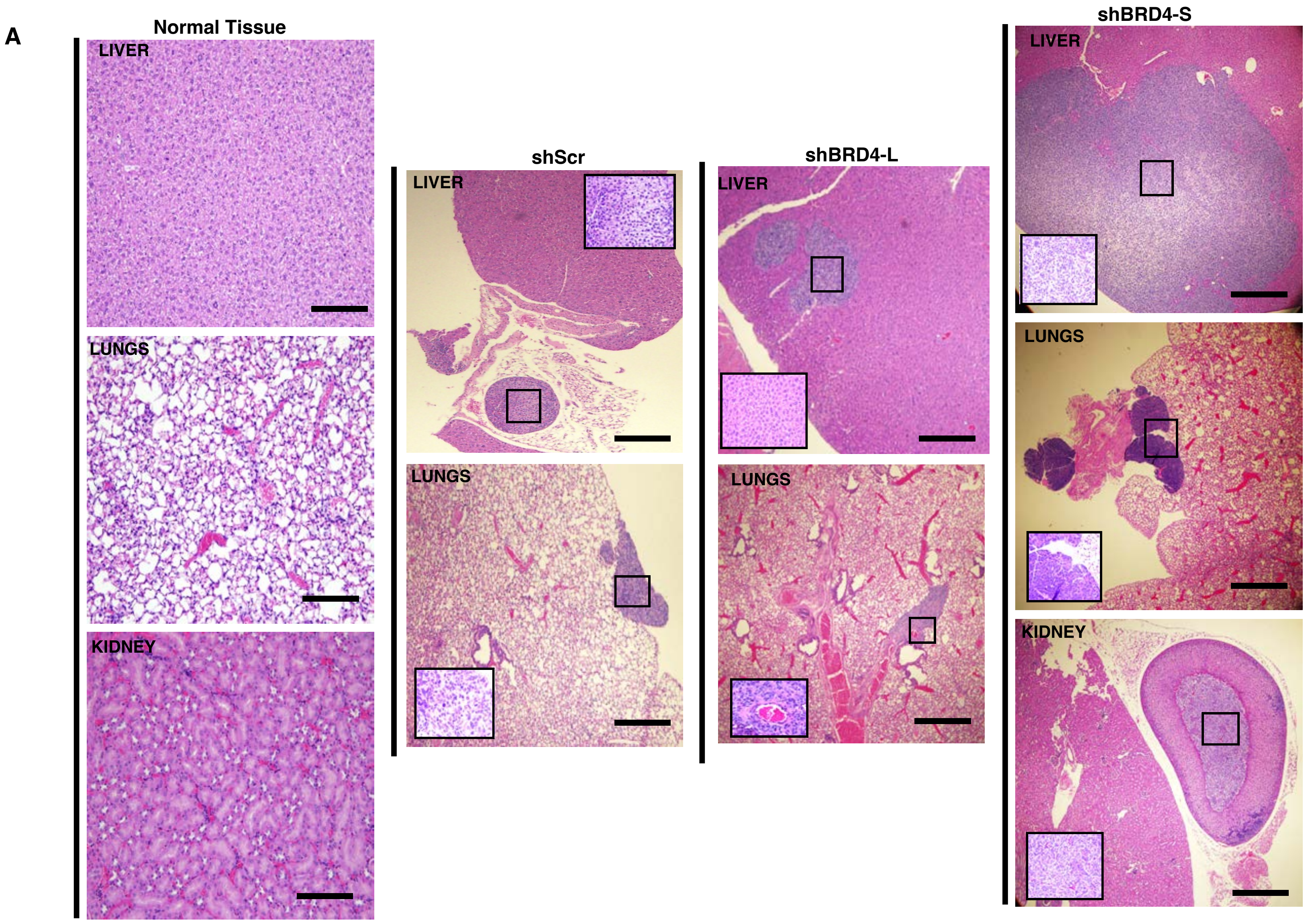

Appendix Figure S5: Histological analysis of metastasis

(**A**) Histology was assessed by H&E staining in the liver and lungs of shScr and shBRD4-L group and in liver, lung and kidney from the shBRD4-S group. Images were taken at 4X magnification to capture the tumor microenvironment. The inset shows 40X magnification of tumors in different organs and healthy tissues as a control (left panel). Scale bar: 100µm.

Gene	Forward Primer	Reverse Primer
BRD4-L	TTCCTTCCCACCTGTGTTTC	CATTGTGTCTGGAGGAGAAGAG
BRD4-S	ACTACTTCCCTCCTGAACCT	CTCTAAAGCTCCCACTCCTTATC
MSTN	CAACGGTGCTAATACGATAGGC	GAGGTGTAGGAAAATGCACCTG
MYL12A	GTTTGACCAGTCGCAGATTCAGG	TAGATACTCATCAGTTGGATTCTTC
MYL5	TCAAGCGTCTGCTGATGTCCCA	TCACGTAGCTGAGCGCCTTGTA
ITGA1	CCGAAGAGGTACTTGTTGCAGC	GGCTTCCGTGAATGCCTCCTTT
ITGA3	GCCTGACAACAAGTGTGAGAGC	GGTGTTCGTCACGTTGATGCTC
ITGA4	GCATACAGGTGTCCAGCAGAGA	AGGACCAAGGTGGTAAGCAGCT
ITGA5	GCCGATTCACATCGCTCTCAAC	GTCTTCTCCACAGTCCAGCAAG

Appendix Table 1 : The primer sequences used for RT-qPCR are shown.

Gene	Forward Primer	Reverse Primer	Position from TSS (+1)
MSTN promoter	GCTCAGTAAGTTGCTCAGTGT	GAGACACTGTGGAGGAACAAC	+1821 to +1971
ITGA1 promoter	GCAATCCGTCTGGGATGTGA	CTCTGGCTGGGCCACTTATC	+219 to +368
ITGA3 promoter	GTTGCCGACAGGTGTTTGG	CTCCTCTTAAAGGGGCAGCAC	-244 to -160
ITGA4 promoter	GGCCCGTACCCGGAGAA	ACACTAAACGGCCACTACCC	-14 to +246
ITGA5 promoter	GCGGGCTCAGAGTTCCAG	CCGCTTCCTAAACCTCCCAG	-107 to +28

Appendix Table 2: Primer sequences used for ChIP -qPCR are shown.
