# Supplementary figures and images for: BRD4 isoforms have distinct roles in tumour progression and metastasis in rhabdomyosarcoma (part 1 of 2)
Source: EMBO Rep. 2024 Jan 8;25(2):832–52. doi: 10.1038/s44319-023-00033-1 (PMC10897194; doi:10.1038/s44319-023-00033-1)

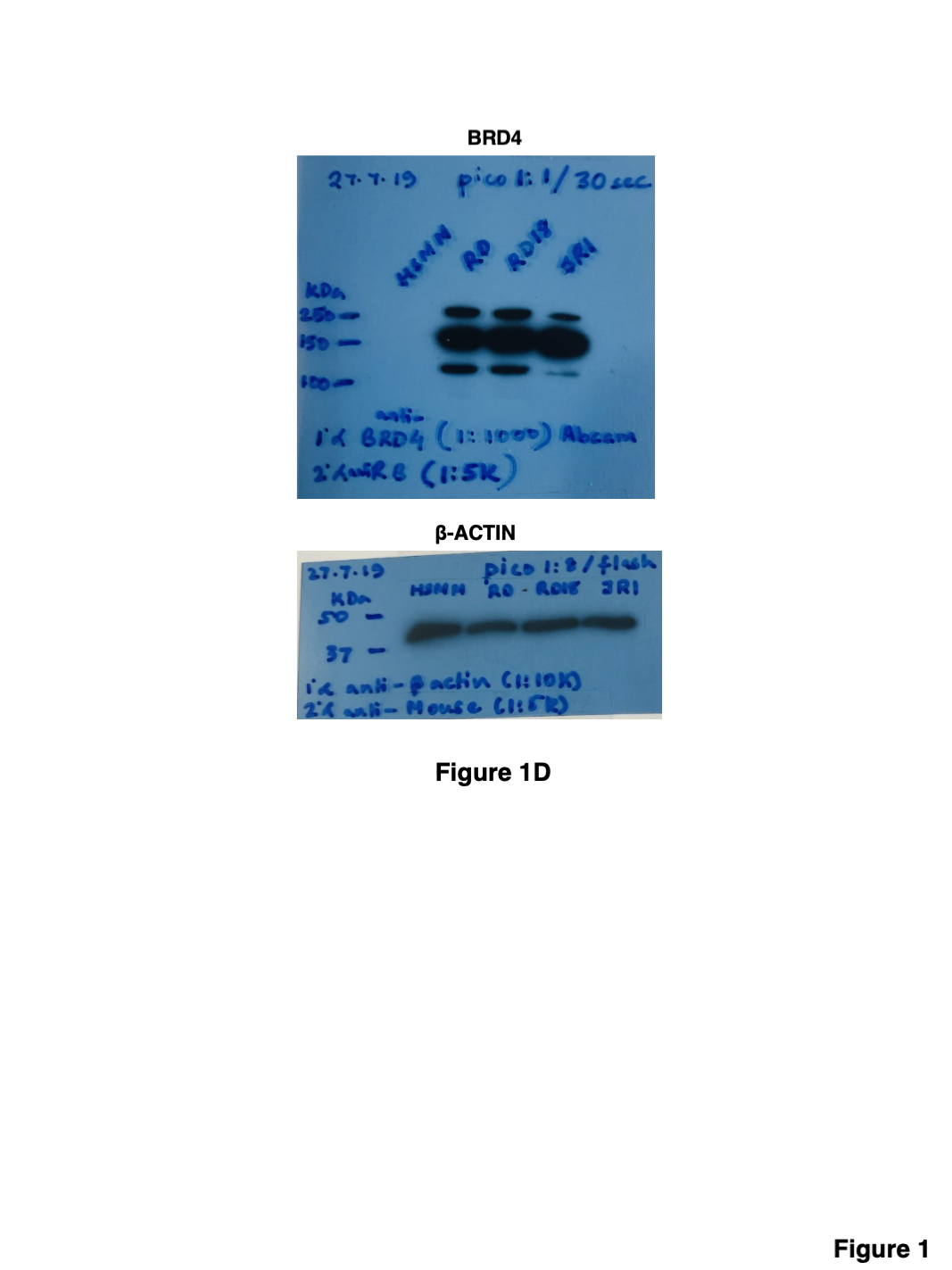

Supplement: Supplementary file 4 — Source Data Fig. 1 [file 44319_2023_33_MOESM4_ESM.zip › Fig.1/Fig. 1D_western.tiff]

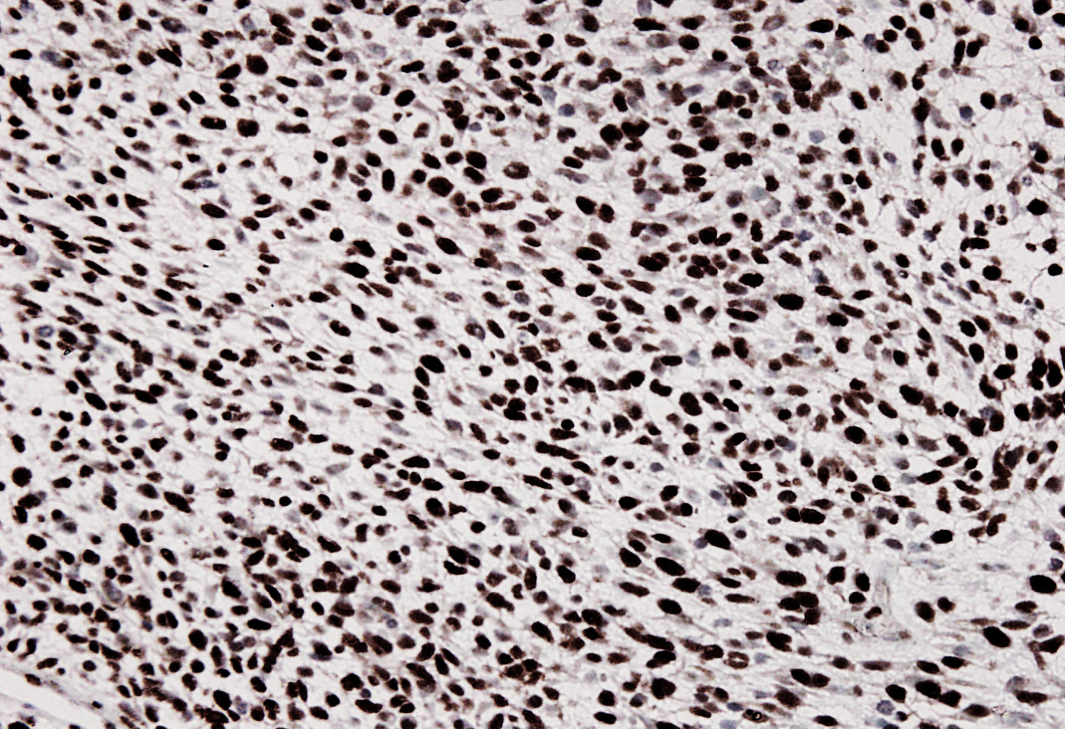

Supplement: Supplementary file 4 — Source Data Fig. 1 [file 44319_2023_33_MOESM4_ESM.zip › Fig.1/Fig. 1G/P6.tiff]

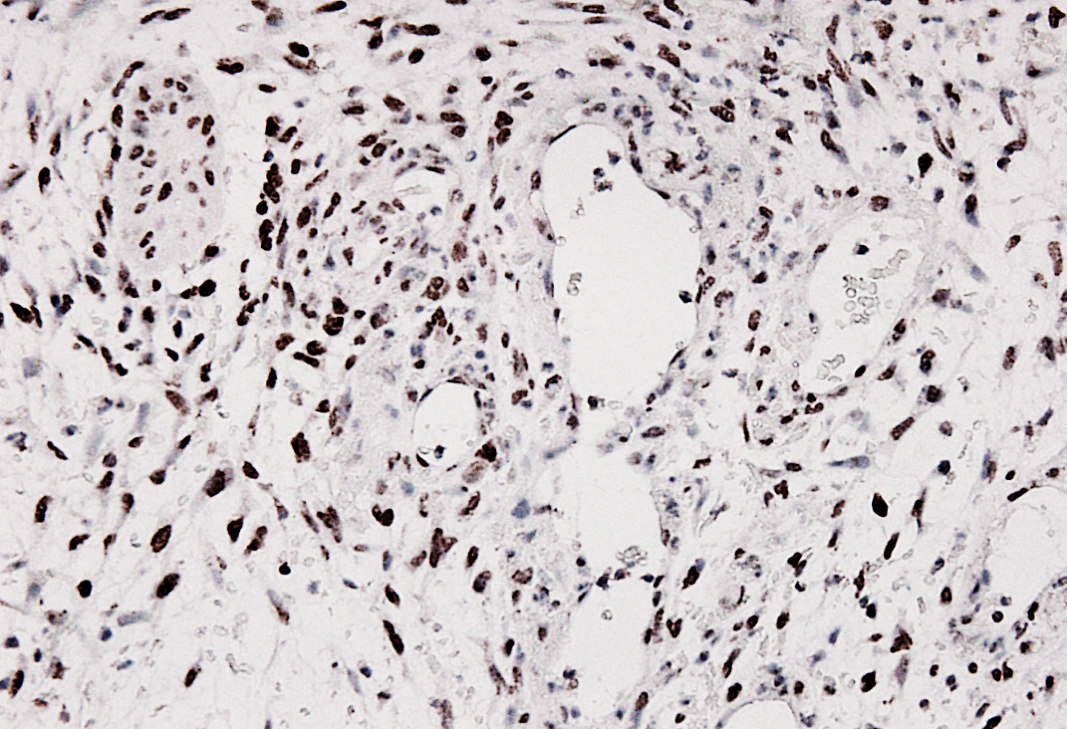

Supplement: Supplementary file 4 — Source Data Fig. 1 [file 44319_2023_33_MOESM4_ESM.zip › Fig.1/Fig. 1G/P1.tiff]

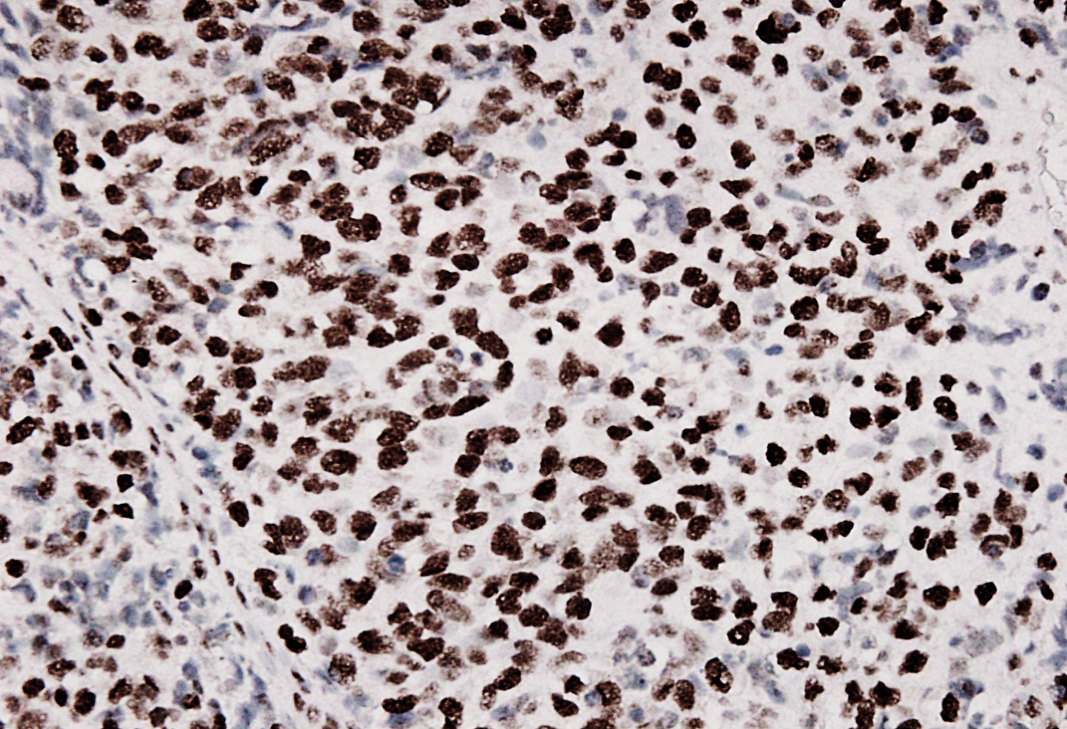

Supplement: Supplementary file 4 — Source Data Fig. 1 [file 44319_2023_33_MOESM4_ESM.zip › Fig.1/Fig. 1G/P2.tiff]

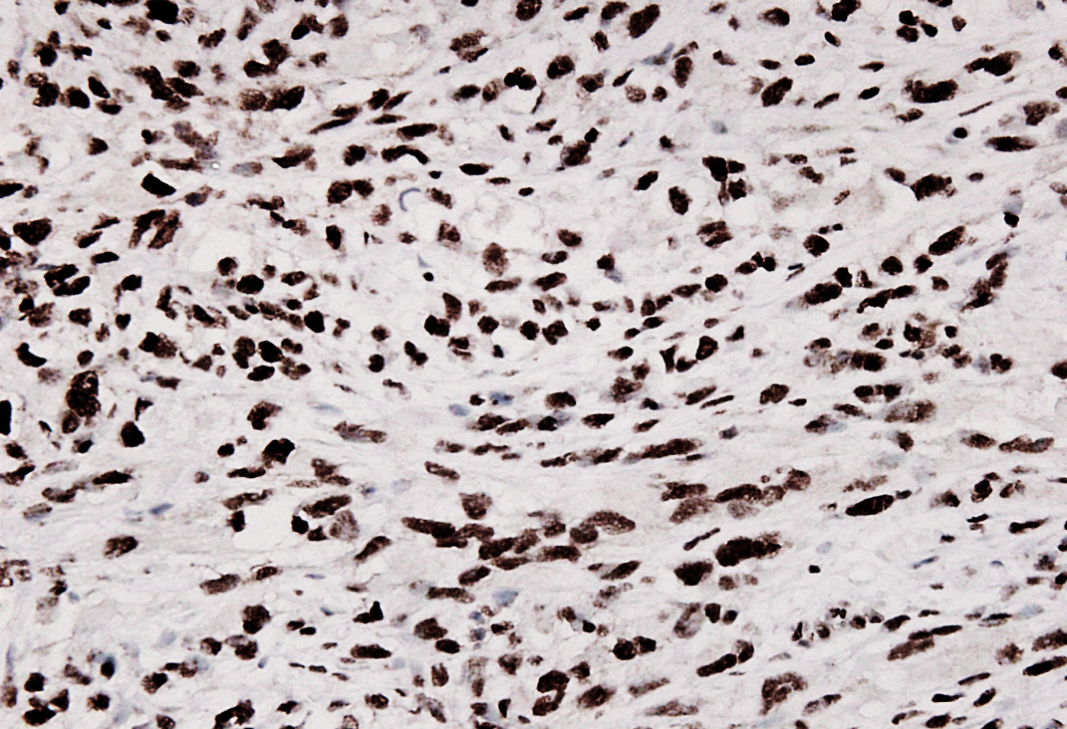

Supplement: Supplementary file 4 — Source Data Fig. 1 [file 44319_2023_33_MOESM4_ESM.zip › Fig.1/Fig. 1G/P3.tiff]

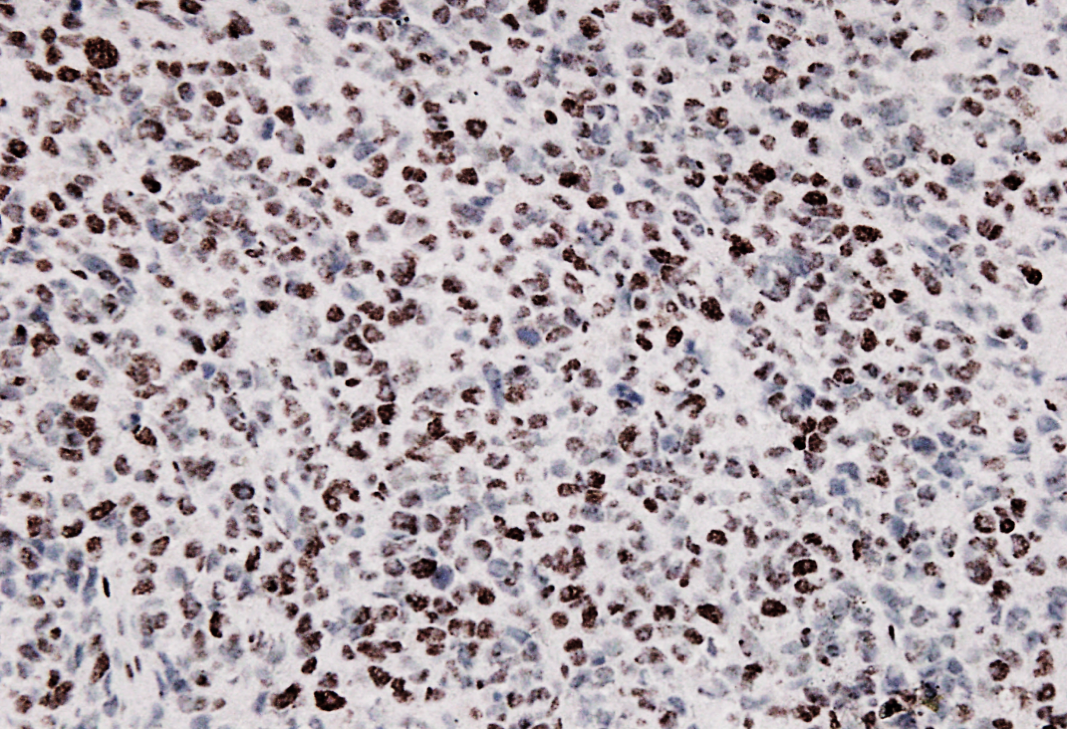

Supplement: Supplementary file 4 — Source Data Fig. 1 [file 44319_2023_33_MOESM4_ESM.zip › Fig.1/Fig. 1G/P4.tiff]

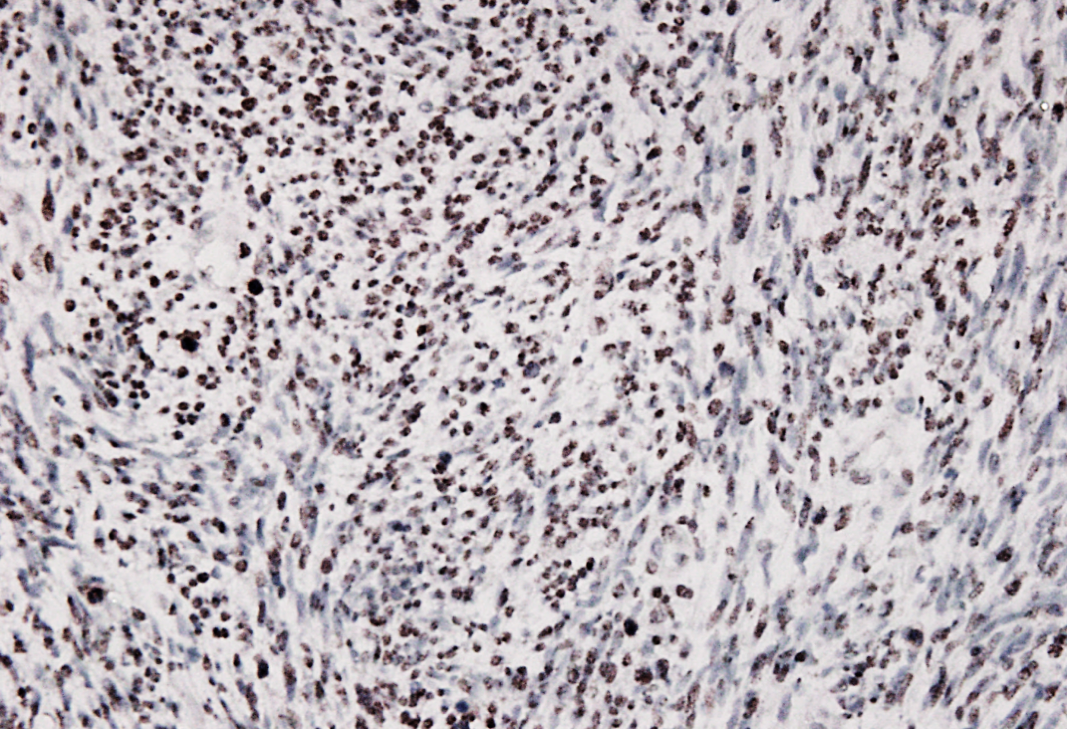

Supplement: Supplementary file 4 — Source Data Fig. 1 [file 44319_2023_33_MOESM4_ESM.zip › Fig.1/Fig. 1G/P5.tiff]

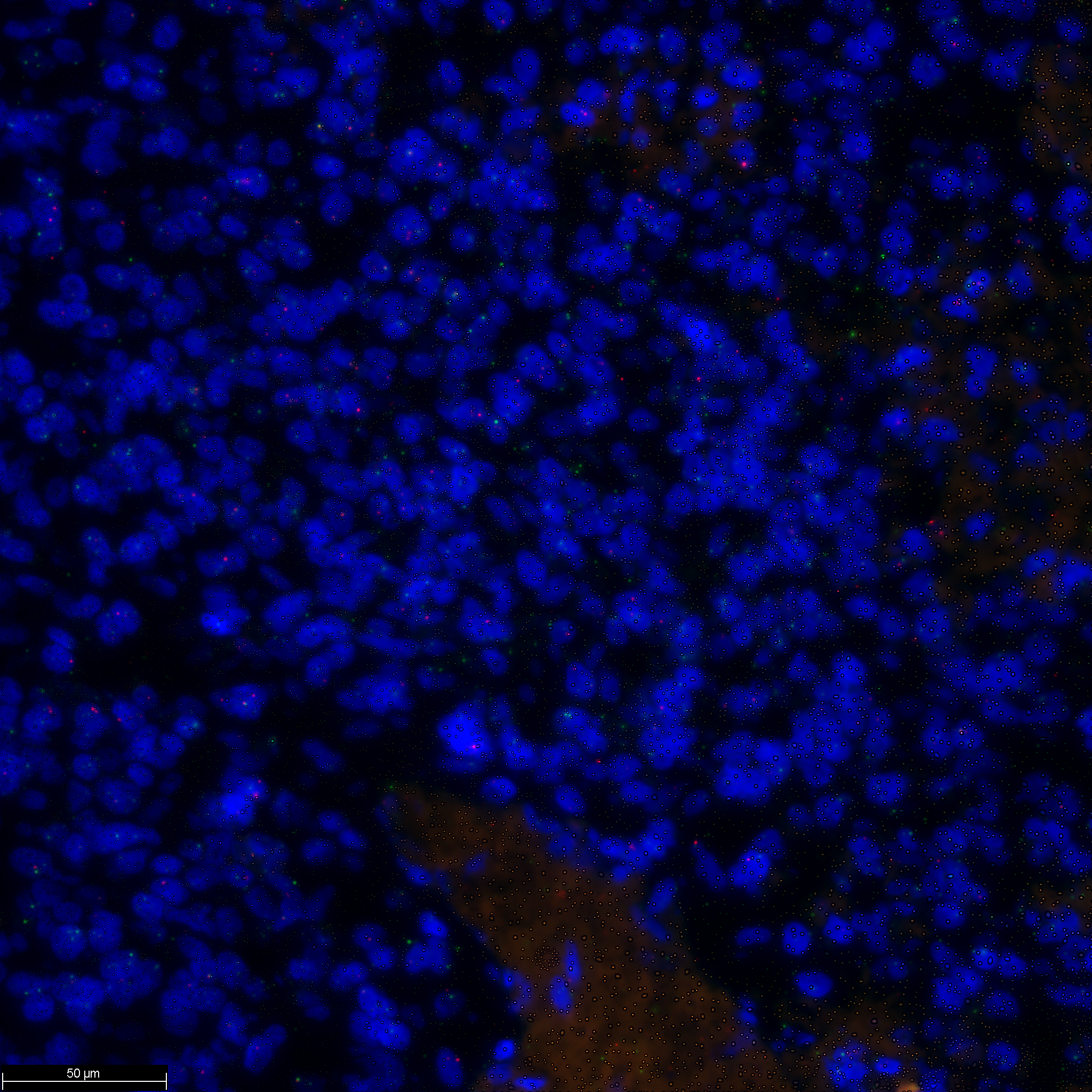

Supplement: Supplementary file 4 — Source Data Fig. 1 [file 44319_2023_33_MOESM4_ESM.zip › Fig.1/Fig. 1H/P4/P4_merge.tif]

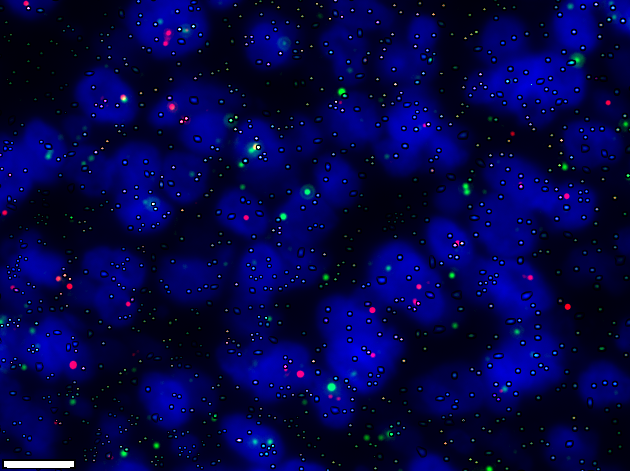

Supplement: Supplementary file 4 — Source Data Fig. 1 [file 44319_2023_33_MOESM4_ESM.zip › Fig.1/Fig. 1H/P4/P4_crop_merge.tif]

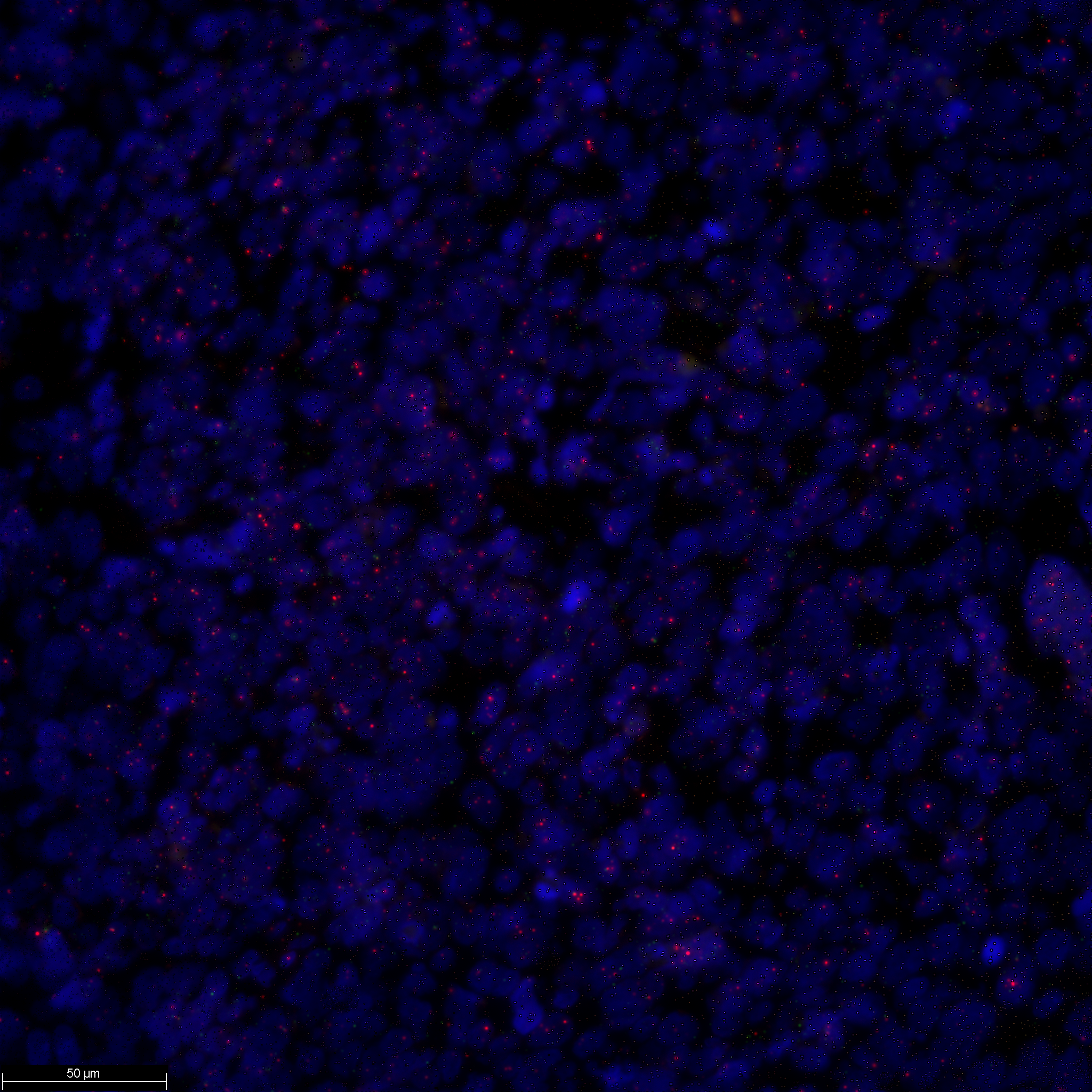

Supplement: Supplementary file 4 — Source Data Fig. 1 [file 44319_2023_33_MOESM4_ESM.zip › Fig.1/Fig. 1H/P3/P3_merge.tif]

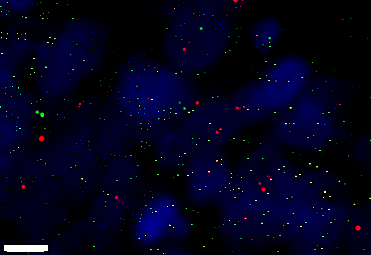

Supplement: Supplementary file 4 — Source Data Fig. 1 [file 44319_2023_33_MOESM4_ESM.zip › Fig.1/Fig. 1H/P3/P3_crop_merge.tif]

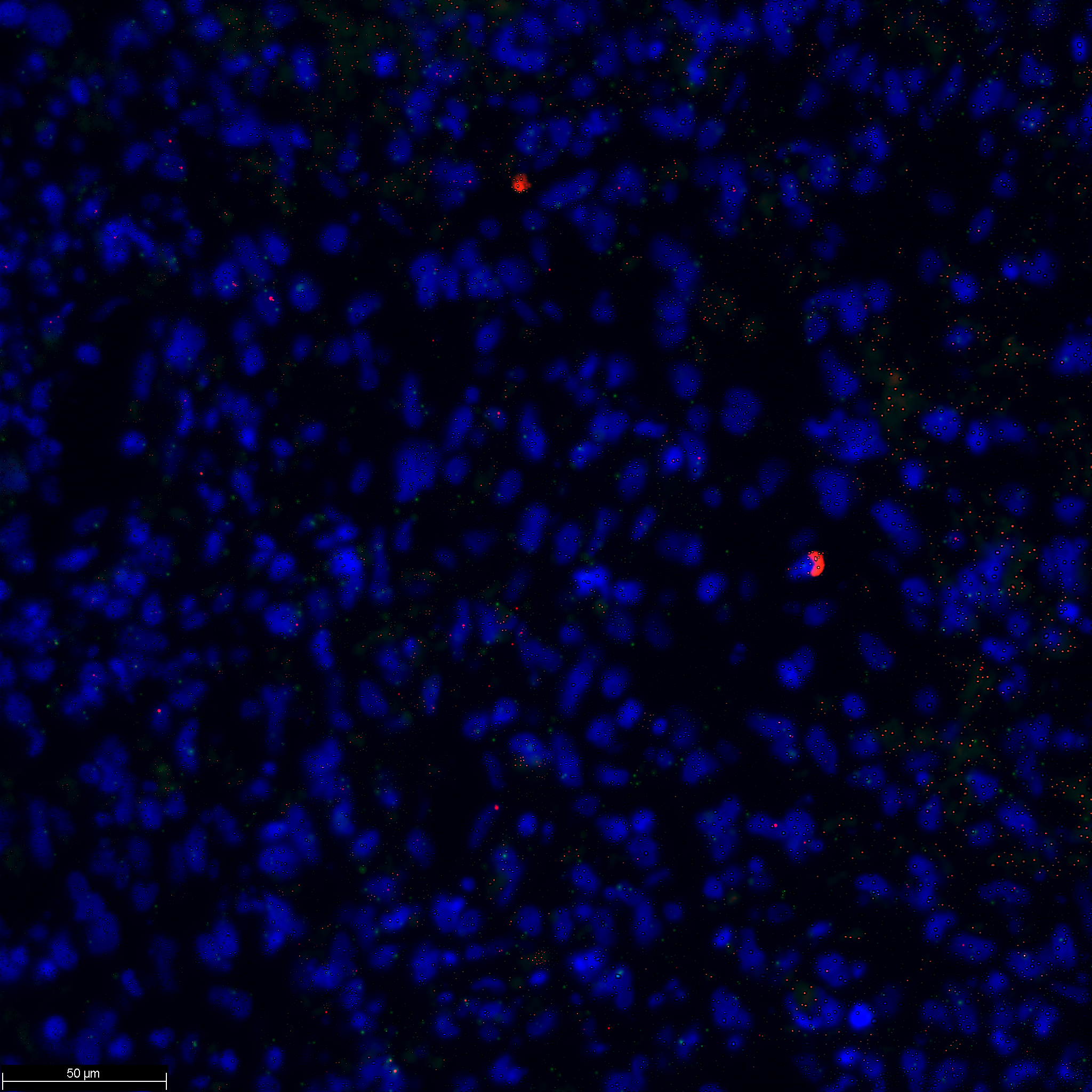

Supplement: Supplementary file 4 — Source Data Fig. 1 [file 44319_2023_33_MOESM4_ESM.zip › Fig.1/Fig. 1H/P2/P2_merge.tif]

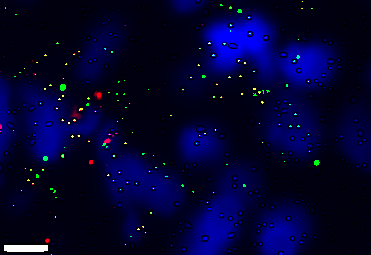

Supplement: Supplementary file 4 — Source Data Fig. 1 [file 44319_2023_33_MOESM4_ESM.zip › Fig.1/Fig. 1H/P2/P2_merge_Crop.tif]

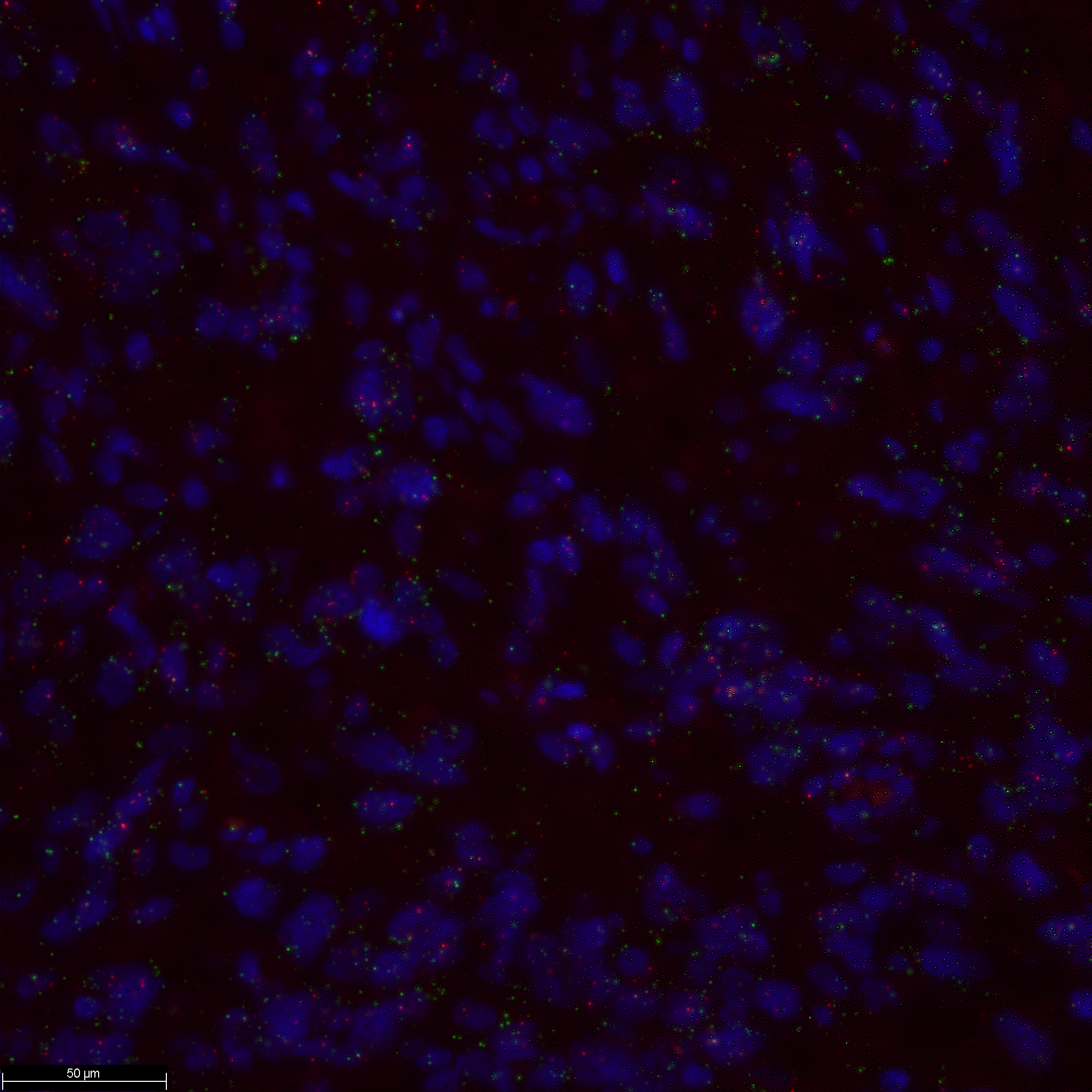

Supplement: Supplementary file 4 — Source Data Fig. 1 [file 44319_2023_33_MOESM4_ESM.zip › Fig.1/Fig. 1H/P5/P5_merge.tif]

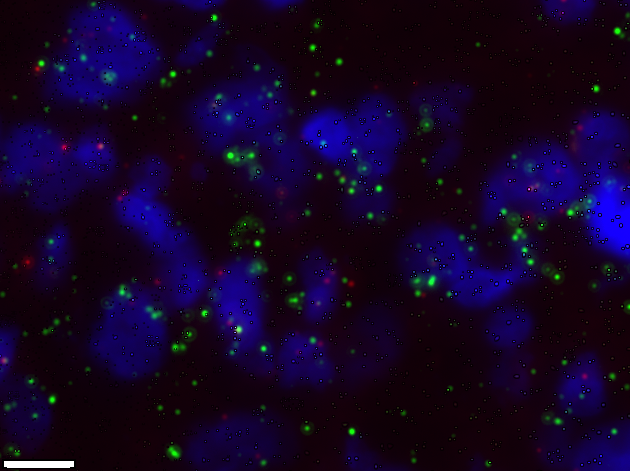

Supplement: Supplementary file 4 — Source Data Fig. 1 [file 44319_2023_33_MOESM4_ESM.zip › Fig.1/Fig. 1H/P5/P5_crop_merge.tif]

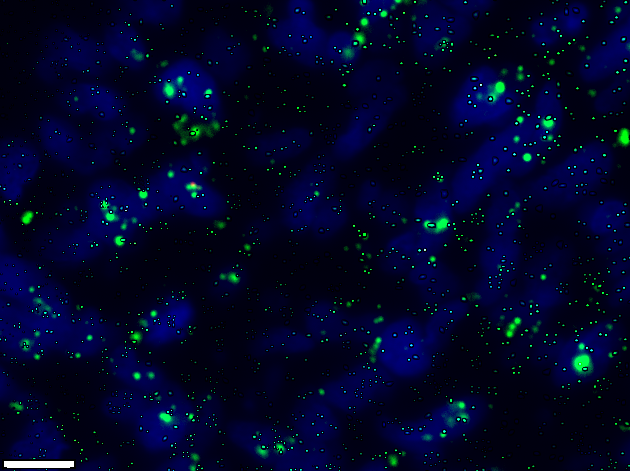

Supplement: Supplementary file 4 — Source Data Fig. 1 [file 44319_2023_33_MOESM4_ESM.zip › Fig.1/Fig. 1H/P1/P1_Merge_Crop.tif]

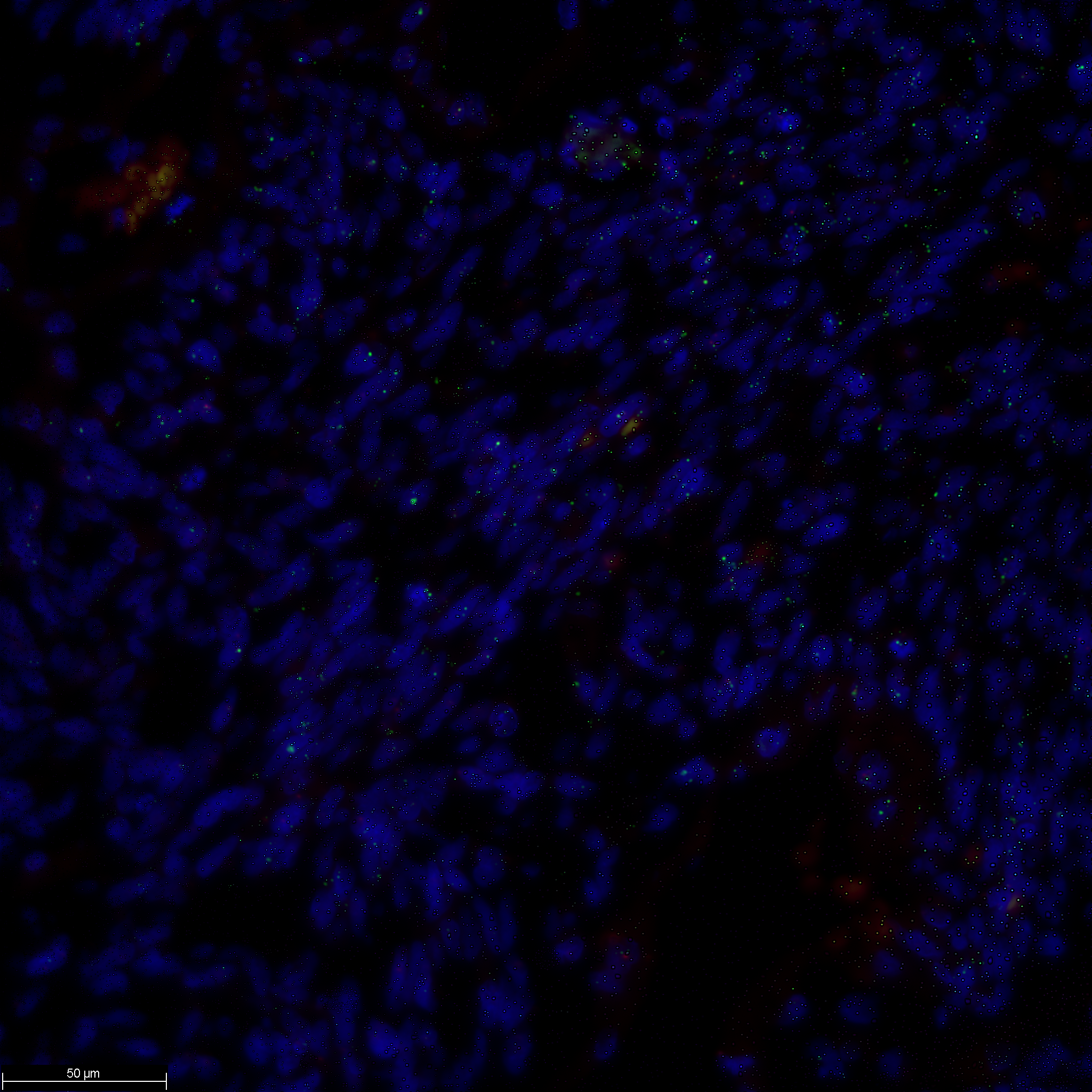

Supplement: Supplementary file 4 — Source Data Fig. 1 [file 44319_2023_33_MOESM4_ESM.zip › Fig.1/Fig. 1H/P1/P1_merge.tif]

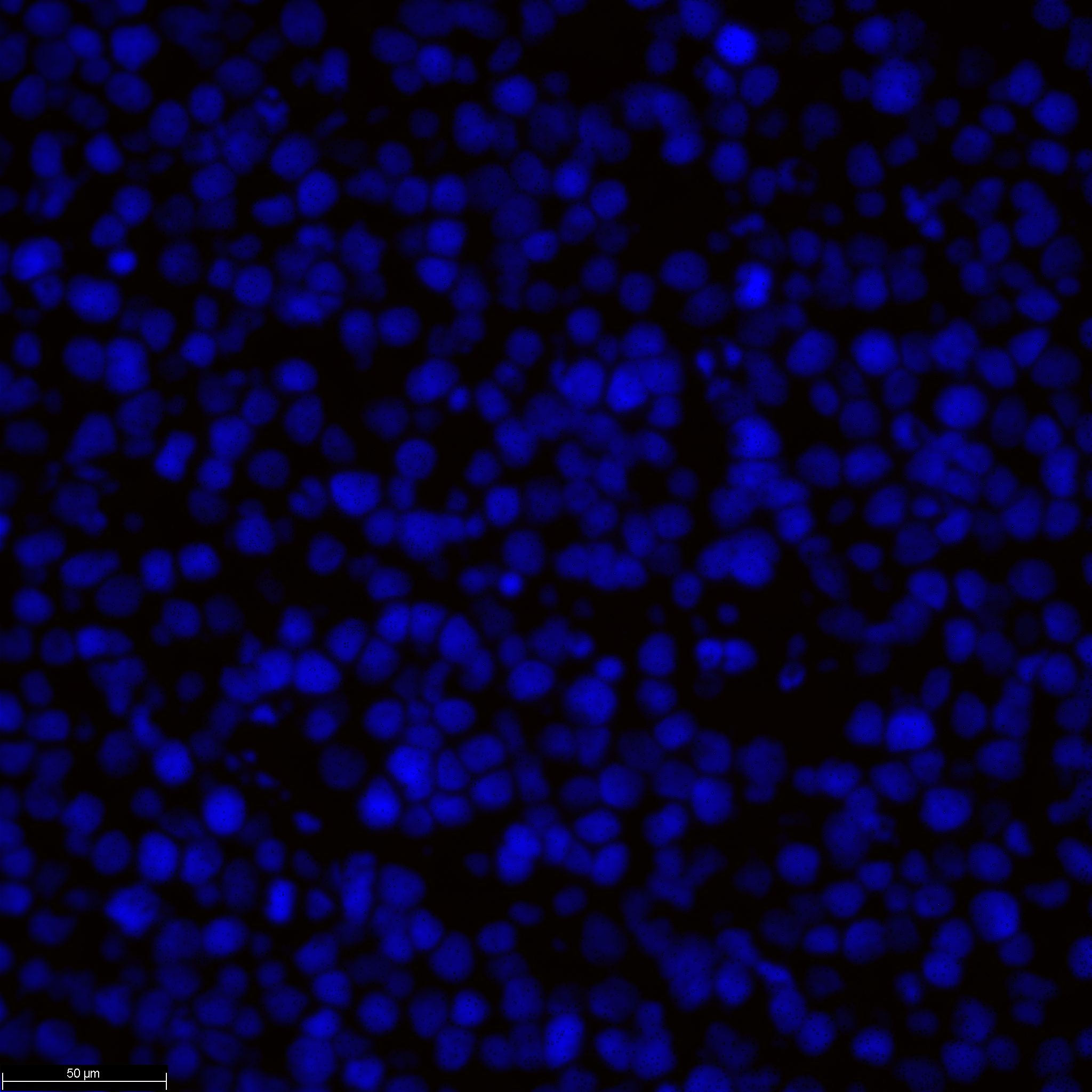

Supplement: Supplementary file 4 — Source Data Fig. 1 [file 44319_2023_33_MOESM4_ESM.zip › Fig.1/Fig. 1H/NC/Neg control_merge.tif]

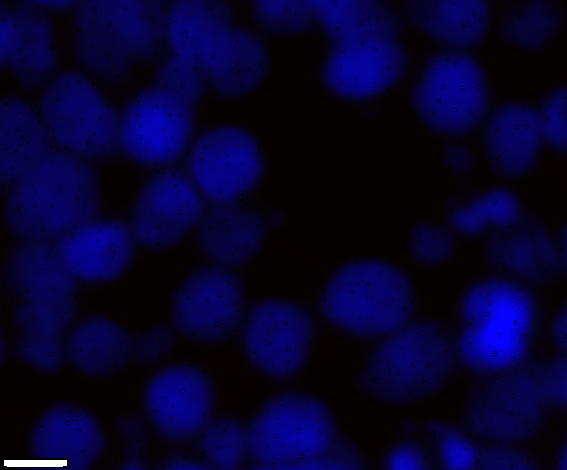

Supplement: Supplementary file 4 — Source Data Fig. 1 [file 44319_2023_33_MOESM4_ESM.zip › Fig.1/Fig. 1H/NC/Neg control_merge_Crop.tif]

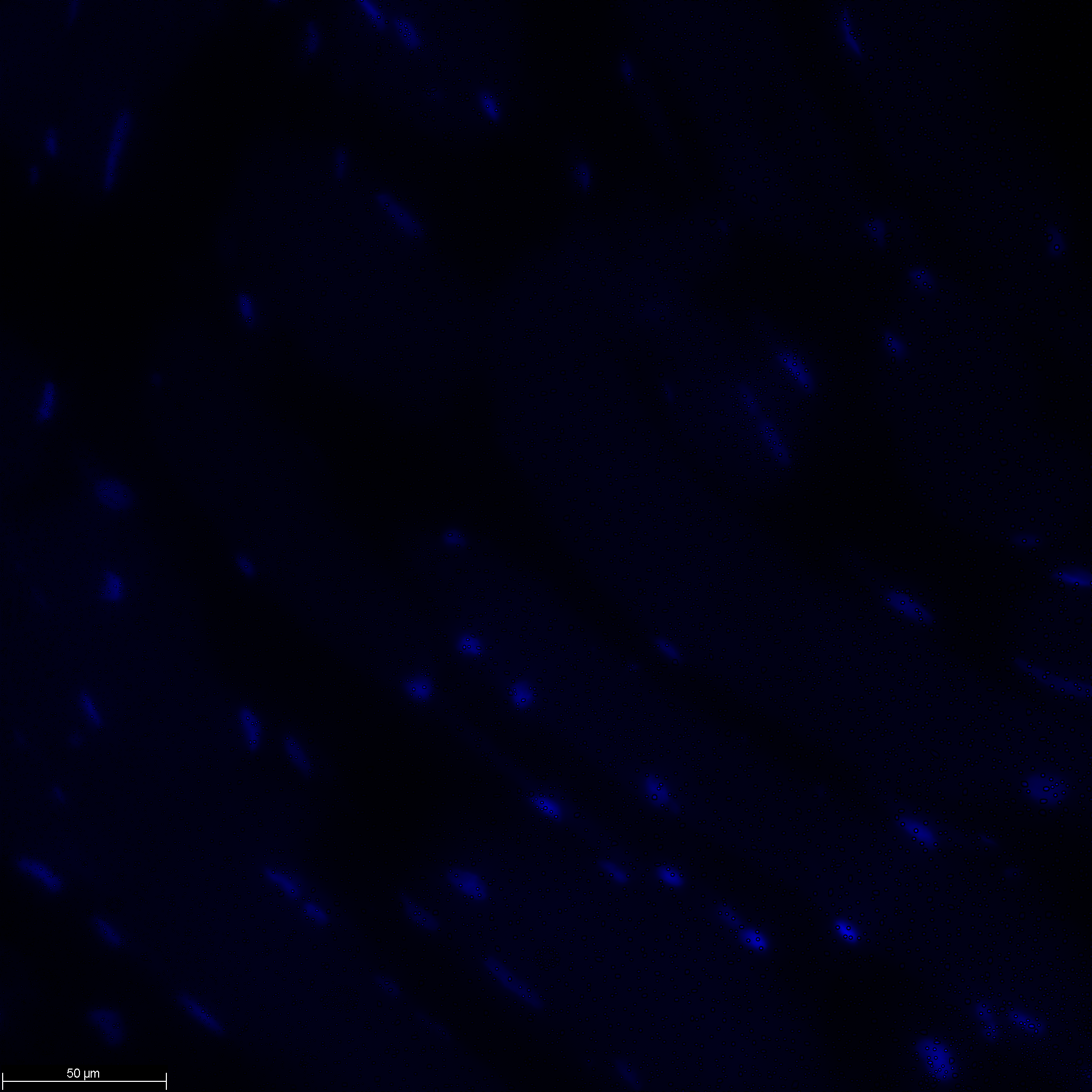

Supplement: Supplementary file 4 — Source Data Fig. 1 [file 44319_2023_33_MOESM4_ESM.zip › Fig.1/Fig. 1H/NM/NM_merge.tif]

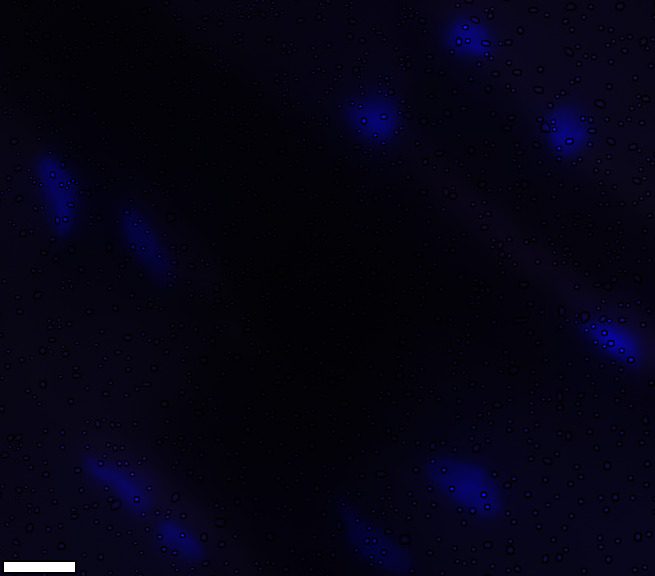

Supplement: Supplementary file 4 — Source Data Fig. 1 [file 44319_2023_33_MOESM4_ESM.zip › Fig.1/Fig. 1H/NM/NM_merge_crop.tif]

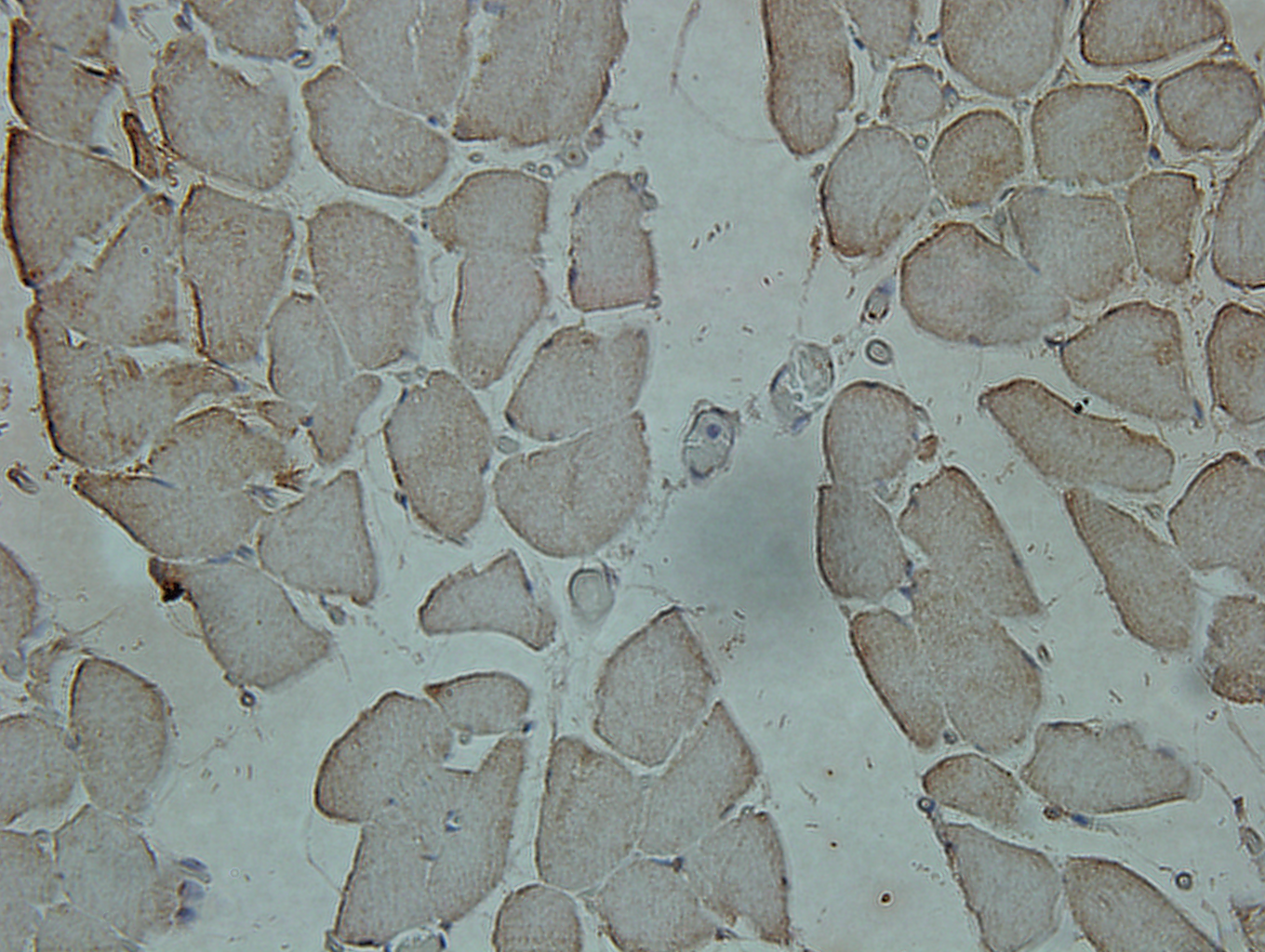

Supplement: Supplementary file 4 — Source Data Fig. 1 [file 44319_2023_33_MOESM4_ESM.zip › Fig.1/Fig. 1E/NORMAL MUSCLE/M5_Fig.tif]

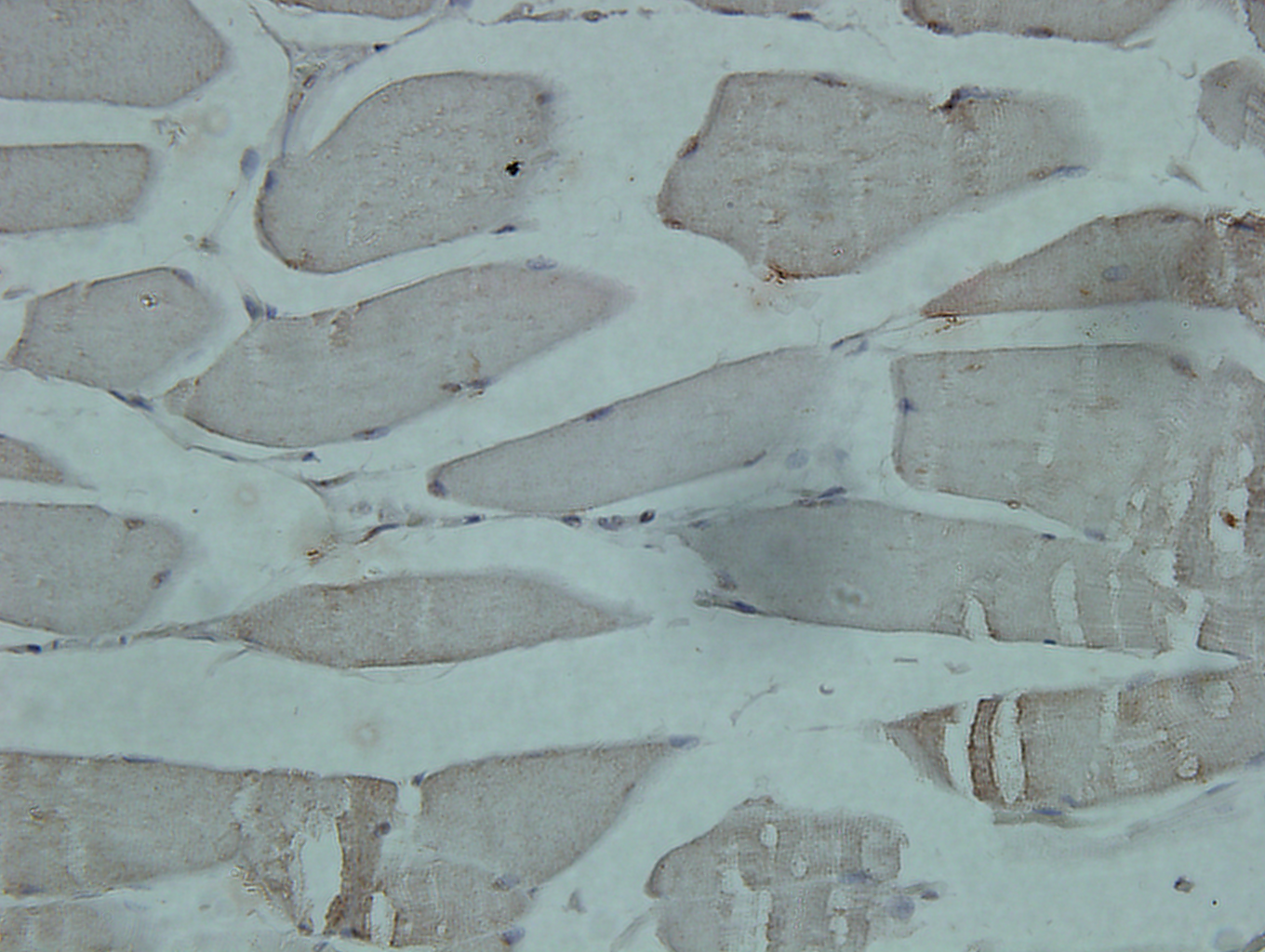

Supplement: Supplementary file 4 — Source Data Fig. 1 [file 44319_2023_33_MOESM4_ESM.zip › Fig.1/Fig. 1E/NORMAL MUSCLE/M4_Fig.tif]

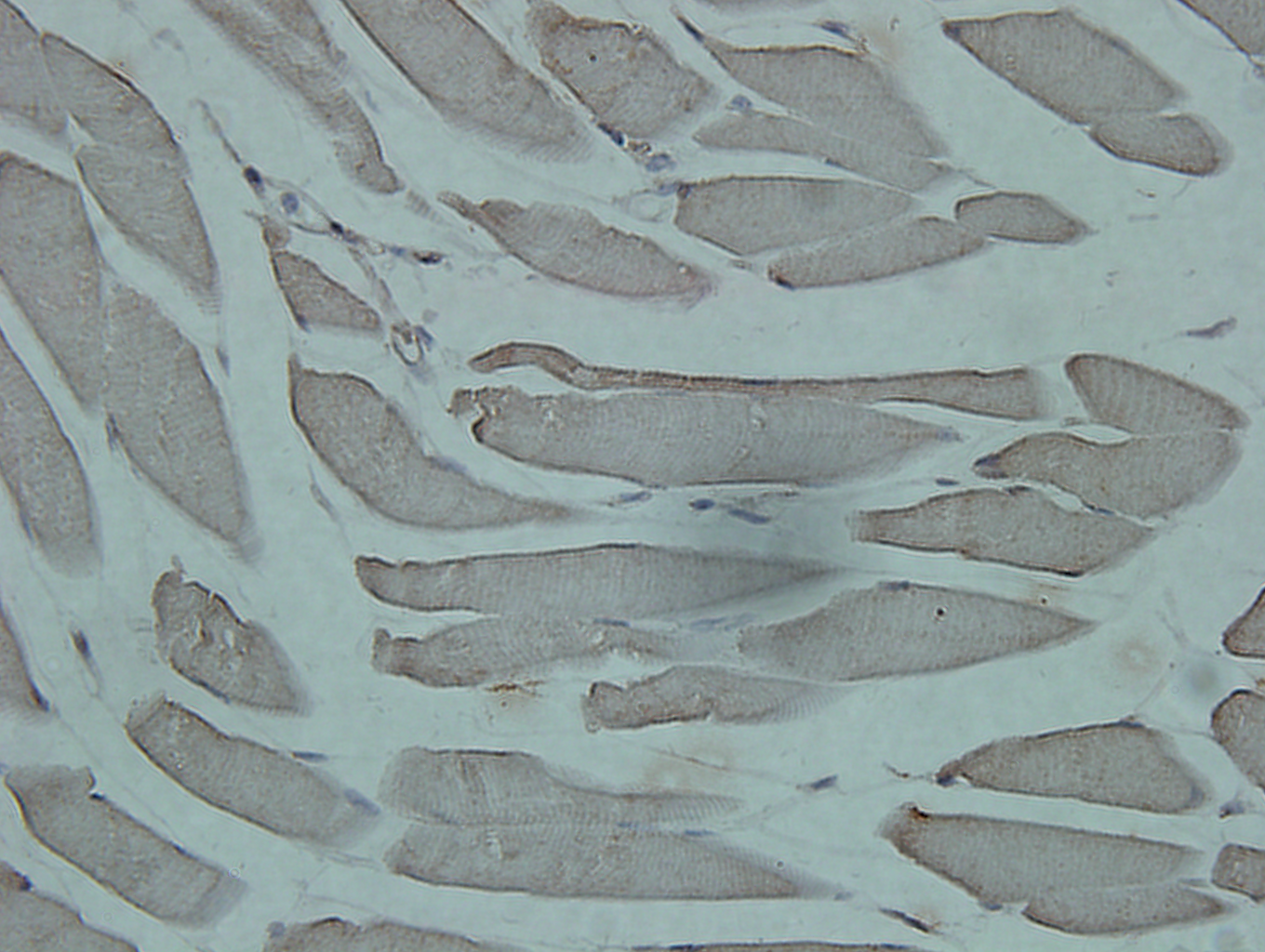

Supplement: Supplementary file 4 — Source Data Fig. 1 [file 44319_2023_33_MOESM4_ESM.zip › Fig.1/Fig. 1E/NORMAL MUSCLE/M6_Fig.tif]

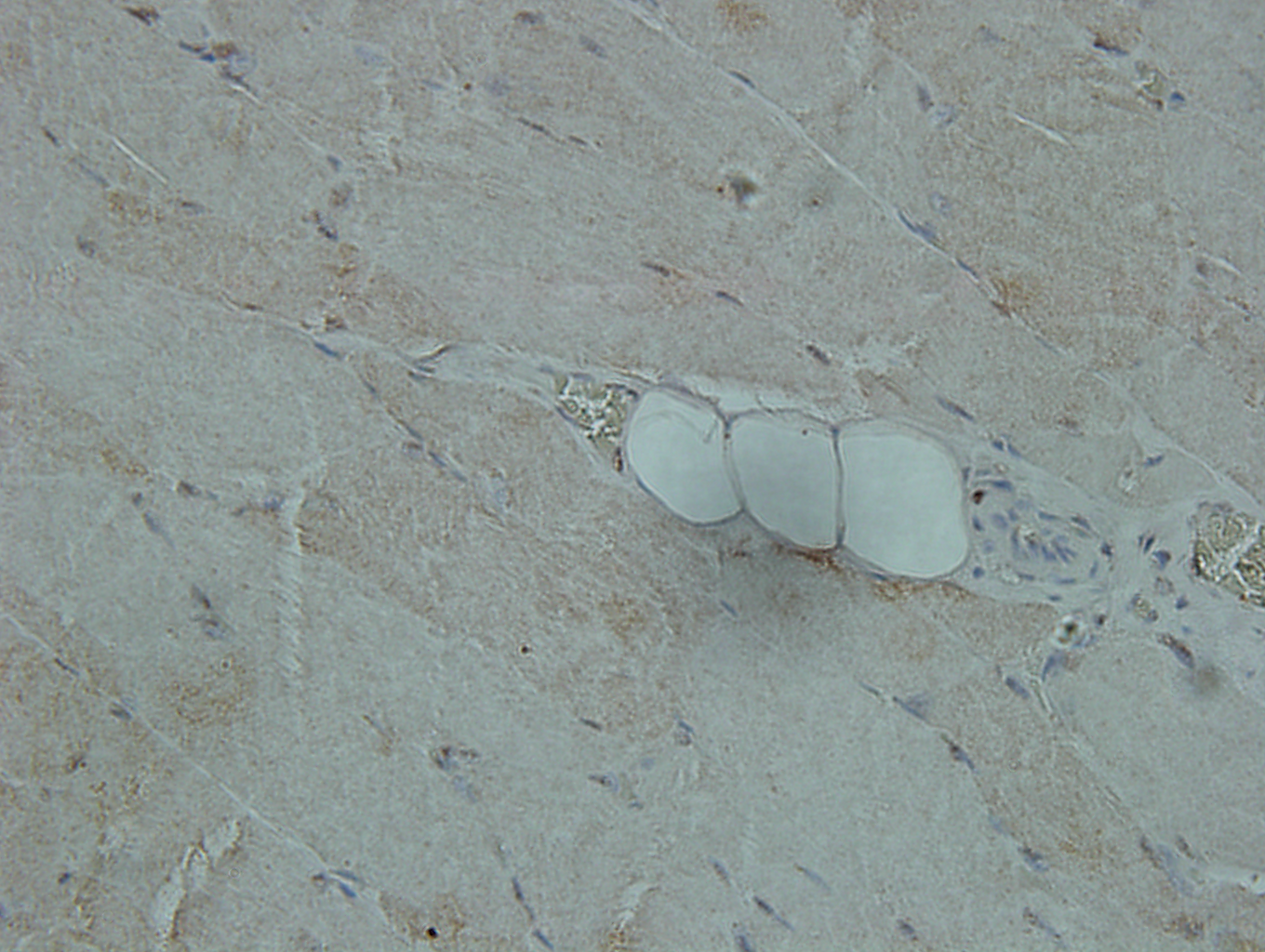

Supplement: Supplementary file 4 — Source Data Fig. 1 [file 44319_2023_33_MOESM4_ESM.zip › Fig.1/Fig. 1E/NORMAL MUSCLE/M7_Fig.tif]

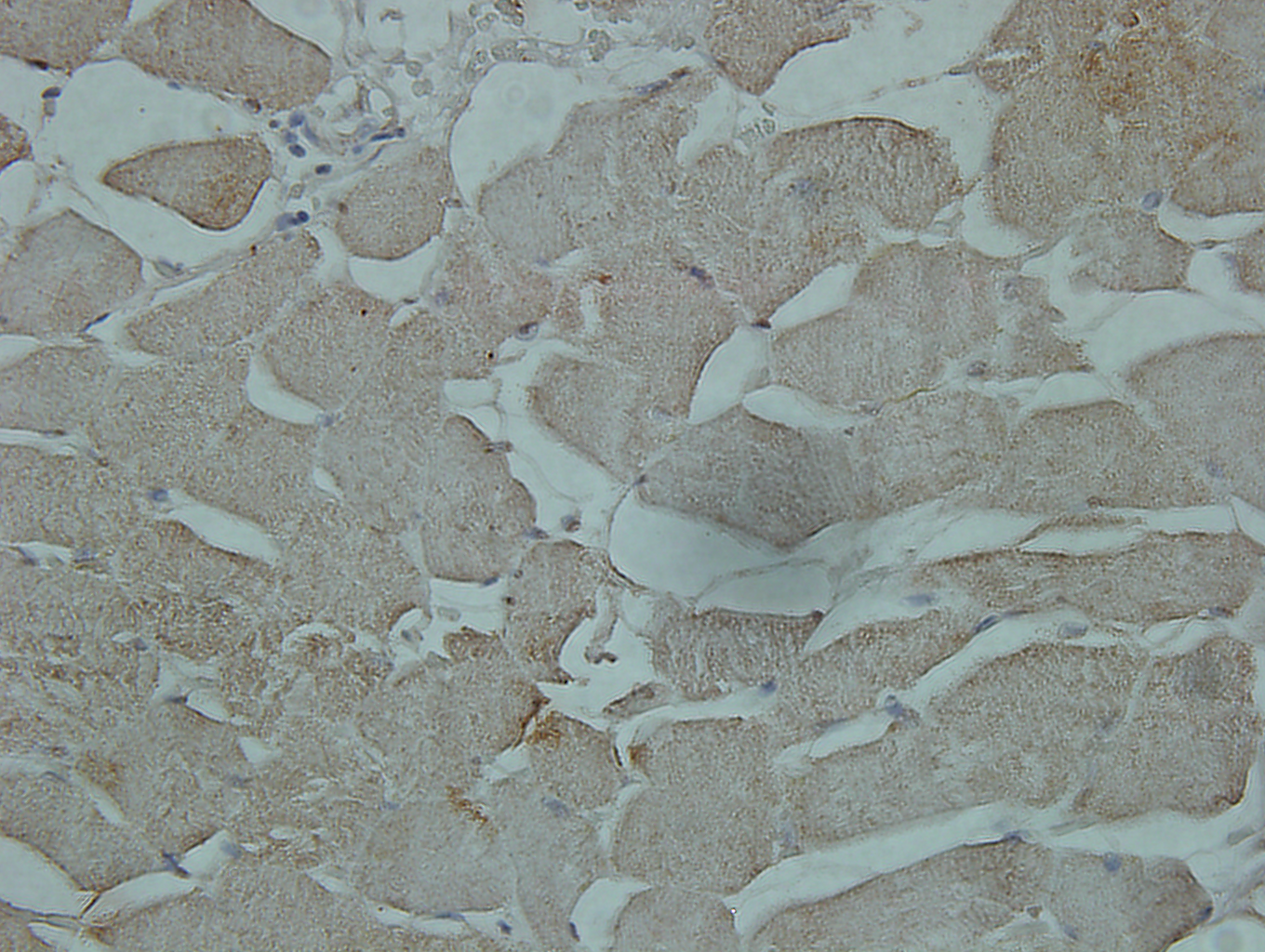

Supplement: Supplementary file 4 — Source Data Fig. 1 [file 44319_2023_33_MOESM4_ESM.zip › Fig.1/Fig. 1E/NORMAL MUSCLE/M2_Fig.tif]

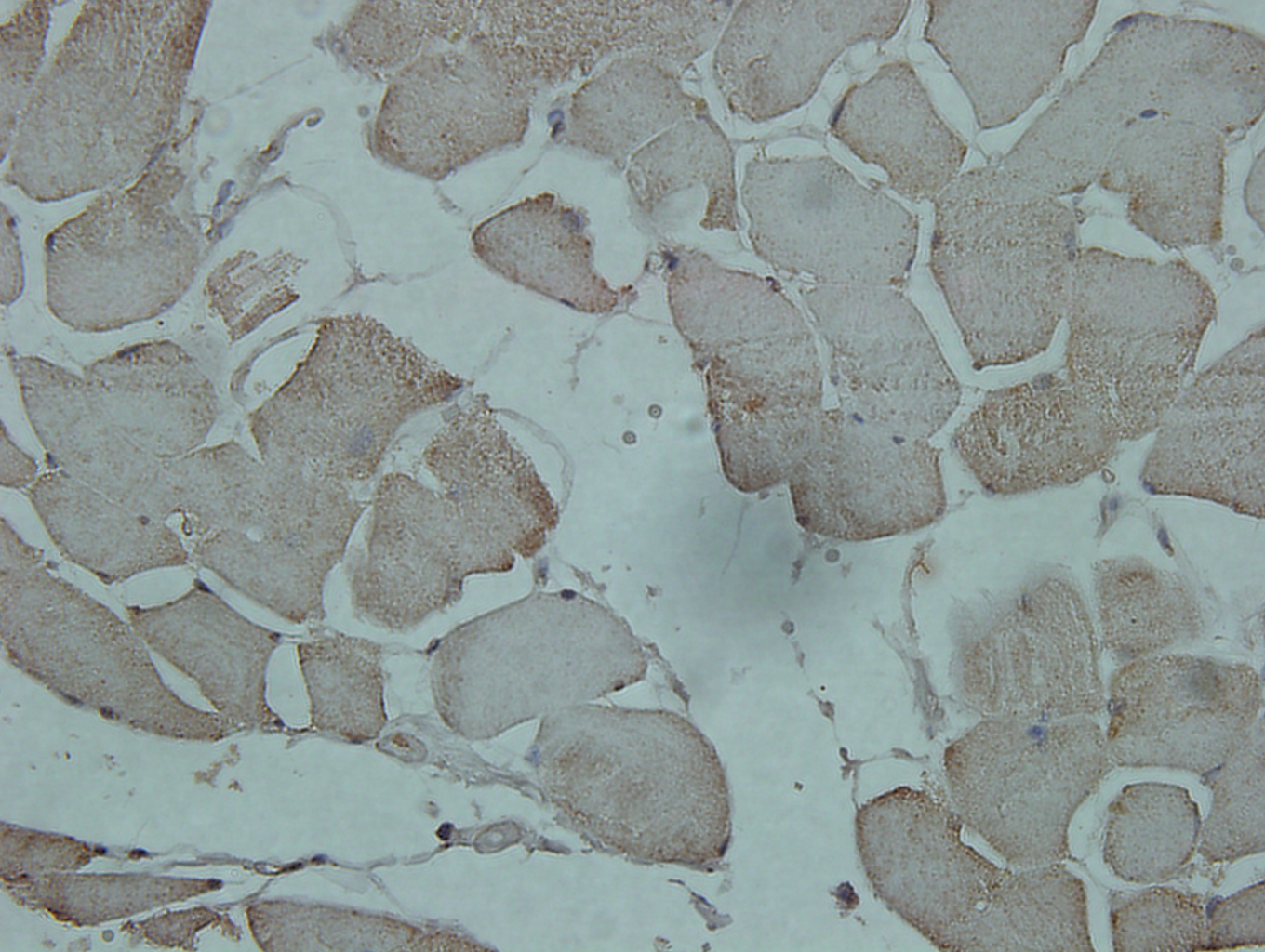

Supplement: Supplementary file 4 — Source Data Fig. 1 [file 44319_2023_33_MOESM4_ESM.zip › Fig.1/Fig. 1E/NORMAL MUSCLE/M3_Fig.tif]

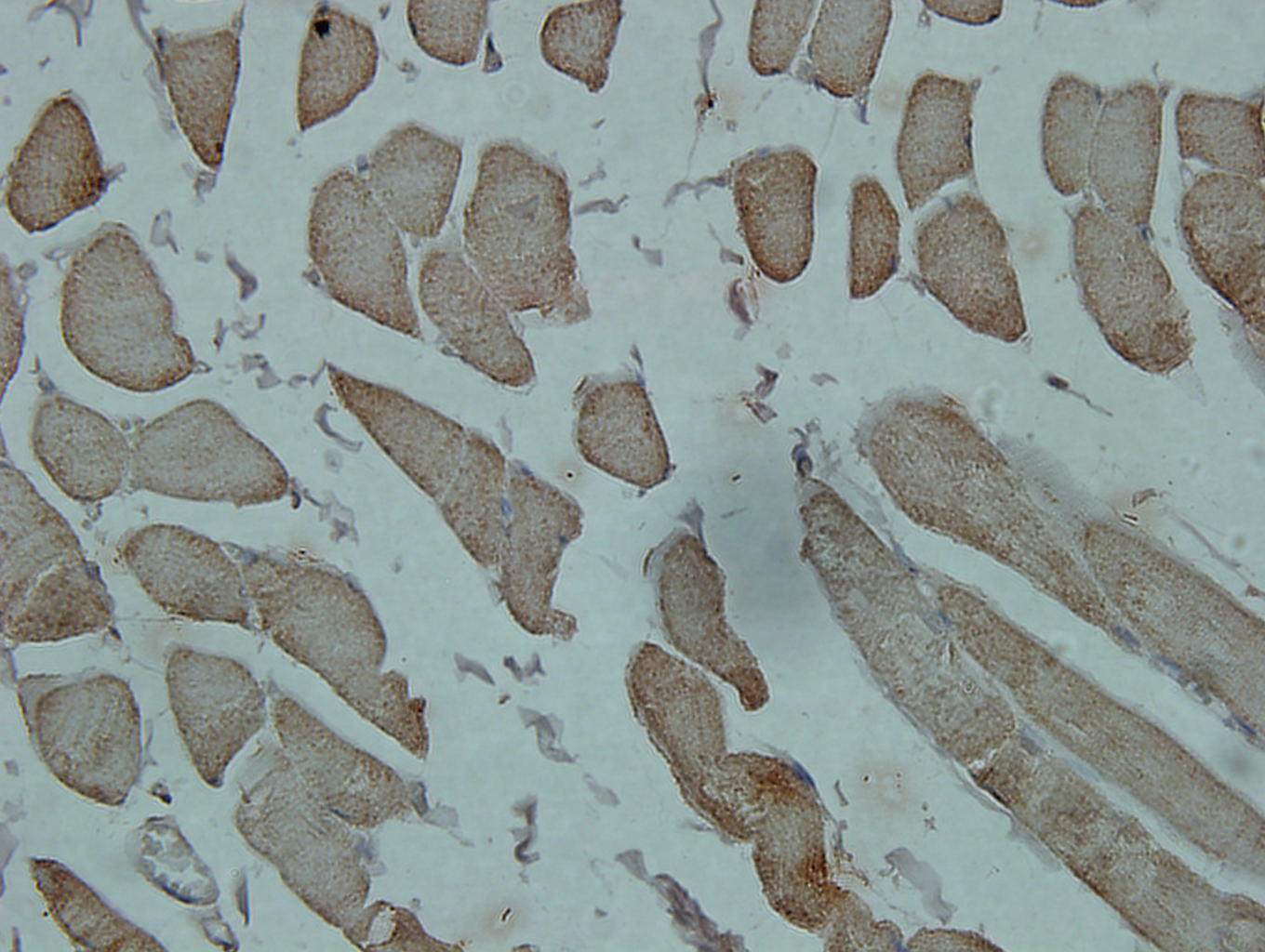

Supplement: Supplementary file 4 — Source Data Fig. 1 [file 44319_2023_33_MOESM4_ESM.zip › Fig.1/Fig. 1E/NORMAL MUSCLE/M8_Fig.tif]

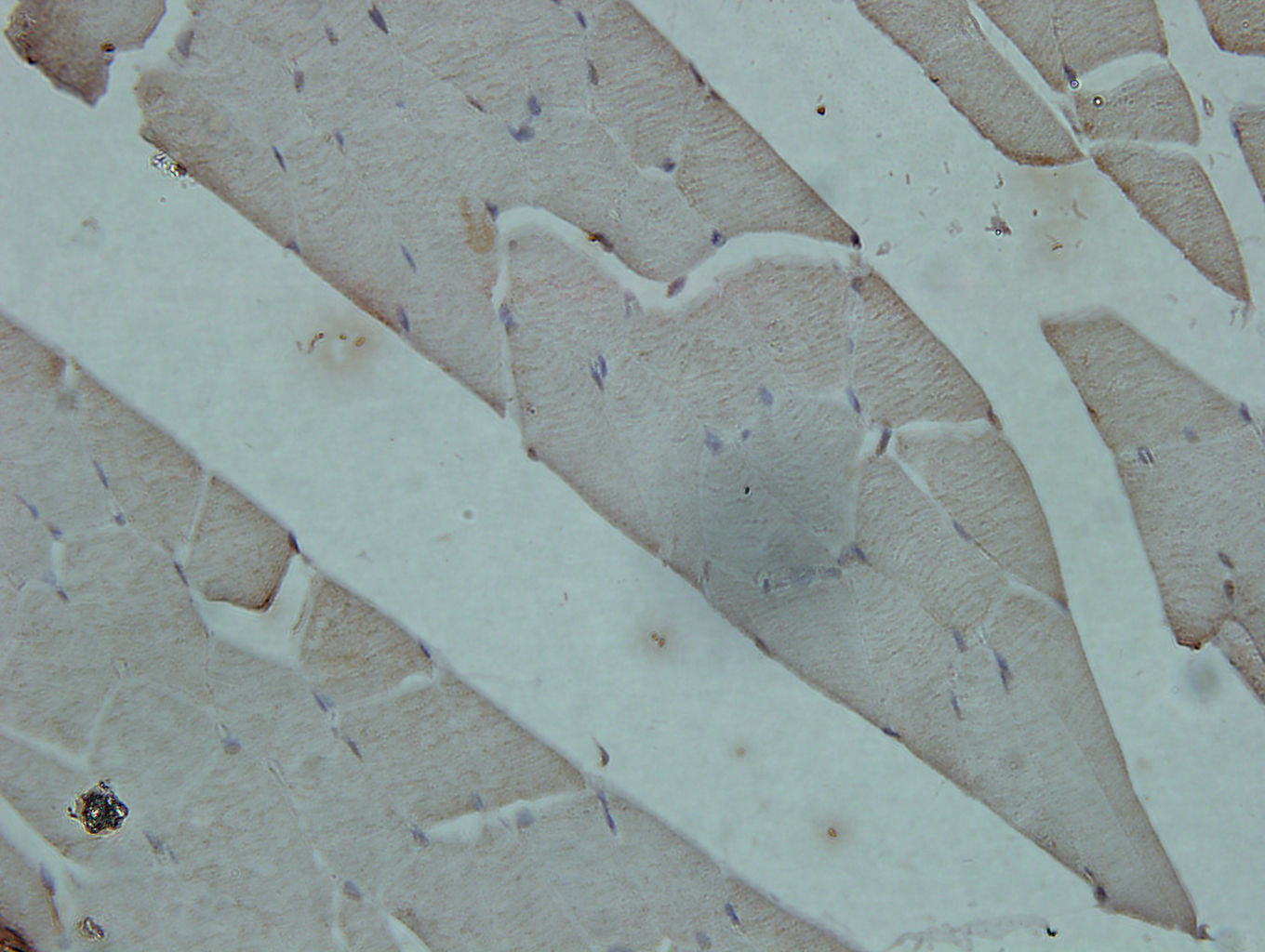

Supplement: Supplementary file 4 — Source Data Fig. 1 [file 44319_2023_33_MOESM4_ESM.zip › Fig.1/Fig. 1E/NORMAL MUSCLE/M1_Fig.tif]

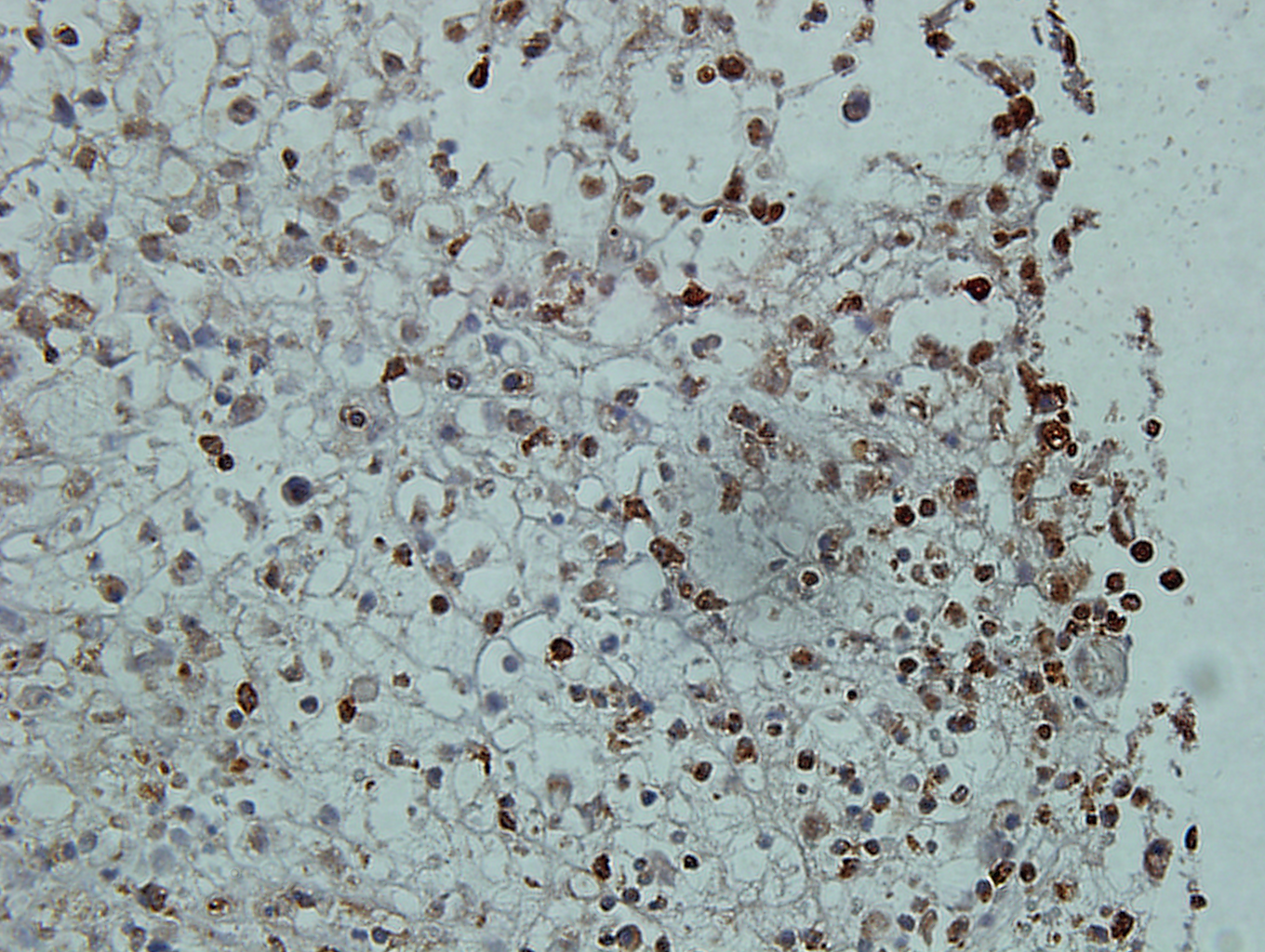

Supplement: Supplementary file 4 — Source Data Fig. 1 [file 44319_2023_33_MOESM4_ESM.zip › Fig.1/Fig. 1E/ERMS PATIENT TUMORS/E19_Fig.tif]

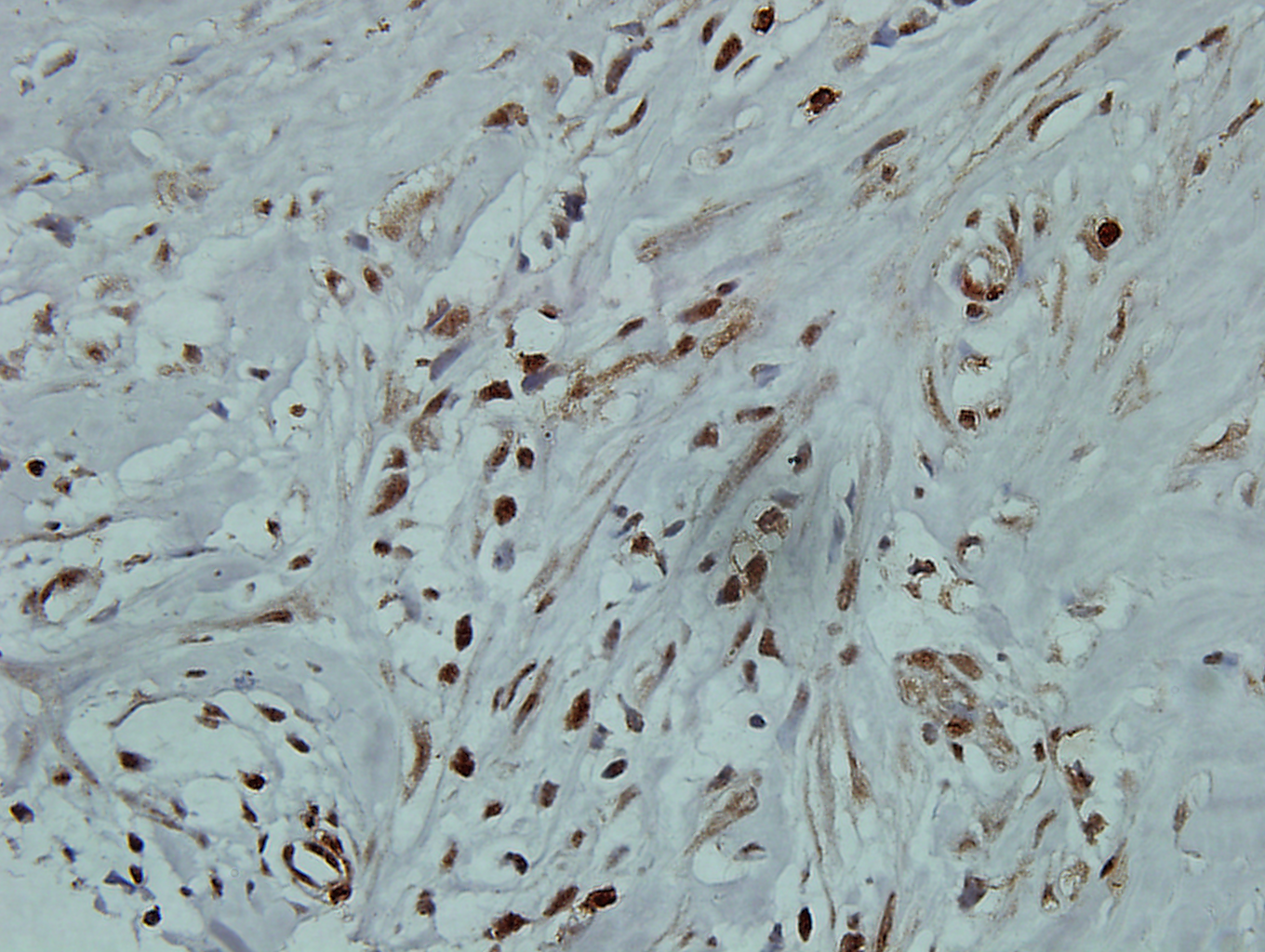

Supplement: Supplementary file 4 — Source Data Fig. 1 [file 44319_2023_33_MOESM4_ESM.zip › Fig.1/Fig. 1E/ERMS PATIENT TUMORS/E3_Fig.tif]

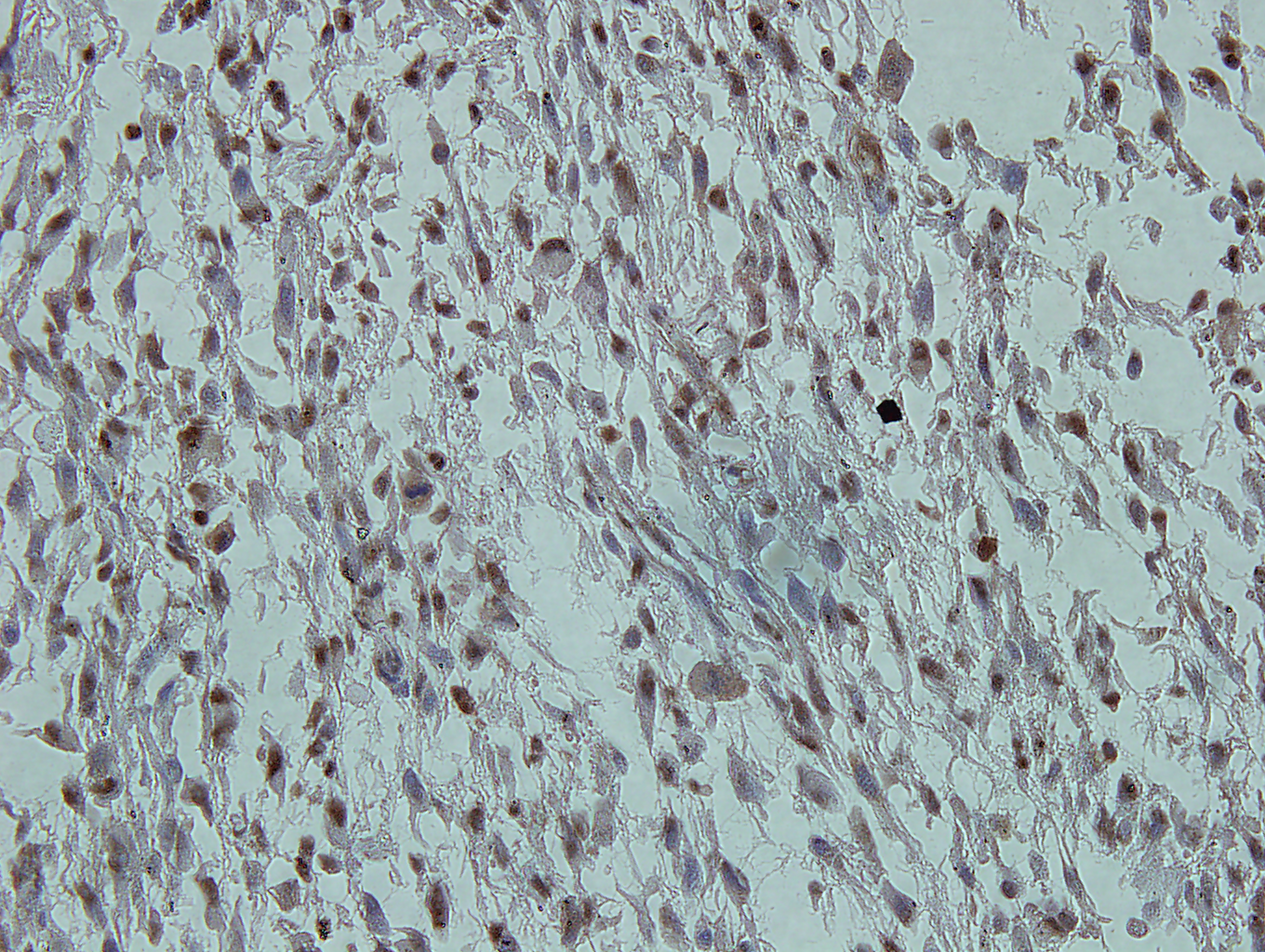

Supplement: Supplementary file 4 — Source Data Fig. 1 [file 44319_2023_33_MOESM4_ESM.zip › Fig.1/Fig. 1E/ERMS PATIENT TUMORS/E10_Fig.tif]

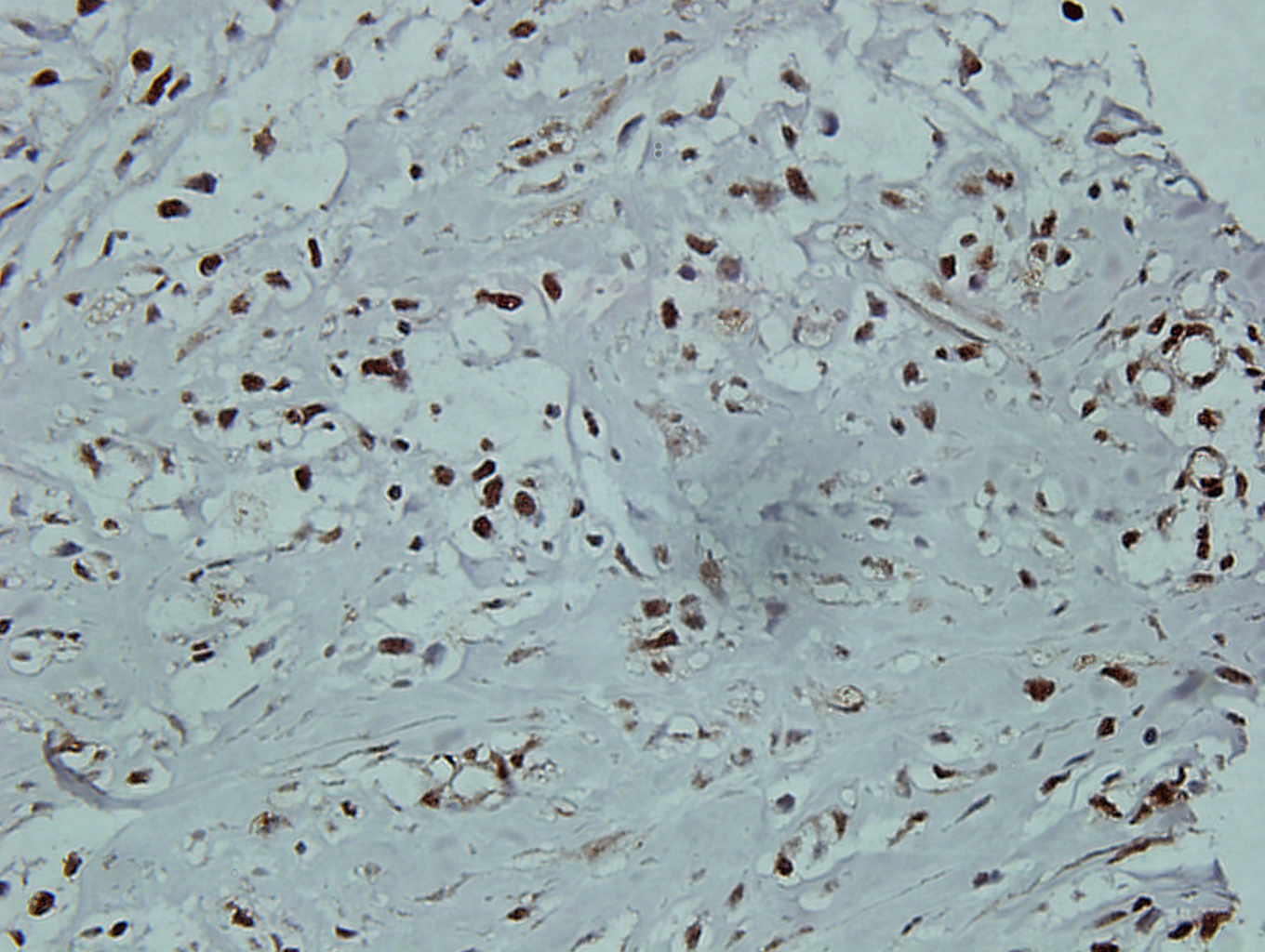

Supplement: Supplementary file 4 — Source Data Fig. 1 [file 44319_2023_33_MOESM4_ESM.zip › Fig.1/Fig. 1E/ERMS PATIENT TUMORS/E2_Fig.tif]

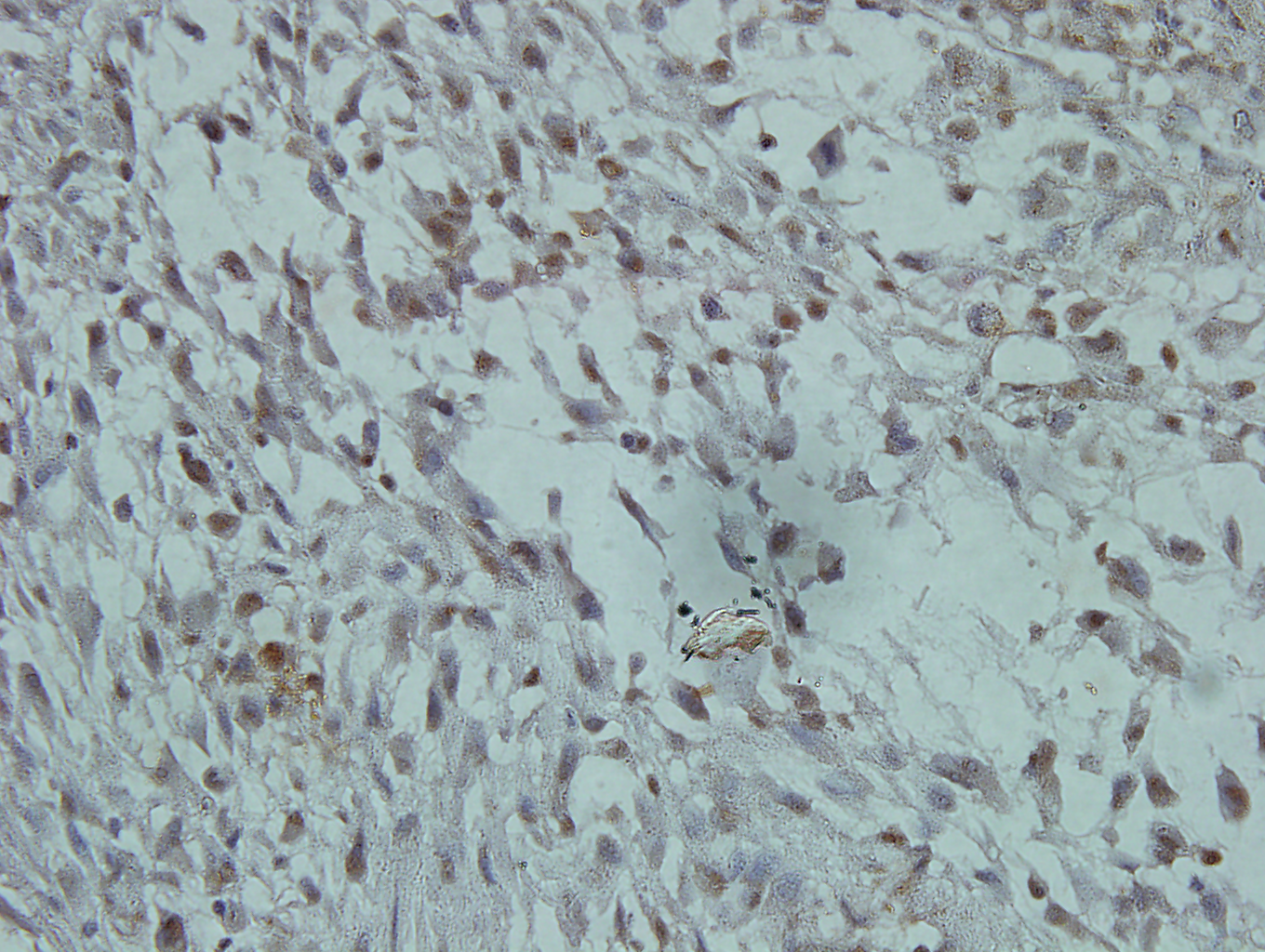

Supplement: Supplementary file 4 — Source Data Fig. 1 [file 44319_2023_33_MOESM4_ESM.zip › Fig.1/Fig. 1E/ERMS PATIENT TUMORS/E11_Fig.tif]

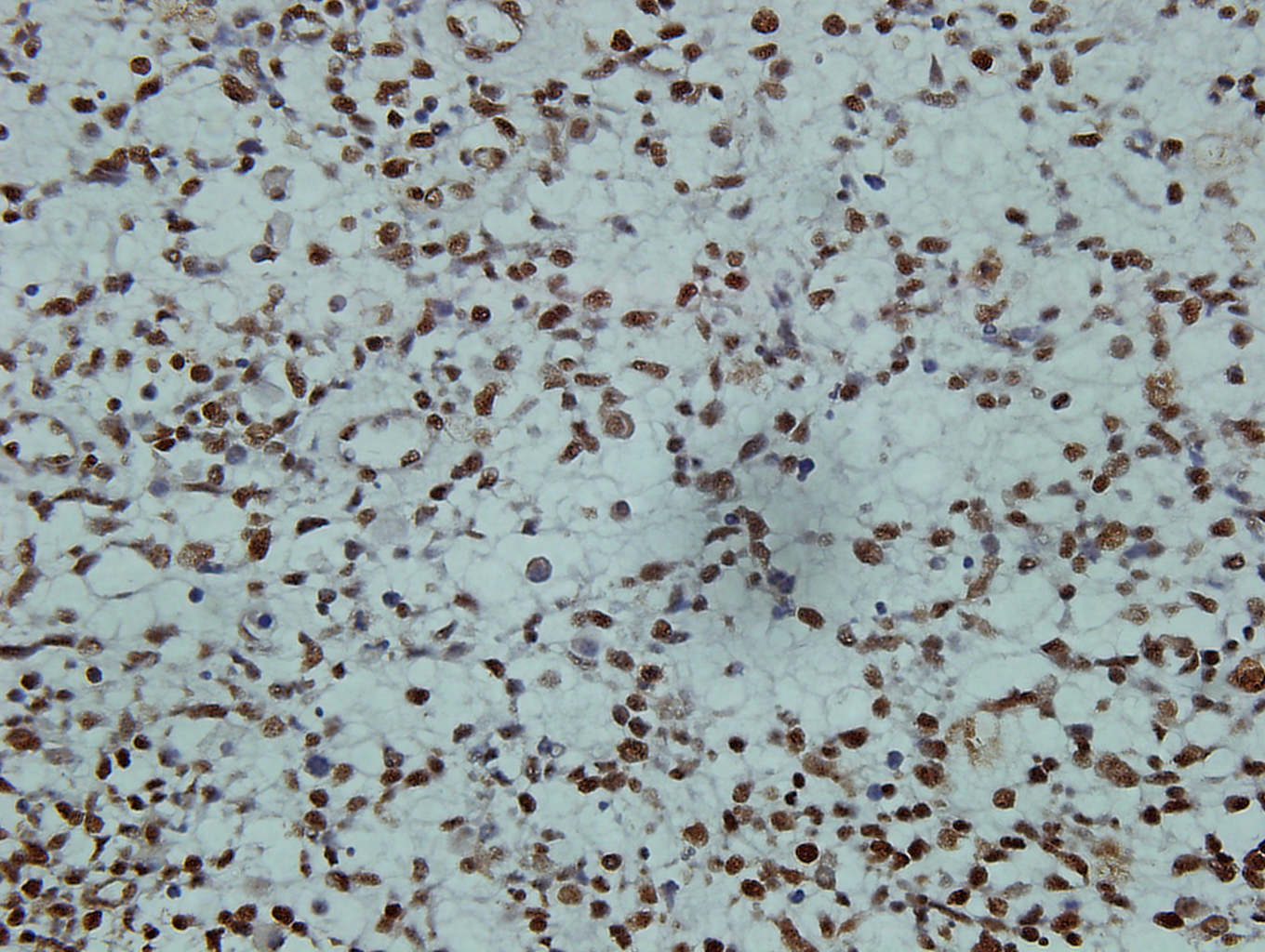

Supplement: Supplementary file 4 — Source Data Fig. 1 [file 44319_2023_33_MOESM4_ESM.zip › Fig.1/Fig. 1E/ERMS PATIENT TUMORS/E18_Fig.tif]

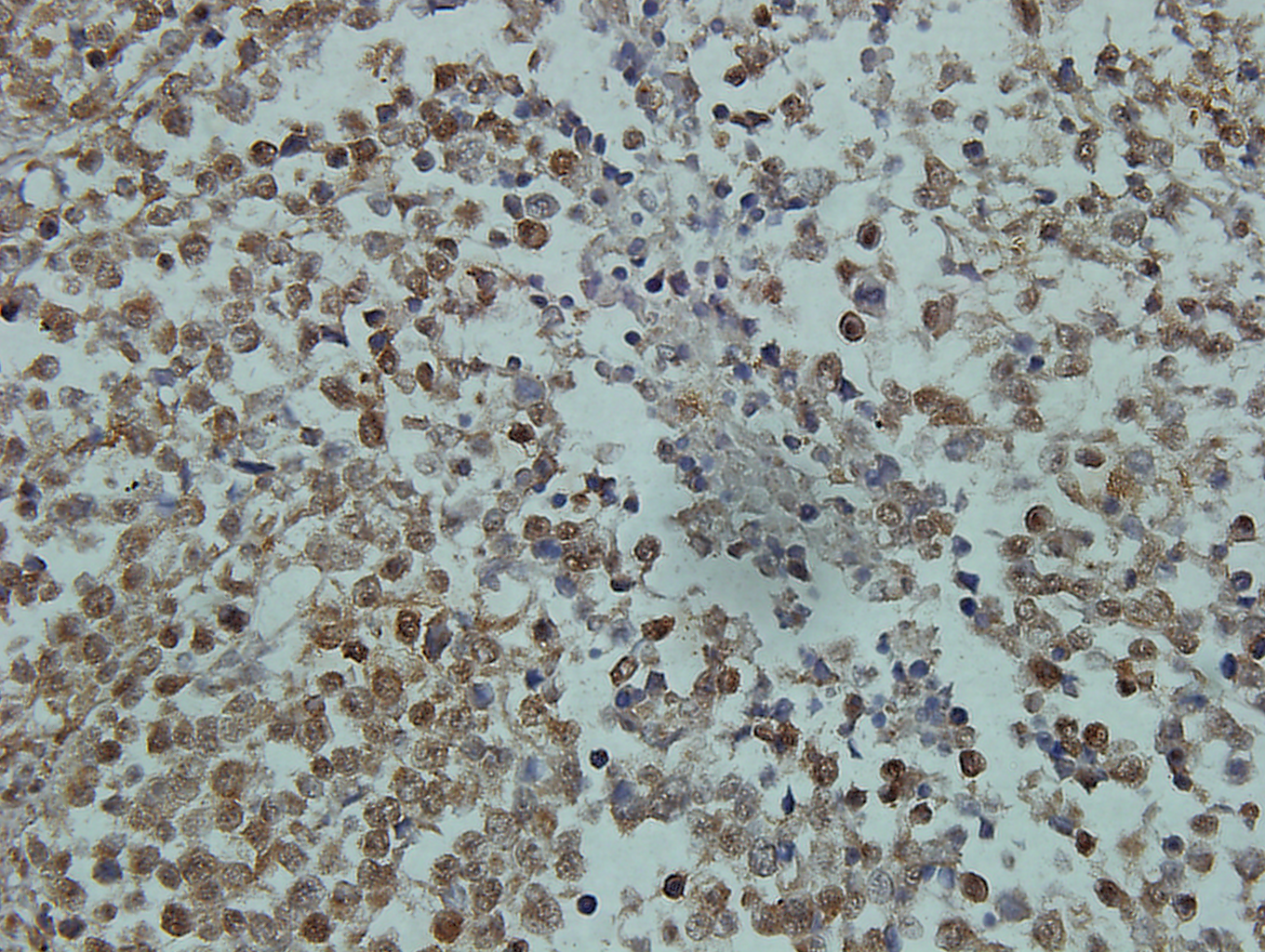

Supplement: Supplementary file 4 — Source Data Fig. 1 [file 44319_2023_33_MOESM4_ESM.zip › Fig.1/Fig. 1E/ERMS PATIENT TUMORS/E13_Fig.tif]

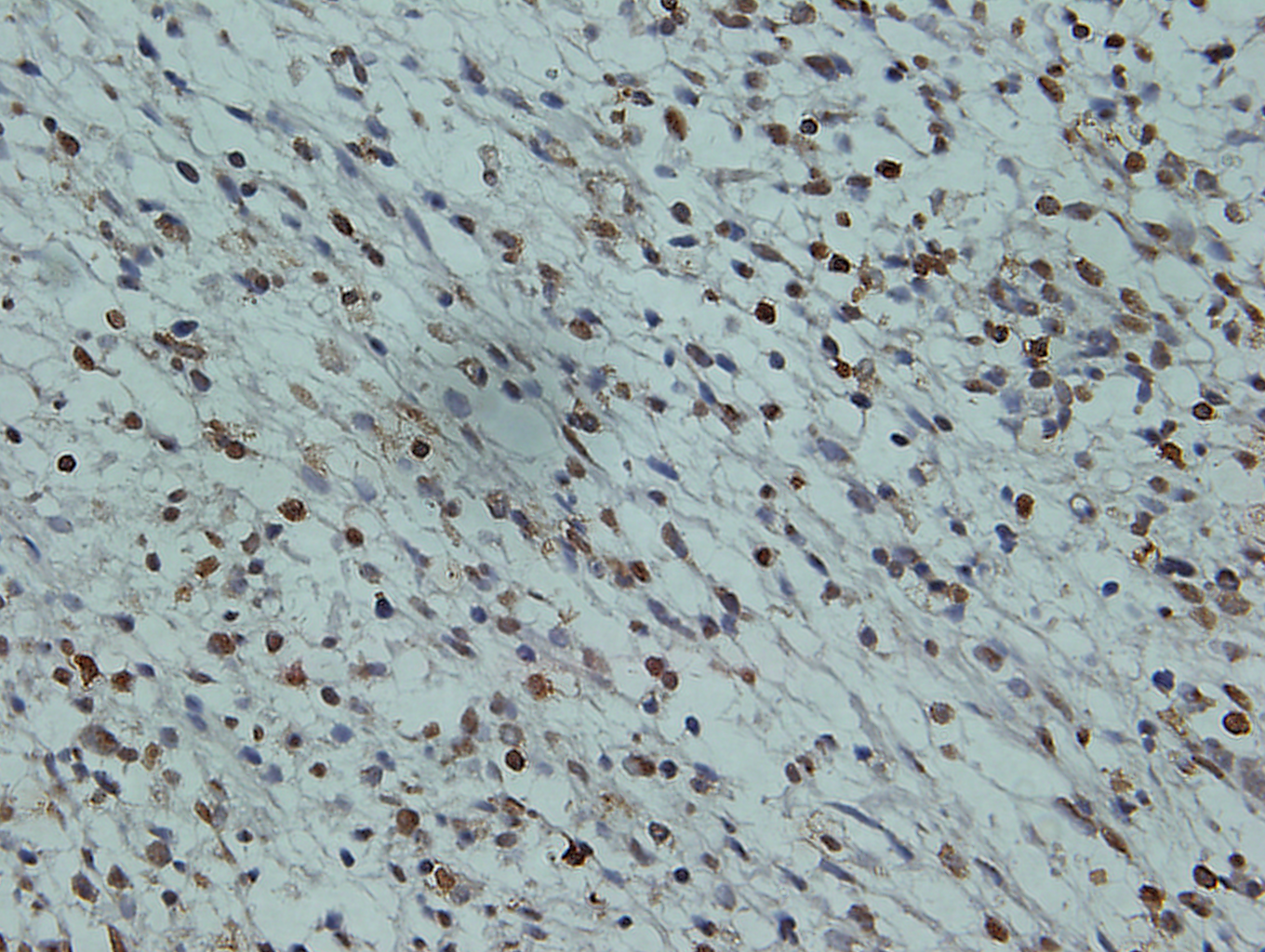

Supplement: Supplementary file 4 — Source Data Fig. 1 [file 44319_2023_33_MOESM4_ESM.zip › Fig.1/Fig. 1E/ERMS PATIENT TUMORS/E9_Fig.tif]

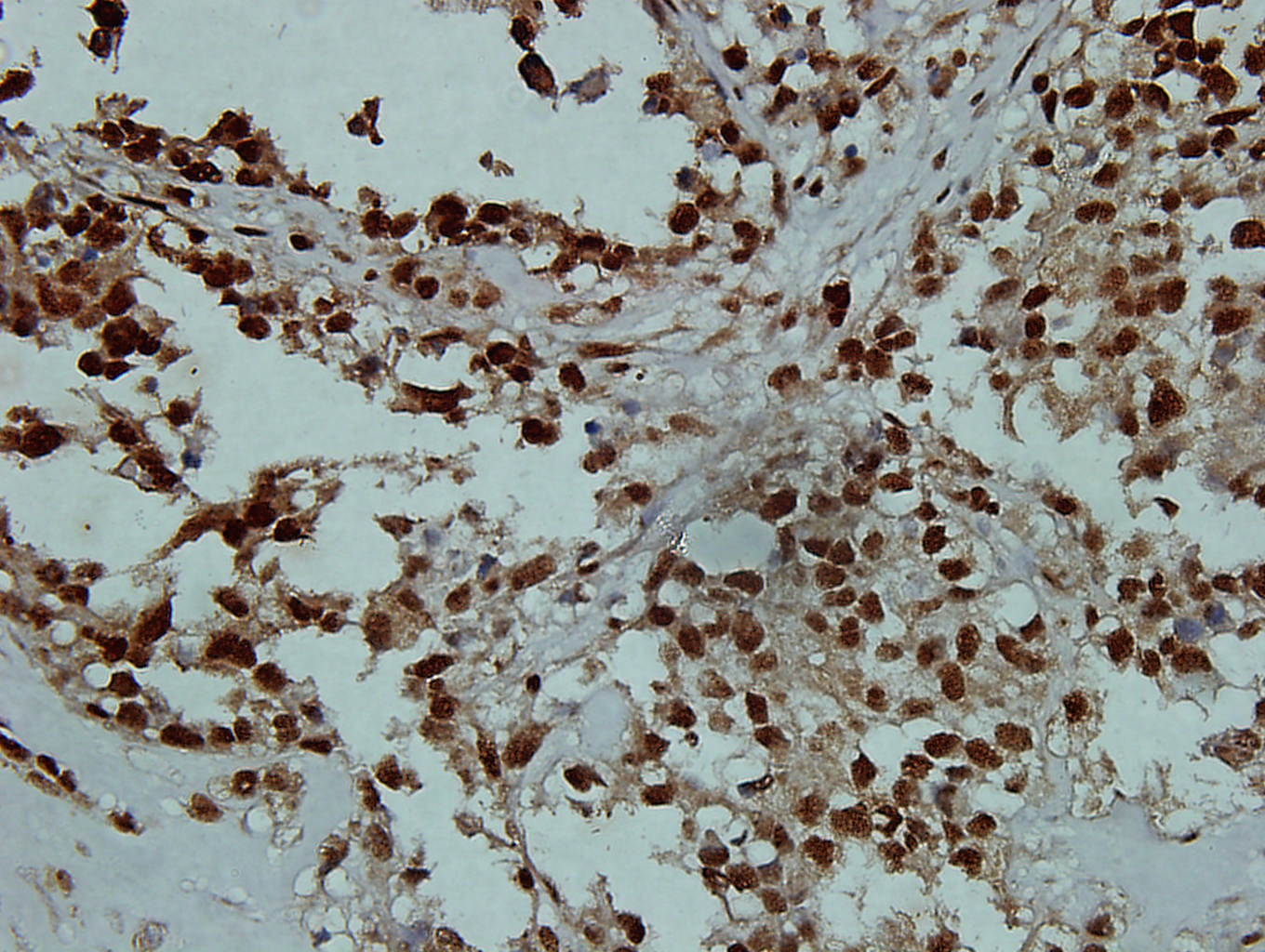

Supplement: Supplementary file 4 — Source Data Fig. 1 [file 44319_2023_33_MOESM4_ESM.zip › Fig.1/Fig. 1E/ERMS PATIENT TUMORS/E8_Fig.tif]

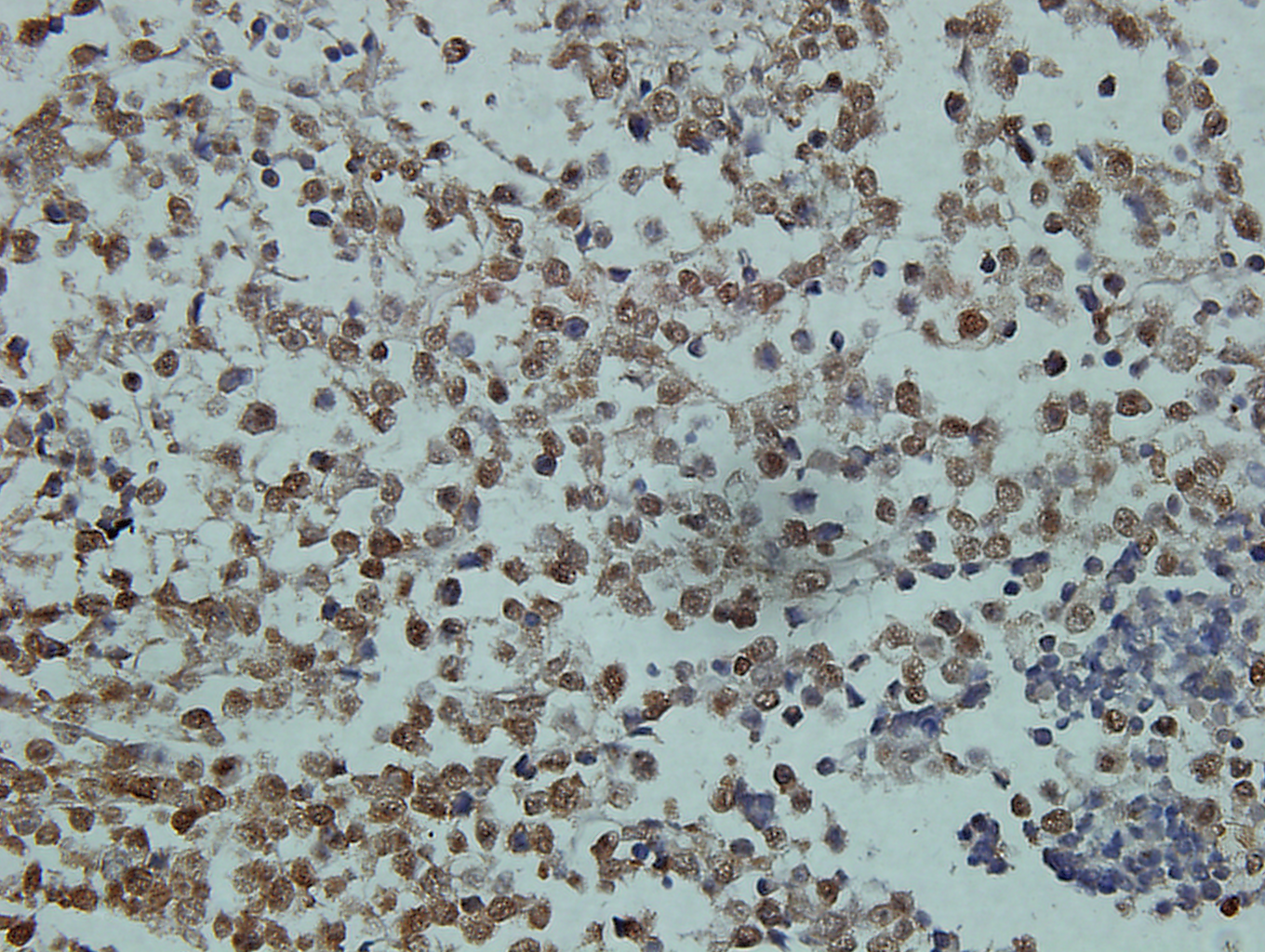

Supplement: Supplementary file 4 — Source Data Fig. 1 [file 44319_2023_33_MOESM4_ESM.zip › Fig.1/Fig. 1E/ERMS PATIENT TUMORS/E12_Fig.tif]

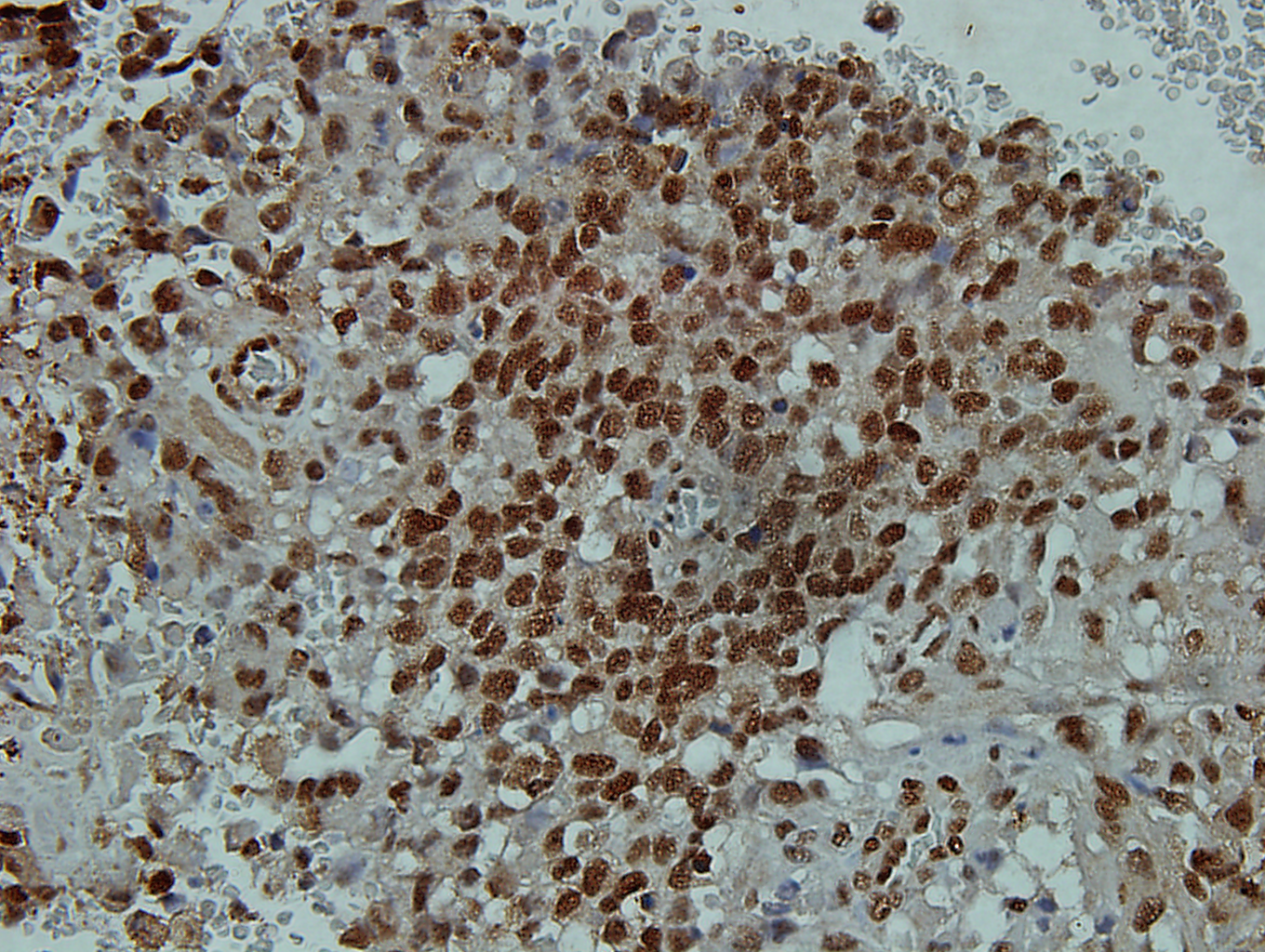

Supplement: Supplementary file 4 — Source Data Fig. 1 [file 44319_2023_33_MOESM4_ESM.zip › Fig.1/Fig. 1E/ERMS PATIENT TUMORS/E1_Fig.tif]

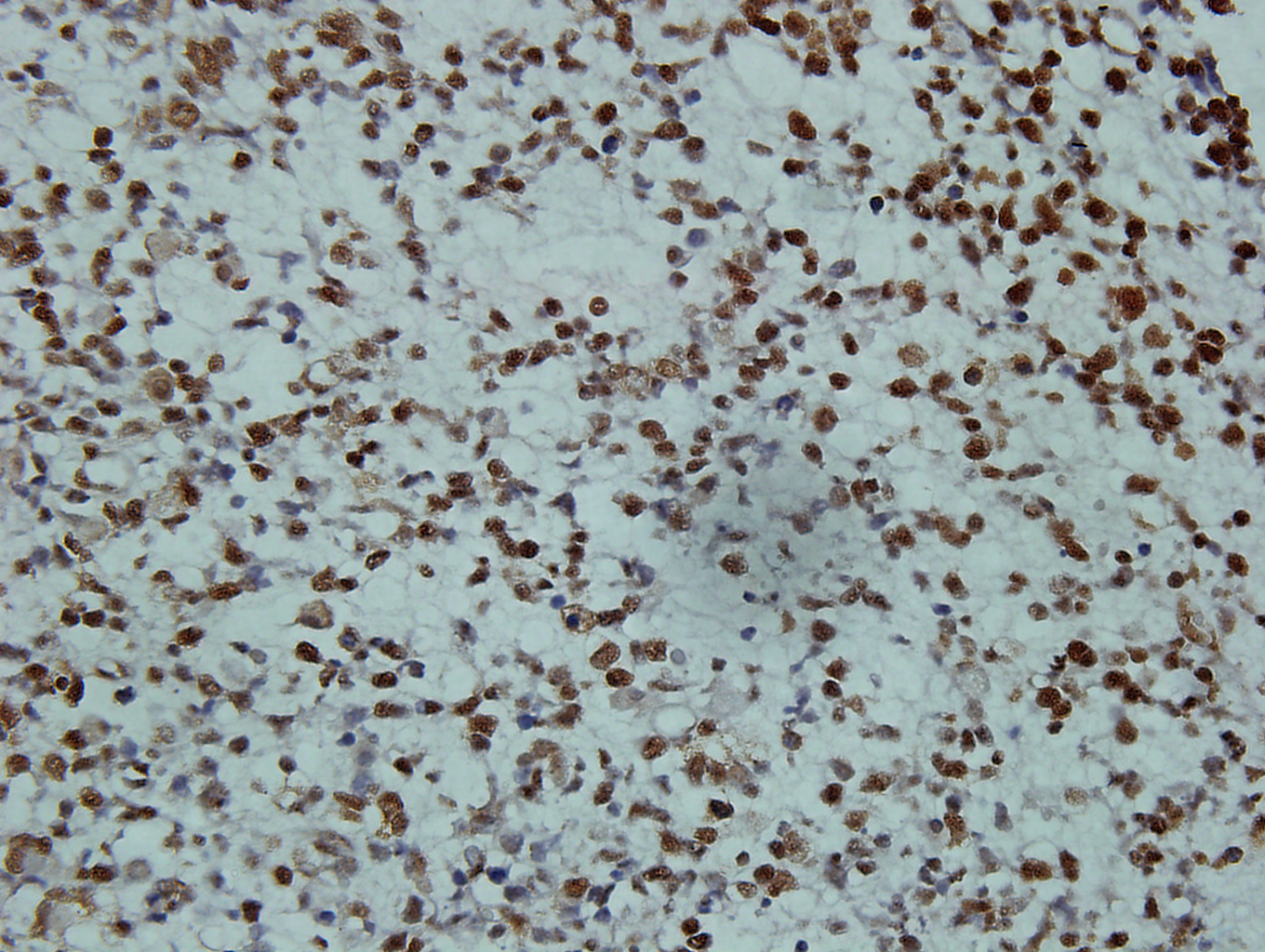

Supplement: Supplementary file 4 — Source Data Fig. 1 [file 44319_2023_33_MOESM4_ESM.zip › Fig.1/Fig. 1E/ERMS PATIENT TUMORS/E17_Fig.tif]

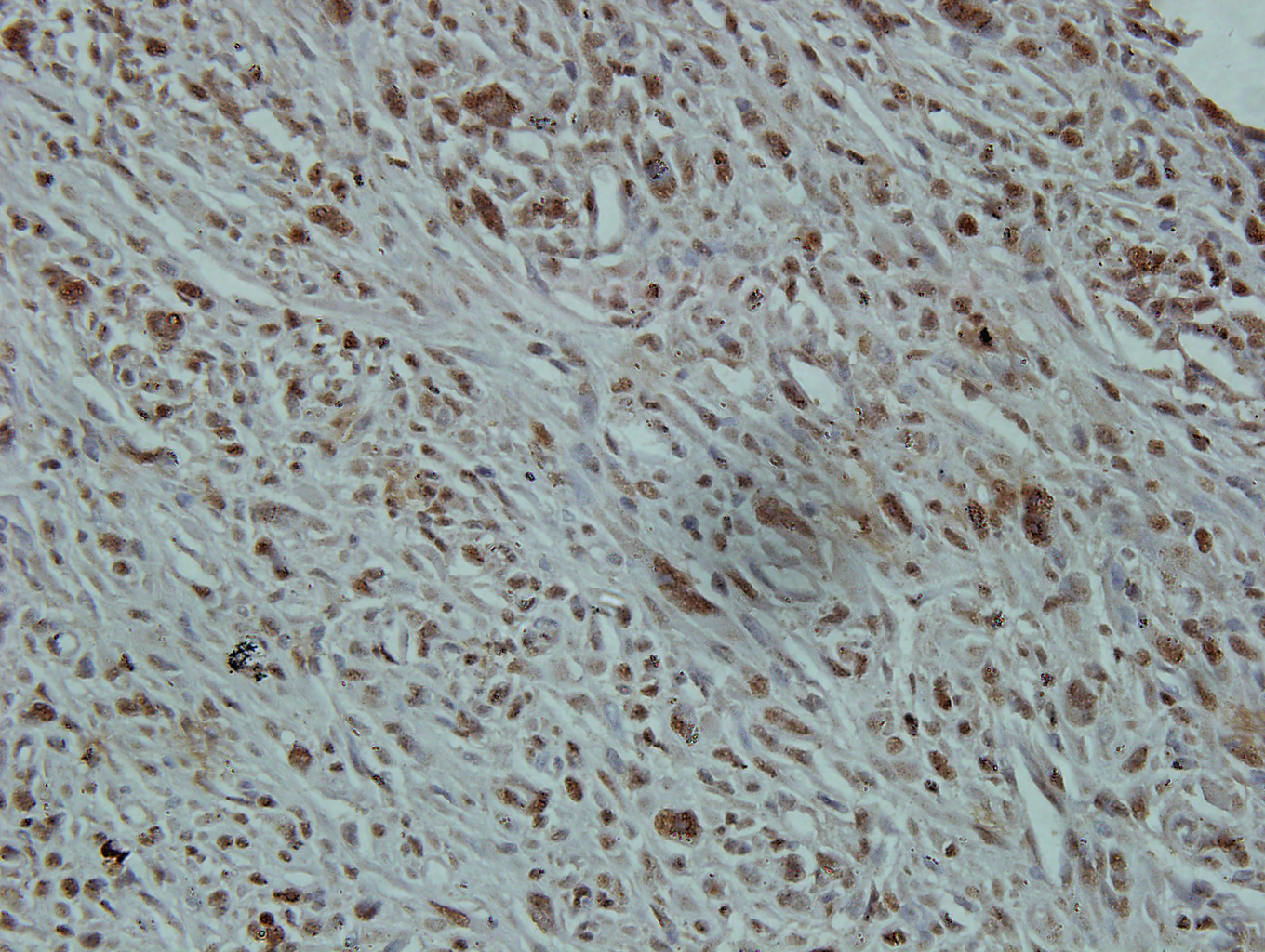

Supplement: Supplementary file 4 — Source Data Fig. 1 [file 44319_2023_33_MOESM4_ESM.zip › Fig.1/Fig. 1E/ERMS PATIENT TUMORS/E4_Fig.tif]

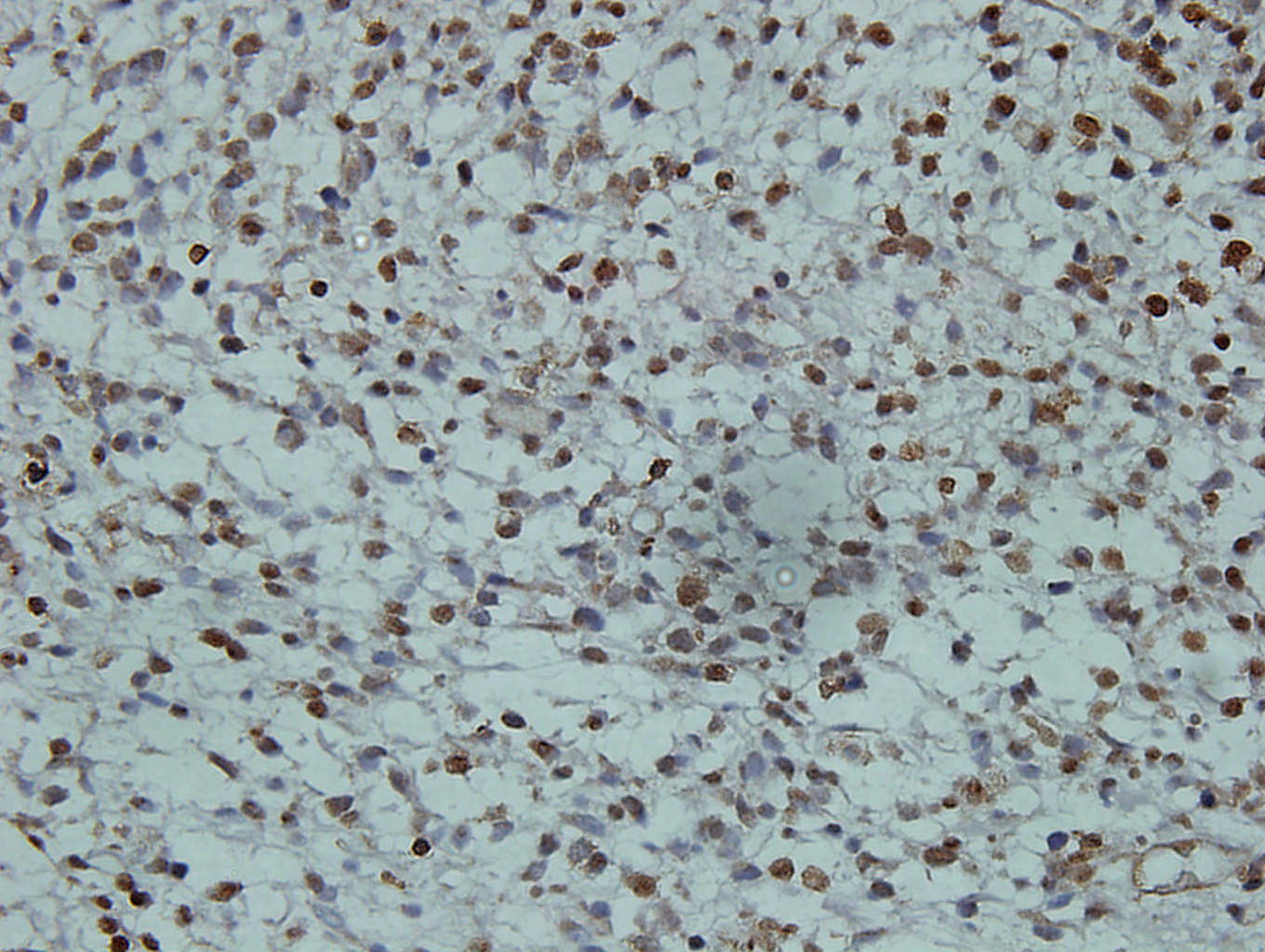

Supplement: Supplementary file 4 — Source Data Fig. 1 [file 44319_2023_33_MOESM4_ESM.zip › Fig.1/Fig. 1E/ERMS PATIENT TUMORS/E21_Fig.tif]

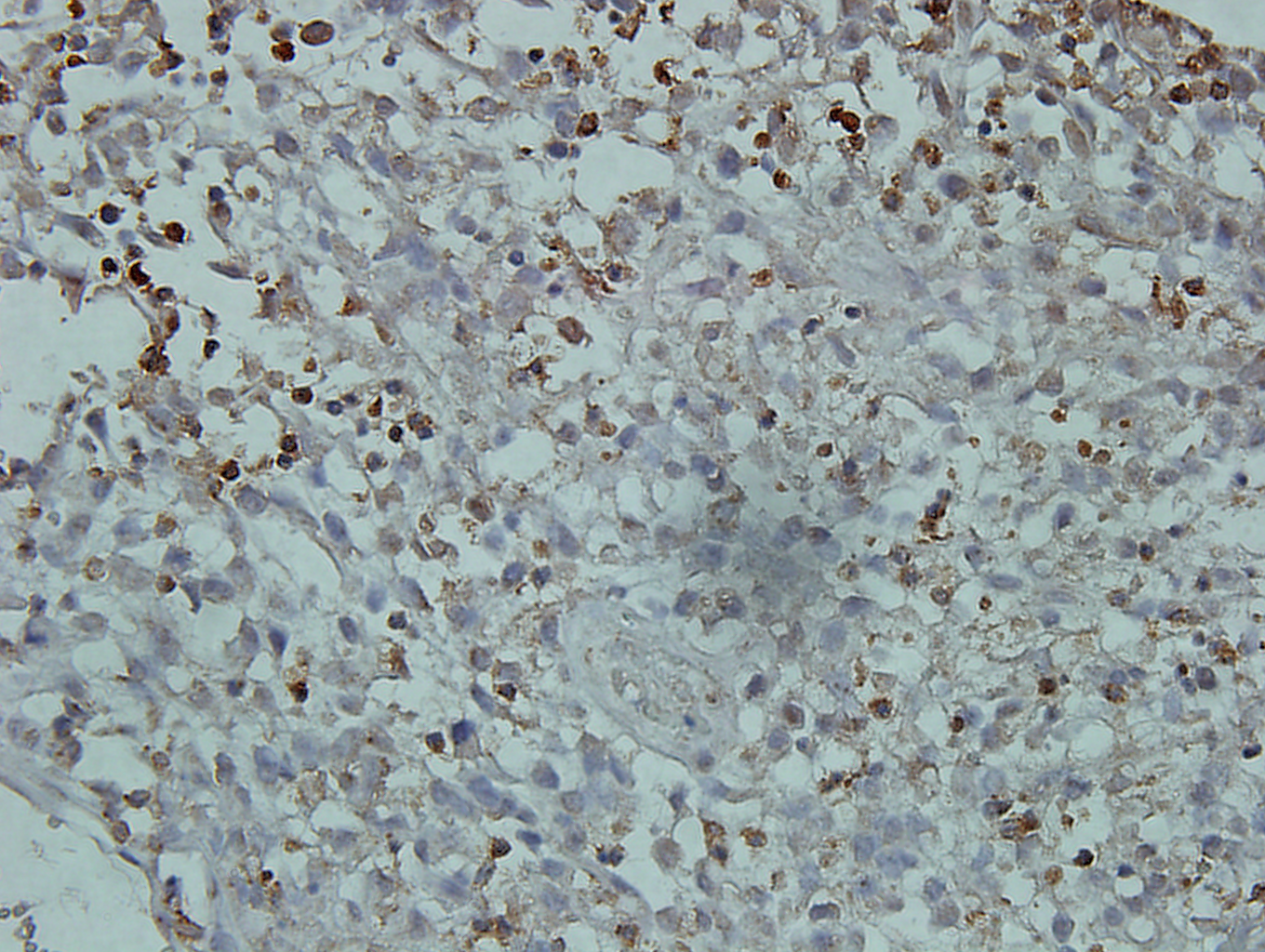

Supplement: Supplementary file 4 — Source Data Fig. 1 [file 44319_2023_33_MOESM4_ESM.zip › Fig.1/Fig. 1E/ERMS PATIENT TUMORS/E20_Fig.tif]

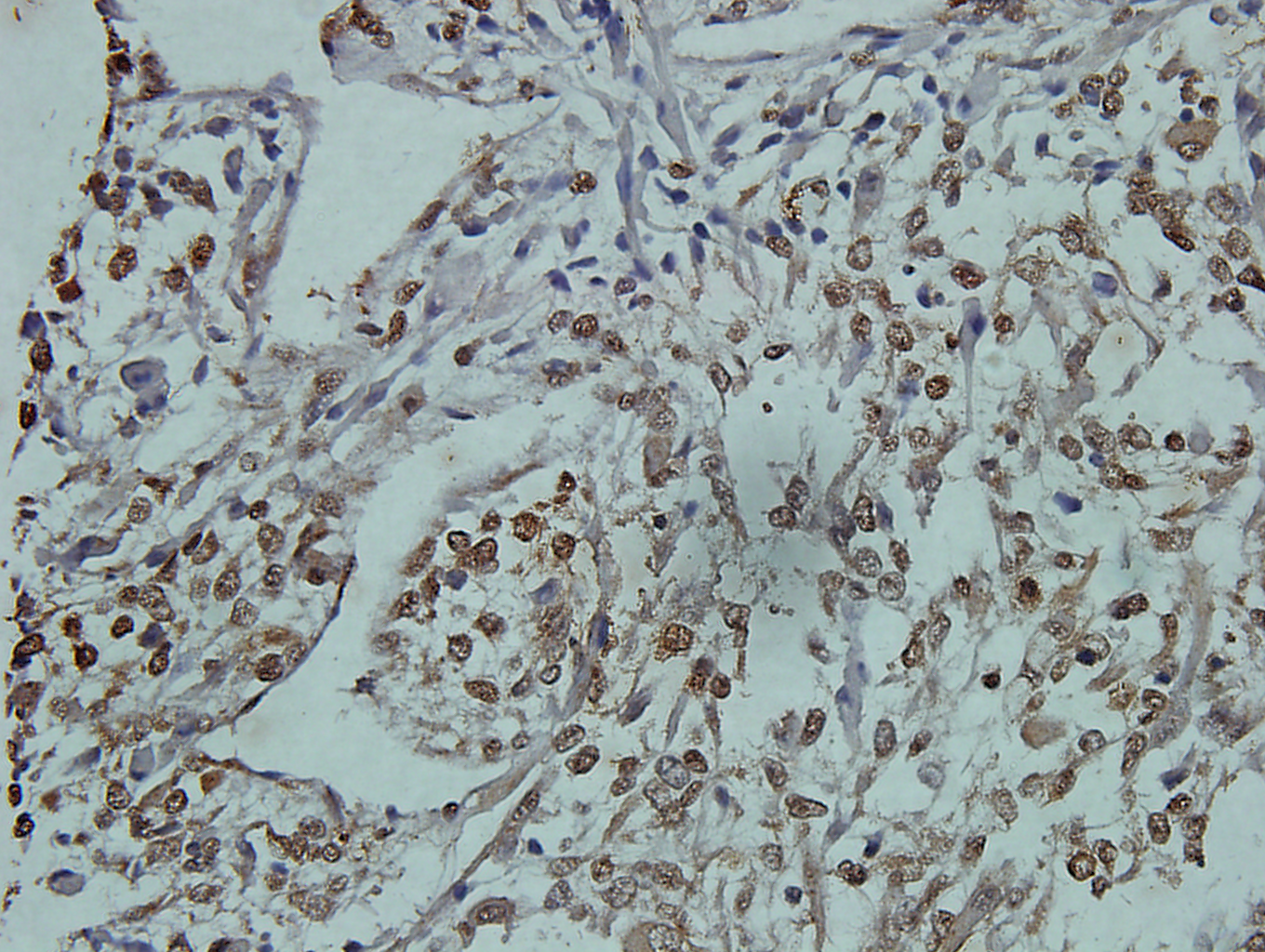

Supplement: Supplementary file 4 — Source Data Fig. 1 [file 44319_2023_33_MOESM4_ESM.zip › Fig.1/Fig. 1E/ERMS PATIENT TUMORS/E16_Fig.tif]

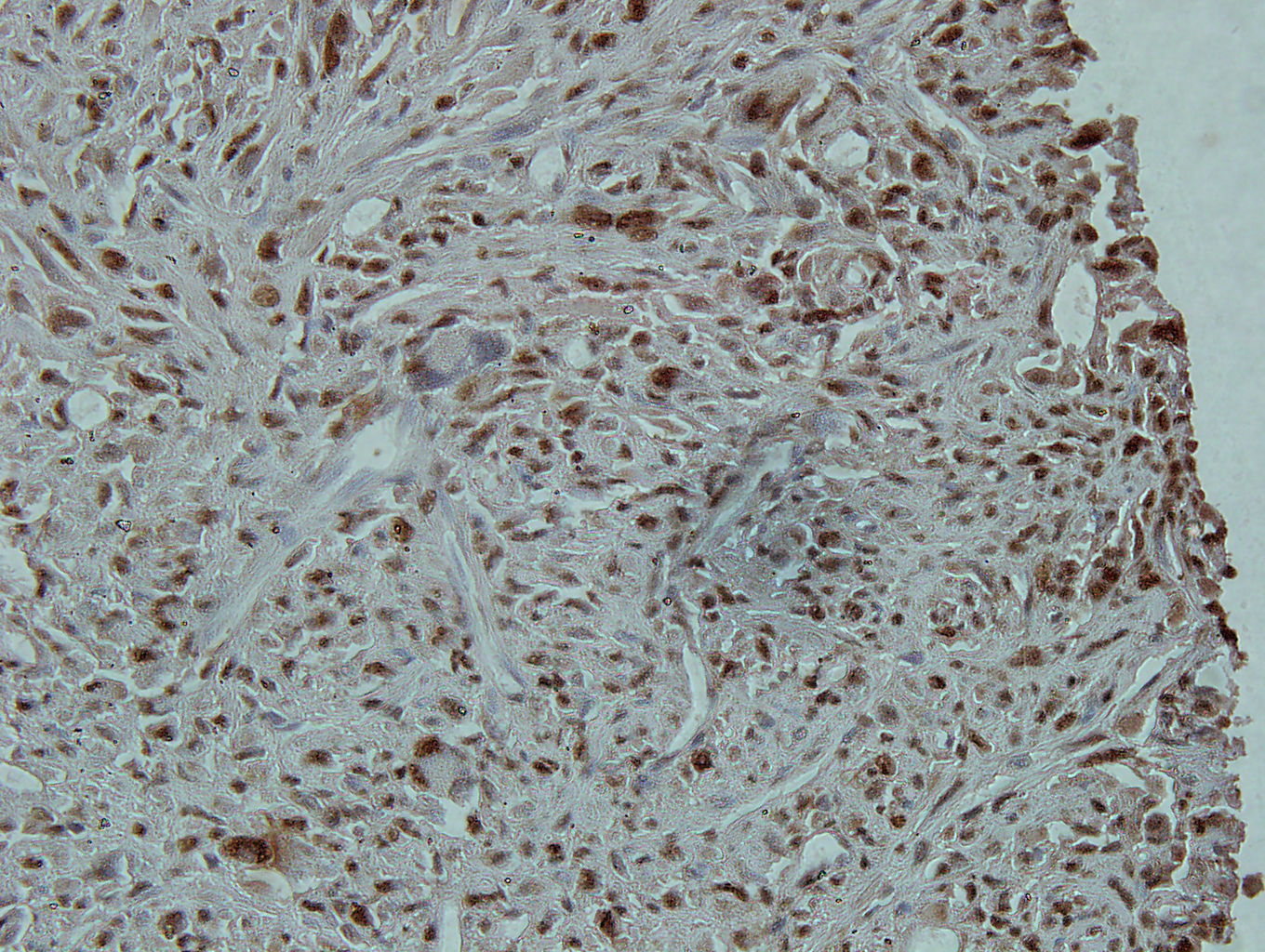

Supplement: Supplementary file 4 — Source Data Fig. 1 [file 44319_2023_33_MOESM4_ESM.zip › Fig.1/Fig. 1E/ERMS PATIENT TUMORS/E5_Fig.tif]

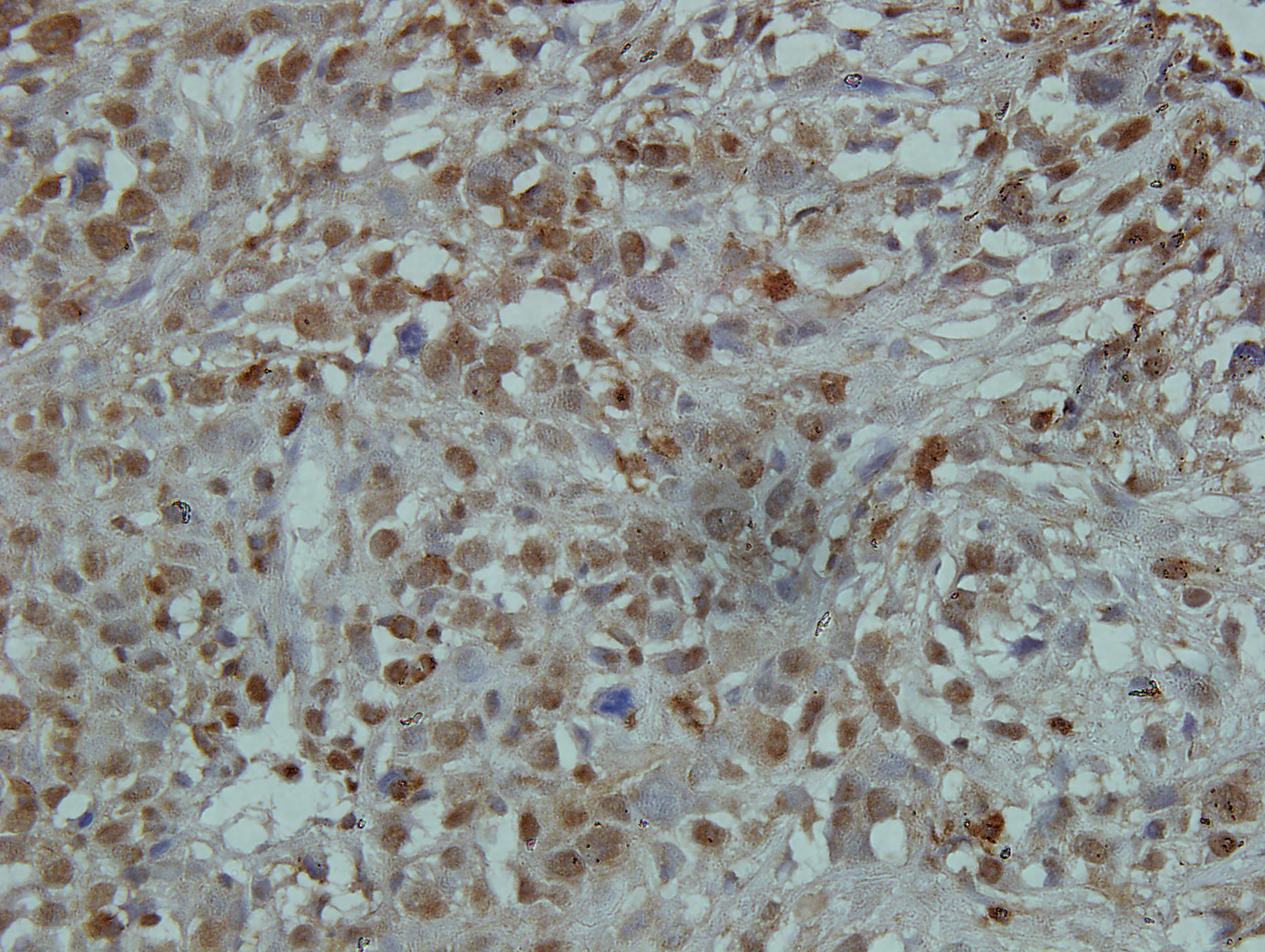

Supplement: Supplementary file 4 — Source Data Fig. 1 [file 44319_2023_33_MOESM4_ESM.zip › Fig.1/Fig. 1E/ERMS PATIENT TUMORS/E7_Fig.tif]

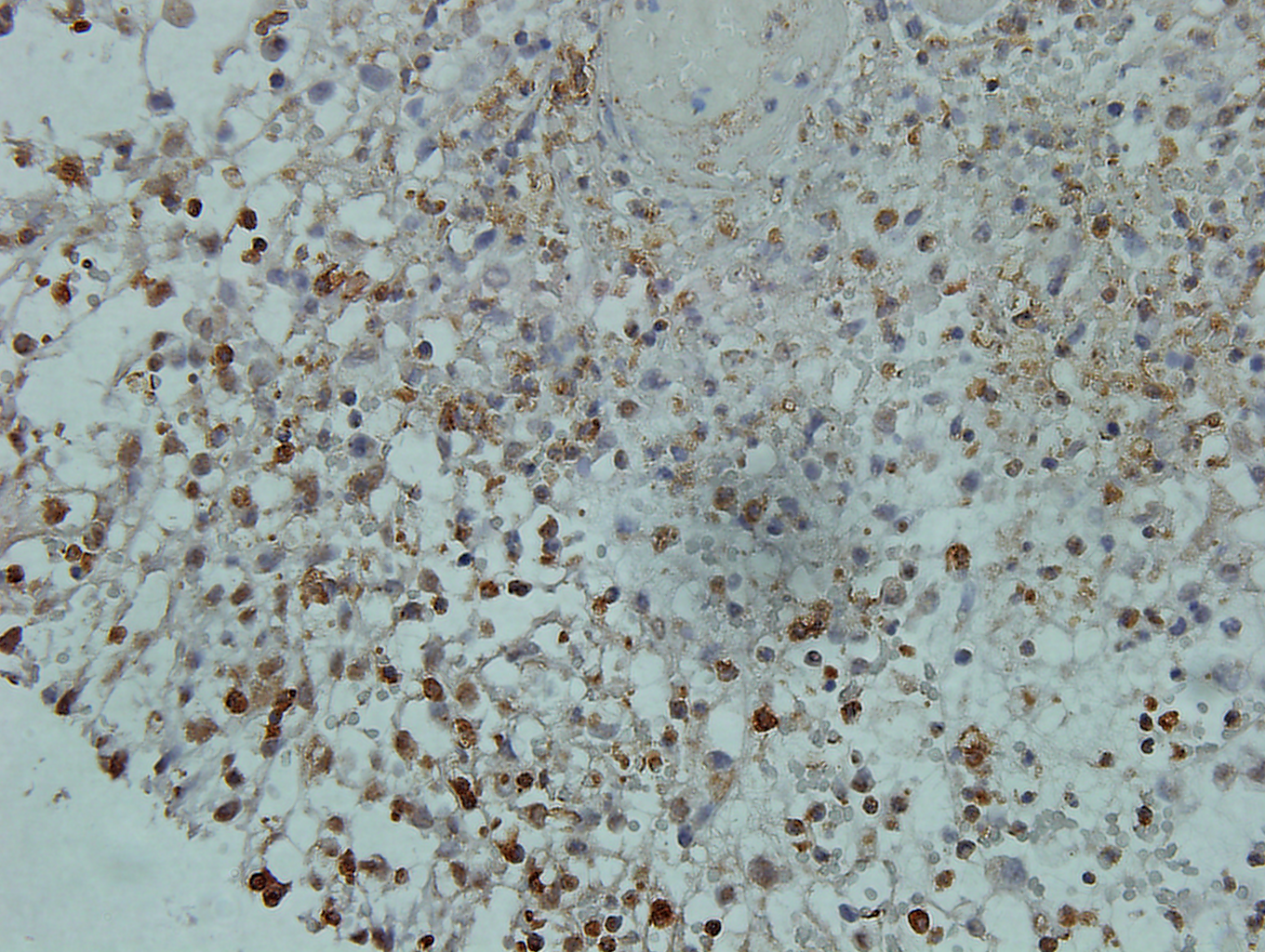

Supplement: Supplementary file 4 — Source Data Fig. 1 [file 44319_2023_33_MOESM4_ESM.zip › Fig.1/Fig. 1E/ERMS PATIENT TUMORS/E14_Fig.tif]

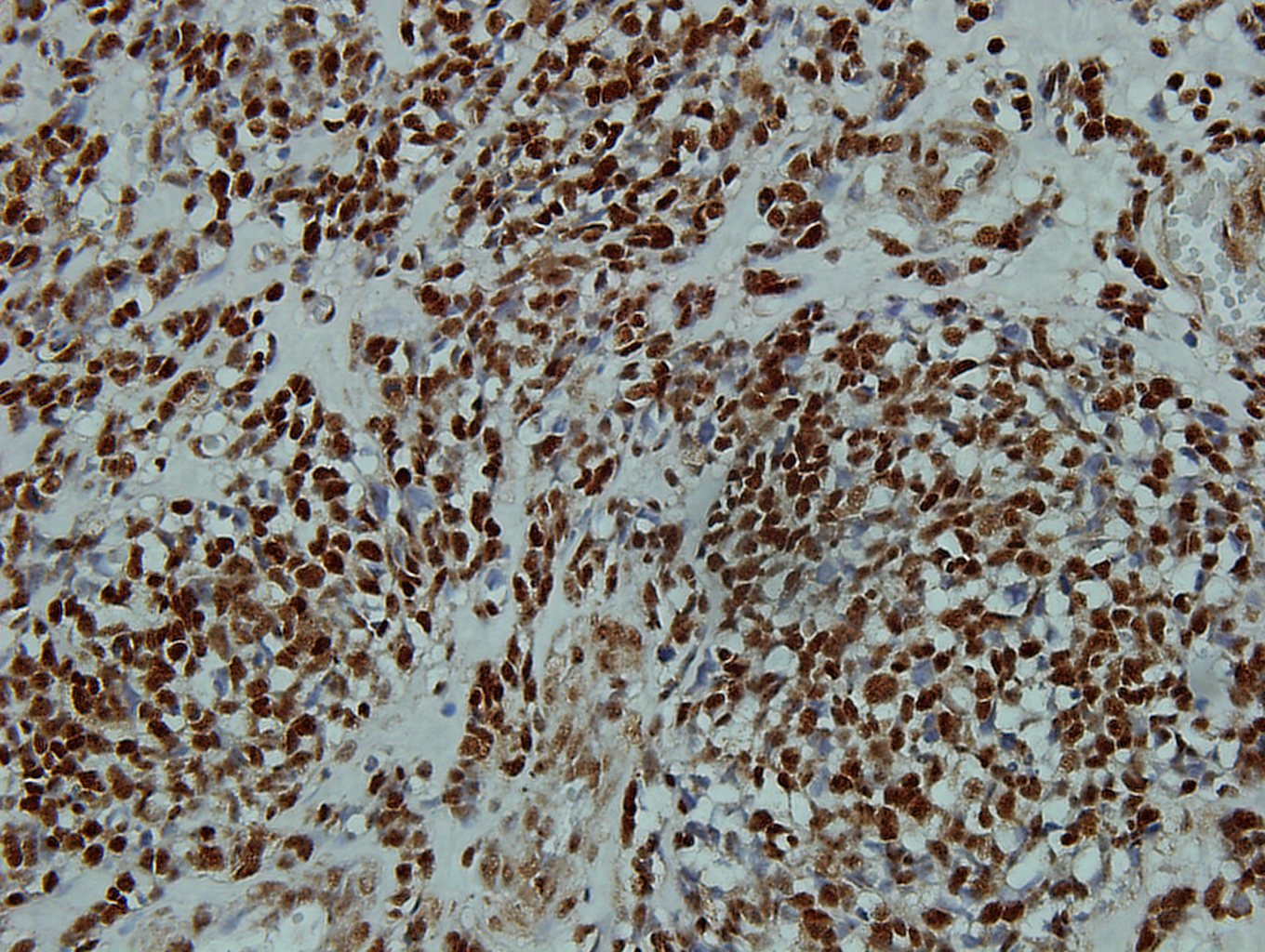

Supplement: Supplementary file 4 — Source Data Fig. 1 [file 44319_2023_33_MOESM4_ESM.zip › Fig.1/Fig. 1E/ERMS PATIENT TUMORS/E6_Fig.tif]

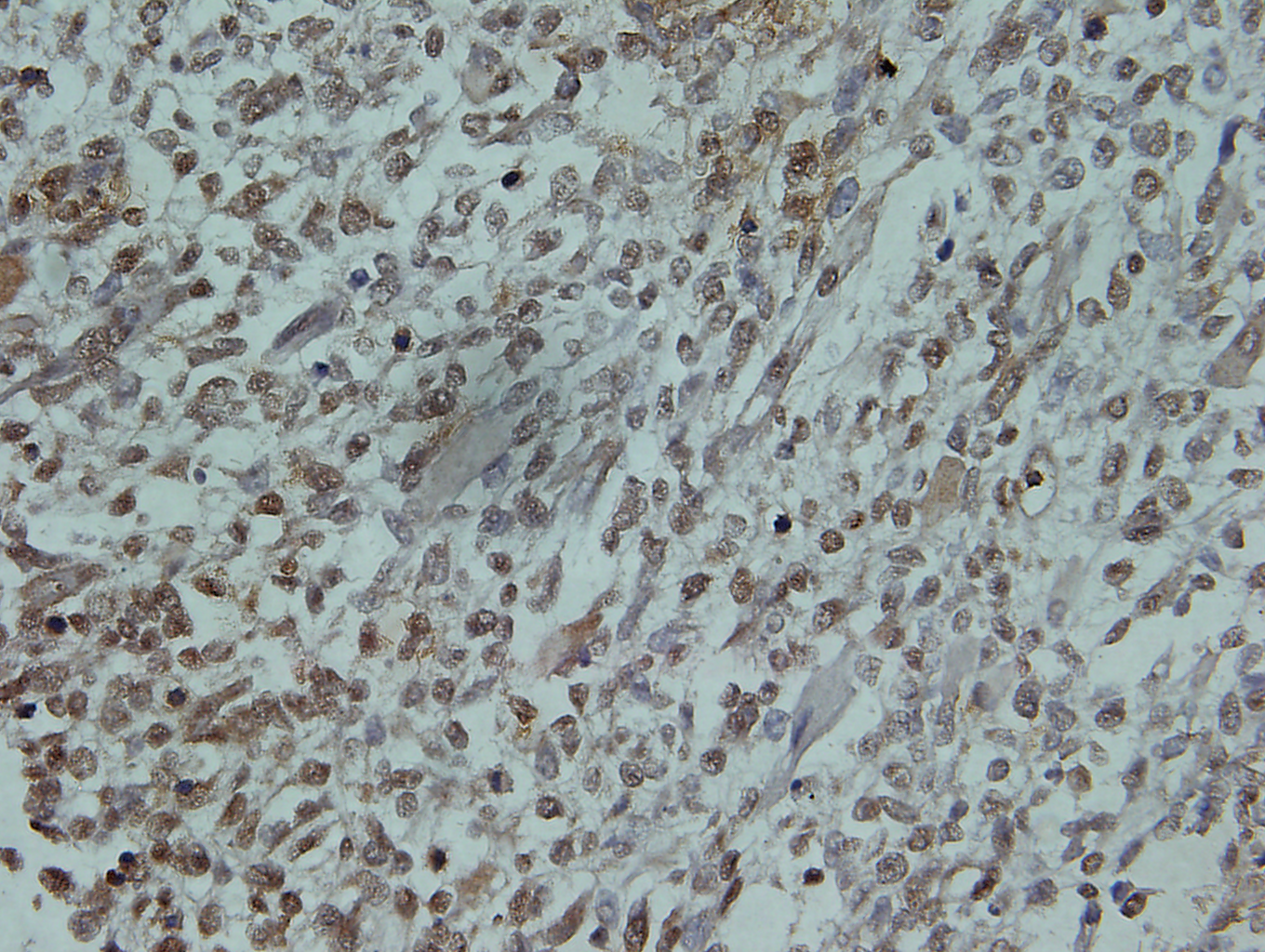

Supplement: Supplementary file 4 — Source Data Fig. 1 [file 44319_2023_33_MOESM4_ESM.zip › Fig.1/Fig. 1E/ERMS PATIENT TUMORS/E15_Fig.tif]

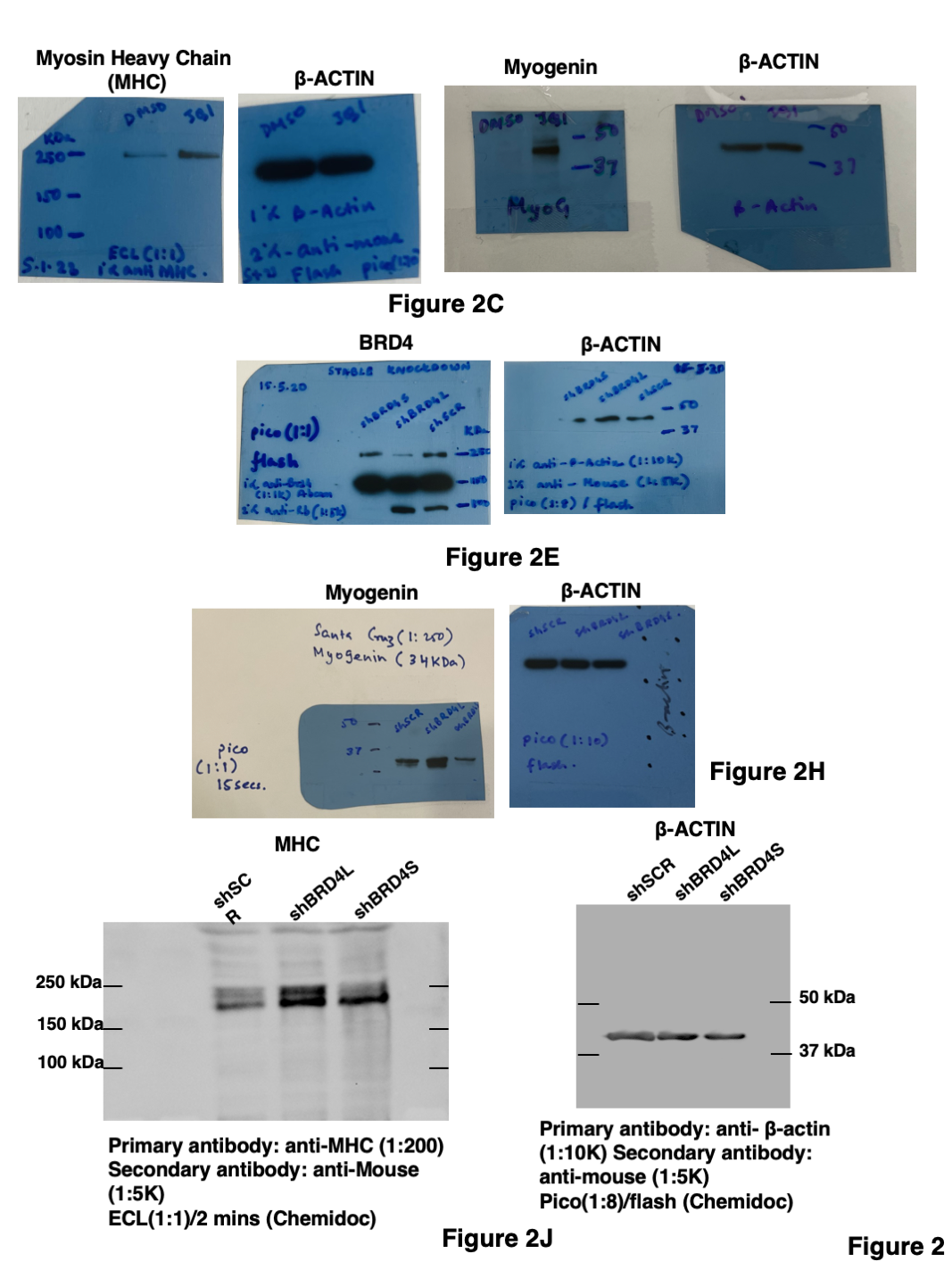

Supplement: Supplementary file 5 — Source Data Fig. 2 [file 44319_2023_33_MOESM5_ESM.zip › Fig.2/Fig. 2CEHJ_western.tiff]

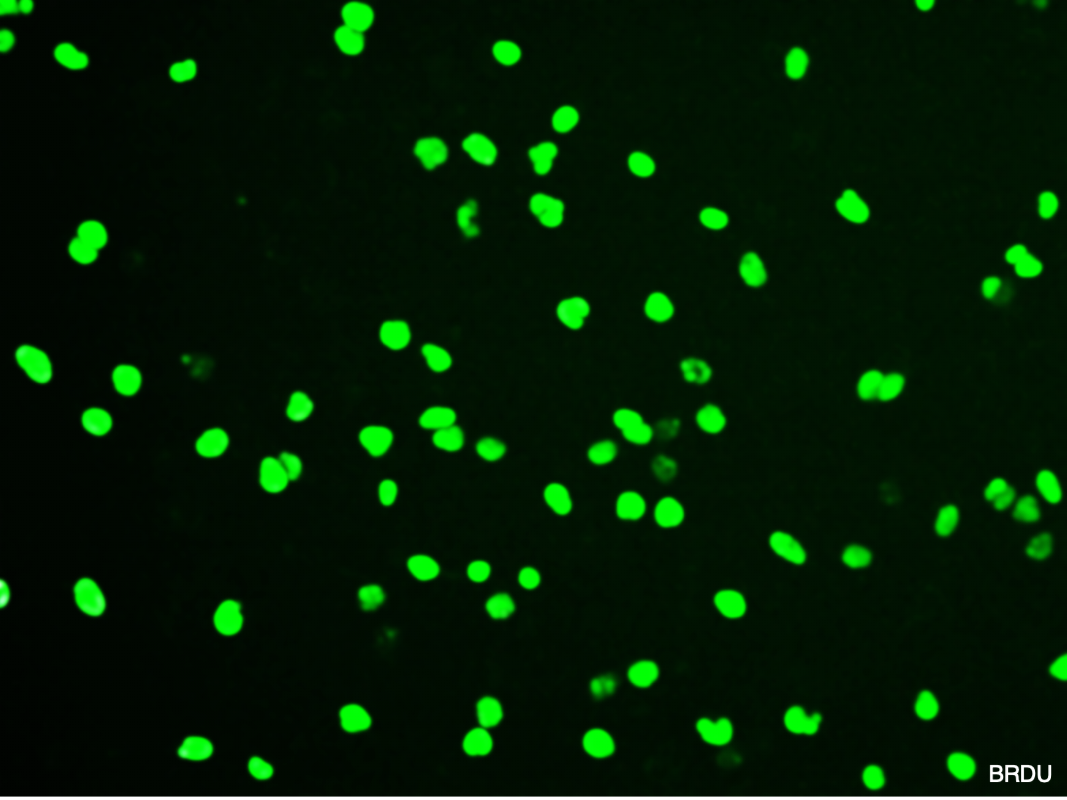

Supplement: Supplementary file 5 — Source Data Fig. 2 [file 44319_2023_33_MOESM5_ESM.zip › Fig.2/Fig. 2F/shScr/BRDU.tiff]

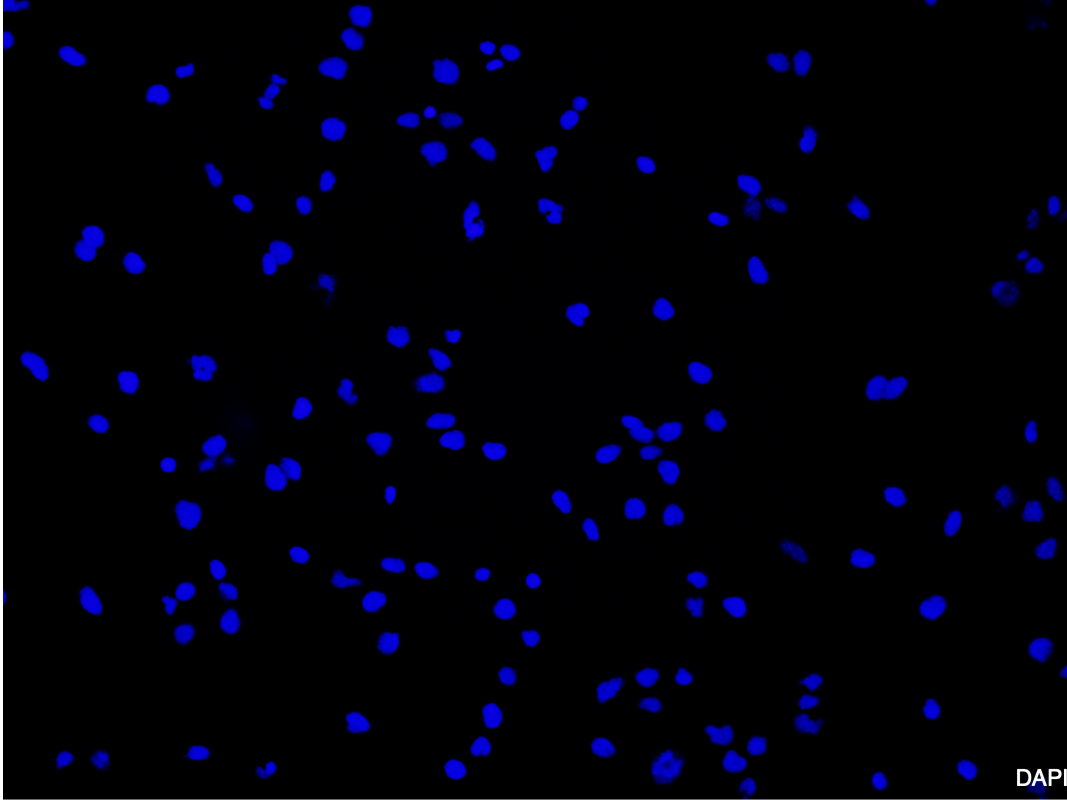

Supplement: Supplementary file 5 — Source Data Fig. 2 [file 44319_2023_33_MOESM5_ESM.zip › Fig.2/Fig. 2F/shScr/DAPI.tiff]

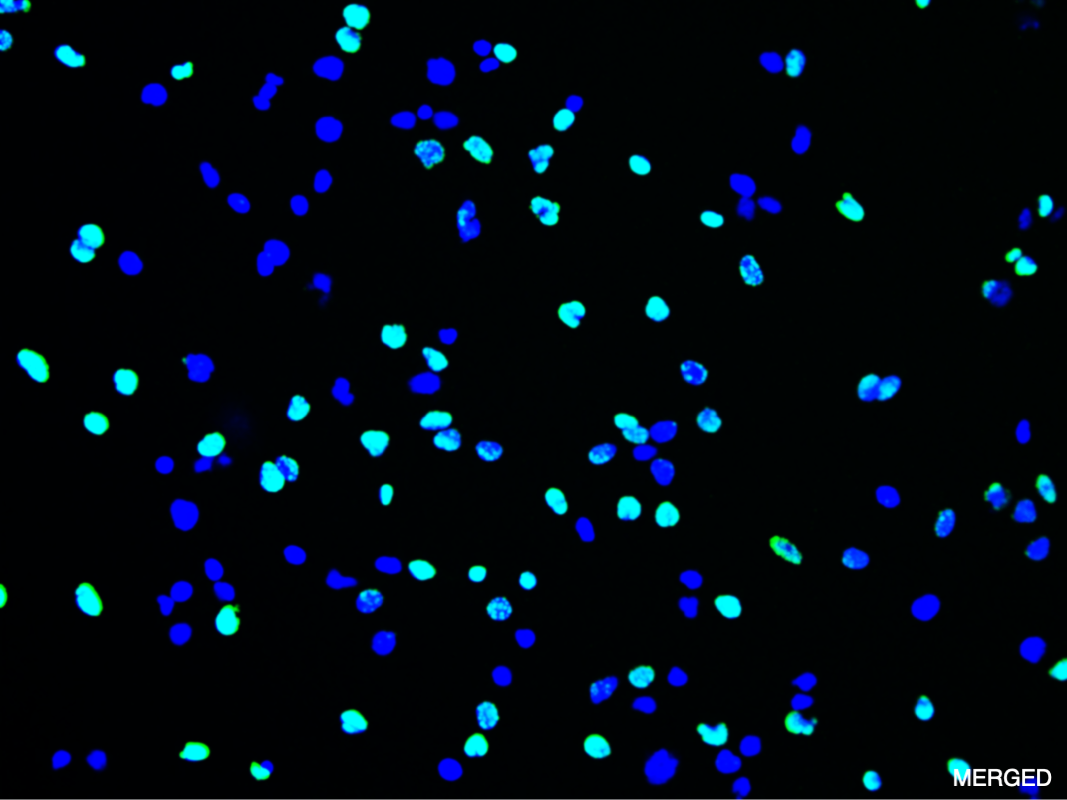

Supplement: Supplementary file 5 — Source Data Fig. 2 [file 44319_2023_33_MOESM5_ESM.zip › Fig.2/Fig. 2F/shScr/MERGED.tiff]

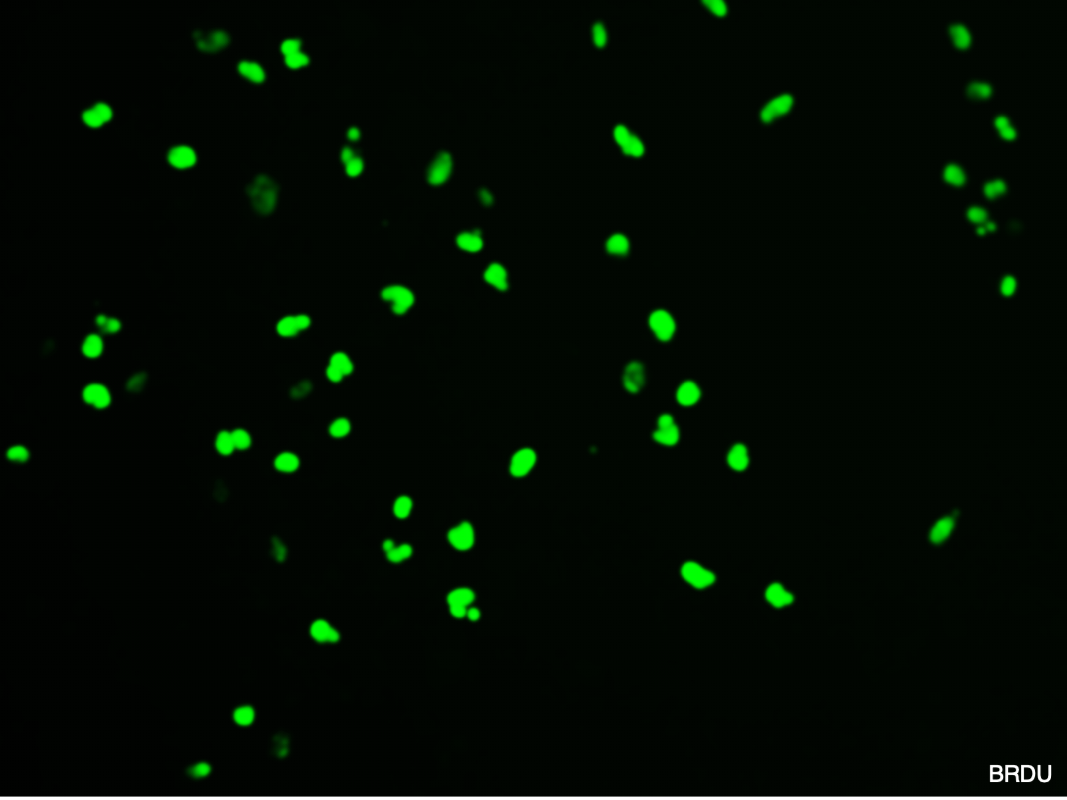

Supplement: Supplementary file 5 — Source Data Fig. 2 [file 44319_2023_33_MOESM5_ESM.zip › Fig.2/Fig. 2F/shBRD4-L/BRDU.tiff]

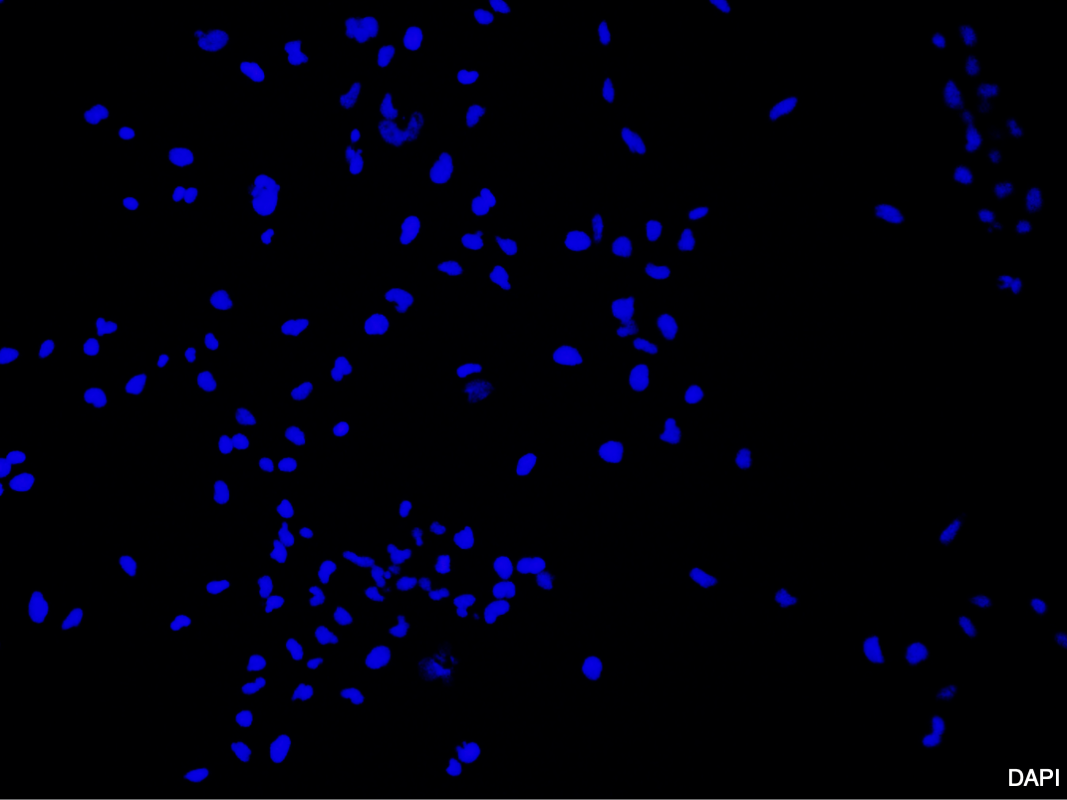

Supplement: Supplementary file 5 — Source Data Fig. 2 [file 44319_2023_33_MOESM5_ESM.zip › Fig.2/Fig. 2F/shBRD4-L/DAPI.tiff]

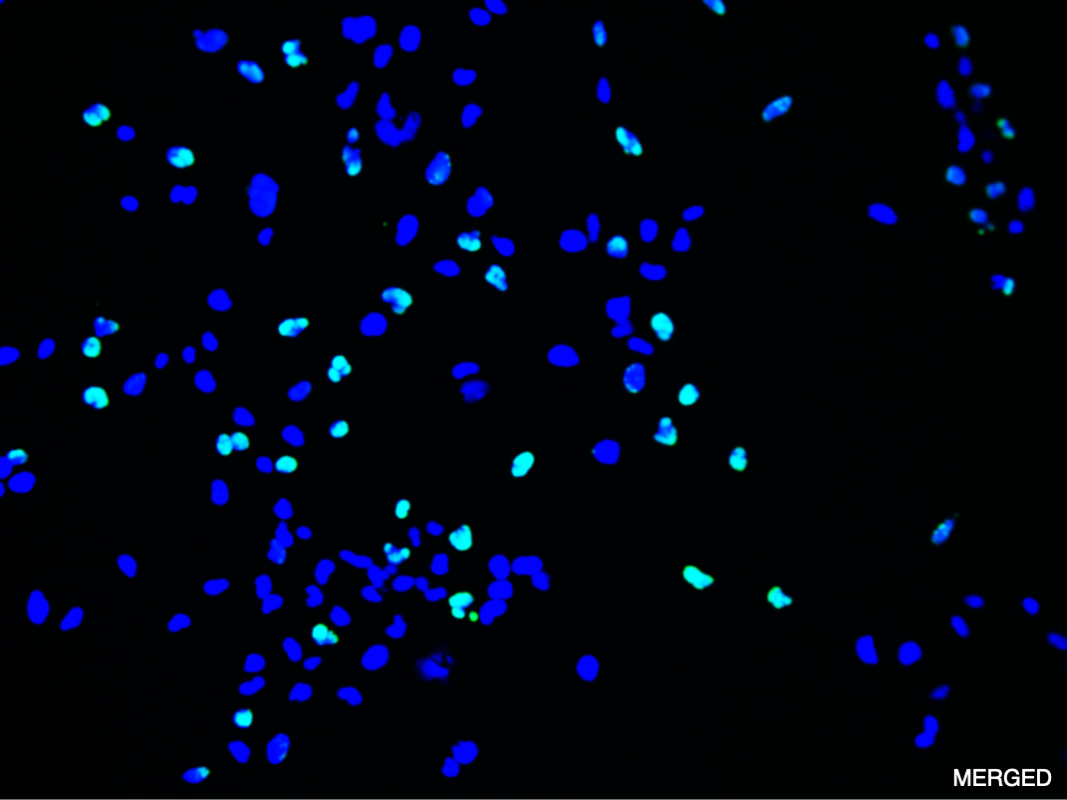

Supplement: Supplementary file 5 — Source Data Fig. 2 [file 44319_2023_33_MOESM5_ESM.zip › Fig.2/Fig. 2F/shBRD4-L/MERGED.tiff]

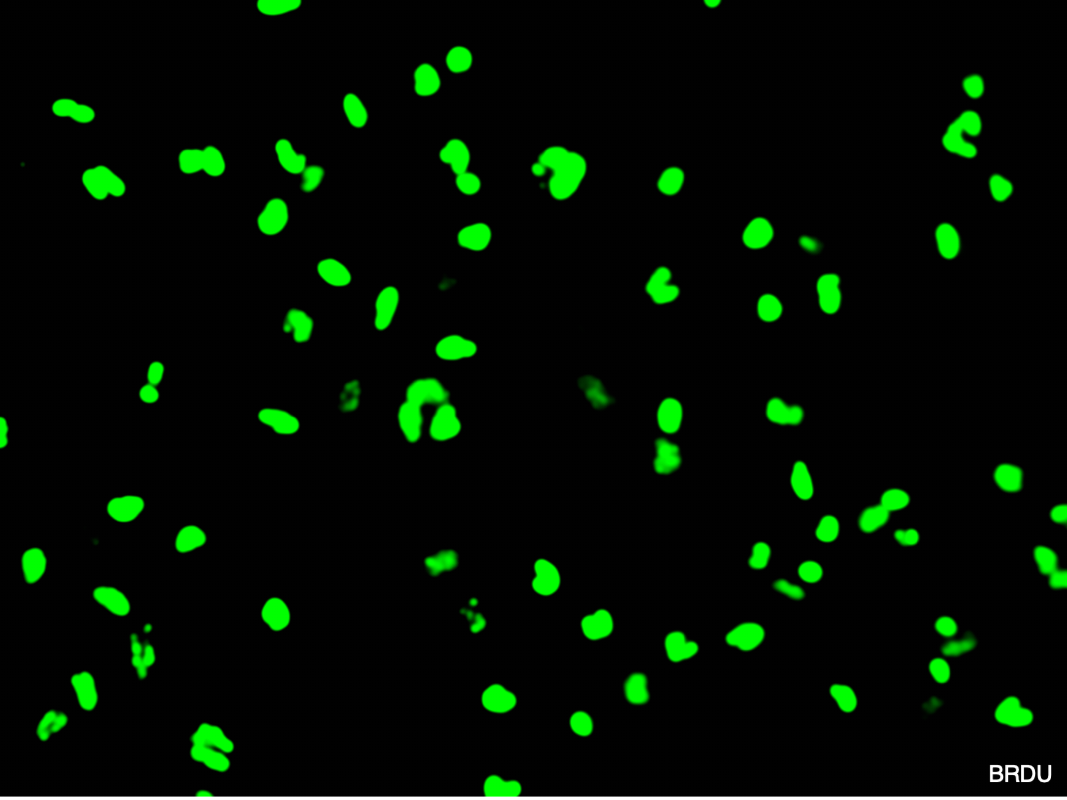

Supplement: Supplementary file 5 — Source Data Fig. 2 [file 44319_2023_33_MOESM5_ESM.zip › Fig.2/Fig. 2F/shBRD4-S/BRDU.tiff]

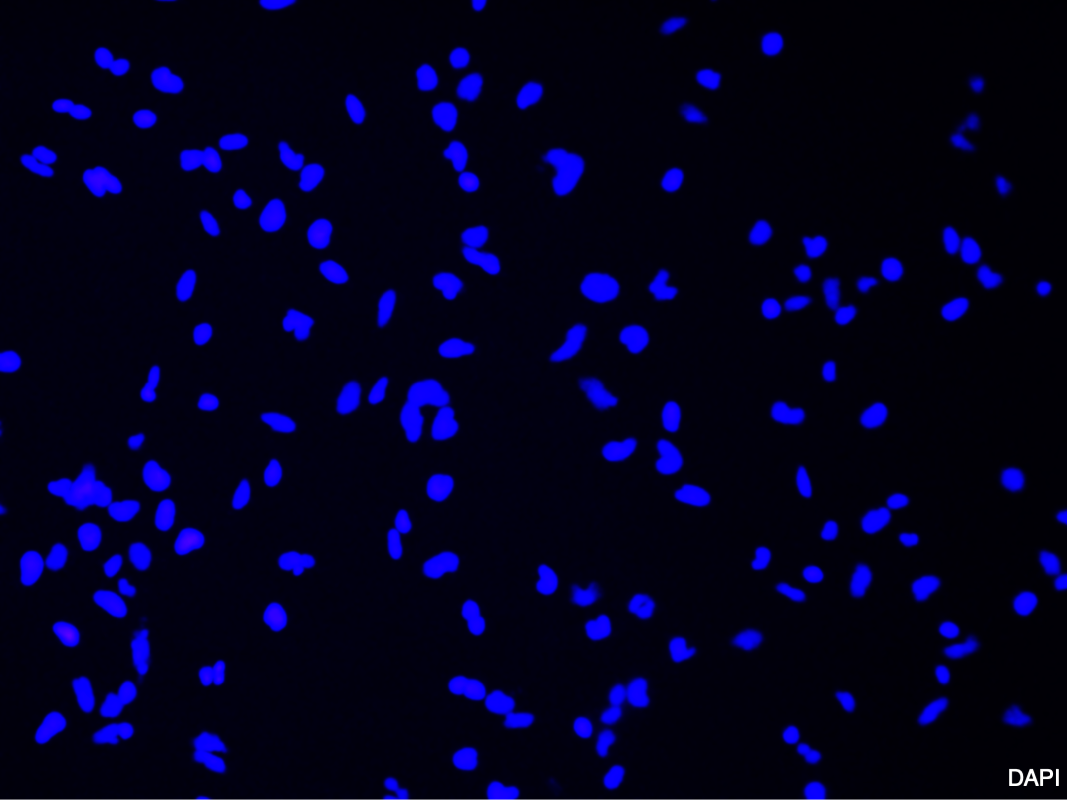

Supplement: Supplementary file 5 — Source Data Fig. 2 [file 44319_2023_33_MOESM5_ESM.zip › Fig.2/Fig. 2F/shBRD4-S/DAPI.tiff]

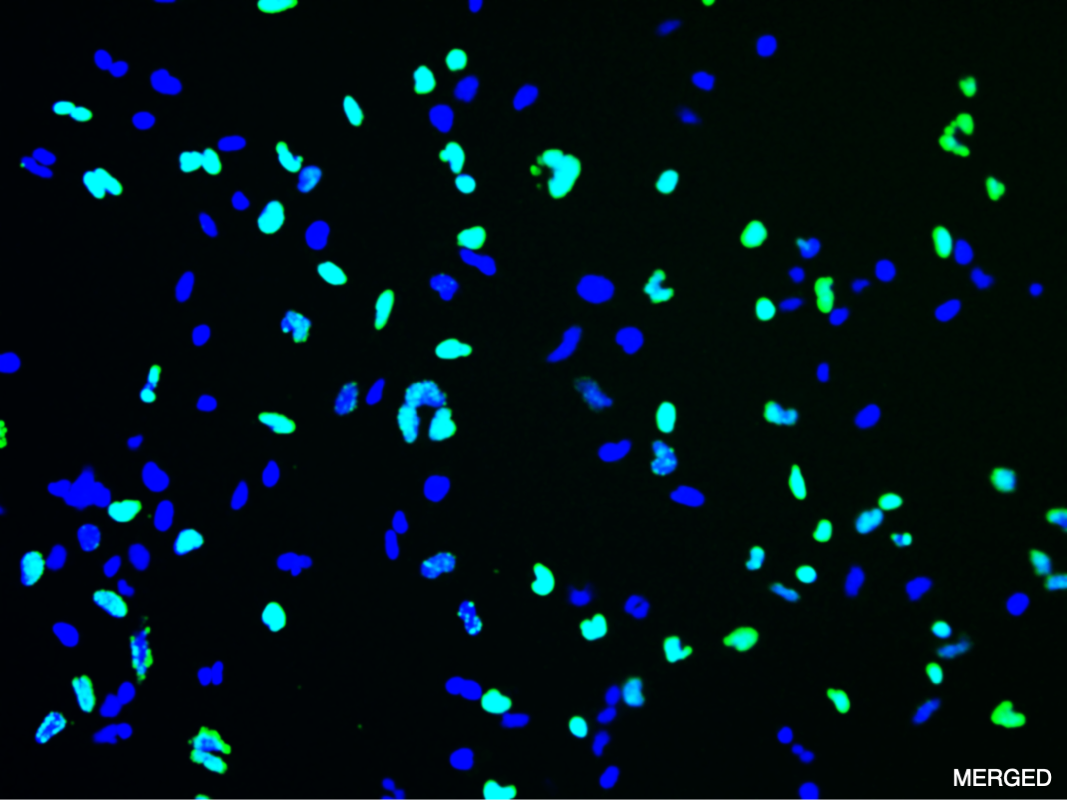

Supplement: Supplementary file 5 — Source Data Fig. 2 [file 44319_2023_33_MOESM5_ESM.zip › Fig.2/Fig. 2F/shBRD4-S/MERGED.tiff]

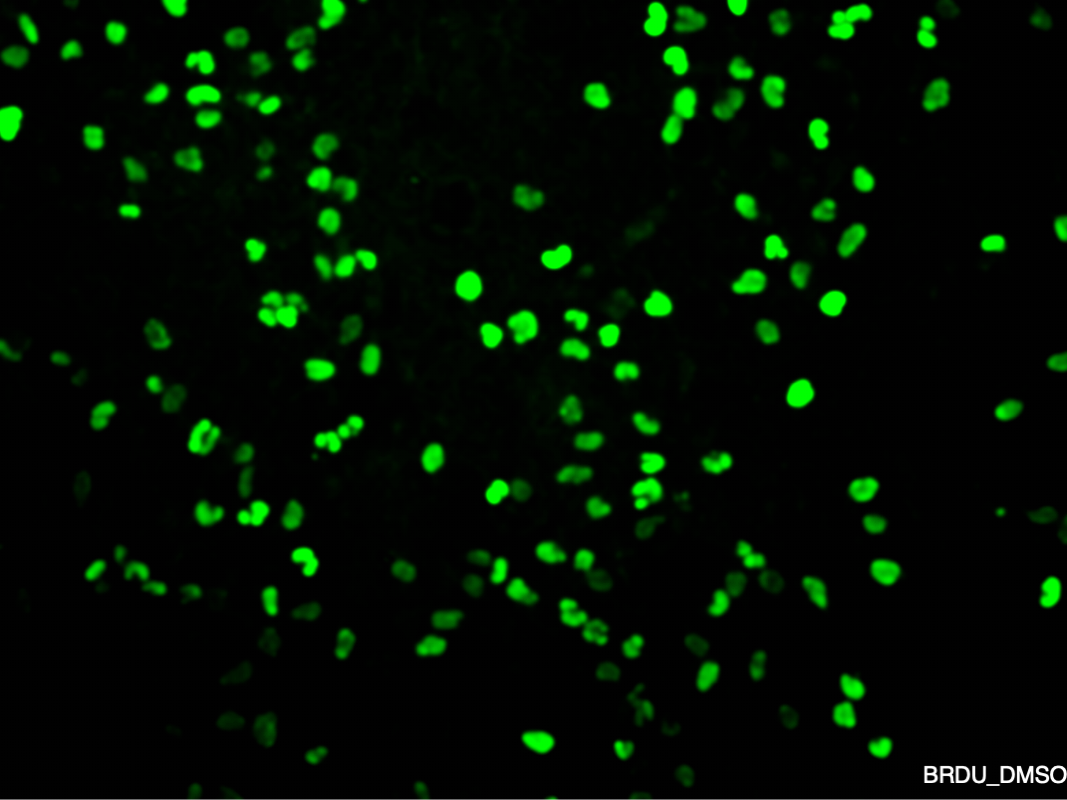

Supplement: Supplementary file 5 — Source Data Fig. 2 [file 44319_2023_33_MOESM5_ESM.zip › Fig.2/Fig. 2A/DMSO/BRDU.tiff]

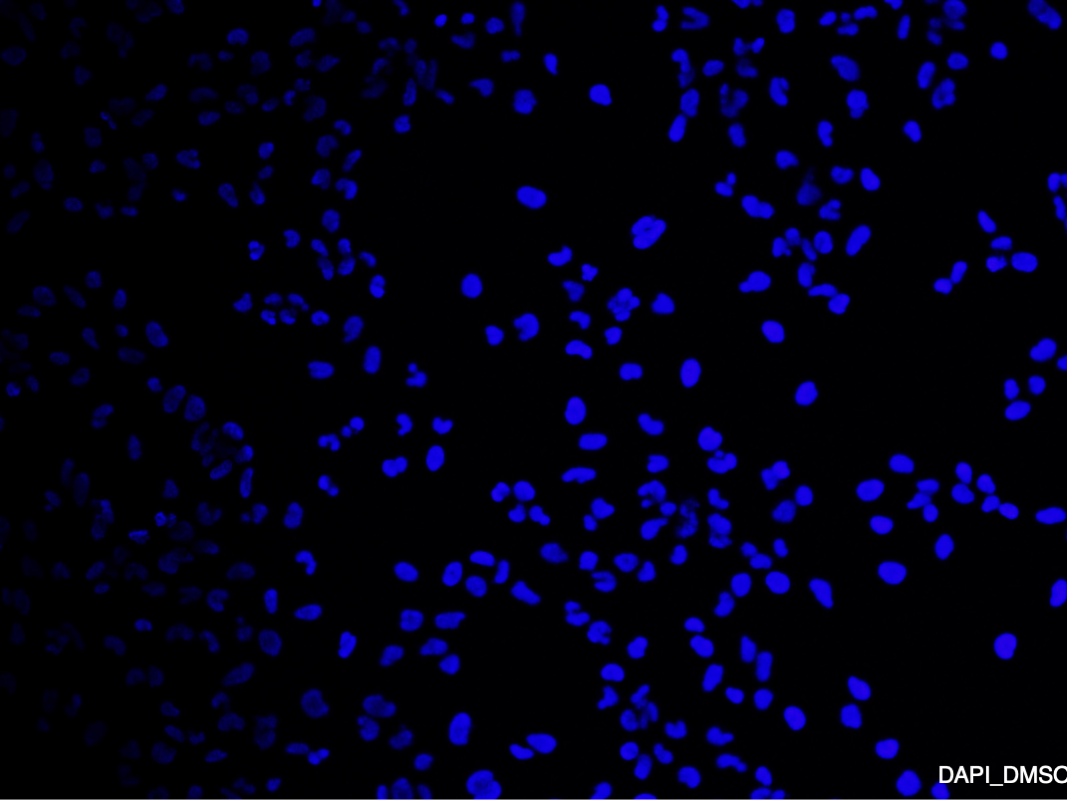

Supplement: Supplementary file 5 — Source Data Fig. 2 [file 44319_2023_33_MOESM5_ESM.zip › Fig.2/Fig. 2A/DMSO/DAPI.tiff]

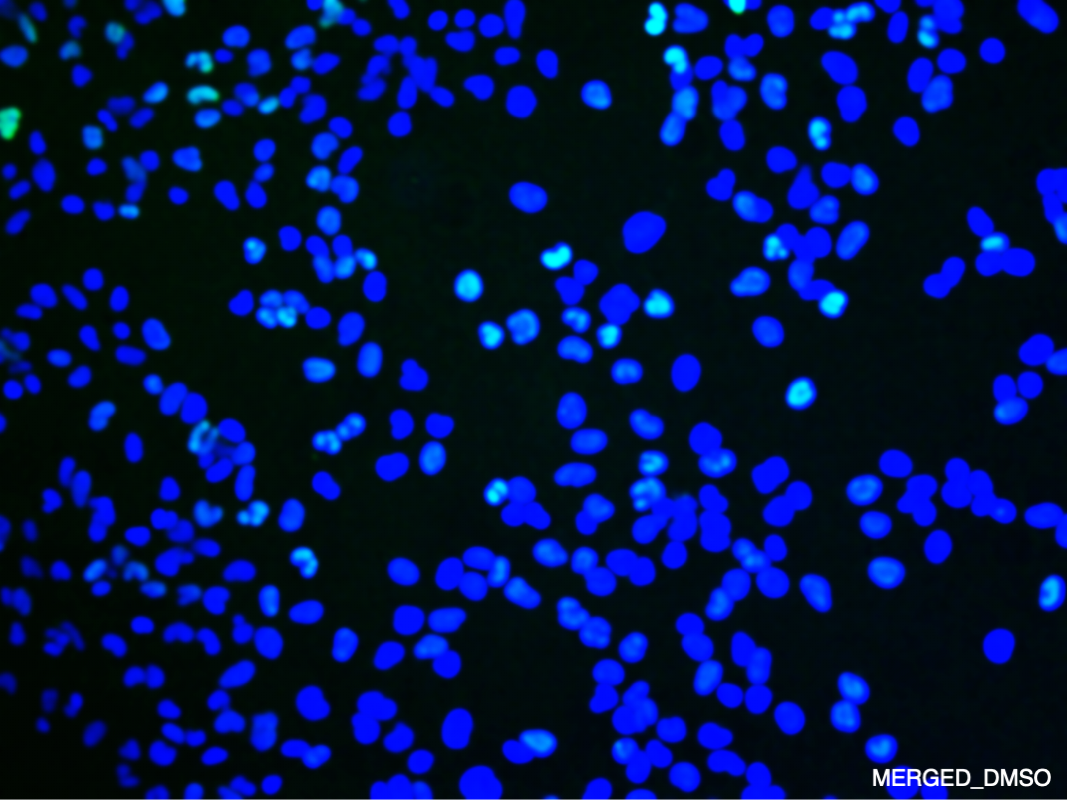

Supplement: Supplementary file 5 — Source Data Fig. 2 [file 44319_2023_33_MOESM5_ESM.zip › Fig.2/Fig. 2A/DMSO/MERGED.tiff]

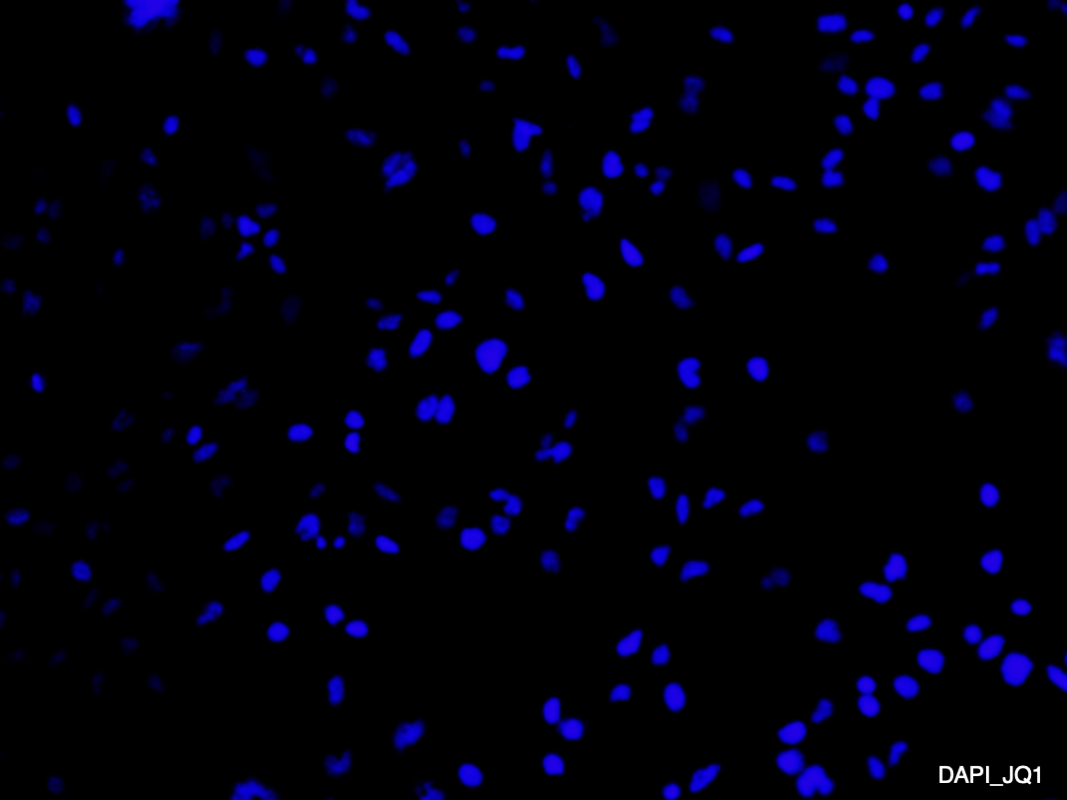

Supplement: Supplementary file 5 — Source Data Fig. 2 [file 44319_2023_33_MOESM5_ESM.zip › Fig.2/Fig. 2A/JQ1/DAPI_JQ1.tiff]

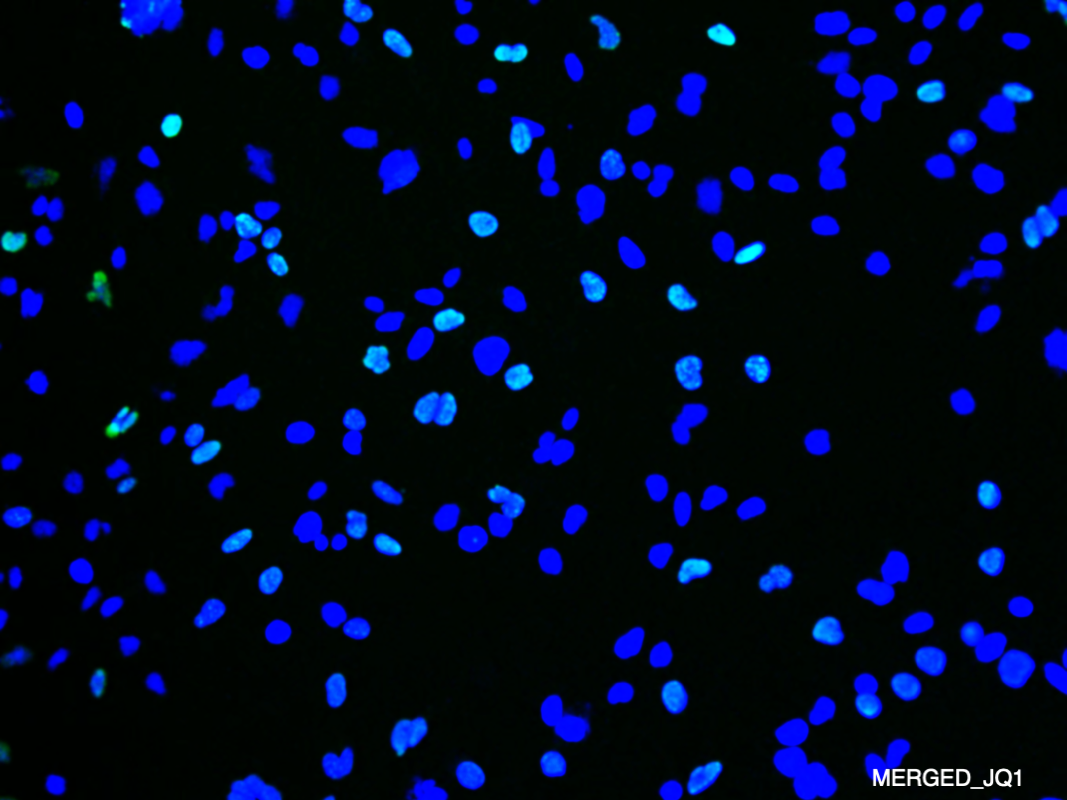

Supplement: Supplementary file 5 — Source Data Fig. 2 [file 44319_2023_33_MOESM5_ESM.zip › Fig.2/Fig. 2A/JQ1/MERGED_JQ1.tiff]

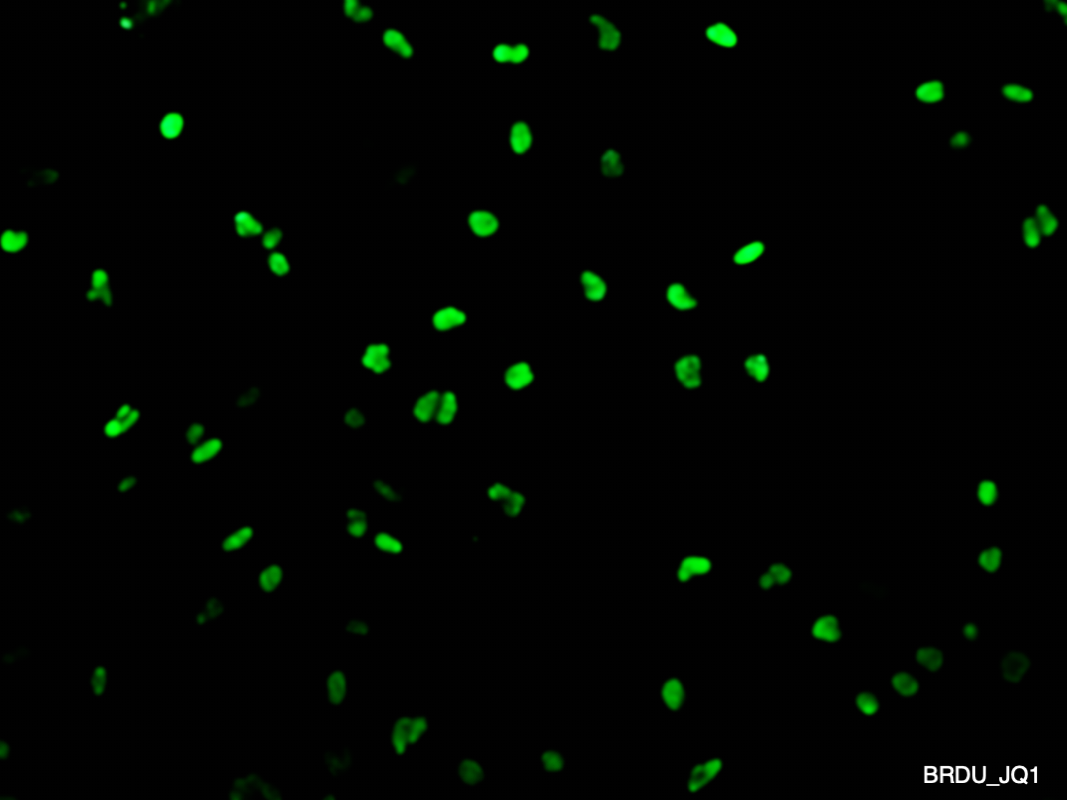

Supplement: Supplementary file 5 — Source Data Fig. 2 [file 44319_2023_33_MOESM5_ESM.zip › Fig.2/Fig. 2A/JQ1/BRDU_JQ1.tiff]

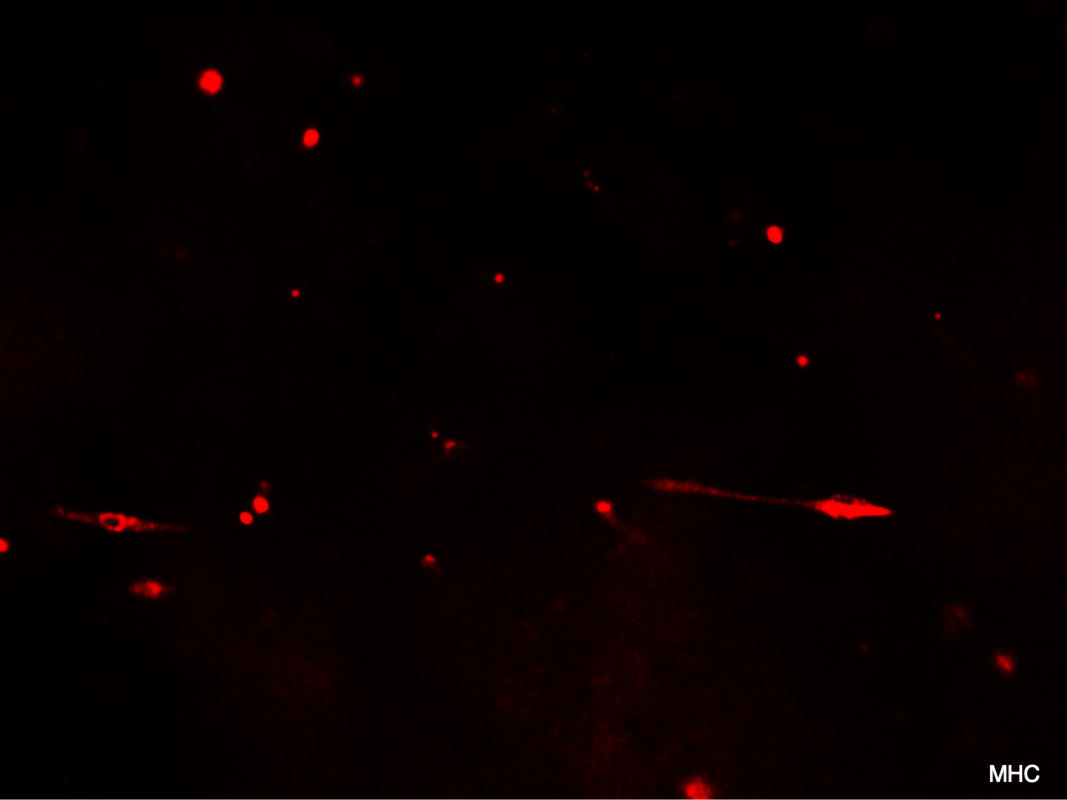

Supplement: Supplementary file 5 — Source Data Fig. 2 [file 44319_2023_33_MOESM5_ESM.zip › Fig.2/Fig. 2I/shScr/MHC.tiff]

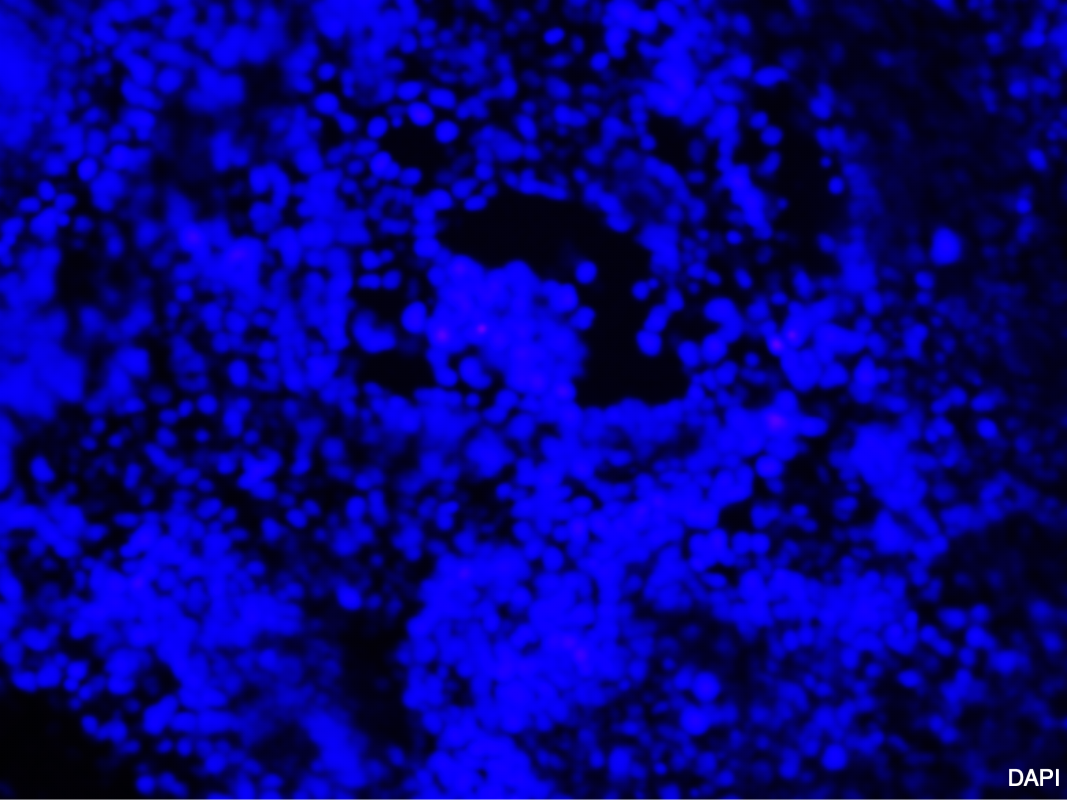

Supplement: Supplementary file 5 — Source Data Fig. 2 [file 44319_2023_33_MOESM5_ESM.zip › Fig.2/Fig. 2I/shScr/DAPI.tiff]

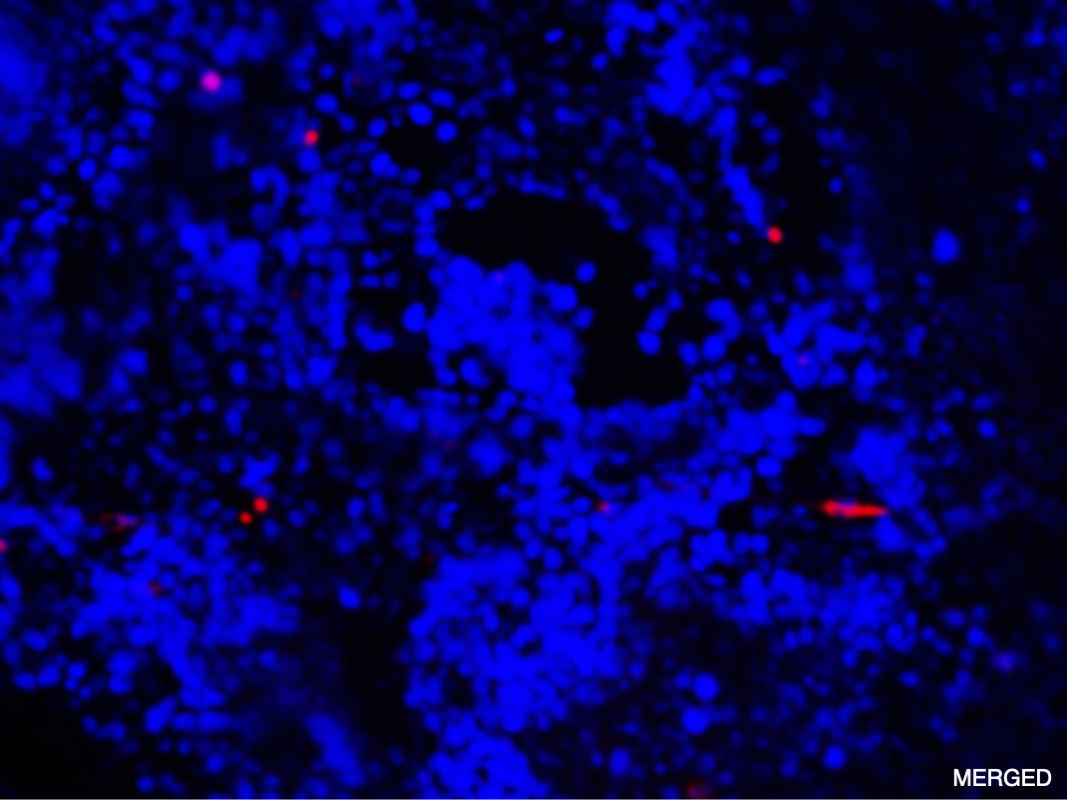

Supplement: Supplementary file 5 — Source Data Fig. 2 [file 44319_2023_33_MOESM5_ESM.zip › Fig.2/Fig. 2I/shScr/MERGED.tiff]

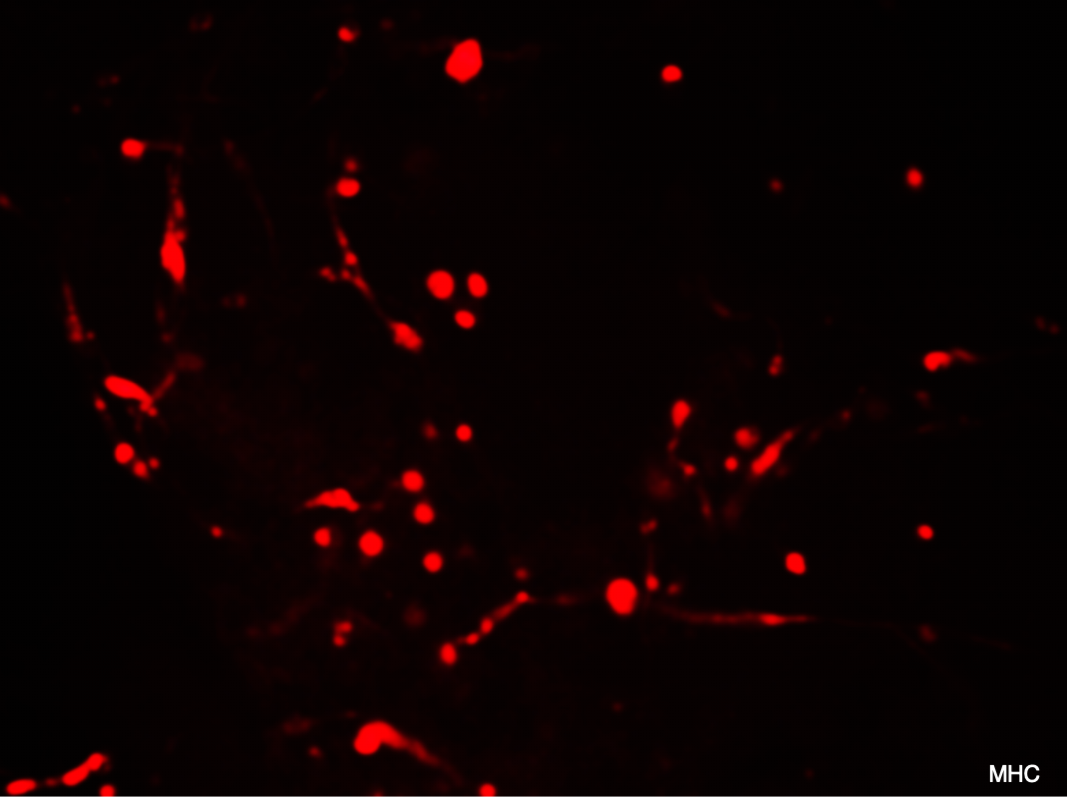

Supplement: Supplementary file 5 — Source Data Fig. 2 [file 44319_2023_33_MOESM5_ESM.zip › Fig.2/Fig. 2I/shBRD4-L/MHC.tiff]

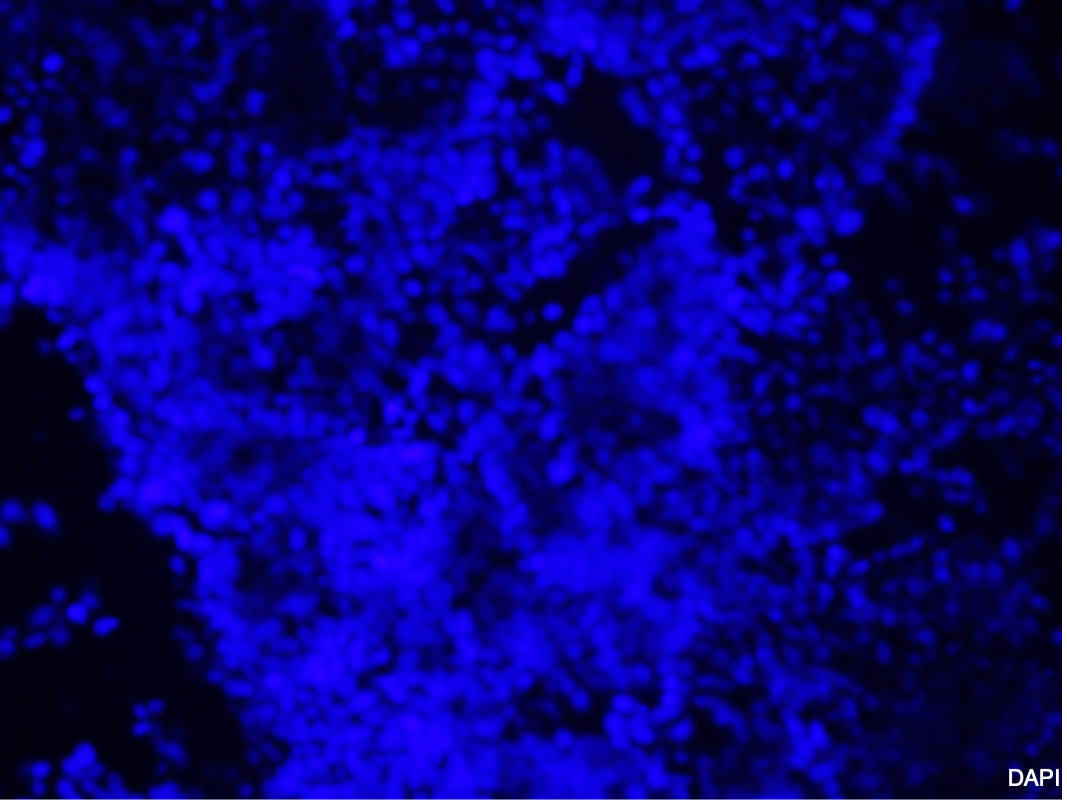

Supplement: Supplementary file 5 — Source Data Fig. 2 [file 44319_2023_33_MOESM5_ESM.zip › Fig.2/Fig. 2I/shBRD4-L/DAPI.tiff]

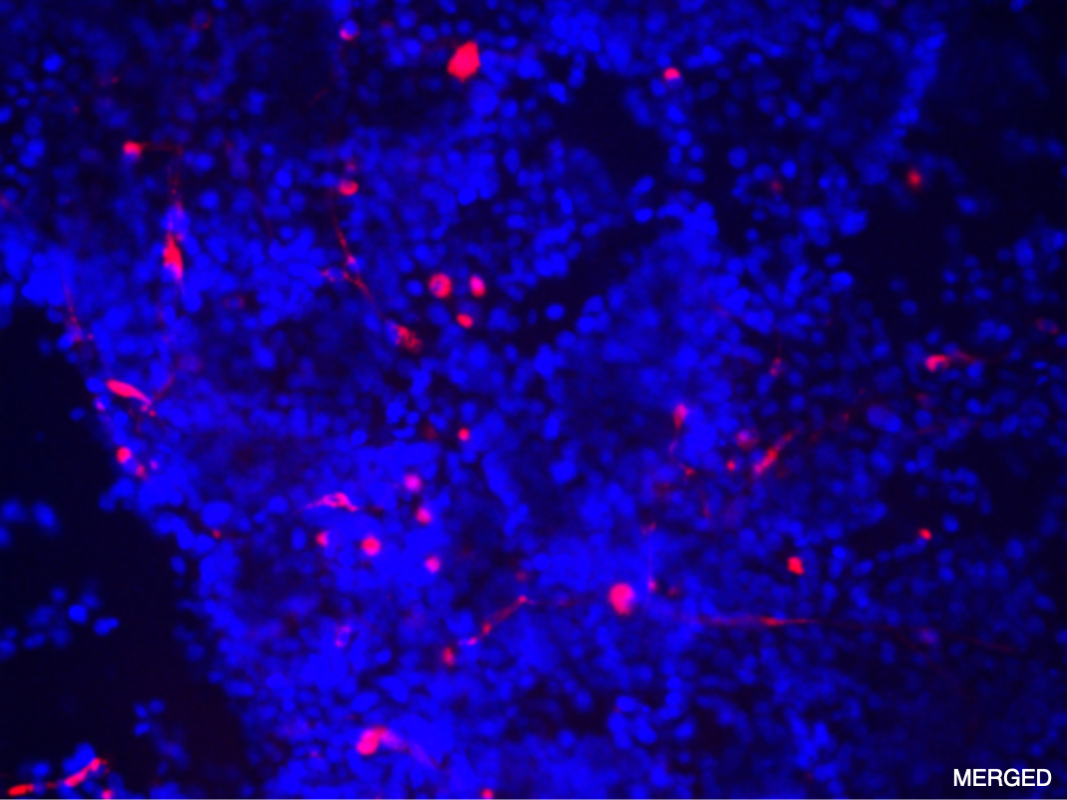

Supplement: Supplementary file 5 — Source Data Fig. 2 [file 44319_2023_33_MOESM5_ESM.zip › Fig.2/Fig. 2I/shBRD4-L/MERGED.tiff]

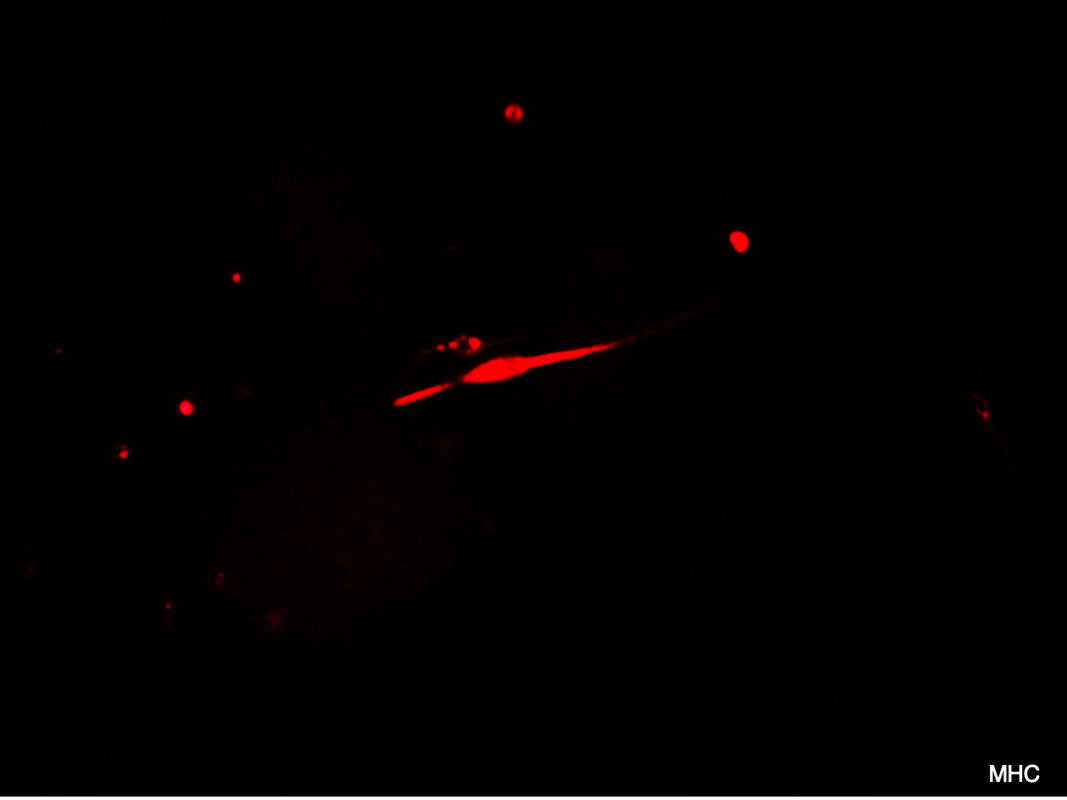

Supplement: Supplementary file 5 — Source Data Fig. 2 [file 44319_2023_33_MOESM5_ESM.zip › Fig.2/Fig. 2I/shBRD4-S/MHC.tiff]

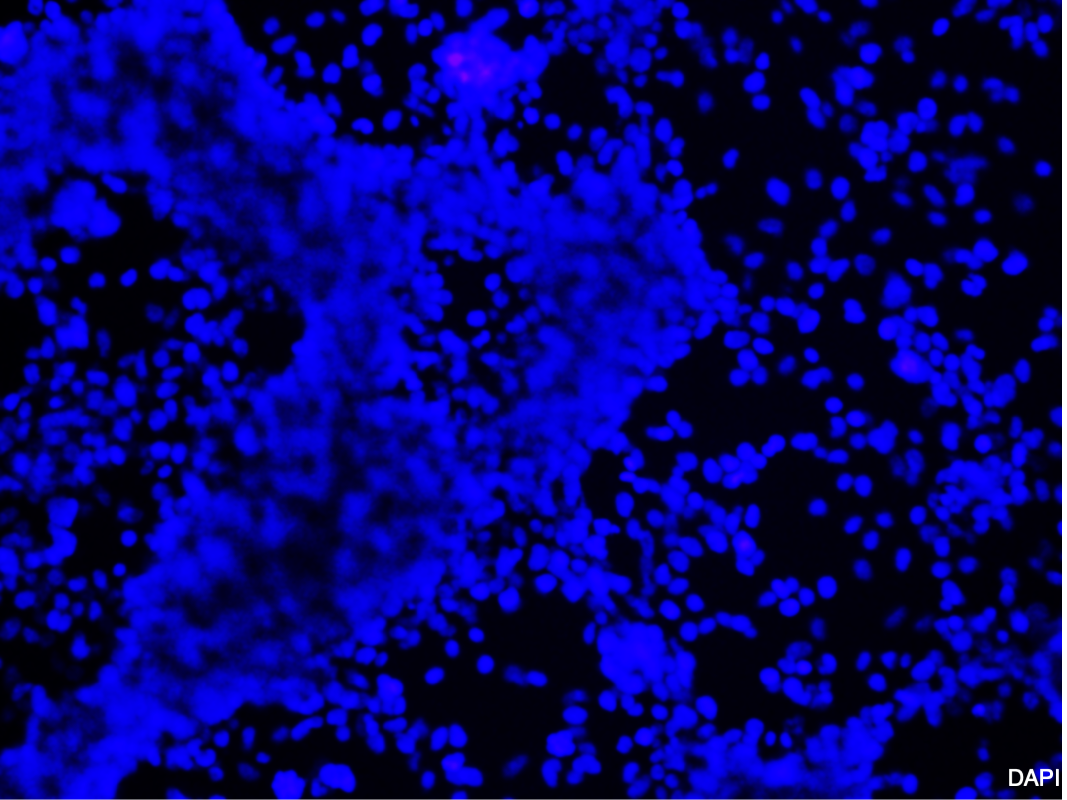

Supplement: Supplementary file 5 — Source Data Fig. 2 [file 44319_2023_33_MOESM5_ESM.zip › Fig.2/Fig. 2I/shBRD4-S/DAPI.tiff]

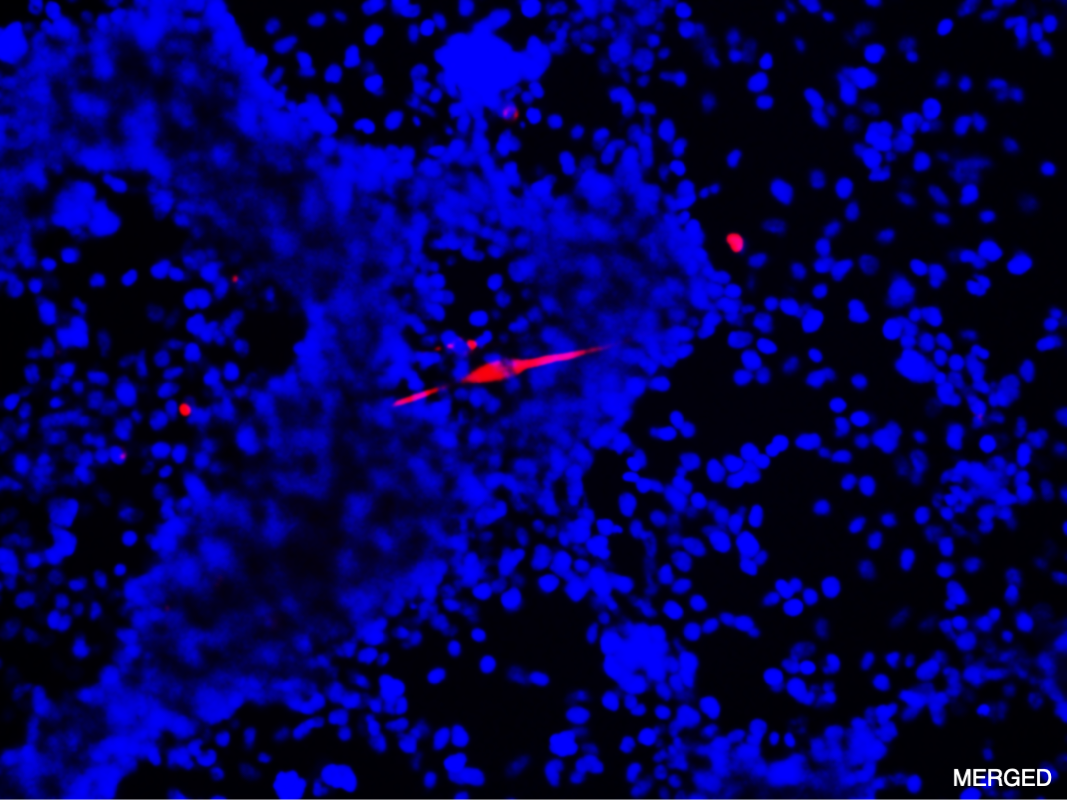

Supplement: Supplementary file 5 — Source Data Fig. 2 [file 44319_2023_33_MOESM5_ESM.zip › Fig.2/Fig. 2I/shBRD4-S/MERGED.tiff]

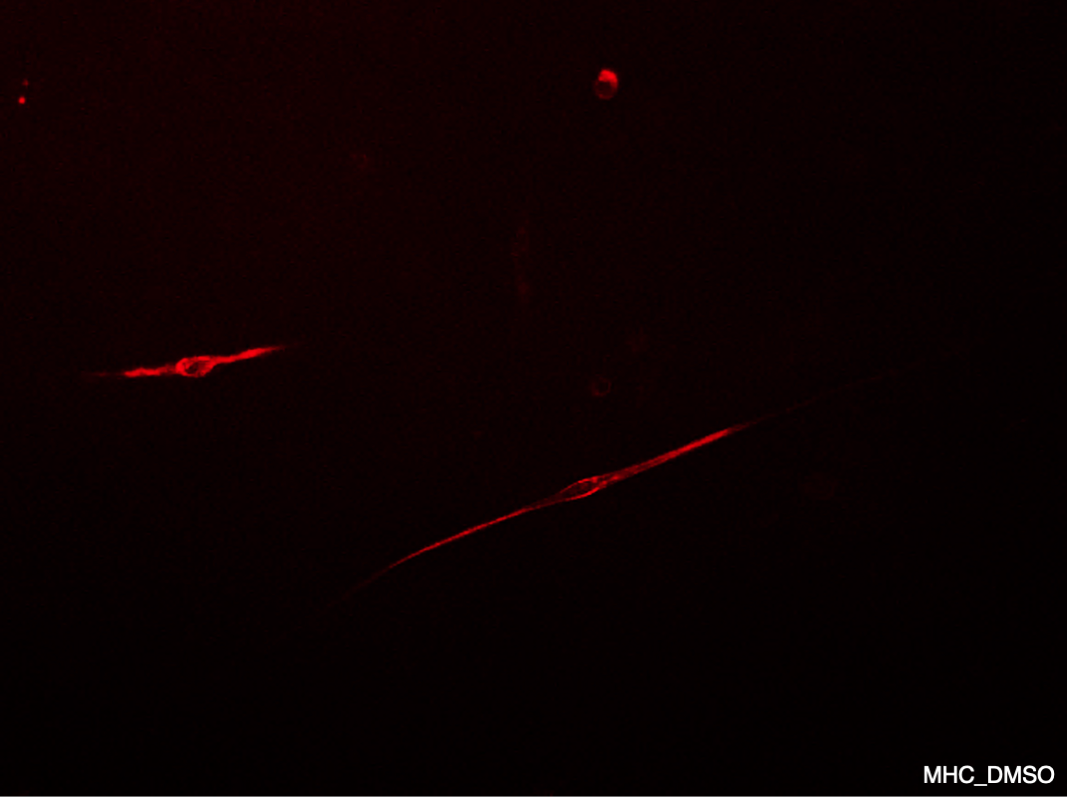

Supplement: Supplementary file 5 — Source Data Fig. 2 [file 44319_2023_33_MOESM5_ESM.zip › Fig.2/Fig. 2B/DMSO/MHC.tiff]

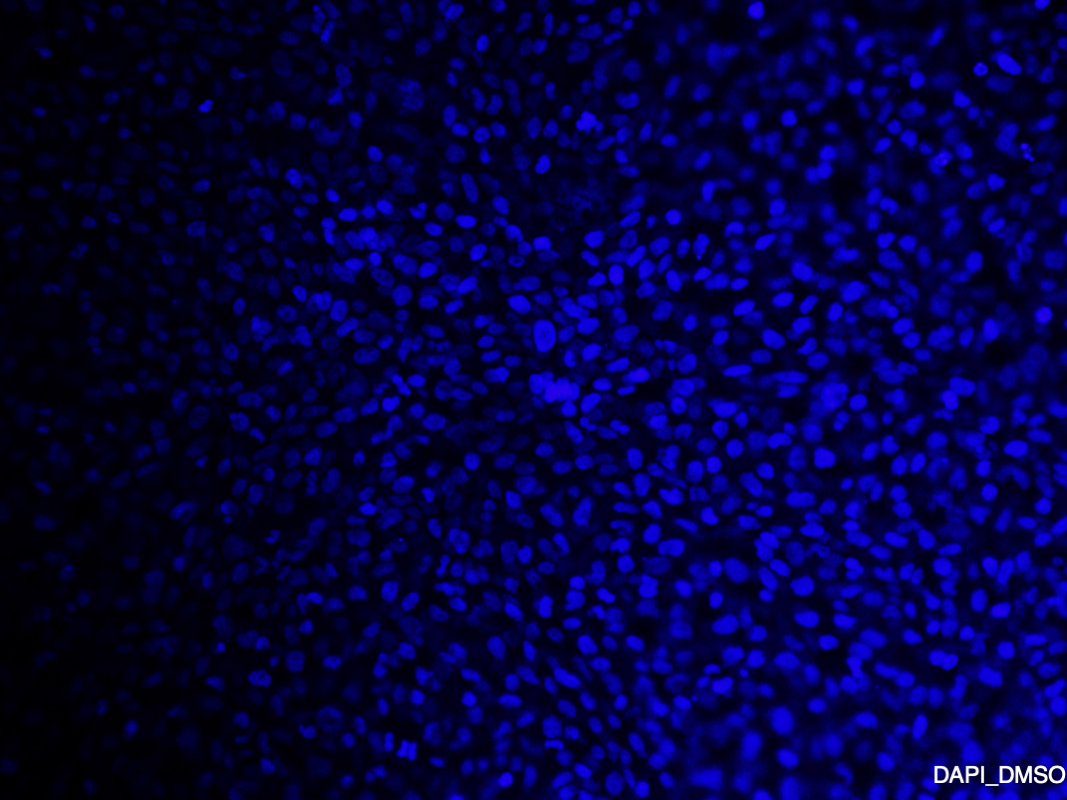

Supplement: Supplementary file 5 — Source Data Fig. 2 [file 44319_2023_33_MOESM5_ESM.zip › Fig.2/Fig. 2B/DMSO/DAPI.tiff]

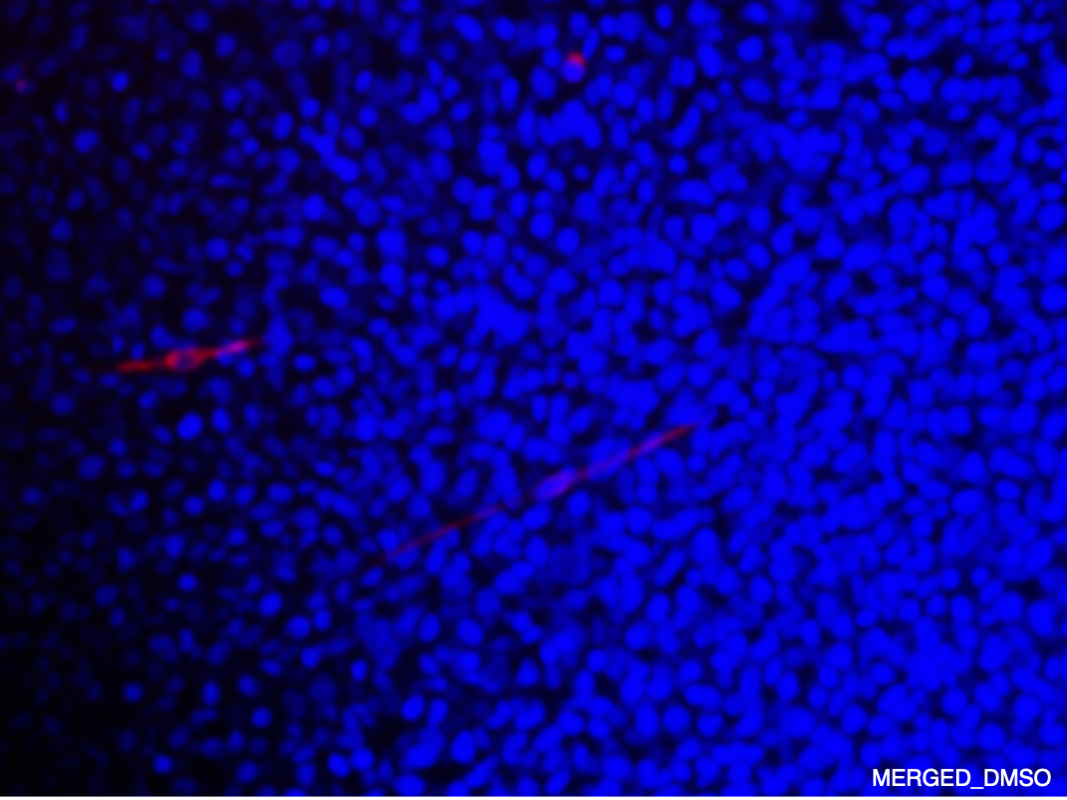

Supplement: Supplementary file 5 — Source Data Fig. 2 [file 44319_2023_33_MOESM5_ESM.zip › Fig.2/Fig. 2B/DMSO/MERGED.tiff]

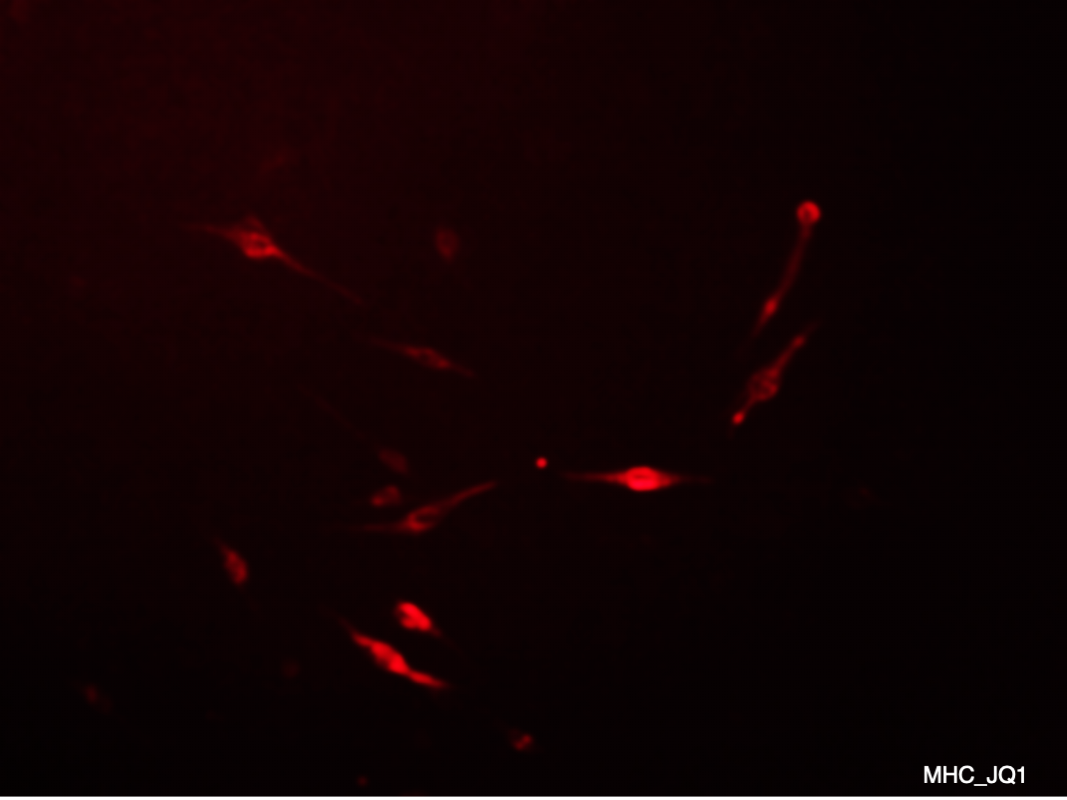

Supplement: Supplementary file 5 — Source Data Fig. 2 [file 44319_2023_33_MOESM5_ESM.zip › Fig.2/Fig. 2B/JQ1/MHC.tiff]

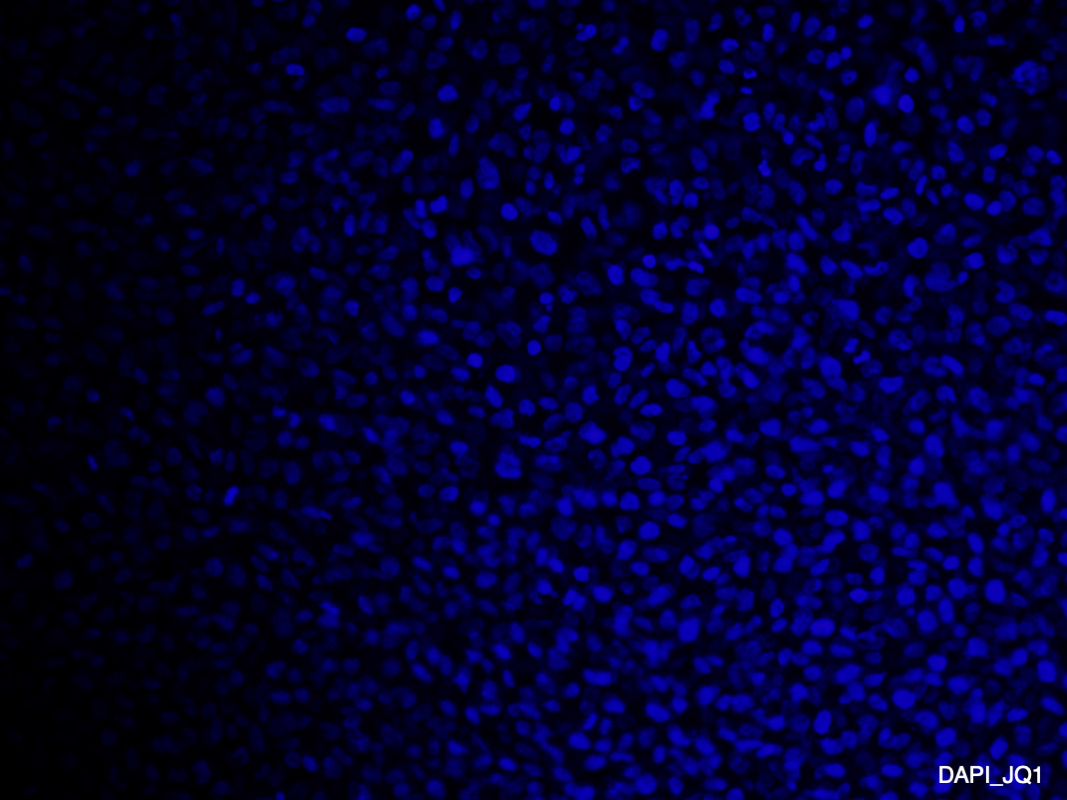

Supplement: Supplementary file 5 — Source Data Fig. 2 [file 44319_2023_33_MOESM5_ESM.zip › Fig.2/Fig. 2B/JQ1/DAPI.tiff]

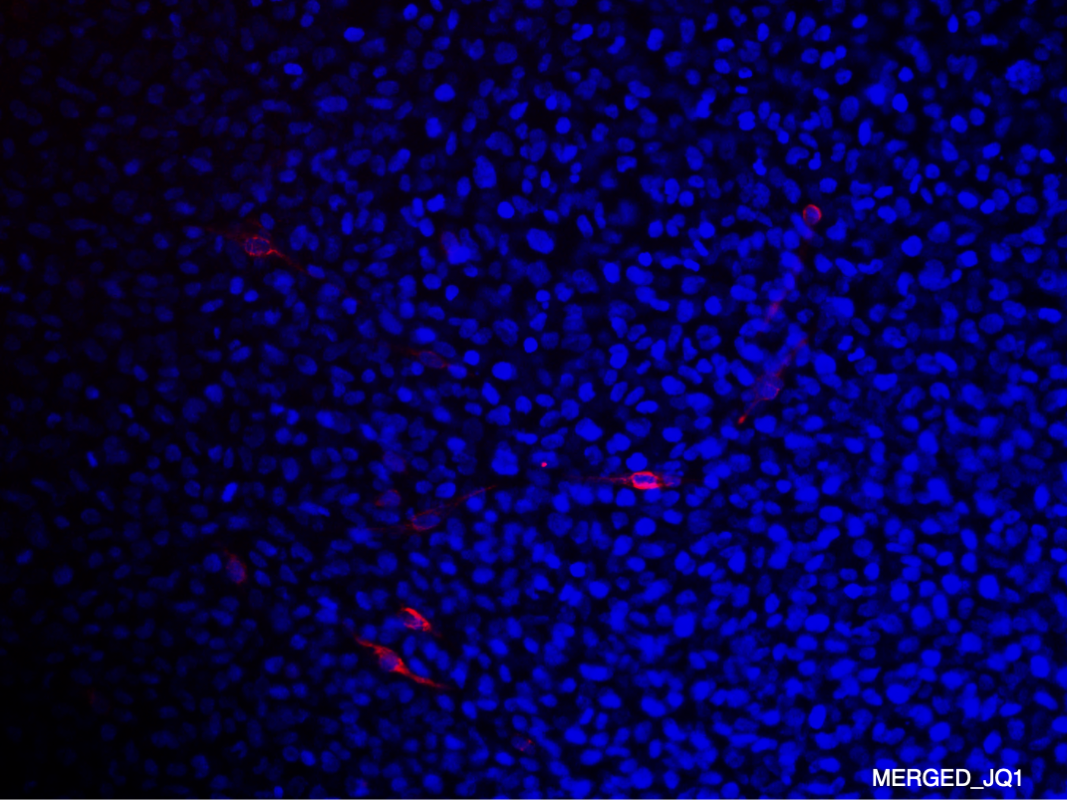

Supplement: Supplementary file 5 — Source Data Fig. 2 [file 44319_2023_33_MOESM5_ESM.zip › Fig.2/Fig. 2B/JQ1/MERGED.tiff]

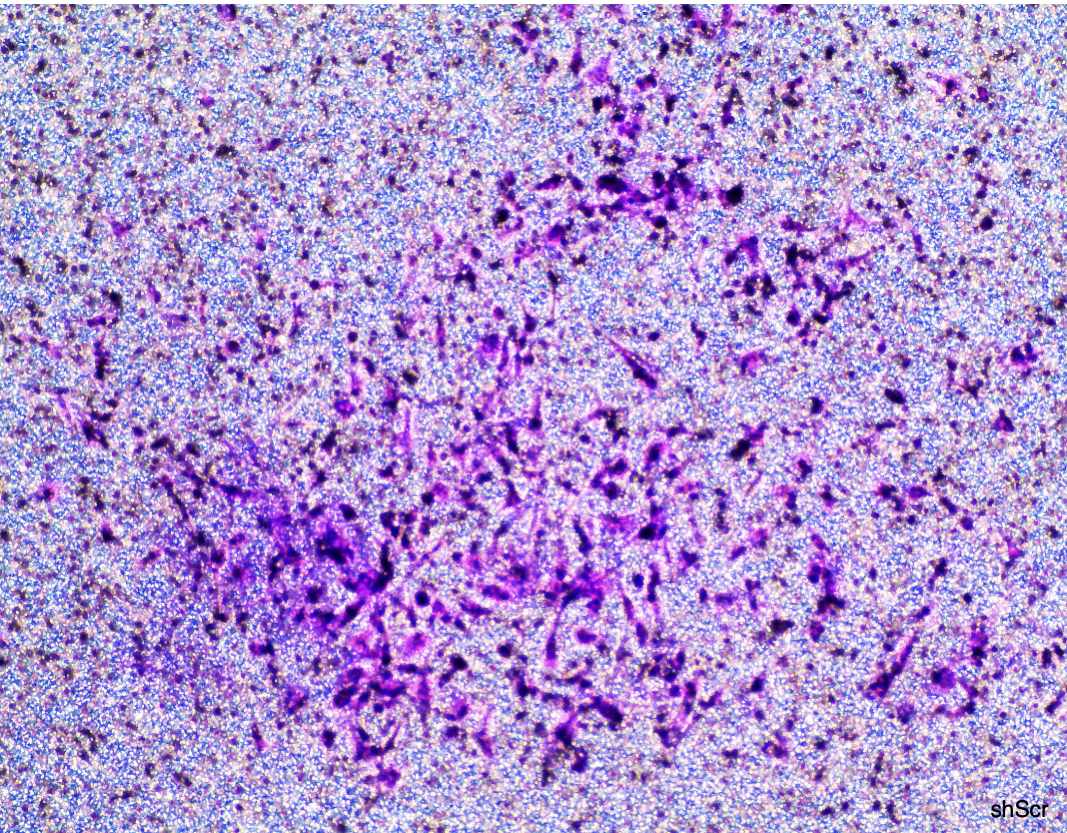

Supplement: Supplementary file 5 — Source Data Fig. 2 [file 44319_2023_33_MOESM5_ESM.zip › Fig.2/Fig. 2L/INVASION/Slide1.tiff]

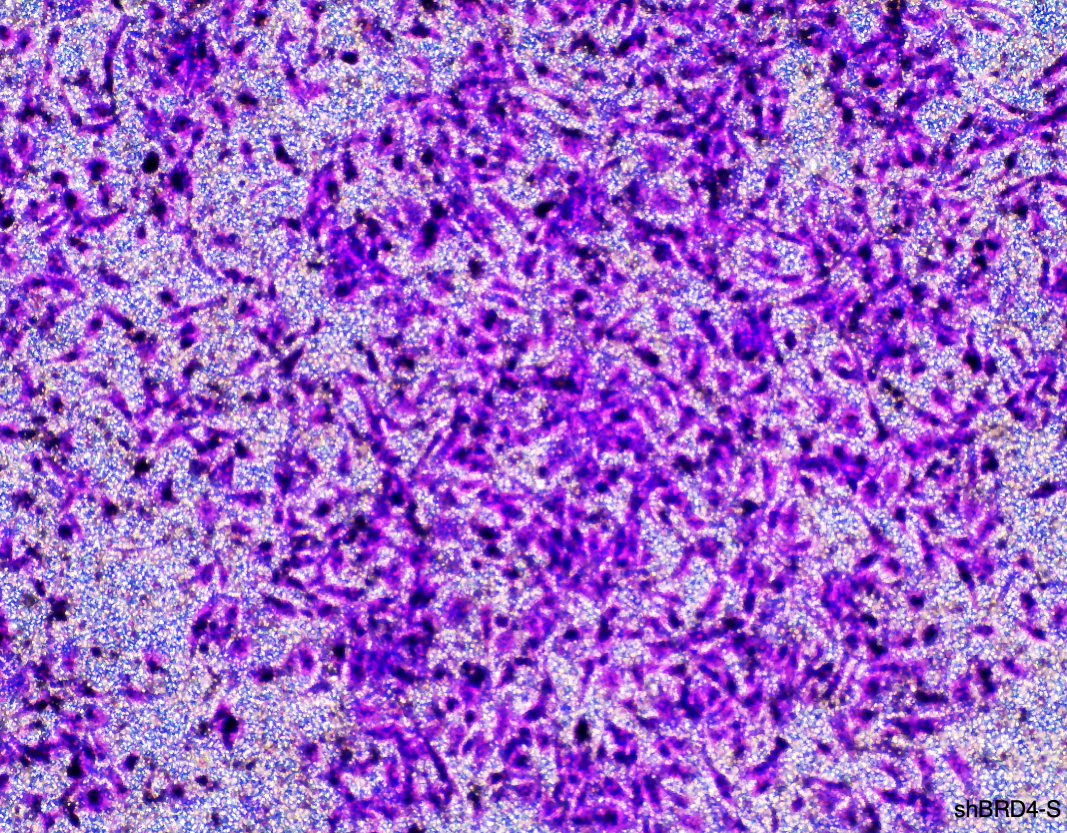

Supplement: Supplementary file 5 — Source Data Fig. 2 [file 44319_2023_33_MOESM5_ESM.zip › Fig.2/Fig. 2L/INVASION/Slide3.tiff]

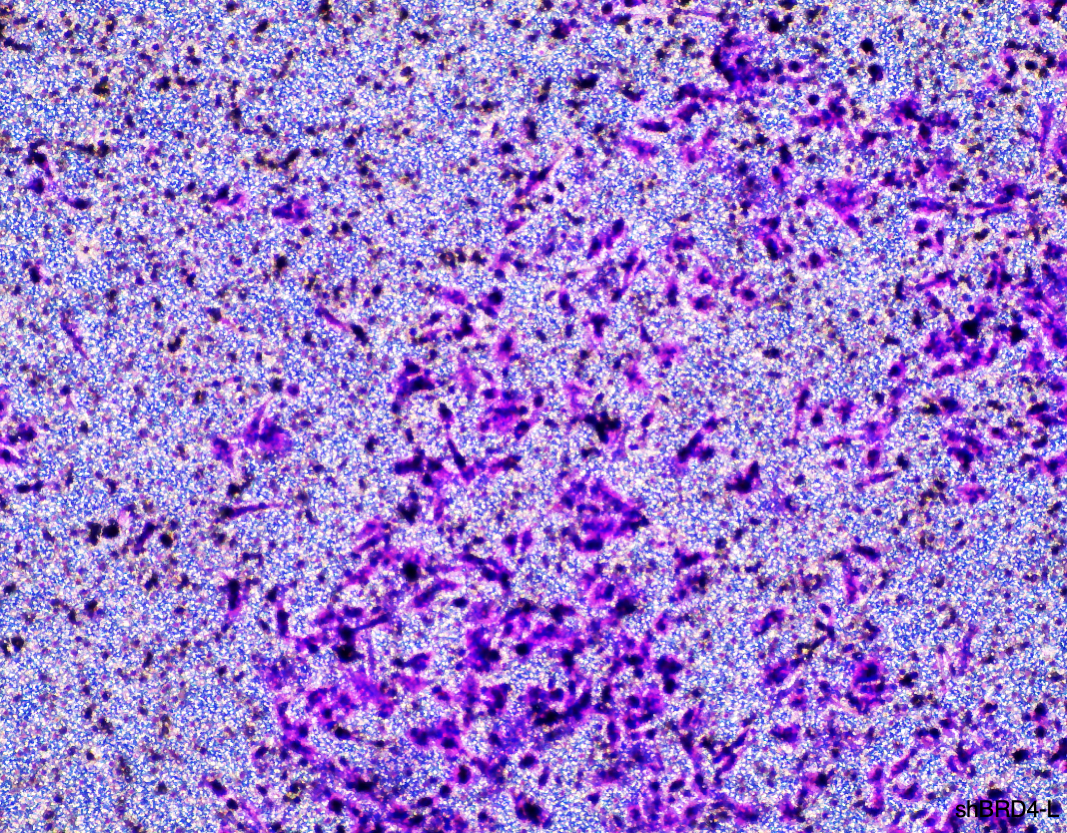

Supplement: Supplementary file 5 — Source Data Fig. 2 [file 44319_2023_33_MOESM5_ESM.zip › Fig.2/Fig. 2L/INVASION/Slide2.tiff]

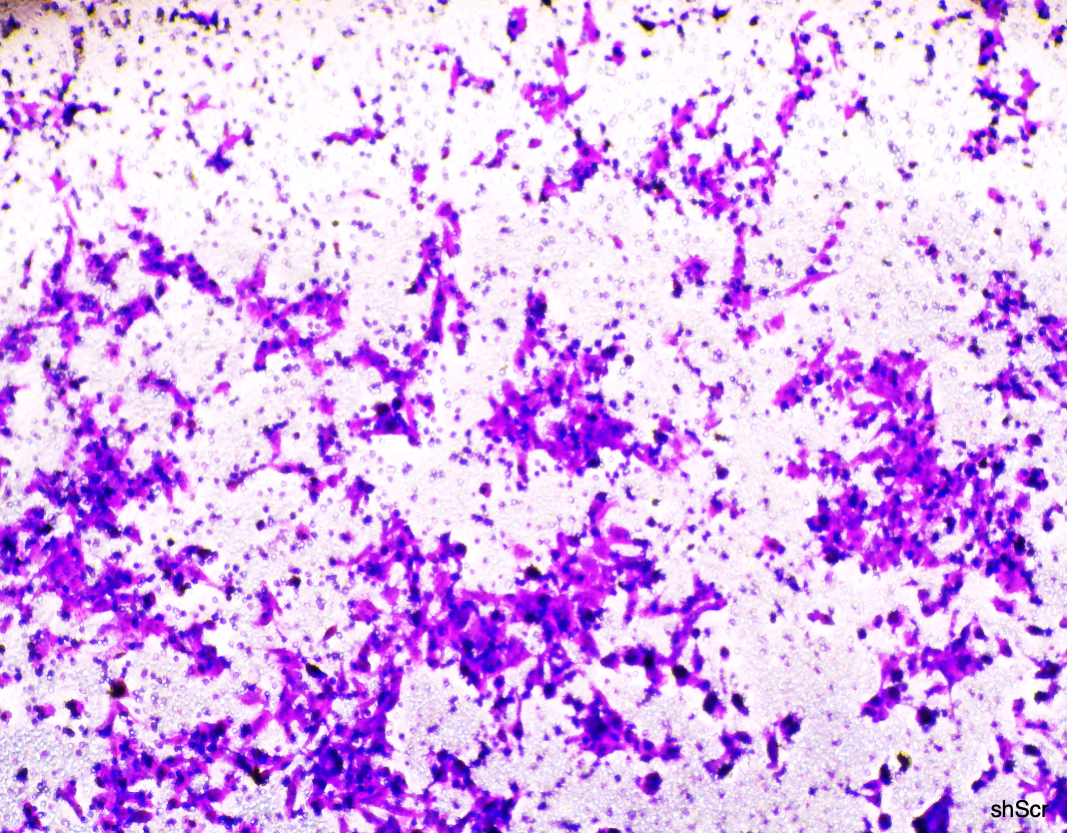

Supplement: Supplementary file 5 — Source Data Fig. 2 [file 44319_2023_33_MOESM5_ESM.zip › Fig.2/Fig. 2L/MIGRATION/shScr.tiff]

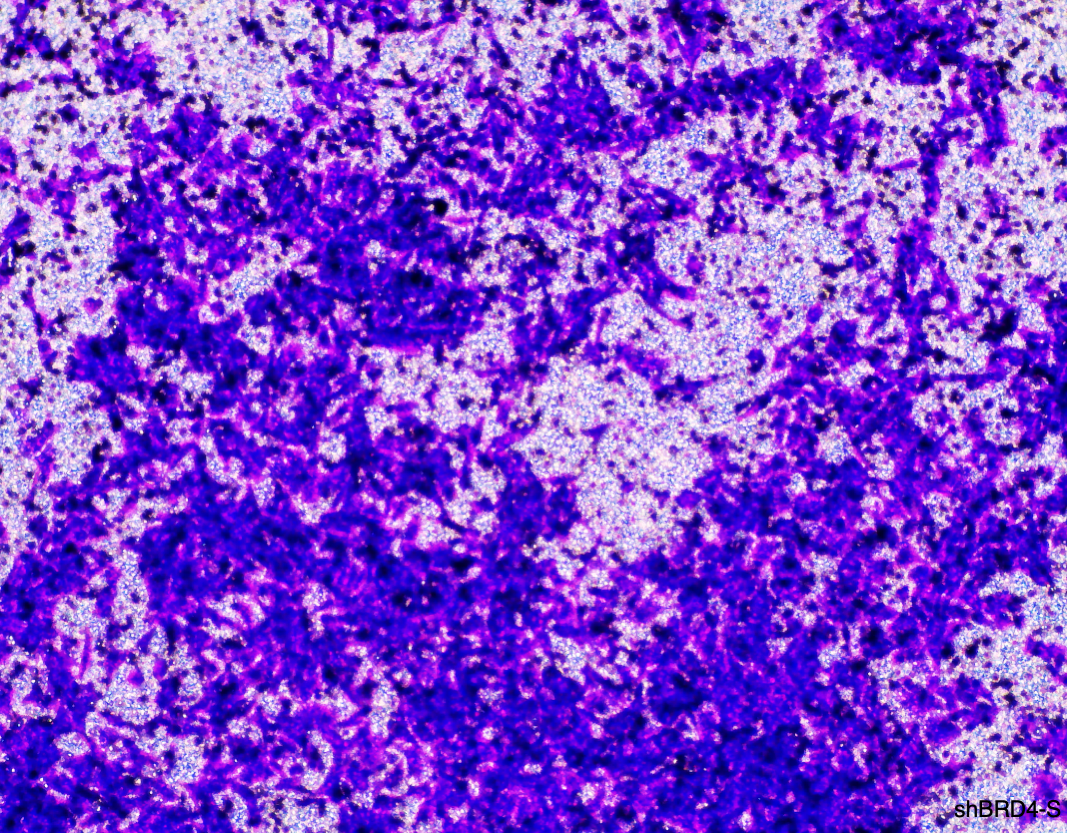

Supplement: Supplementary file 5 — Source Data Fig. 2 [file 44319_2023_33_MOESM5_ESM.zip › Fig.2/Fig. 2L/MIGRATION/shBRD4-S.tiff]

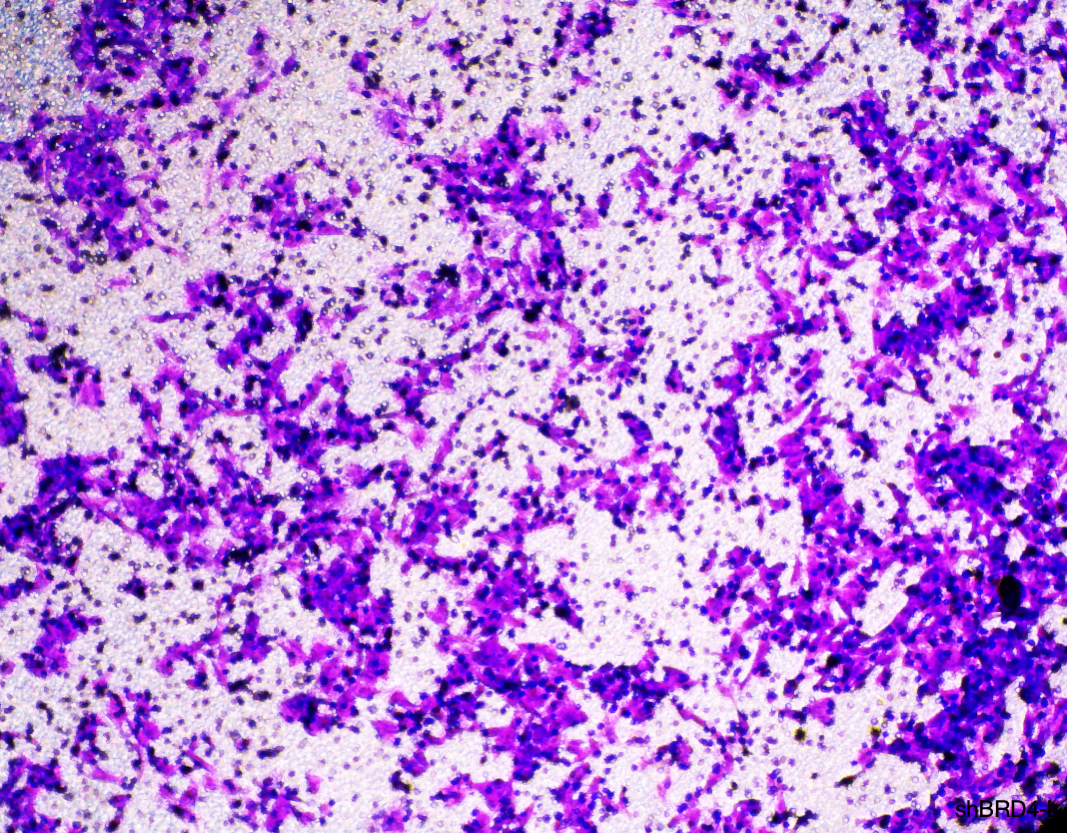

Supplement: Supplementary file 5 — Source Data Fig. 2 [file 44319_2023_33_MOESM5_ESM.zip › Fig.2/Fig. 2L/MIGRATION/shBRD4-L.tiff]

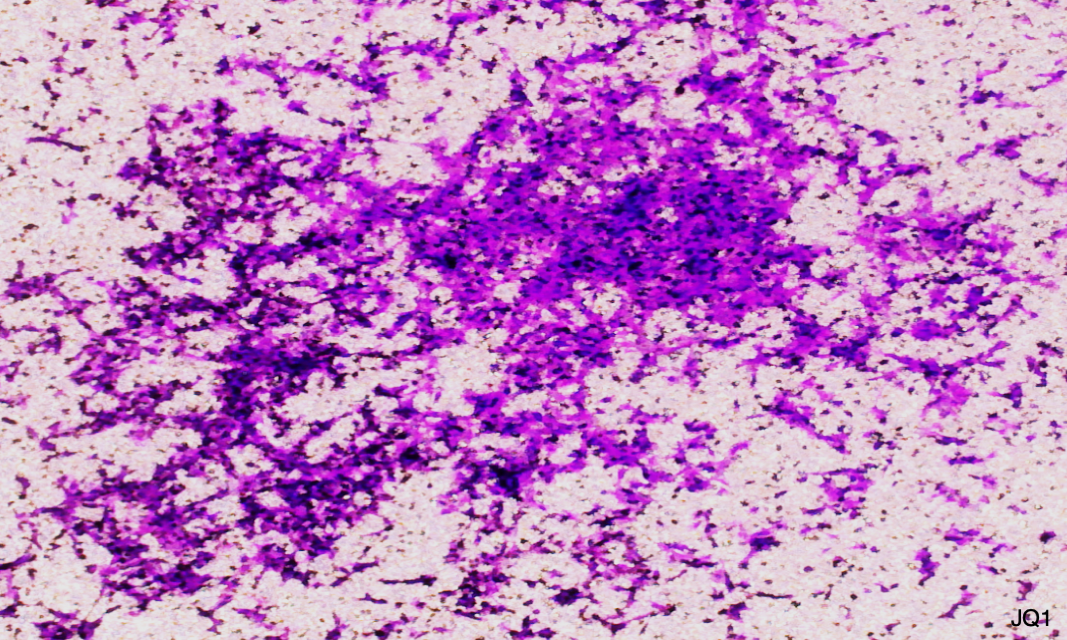

Supplement: Supplementary file 5 — Source Data Fig. 2 [file 44319_2023_33_MOESM5_ESM.zip › Fig.2/Fig. 2D/INVASION/JQ1.tiff]

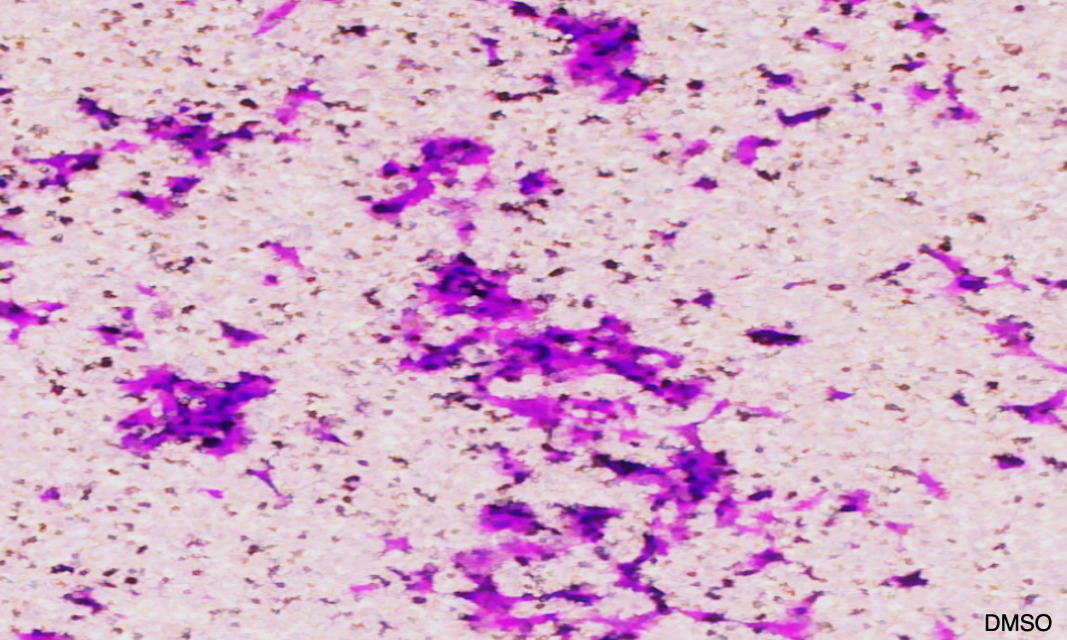

Supplement: Supplementary file 5 — Source Data Fig. 2 [file 44319_2023_33_MOESM5_ESM.zip › Fig.2/Fig. 2D/INVASION/DMSO.tiff]

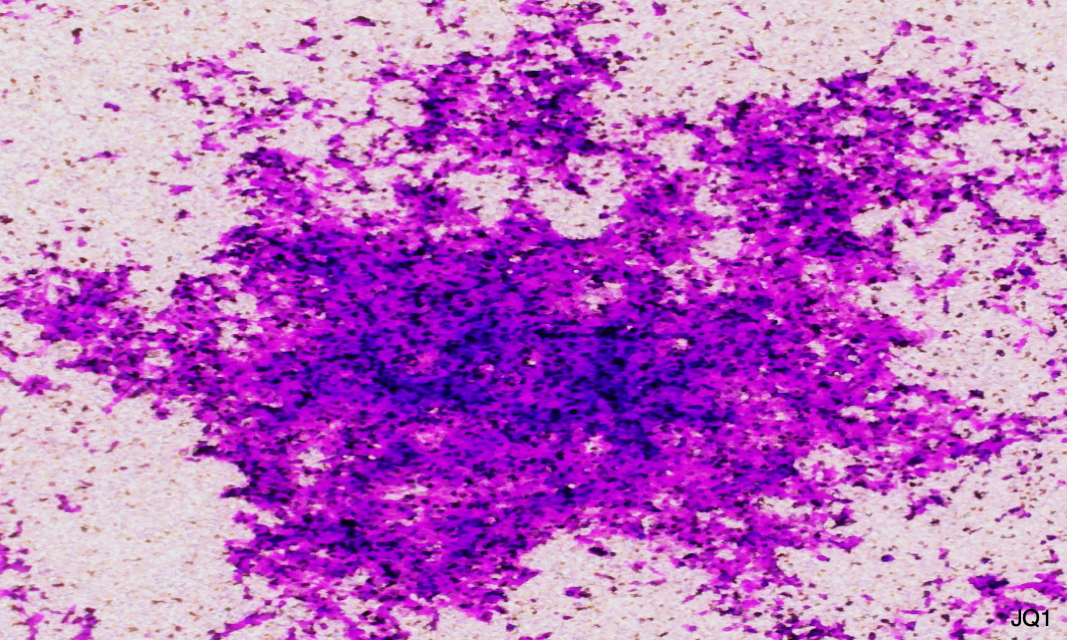

Supplement: Supplementary file 5 — Source Data Fig. 2 [file 44319_2023_33_MOESM5_ESM.zip › Fig.2/Fig. 2D/MIGRATION/JQ1.tiff]

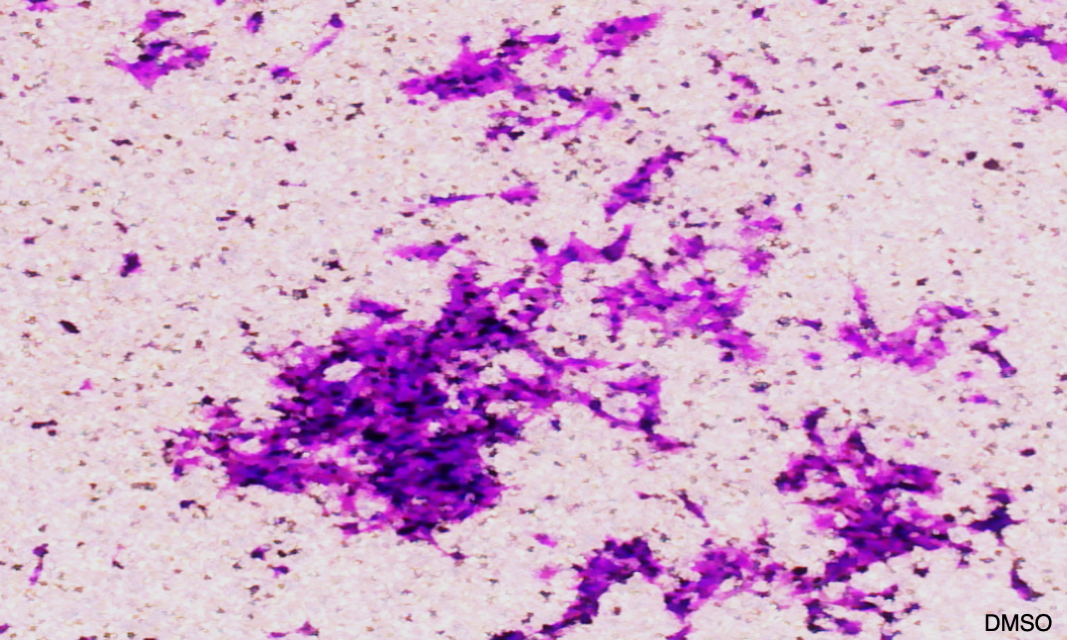

Supplement: Supplementary file 5 — Source Data Fig. 2 [file 44319_2023_33_MOESM5_ESM.zip › Fig.2/Fig. 2D/MIGRATION/DMSO.tiff]

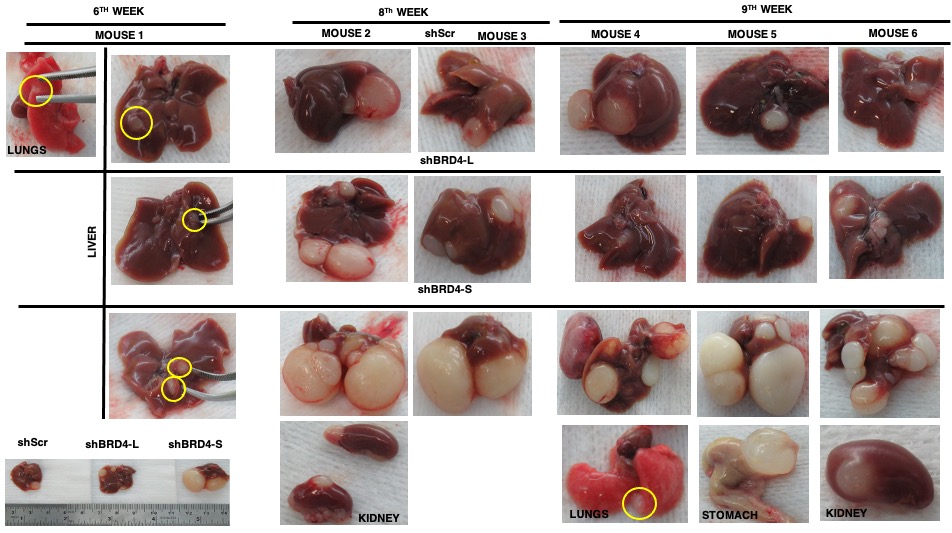

Supplement: Supplementary file 6 — Source Data Fig. 3 [file 44319_2023_33_MOESM6_ESM.zip › Fig.3/Fig. 3D.jpg]

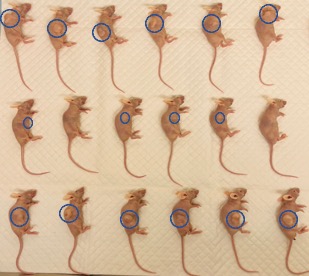

Supplement: Supplementary file 6 — Source Data Fig. 3 [file 44319_2023_33_MOESM6_ESM.zip › Fig.3/Fig. 3A/Fig. 3A.jpg]

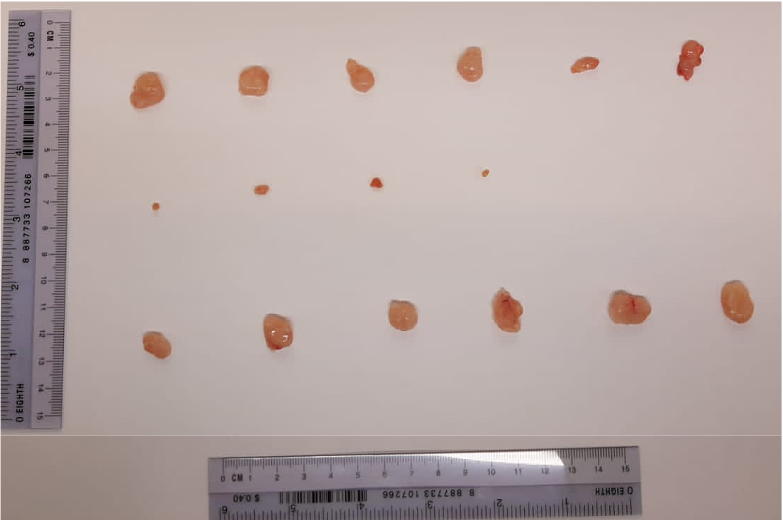

Supplement: Supplementary file 6 — Source Data Fig. 3 [file 44319_2023_33_MOESM6_ESM.zip › Fig.3/Fig. 3A/3A.png]

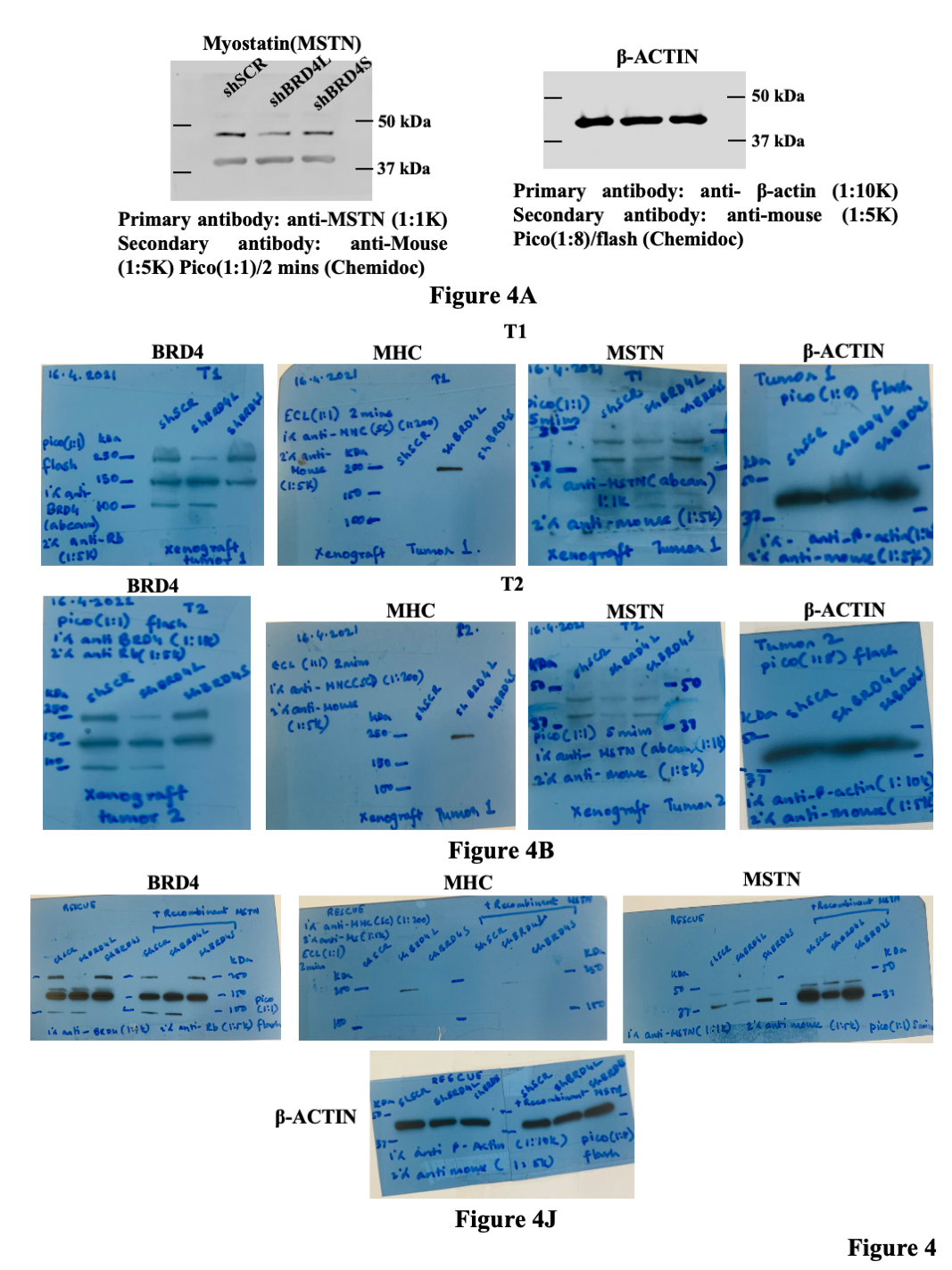

Supplement: Supplementary file 7 — Source Data Fig. 4 [file 44319_2023_33_MOESM7_ESM.zip › Fig.4/Fig 4ABJ_Western.tiff]

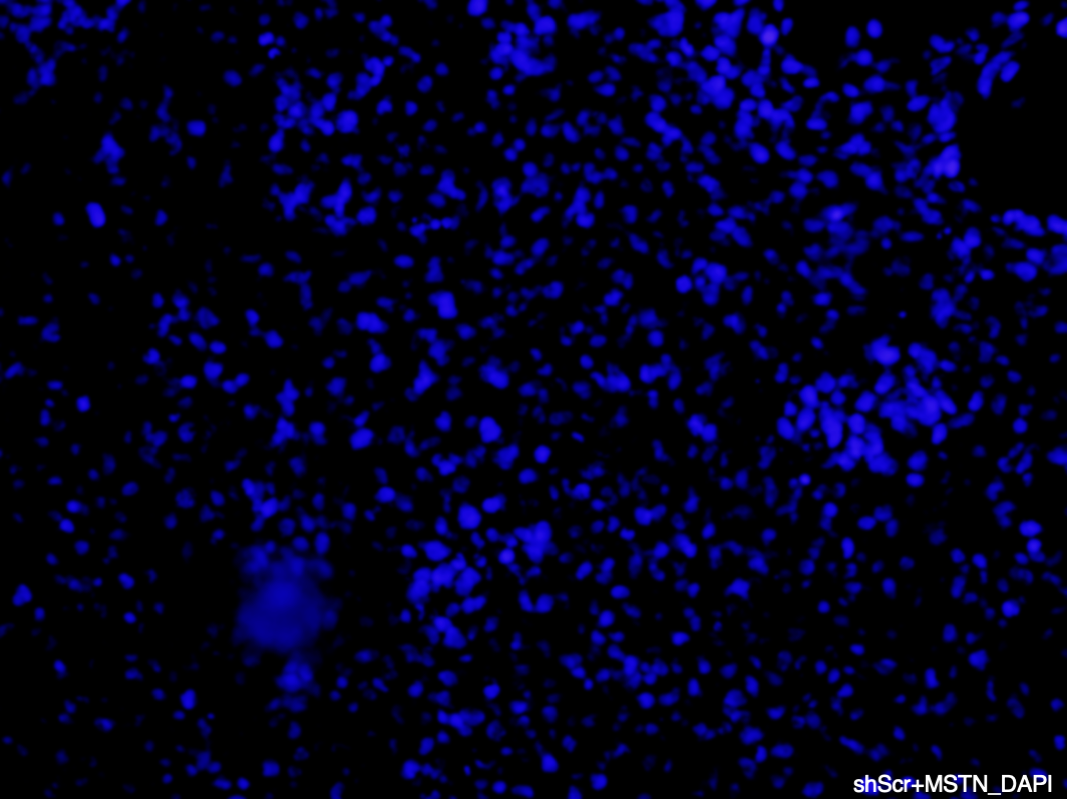

Supplement: Supplementary file 7 — Source Data Fig. 4 [file 44319_2023_33_MOESM7_ESM.zip › Fig.4/Fig. 4I/shScr/shScr+MSTN_DAPI.tiff]

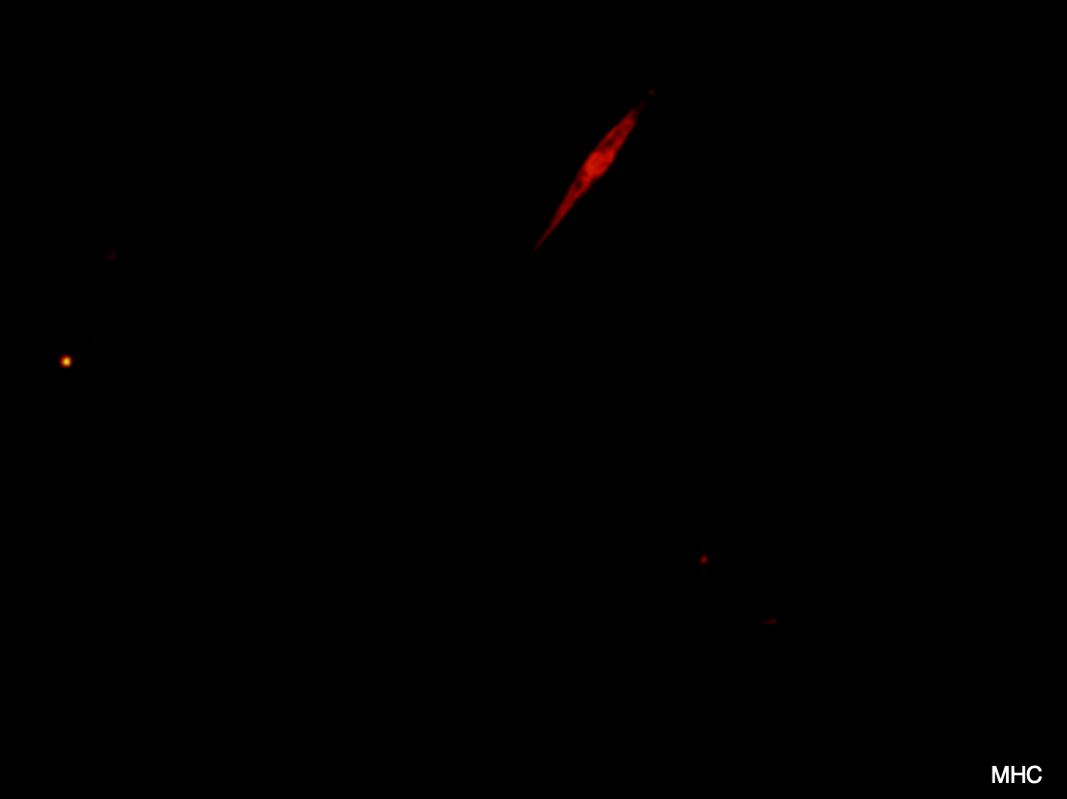

Supplement: Supplementary file 7 — Source Data Fig. 4 [file 44319_2023_33_MOESM7_ESM.zip › Fig.4/Fig. 4I/shScr/MHC.tiff]

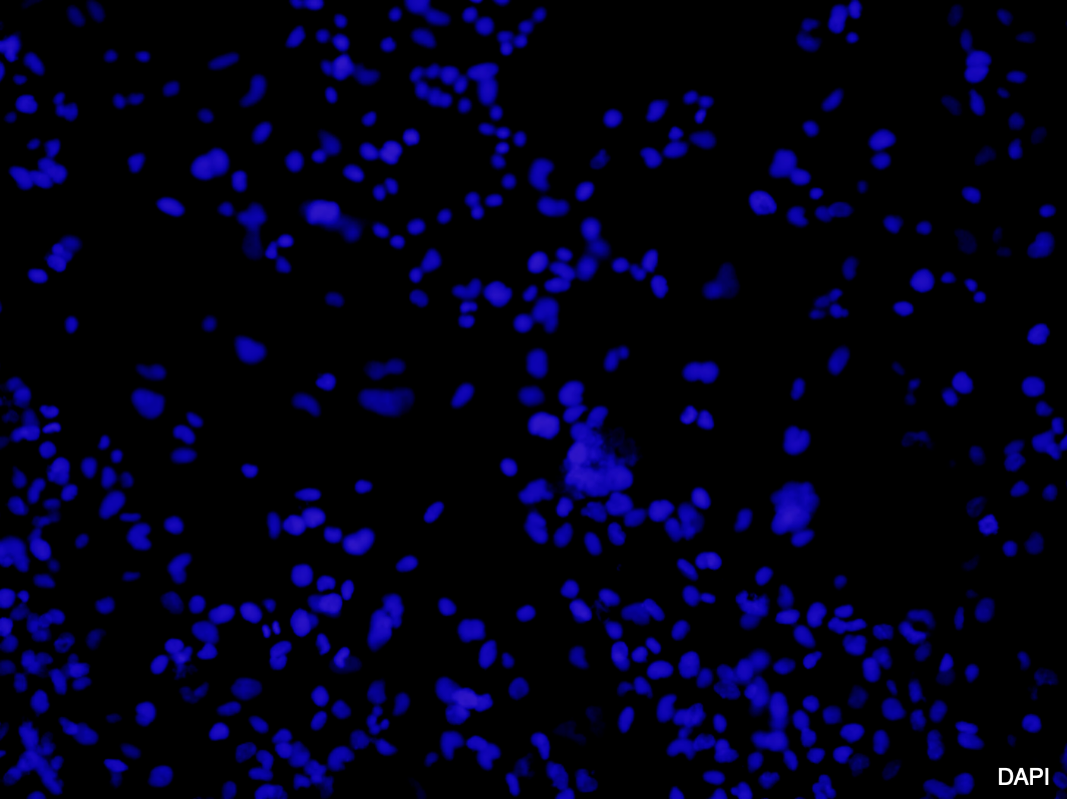

Supplement: Supplementary file 7 — Source Data Fig. 4 [file 44319_2023_33_MOESM7_ESM.zip › Fig.4/Fig. 4I/shScr/DAPI.tiff]

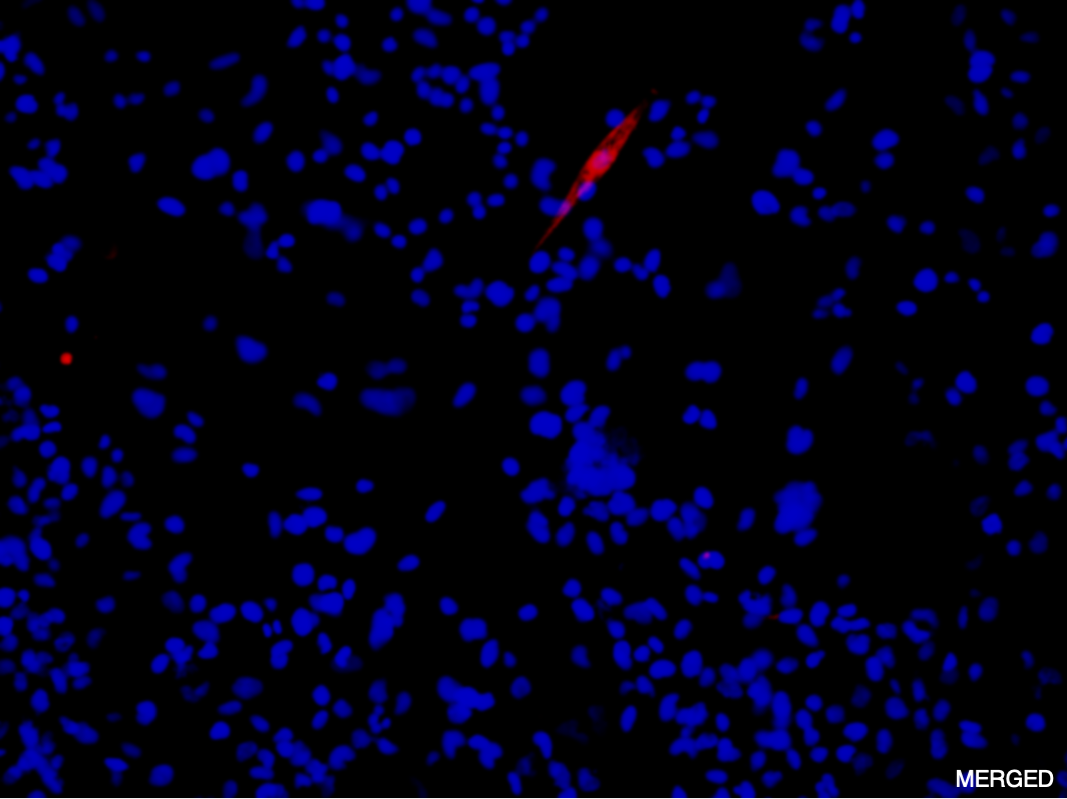

Supplement: Supplementary file 7 — Source Data Fig. 4 [file 44319_2023_33_MOESM7_ESM.zip › Fig.4/Fig. 4I/shScr/MERGED.tiff]

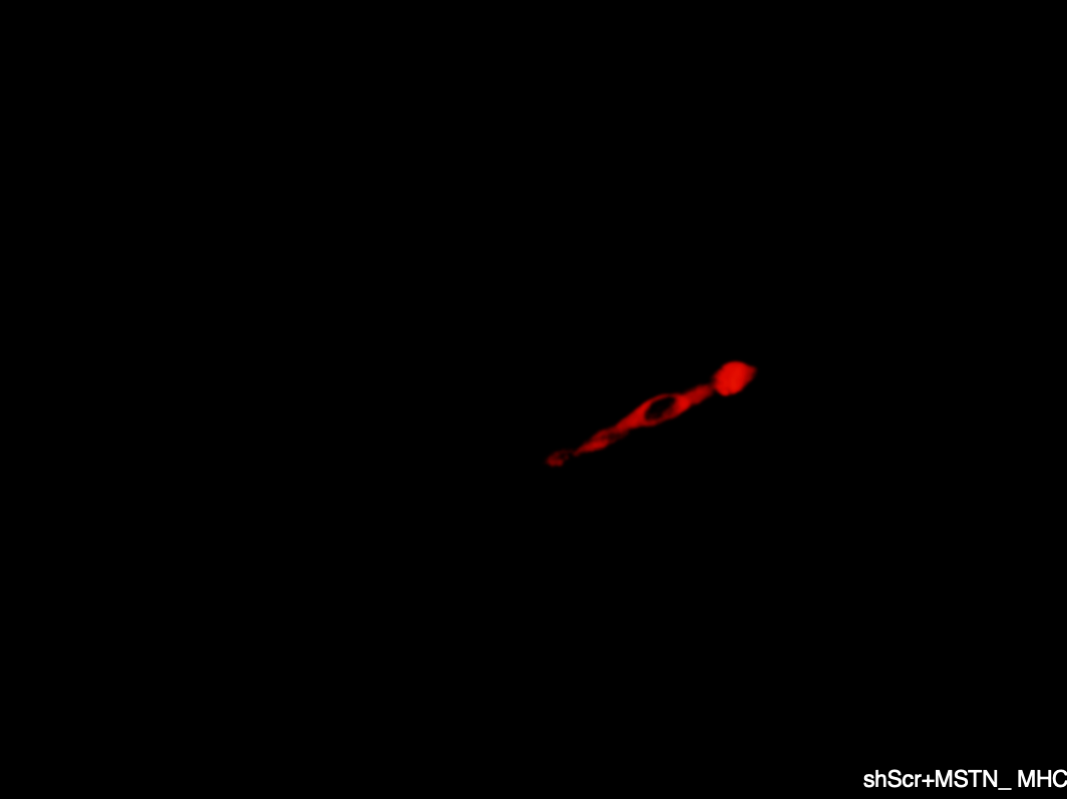

Supplement: Supplementary file 7 — Source Data Fig. 4 [file 44319_2023_33_MOESM7_ESM.zip › Fig.4/Fig. 4I/shScr/shScr+MSTN_MHC.tiff]
