# Supplementary figures and images for: BRD4 isoforms have distinct roles in tumour progression and metastasis in rhabdomyosarcoma (part 2 of 2)
Source: EMBO Rep. 2024 Jan 8;25(2):832–52. doi: 10.1038/s44319-023-00033-1 (PMC10897194; doi:10.1038/s44319-023-00033-1)

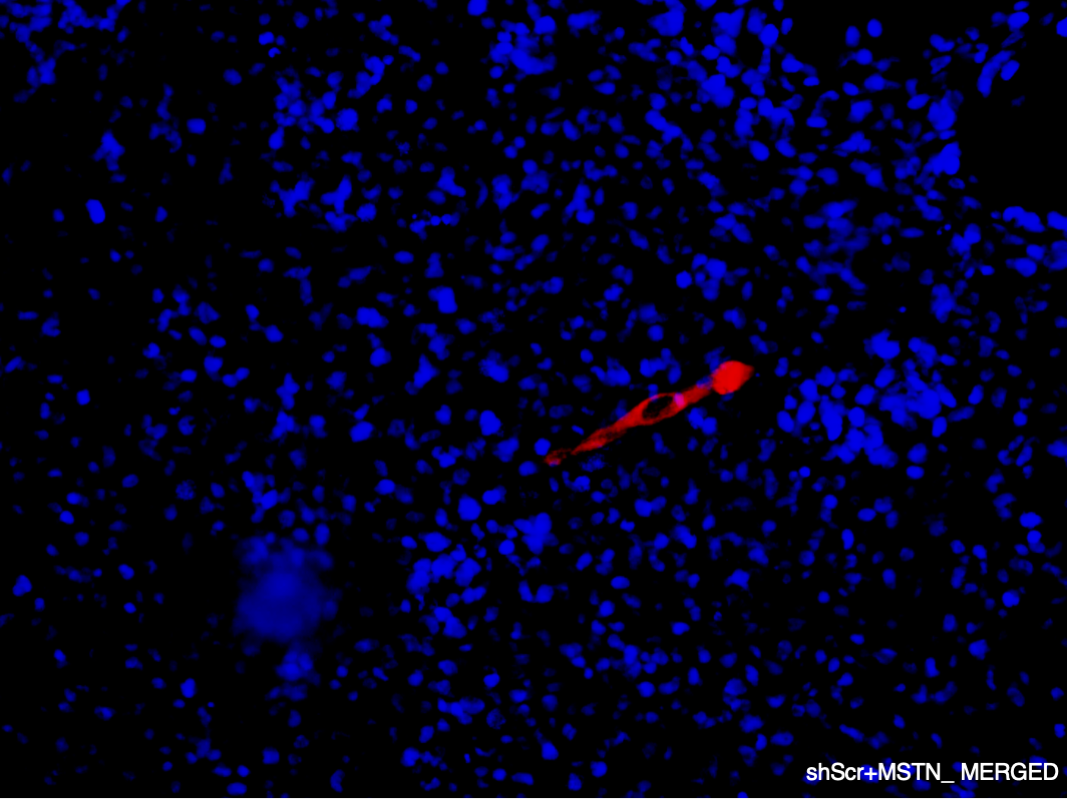

Supplement: Supplementary file 7 — Source Data Fig. 4 [file 44319_2023_33_MOESM7_ESM.zip › Fig.4/Fig. 4I/shScr/shScr+MSTN_MERGED.tiff]

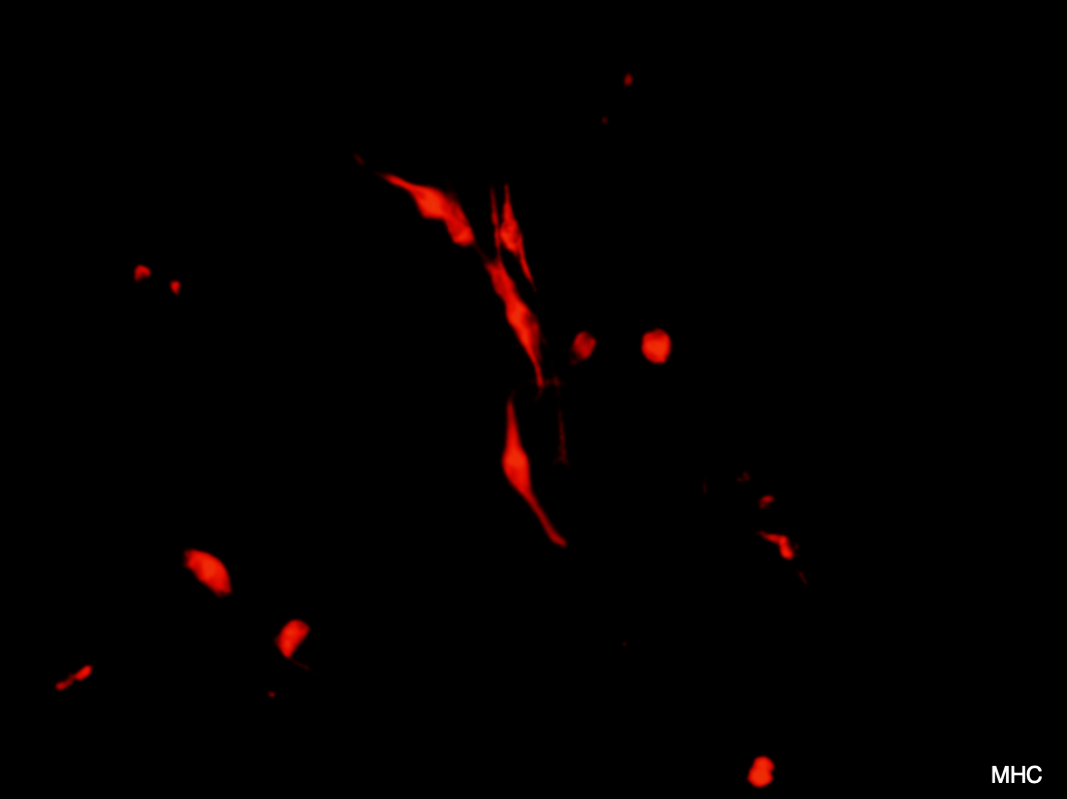

Supplement: Supplementary file 7 — Source Data Fig. 4 [file 44319_2023_33_MOESM7_ESM.zip › Fig.4/Fig. 4I/shBRD4-L/MHC.tiff]

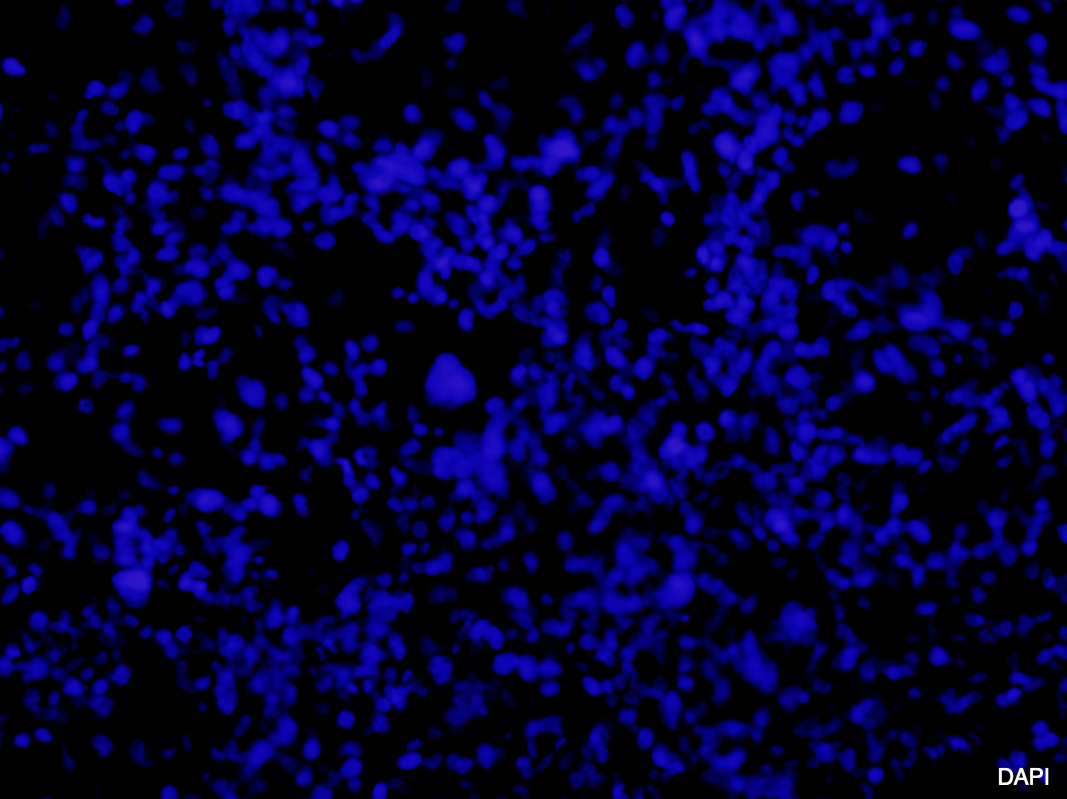

Supplement: Supplementary file 7 — Source Data Fig. 4 [file 44319_2023_33_MOESM7_ESM.zip › Fig.4/Fig. 4I/shBRD4-L/DAPI.tiff]

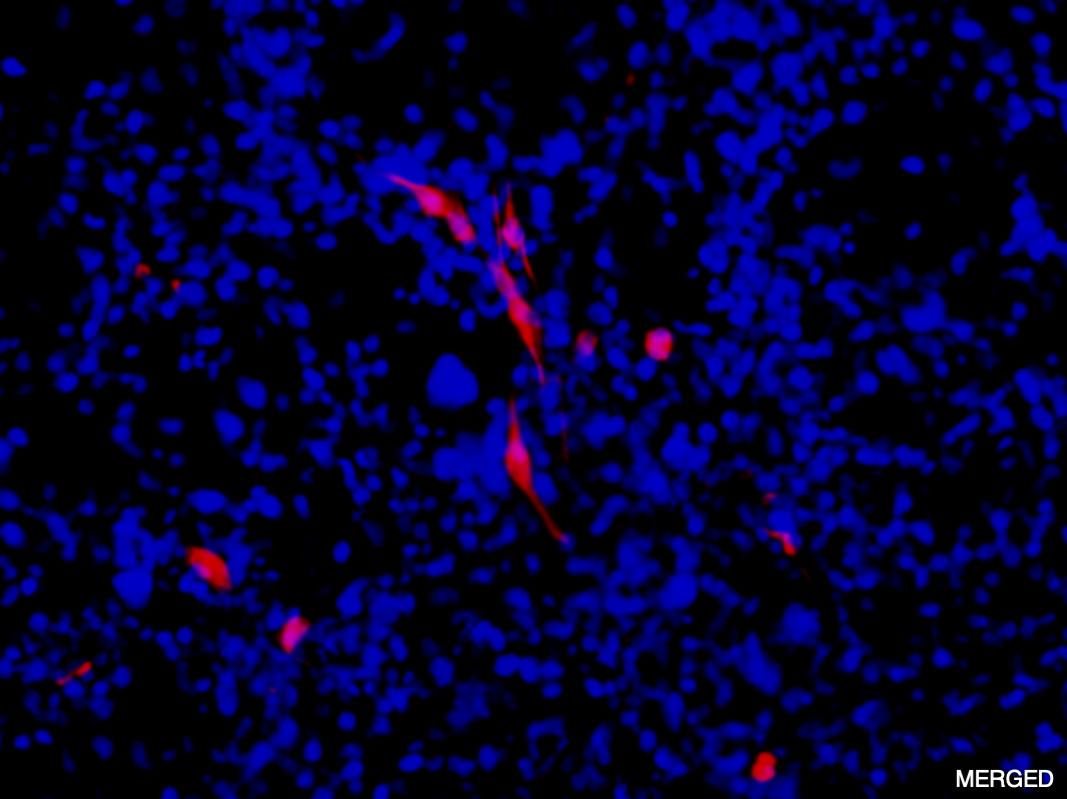

Supplement: Supplementary file 7 — Source Data Fig. 4 [file 44319_2023_33_MOESM7_ESM.zip › Fig.4/Fig. 4I/shBRD4-L/MERGED.tiff]

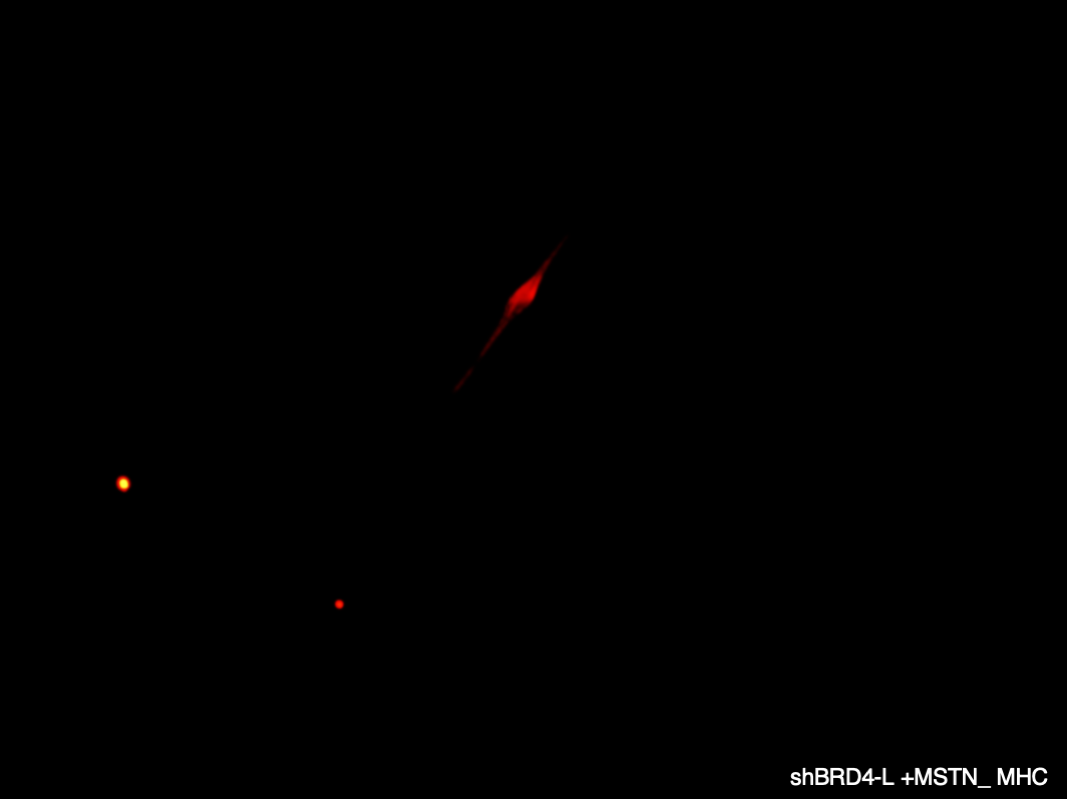

Supplement: Supplementary file 7 — Source Data Fig. 4 [file 44319_2023_33_MOESM7_ESM.zip › Fig.4/Fig. 4I/shBRD4-L/shBRD4-L_MHC.tiff]

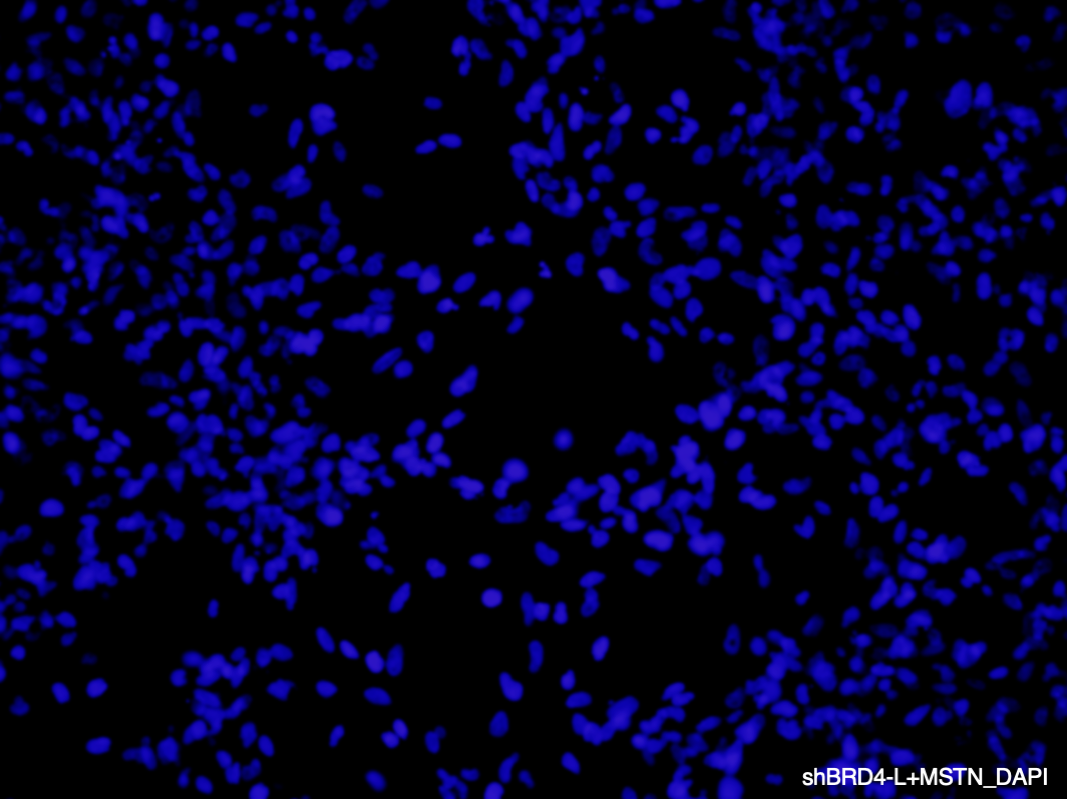

Supplement: Supplementary file 7 — Source Data Fig. 4 [file 44319_2023_33_MOESM7_ESM.zip › Fig.4/Fig. 4I/shBRD4-L/shBRD4-L_DAPI.tiff]

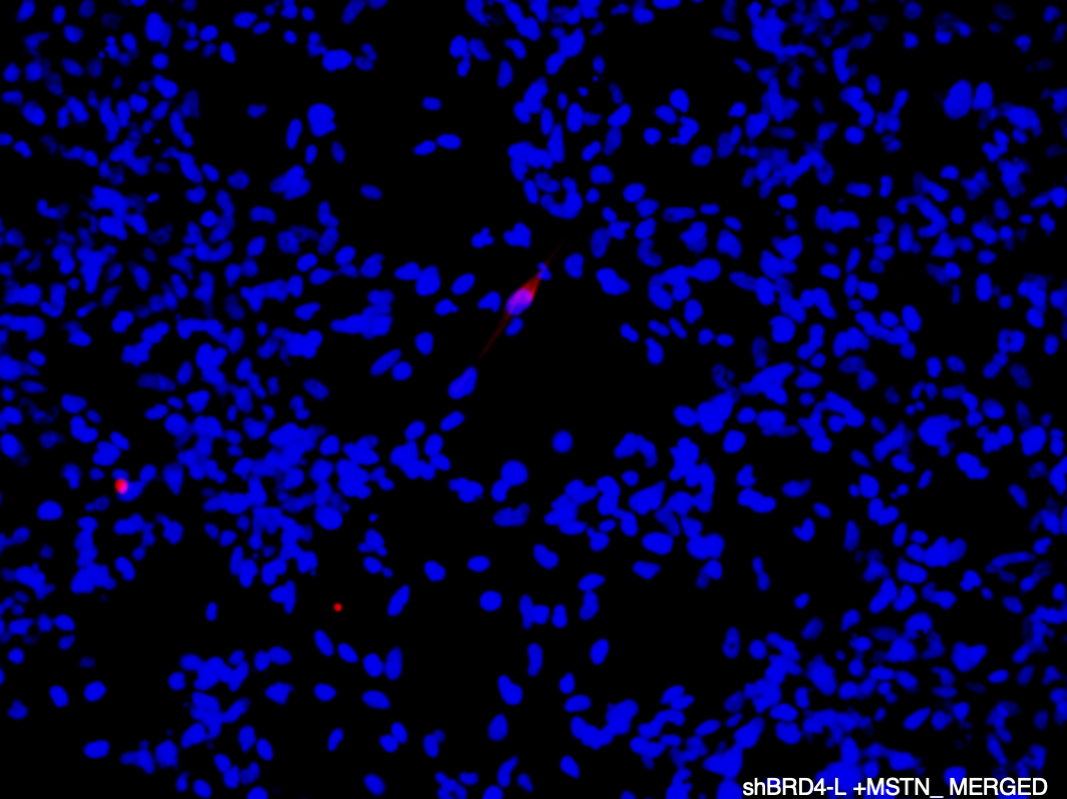

Supplement: Supplementary file 7 — Source Data Fig. 4 [file 44319_2023_33_MOESM7_ESM.zip › Fig.4/Fig. 4I/shBRD4-L/shBRD4-L_MERGED.tiff]

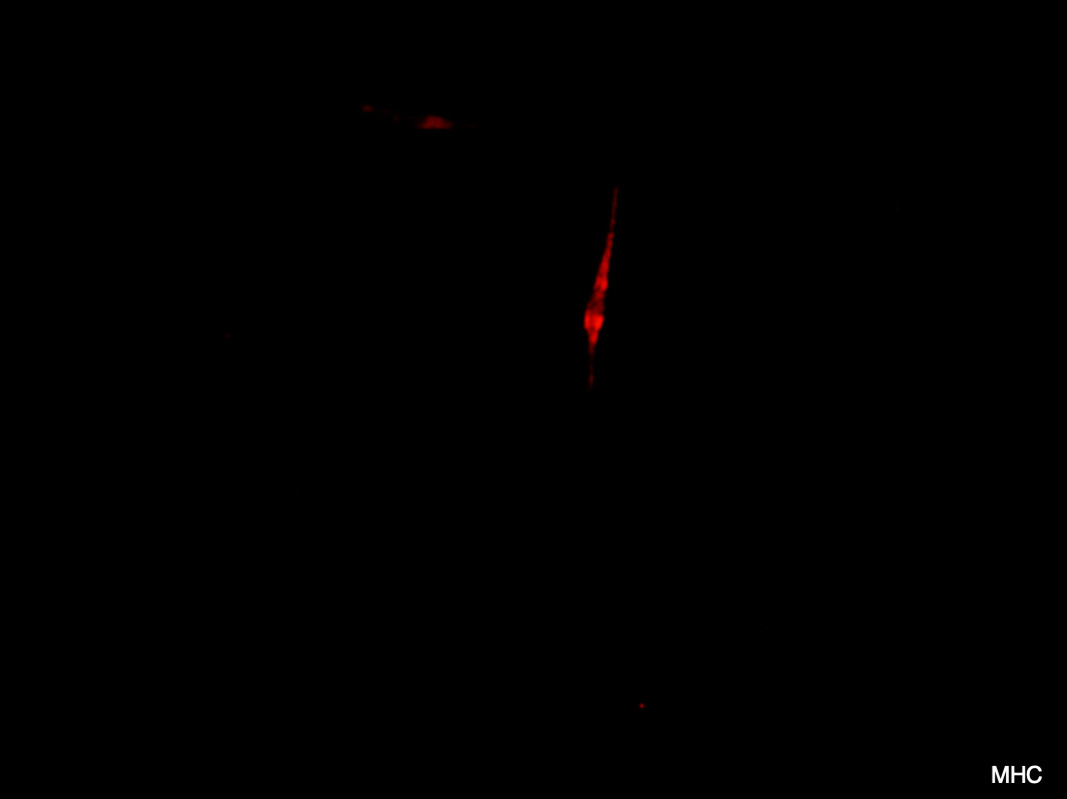

Supplement: Supplementary file 7 — Source Data Fig. 4 [file 44319_2023_33_MOESM7_ESM.zip › Fig.4/Fig. 4I/shBRD4-S/MHC.tiff]

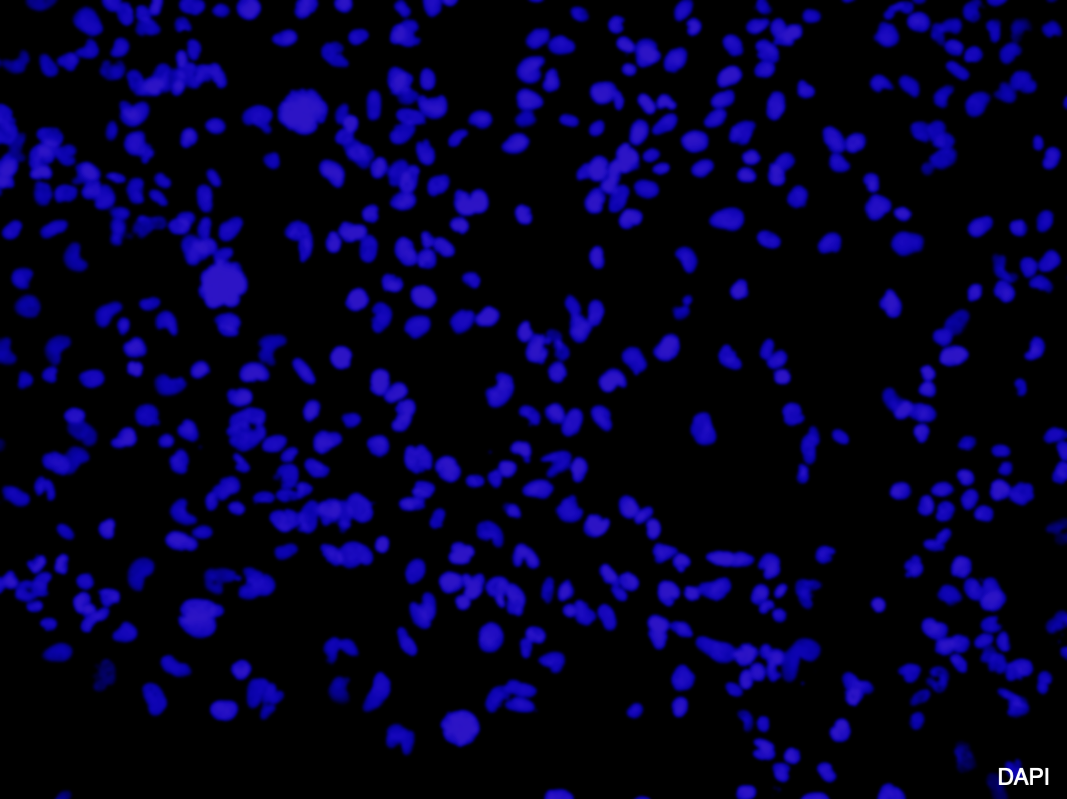

Supplement: Supplementary file 7 — Source Data Fig. 4 [file 44319_2023_33_MOESM7_ESM.zip › Fig.4/Fig. 4I/shBRD4-S/DAPI.tiff]

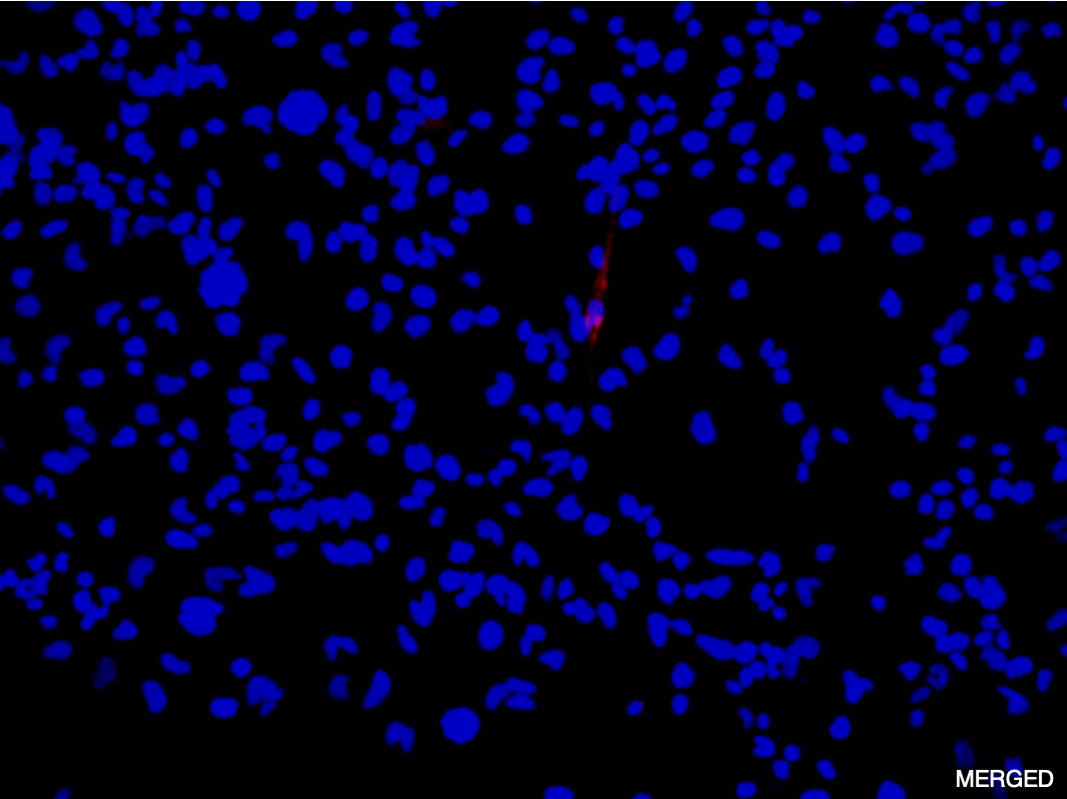

Supplement: Supplementary file 7 — Source Data Fig. 4 [file 44319_2023_33_MOESM7_ESM.zip › Fig.4/Fig. 4I/shBRD4-S/MERGED.tiff]

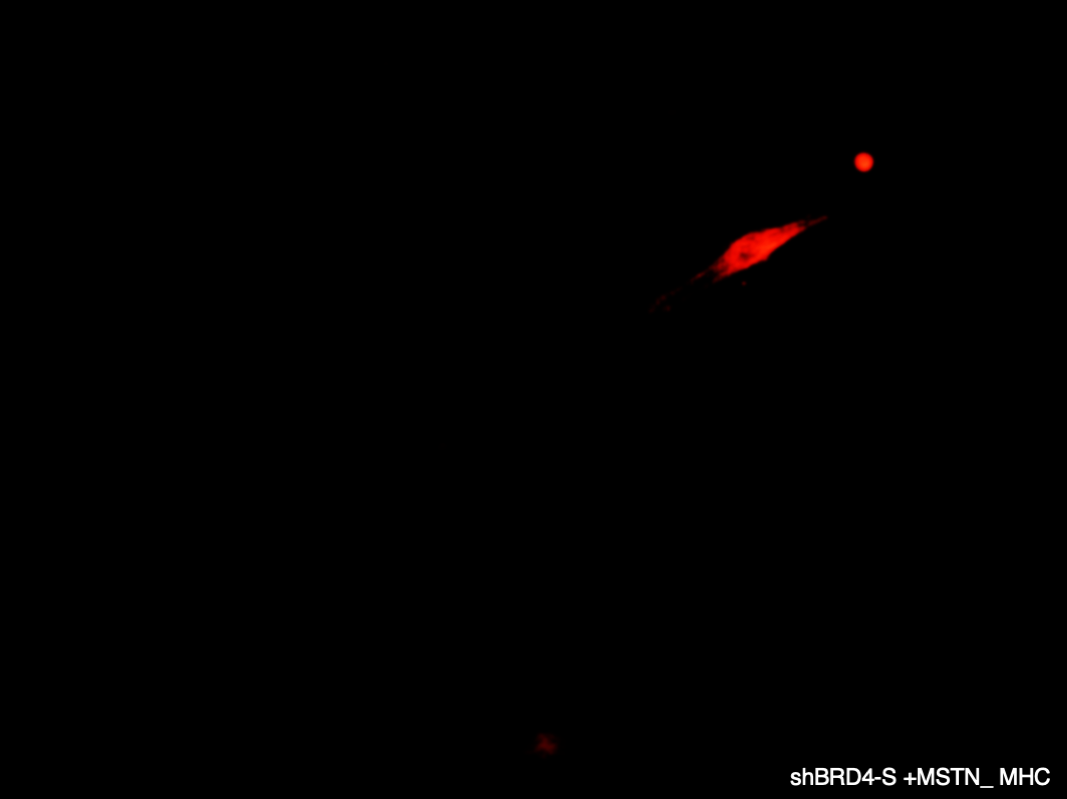

Supplement: Supplementary file 7 — Source Data Fig. 4 [file 44319_2023_33_MOESM7_ESM.zip › Fig.4/Fig. 4I/shBRD4-S/shBRD4_S_MHC.tiff]

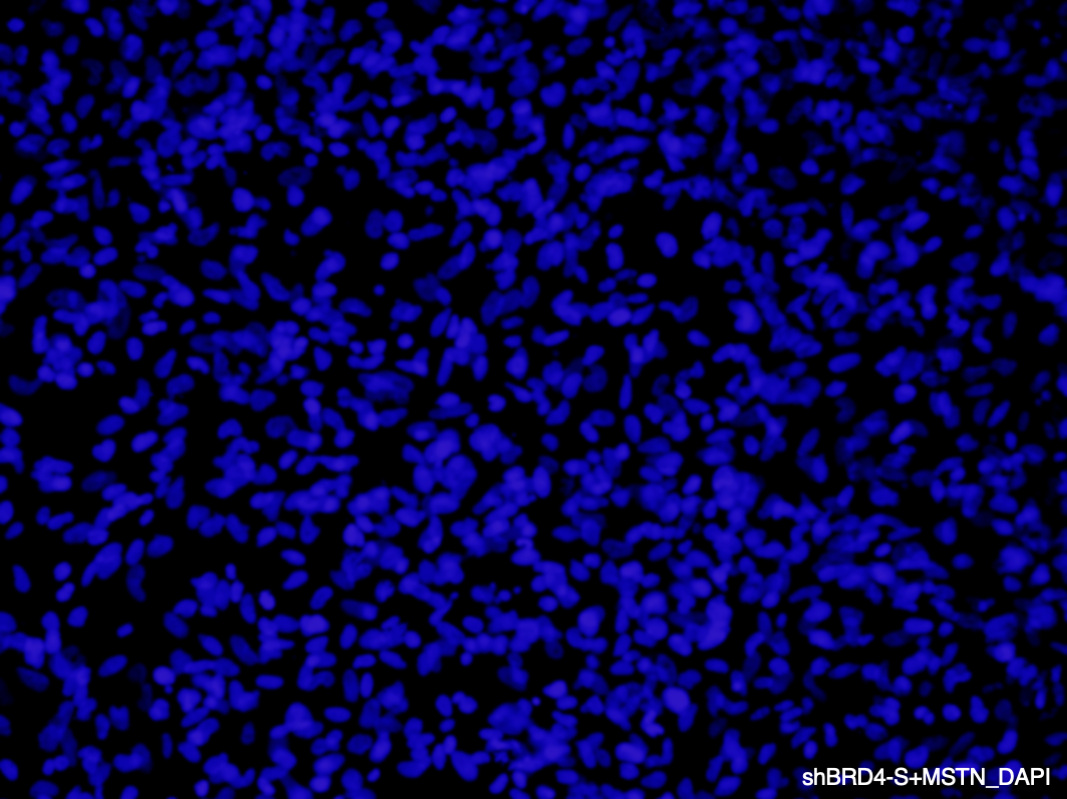

Supplement: Supplementary file 7 — Source Data Fig. 4 [file 44319_2023_33_MOESM7_ESM.zip › Fig.4/Fig. 4I/shBRD4-S/shBRD4-S_DAPI.tiff]

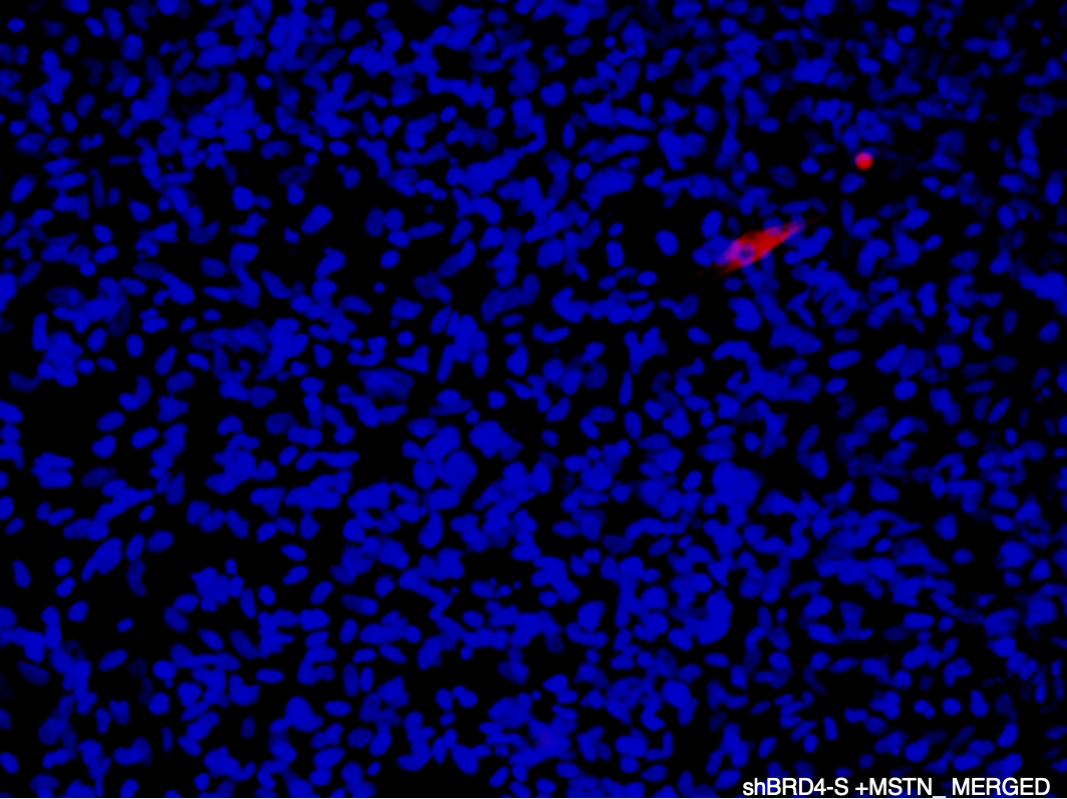

Supplement: Supplementary file 7 — Source Data Fig. 4 [file 44319_2023_33_MOESM7_ESM.zip › Fig.4/Fig. 4I/shBRD4-S/shBRD4-S_MERGED.tiff]

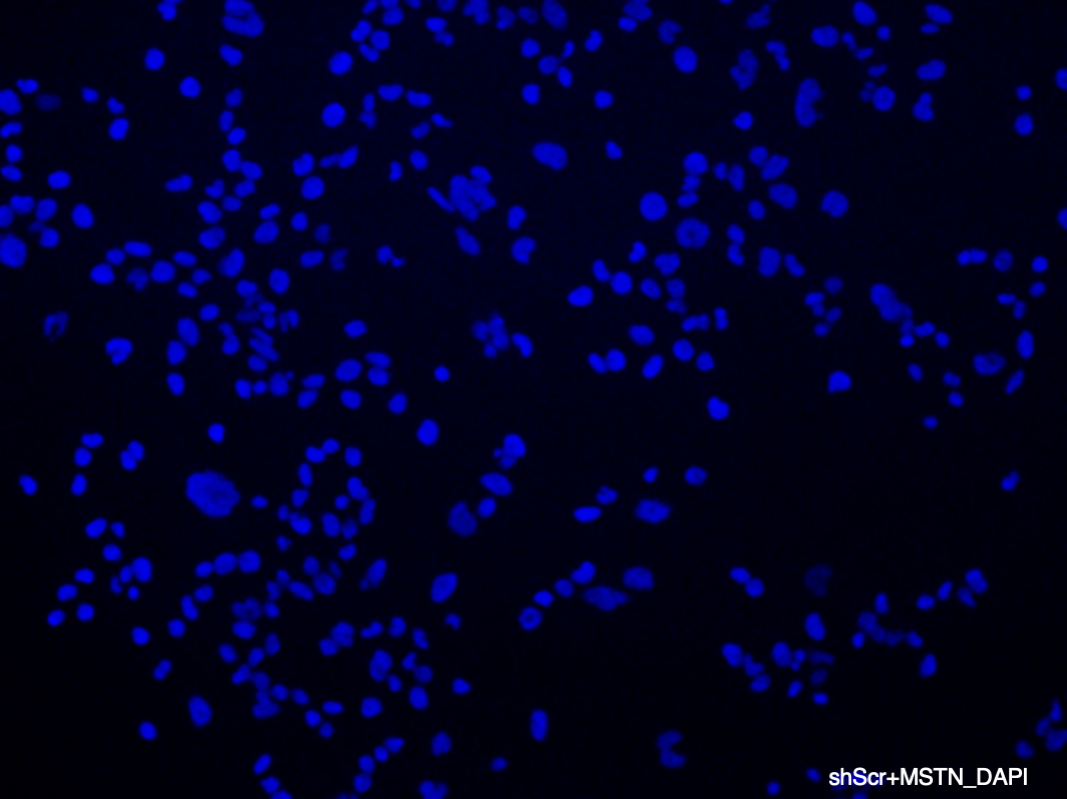

Supplement: Supplementary file 7 — Source Data Fig. 4 [file 44319_2023_33_MOESM7_ESM.zip › Fig.4/Fig. 4H/shScr/shScr+MSTN_DAPI.tiff]

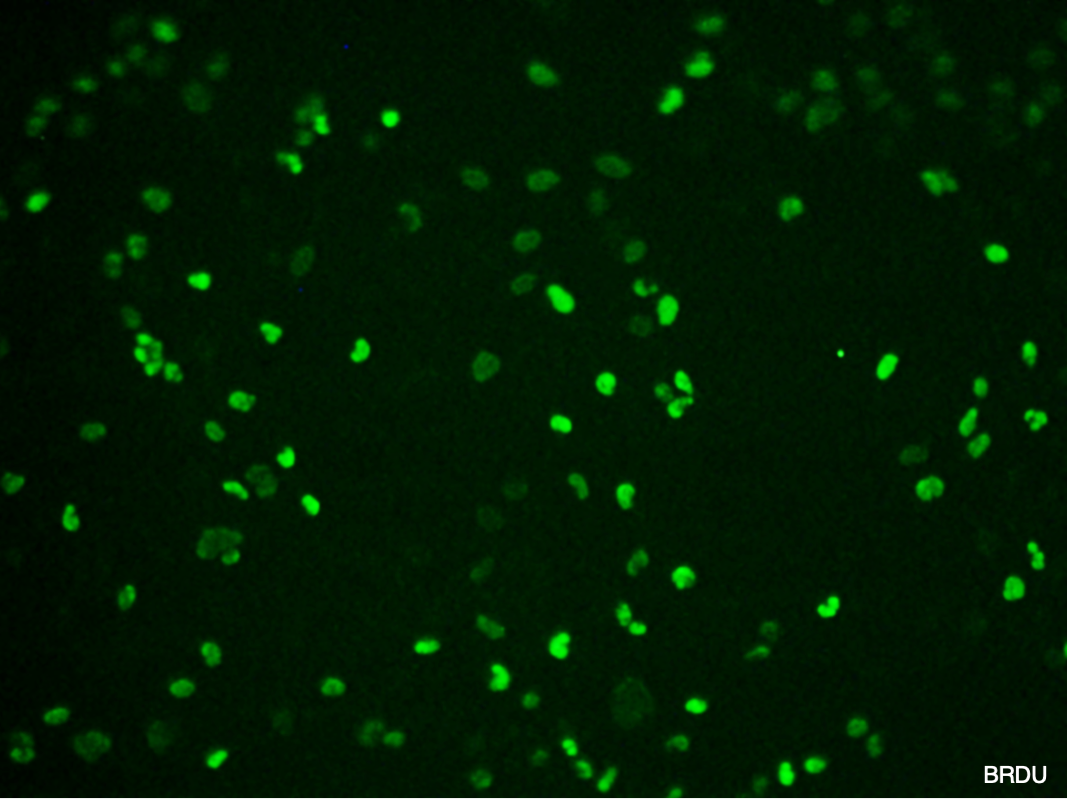

Supplement: Supplementary file 7 — Source Data Fig. 4 [file 44319_2023_33_MOESM7_ESM.zip › Fig.4/Fig. 4H/shScr/BRDU.tiff]

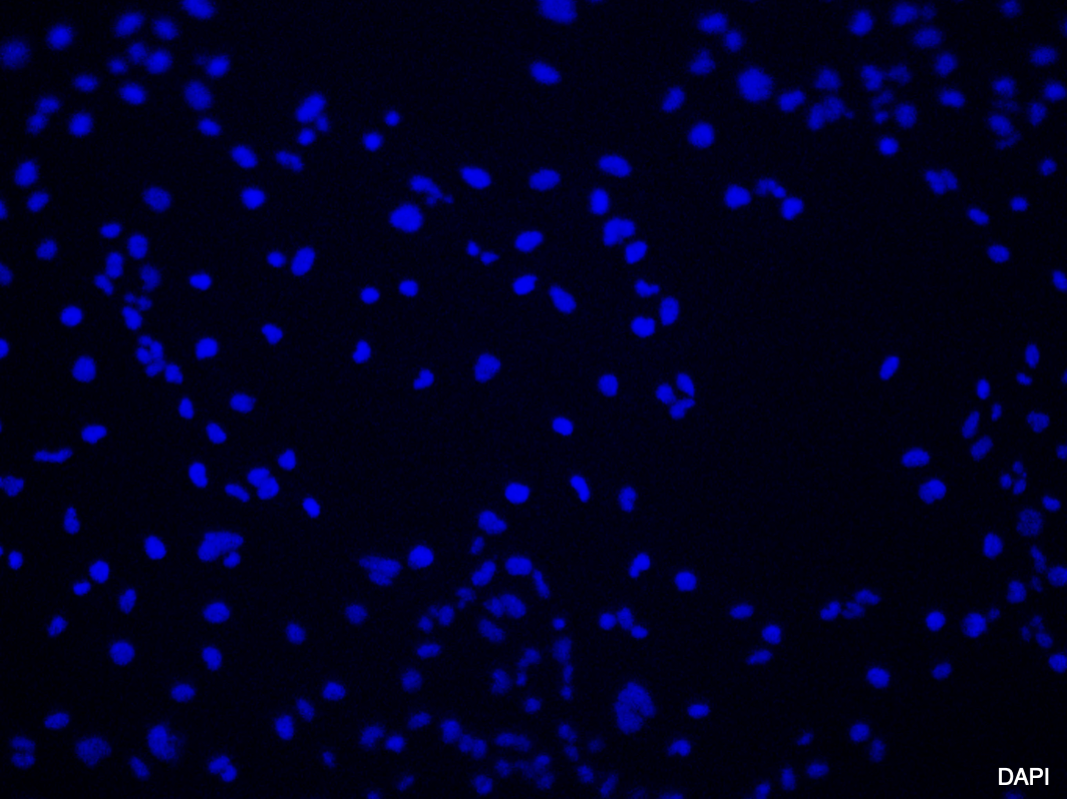

Supplement: Supplementary file 7 — Source Data Fig. 4 [file 44319_2023_33_MOESM7_ESM.zip › Fig.4/Fig. 4H/shScr/DAPI.tiff]

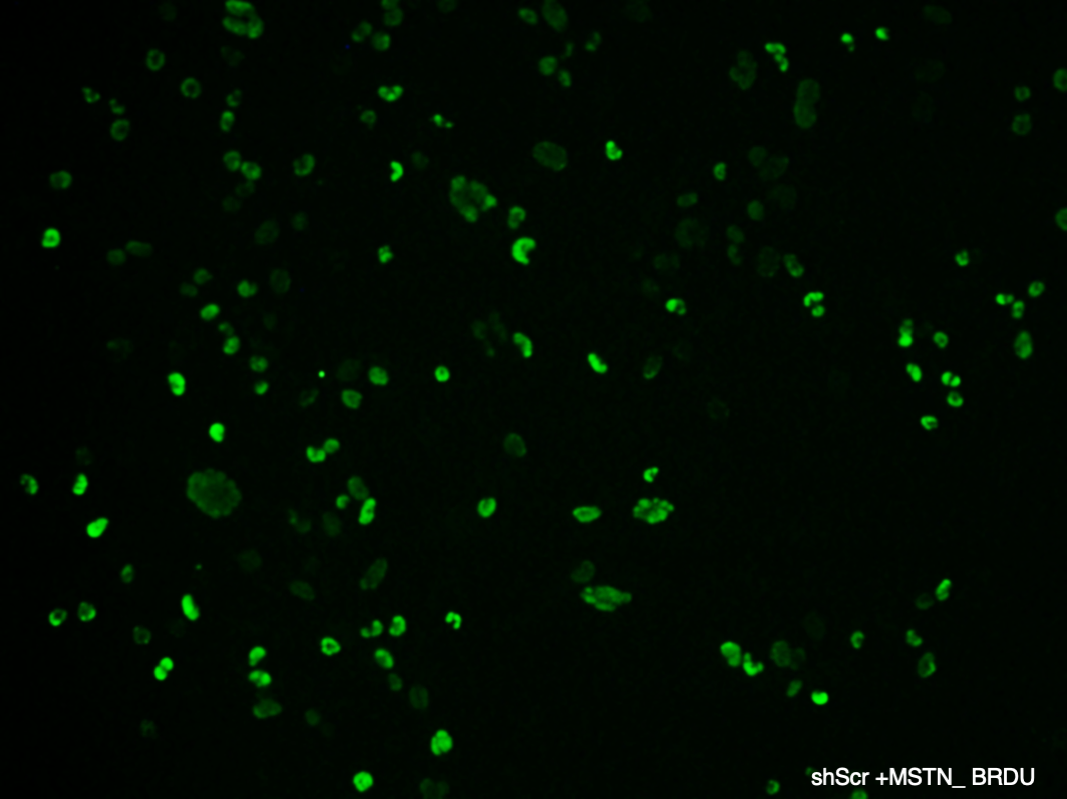

Supplement: Supplementary file 7 — Source Data Fig. 4 [file 44319_2023_33_MOESM7_ESM.zip › Fig.4/Fig. 4H/shScr/shScr+MSTN_BRDU.tiff]

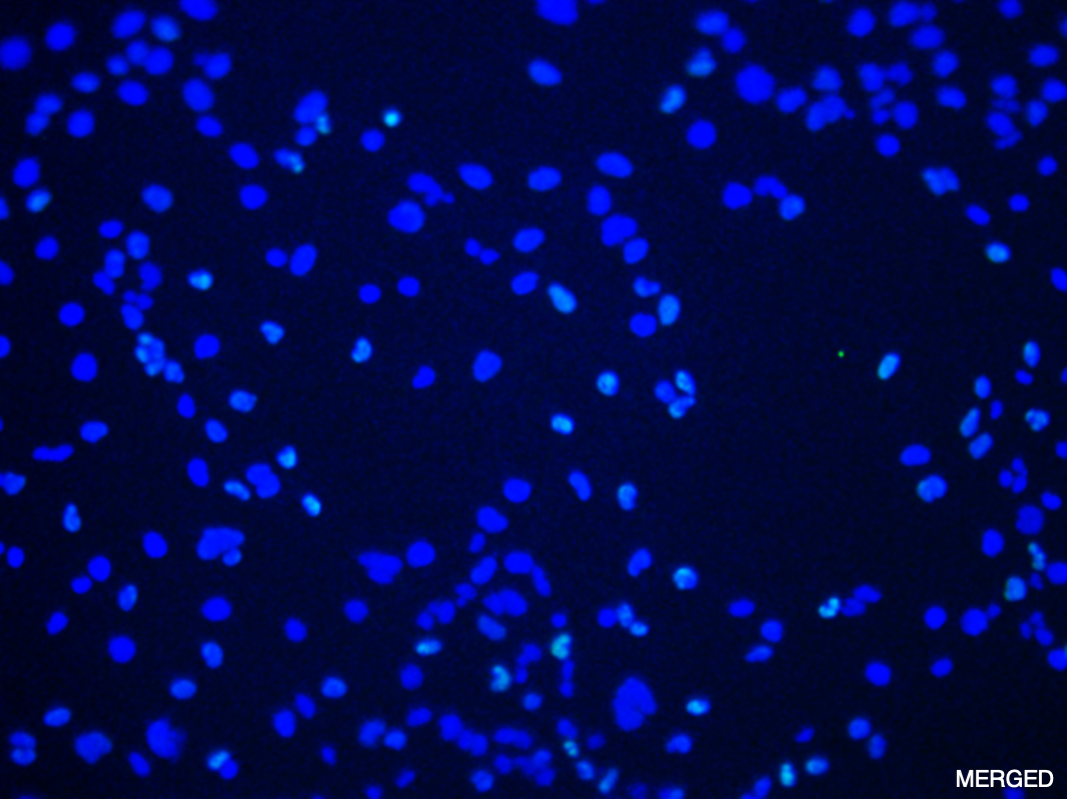

Supplement: Supplementary file 7 — Source Data Fig. 4 [file 44319_2023_33_MOESM7_ESM.zip › Fig.4/Fig. 4H/shScr/MERGED.tiff]

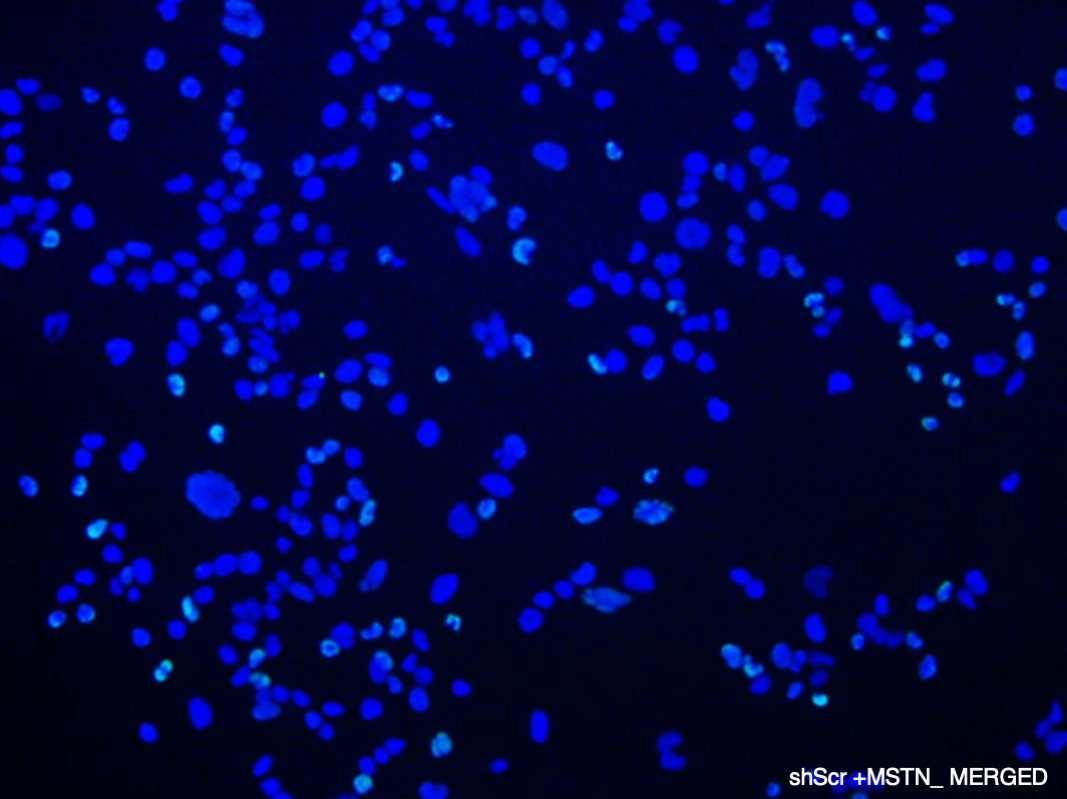

Supplement: Supplementary file 7 — Source Data Fig. 4 [file 44319_2023_33_MOESM7_ESM.zip › Fig.4/Fig. 4H/shScr/shScr+MSTN_MERGED.tiff]

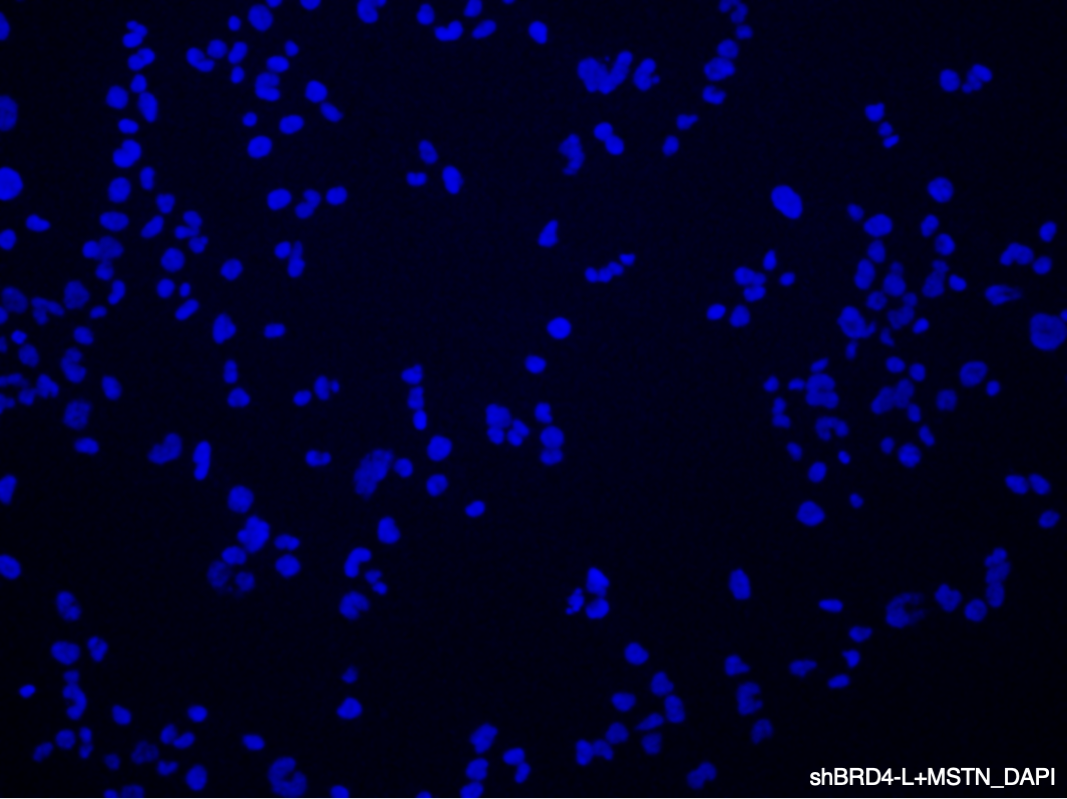

Supplement: Supplementary file 7 — Source Data Fig. 4 [file 44319_2023_33_MOESM7_ESM.zip › Fig.4/Fig. 4H/shBRD4-L/shScr+MSTN_DAPI.tiff]

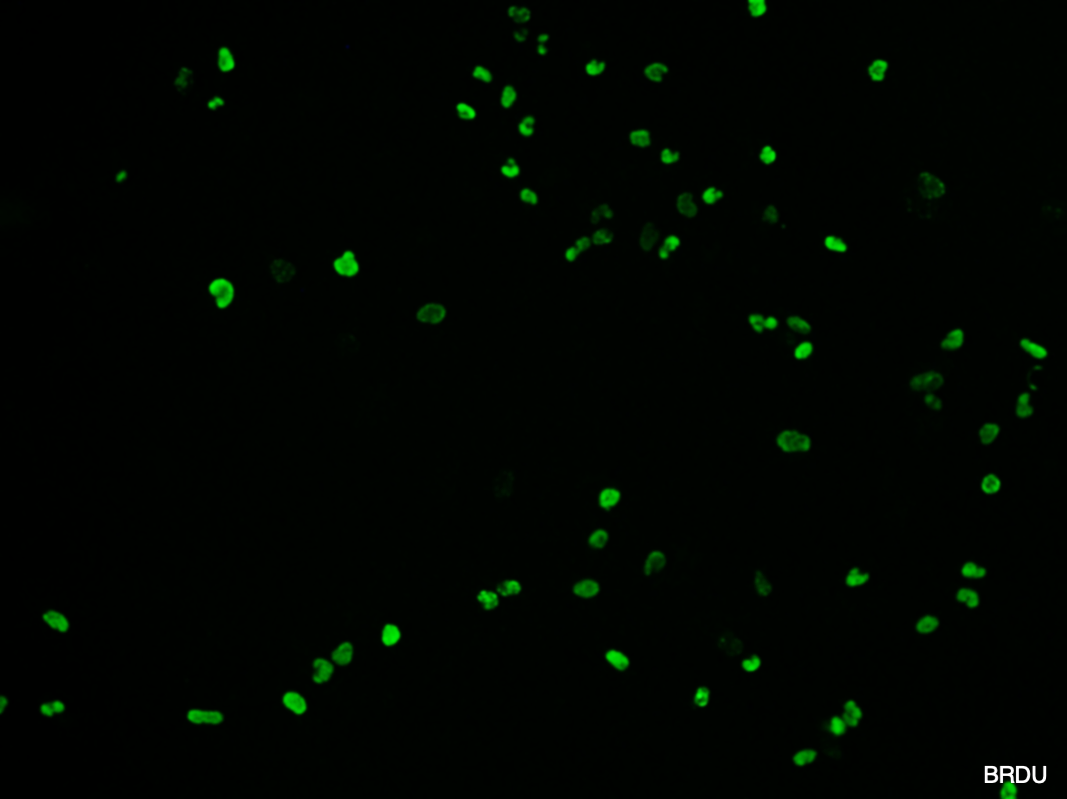

Supplement: Supplementary file 7 — Source Data Fig. 4 [file 44319_2023_33_MOESM7_ESM.zip › Fig.4/Fig. 4H/shBRD4-L/BRDU.tiff]

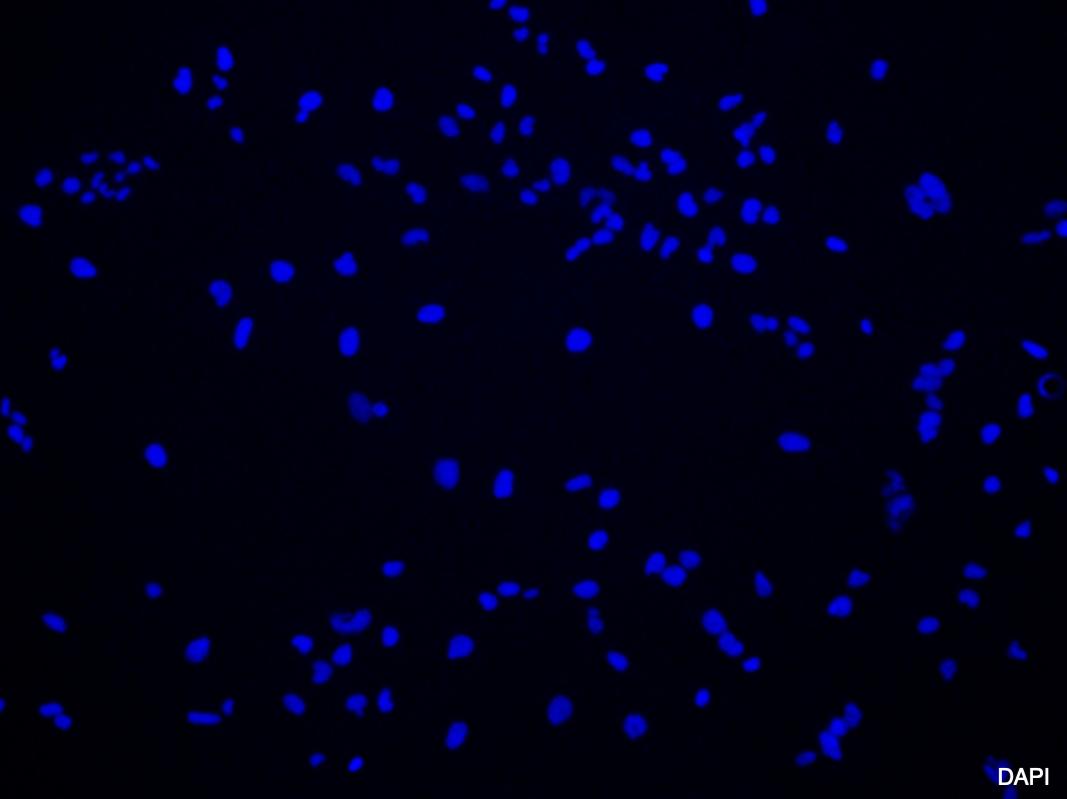

Supplement: Supplementary file 7 — Source Data Fig. 4 [file 44319_2023_33_MOESM7_ESM.zip › Fig.4/Fig. 4H/shBRD4-L/DAPI.tiff]

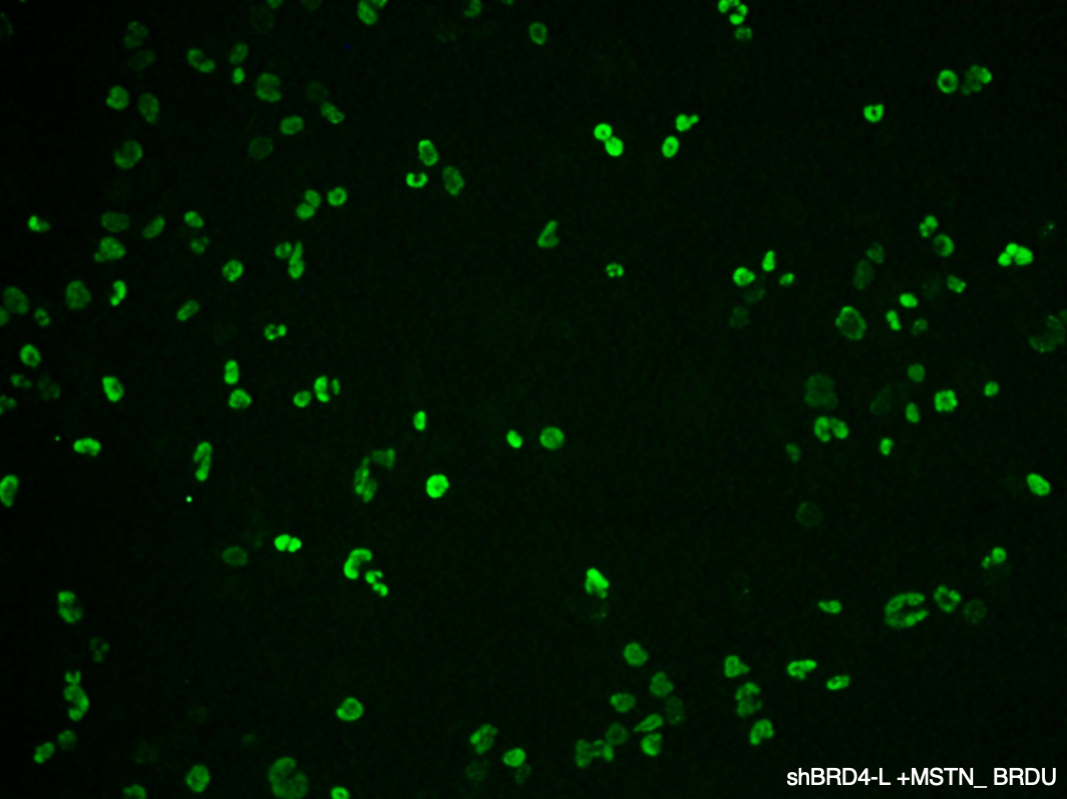

Supplement: Supplementary file 7 — Source Data Fig. 4 [file 44319_2023_33_MOESM7_ESM.zip › Fig.4/Fig. 4H/shBRD4-L/shScr+MSTN_BRDU.tiff]

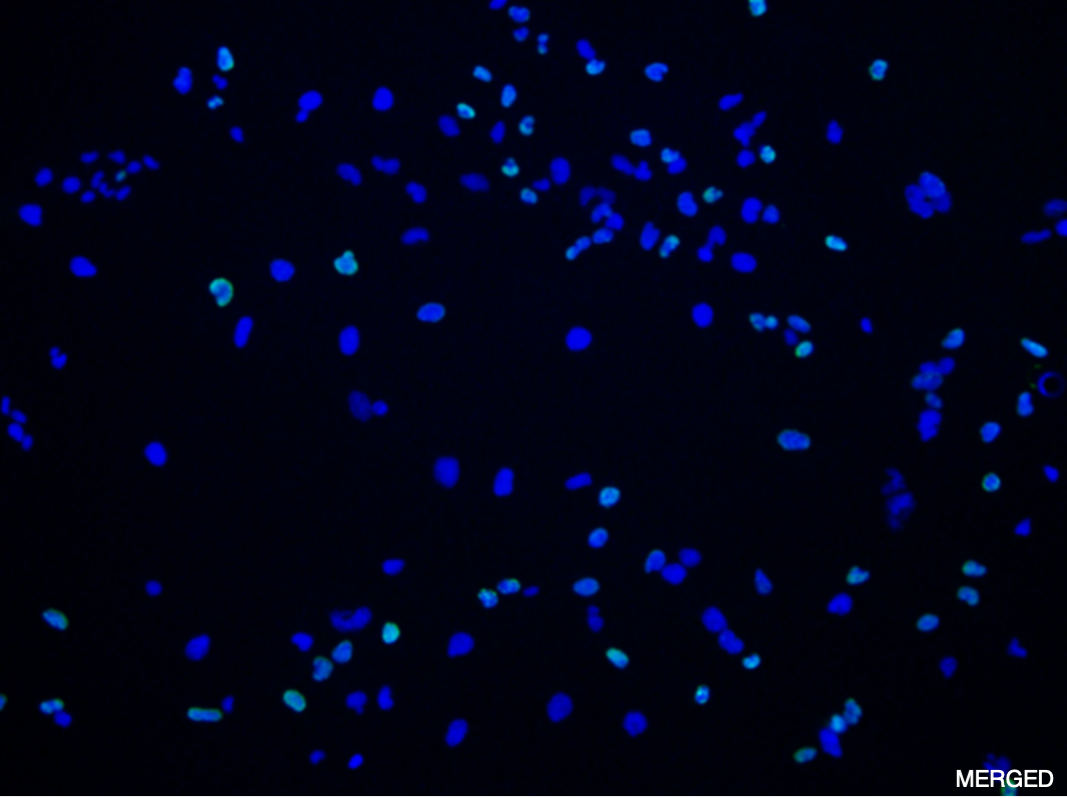

Supplement: Supplementary file 7 — Source Data Fig. 4 [file 44319_2023_33_MOESM7_ESM.zip › Fig.4/Fig. 4H/shBRD4-L/MERGED.tiff]

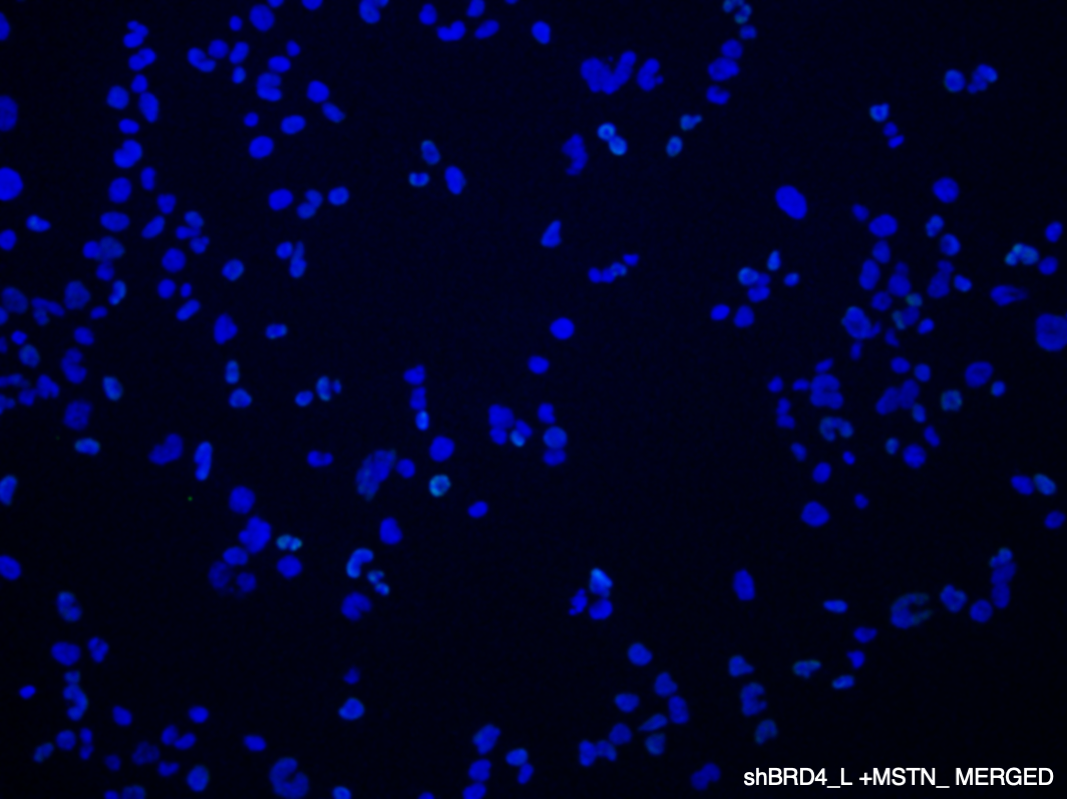

Supplement: Supplementary file 7 — Source Data Fig. 4 [file 44319_2023_33_MOESM7_ESM.zip › Fig.4/Fig. 4H/shBRD4-L/shScr+MSTN_MERGED.tiff]

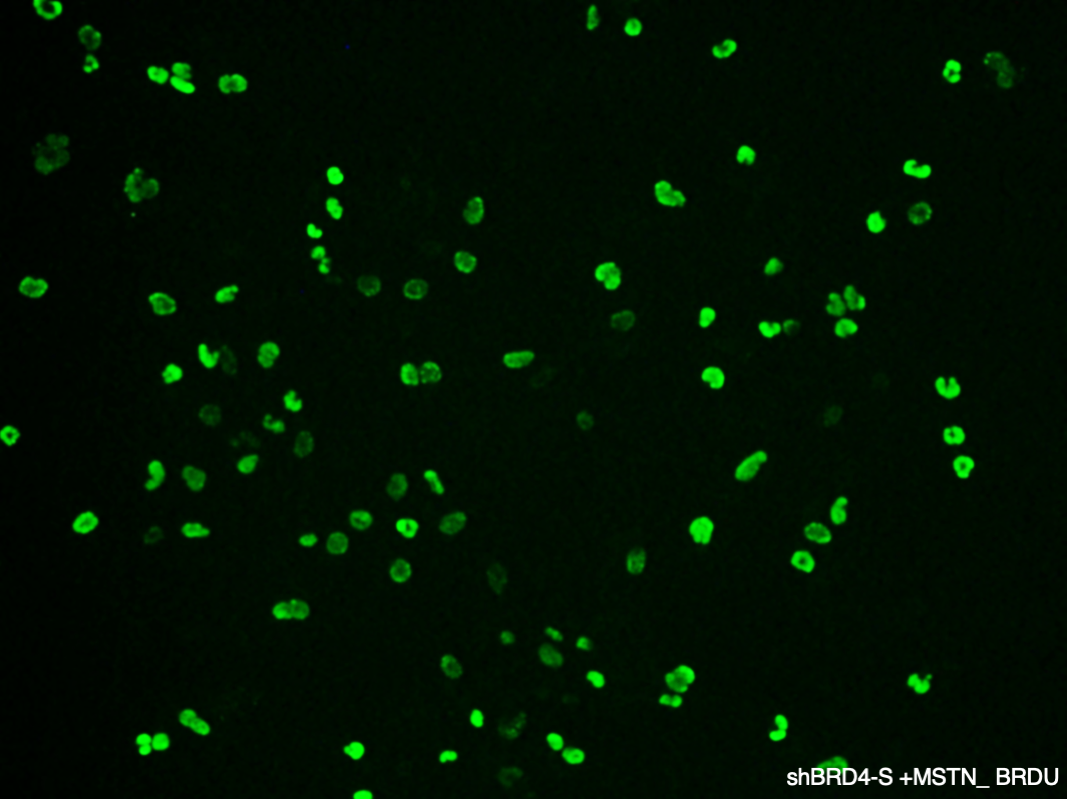

Supplement: Supplementary file 7 — Source Data Fig. 4 [file 44319_2023_33_MOESM7_ESM.zip › Fig.4/Fig. 4H/shBRD4-S/shBRD4-S+MSTN_BRDU.tiff]

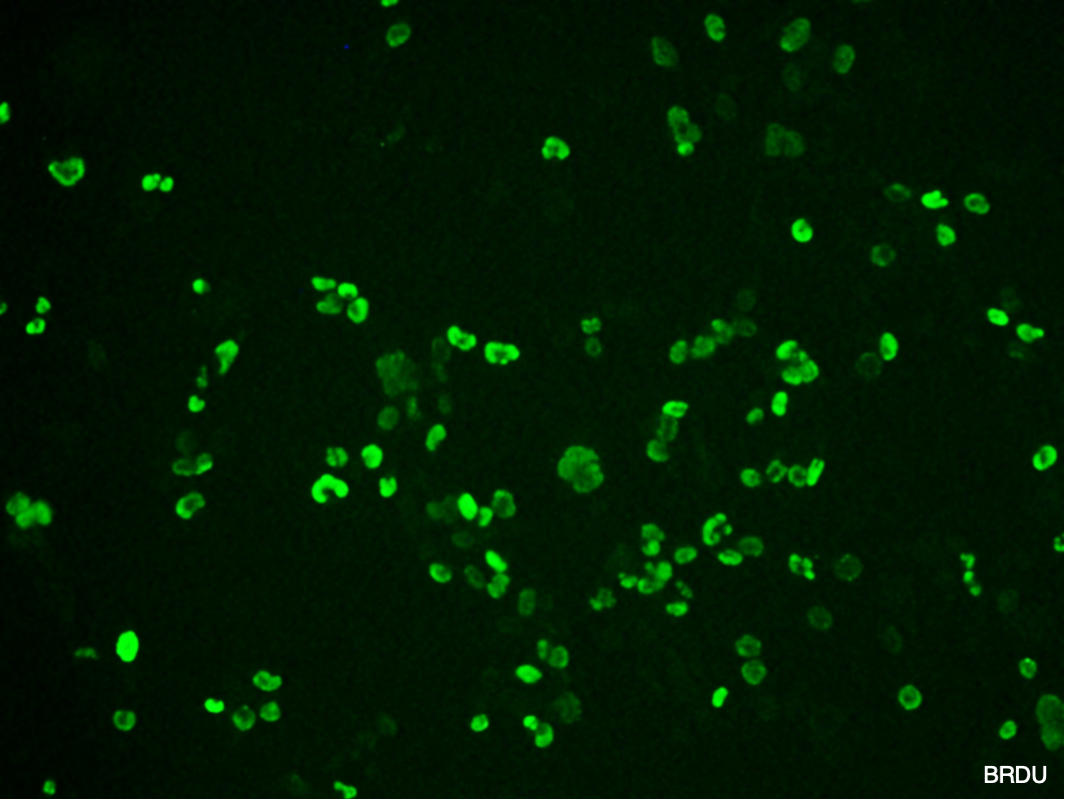

Supplement: Supplementary file 7 — Source Data Fig. 4 [file 44319_2023_33_MOESM7_ESM.zip › Fig.4/Fig. 4H/shBRD4-S/BRDU.tiff]

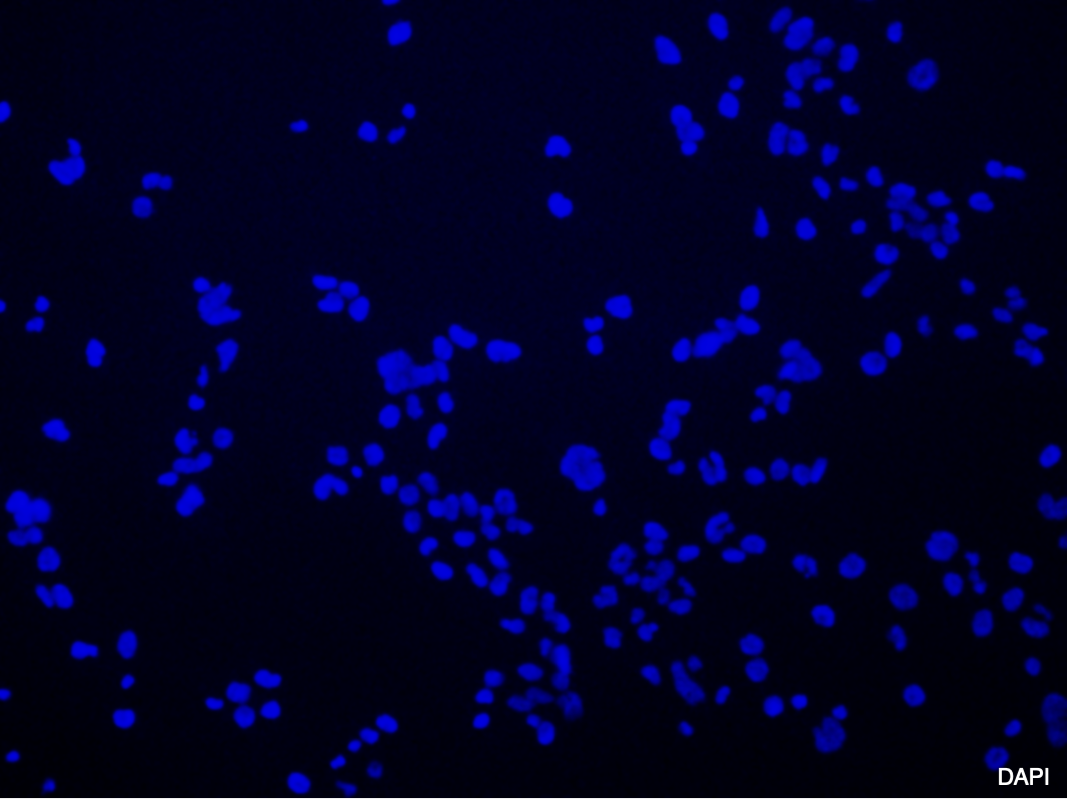

Supplement: Supplementary file 7 — Source Data Fig. 4 [file 44319_2023_33_MOESM7_ESM.zip › Fig.4/Fig. 4H/shBRD4-S/DAPI.tiff]

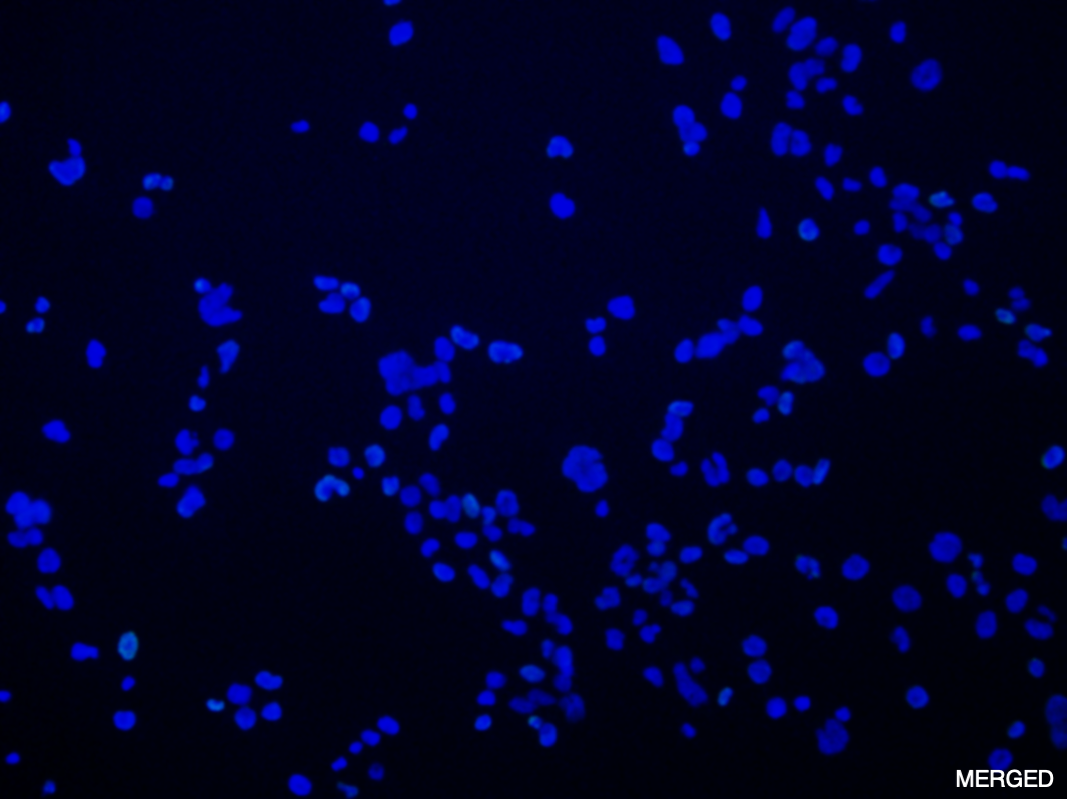

Supplement: Supplementary file 7 — Source Data Fig. 4 [file 44319_2023_33_MOESM7_ESM.zip › Fig.4/Fig. 4H/shBRD4-S/MERGED.tiff]

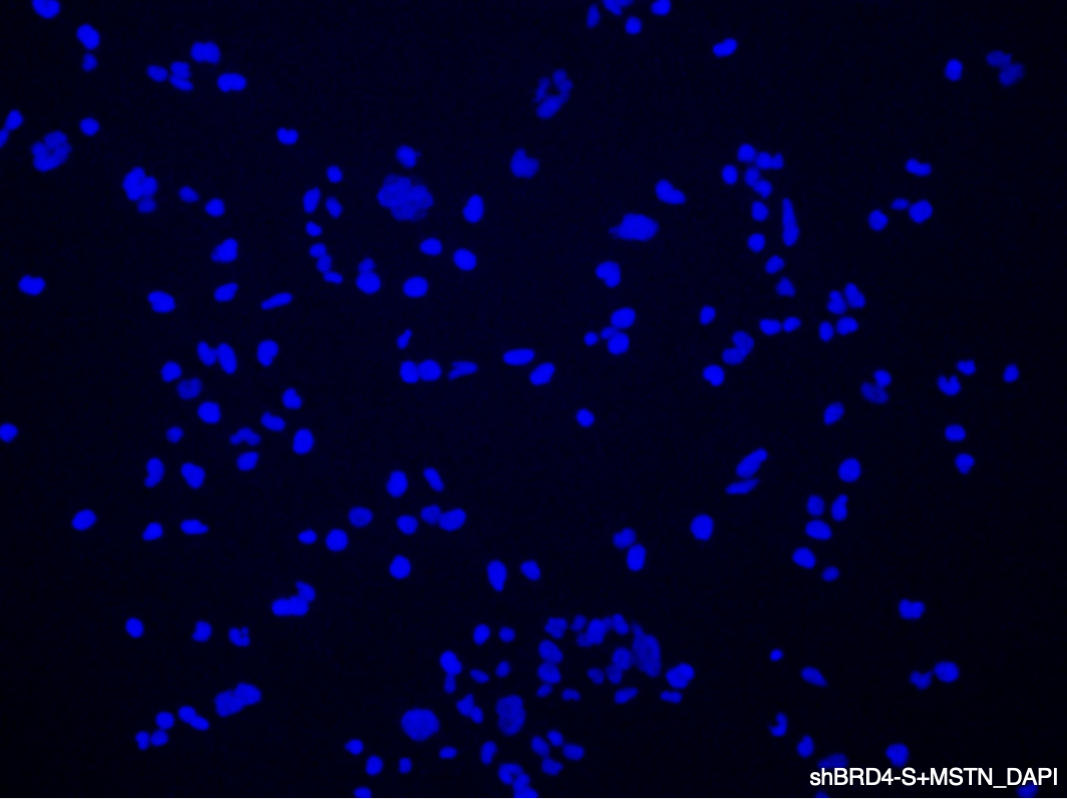

Supplement: Supplementary file 7 — Source Data Fig. 4 [file 44319_2023_33_MOESM7_ESM.zip › Fig.4/Fig. 4H/shBRD4-S/shBRD4-S+MSTN_DAPI.tiff]

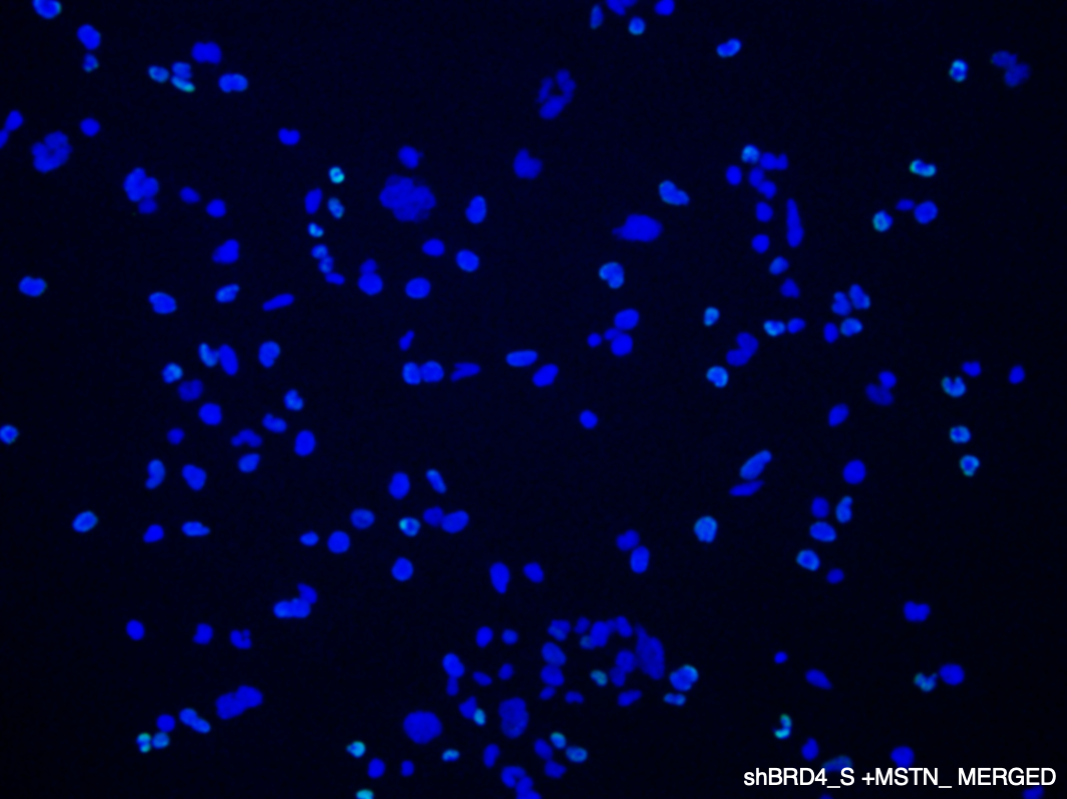

Supplement: Supplementary file 7 — Source Data Fig. 4 [file 44319_2023_33_MOESM7_ESM.zip › Fig.4/Fig. 4H/shBRD4-S/shBRD4-S+MSTN_MERGED.tiff]

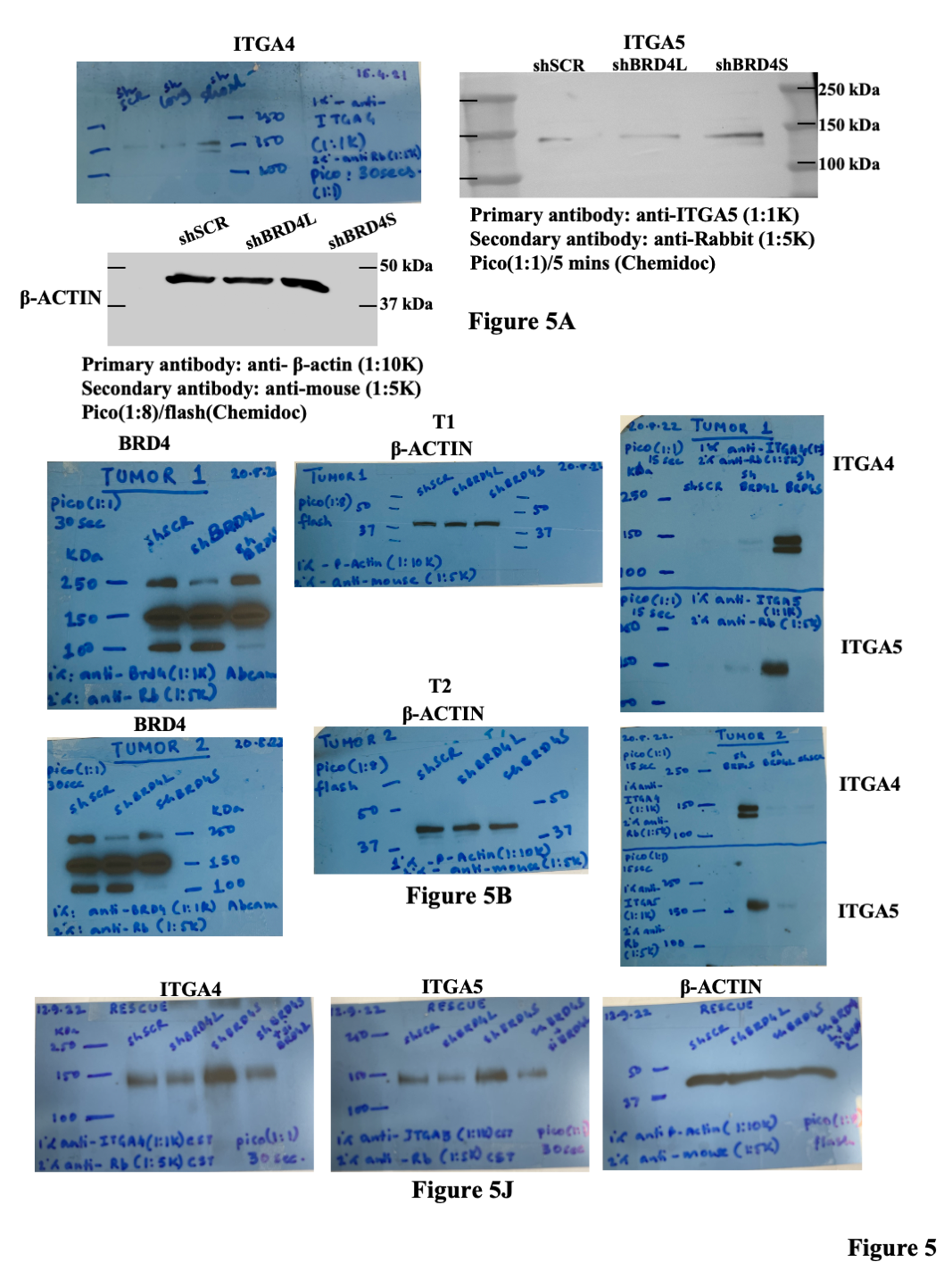

Supplement: Supplementary file 8 — Source Data Fig. 5 [file 44319_2023_33_MOESM8_ESM.zip › Fig.5/Fig. 5ABJ_Western.tiff]

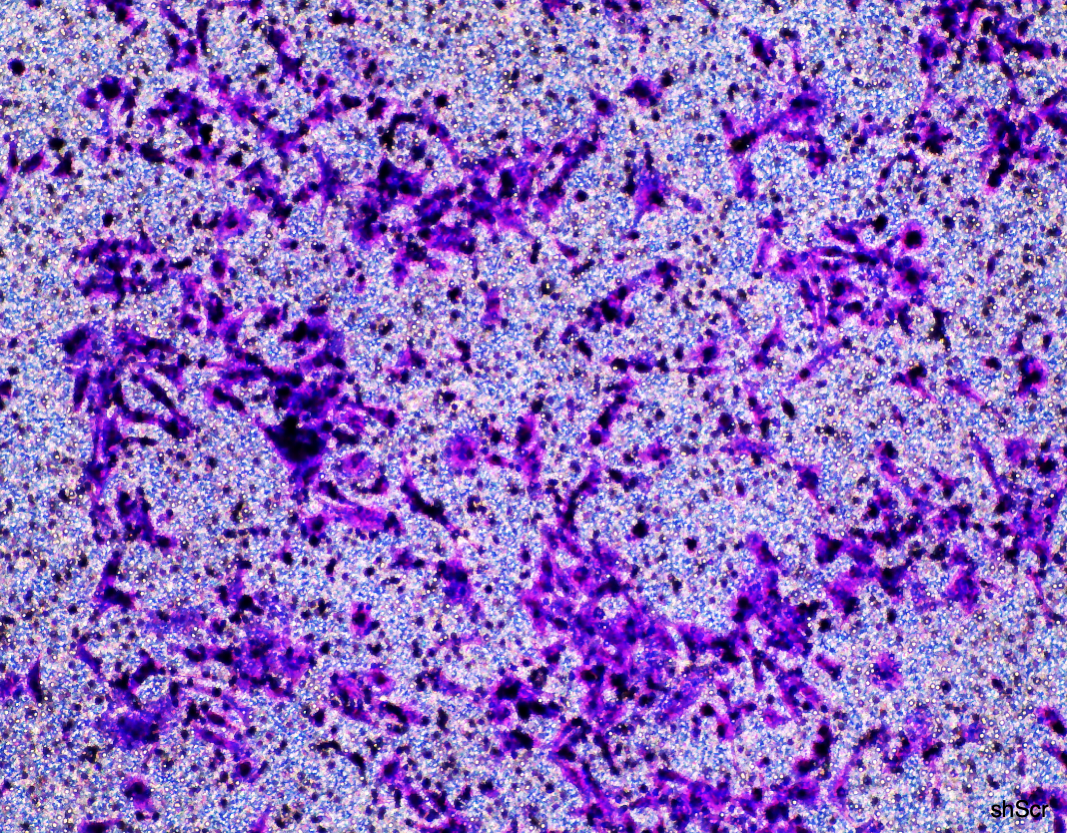

Supplement: Supplementary file 8 — Source Data Fig. 5 [file 44319_2023_33_MOESM8_ESM.zip › Fig.5/Fig. 5I/INVASION/shScr.tiff]

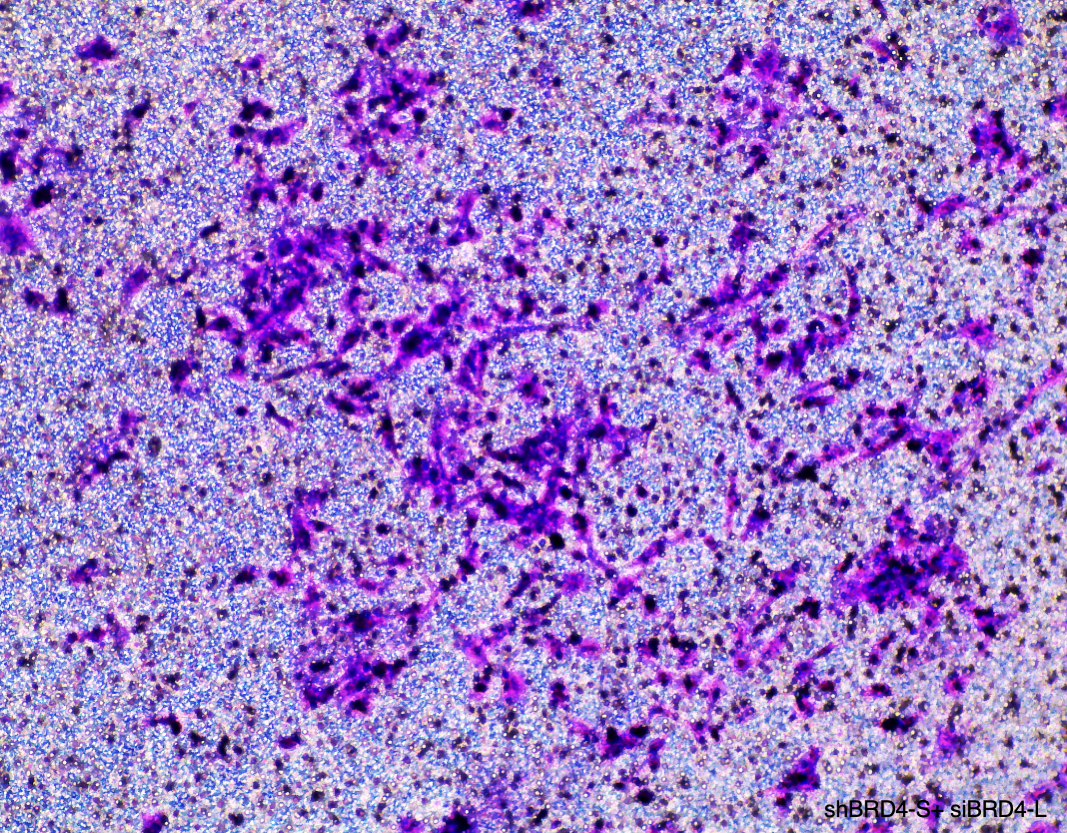

Supplement: Supplementary file 8 — Source Data Fig. 5 [file 44319_2023_33_MOESM8_ESM.zip › Fig.5/Fig. 5I/INVASION/shBRD4-S+siBRD4-L.tiff]

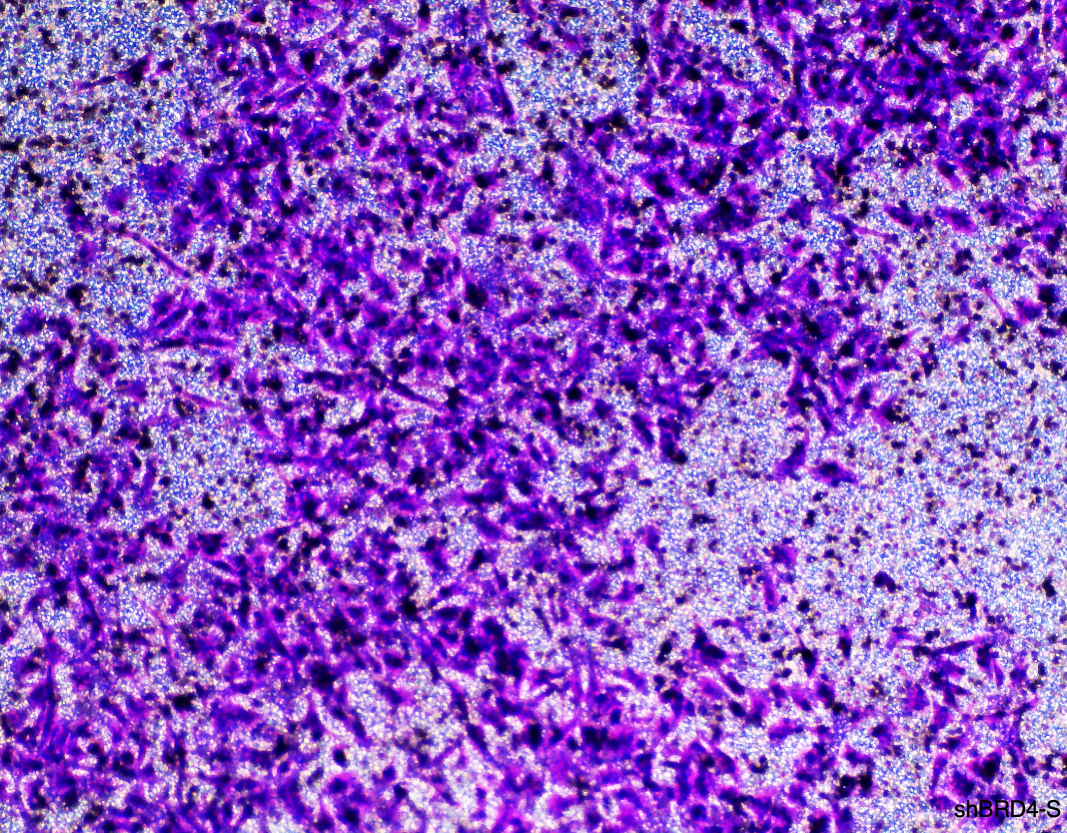

Supplement: Supplementary file 8 — Source Data Fig. 5 [file 44319_2023_33_MOESM8_ESM.zip › Fig.5/Fig. 5I/INVASION/shBRD4-S.tiff]

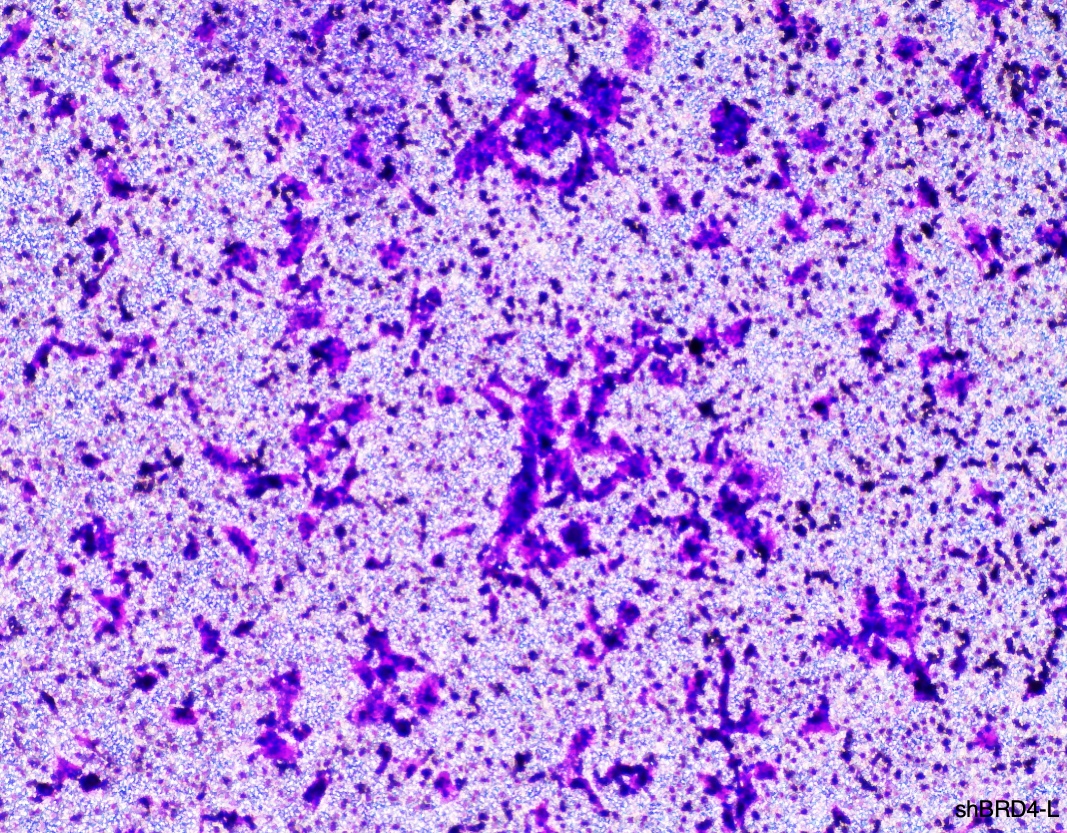

Supplement: Supplementary file 8 — Source Data Fig. 5 [file 44319_2023_33_MOESM8_ESM.zip › Fig.5/Fig. 5I/INVASION/shBRD4-L.tiff]

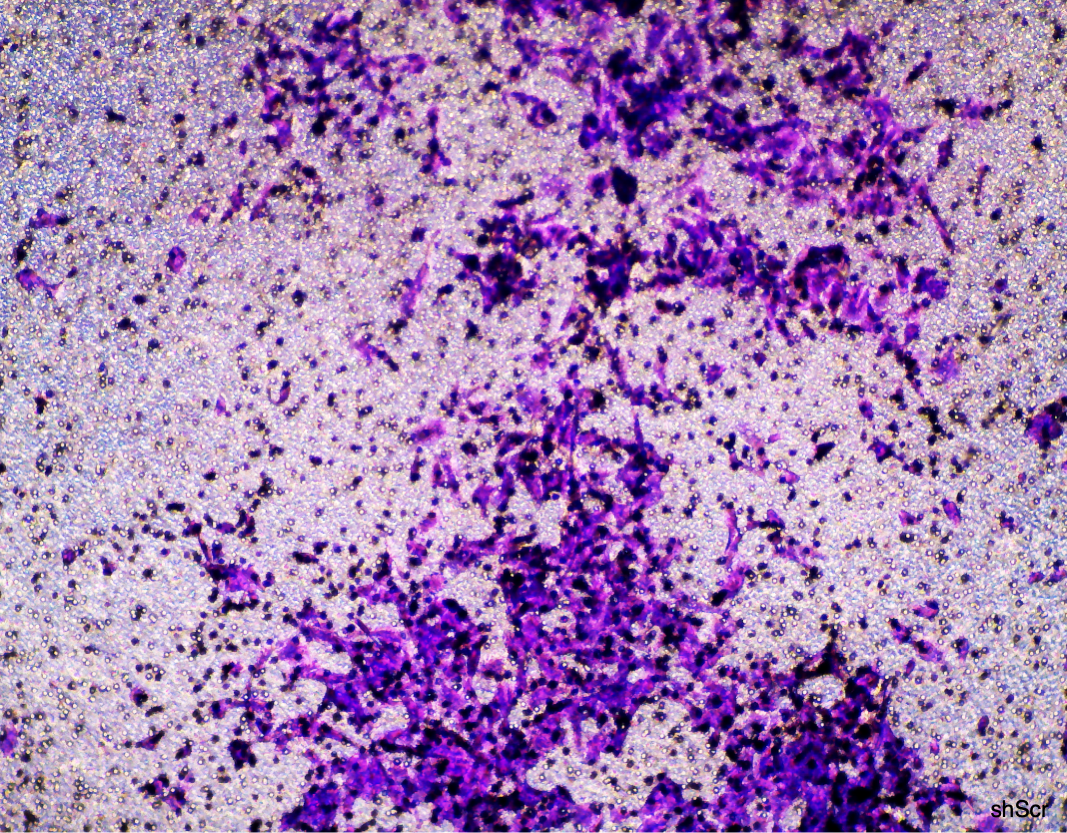

Supplement: Supplementary file 8 — Source Data Fig. 5 [file 44319_2023_33_MOESM8_ESM.zip › Fig.5/Fig. 5I/MIGRATION/shScr.tiff]

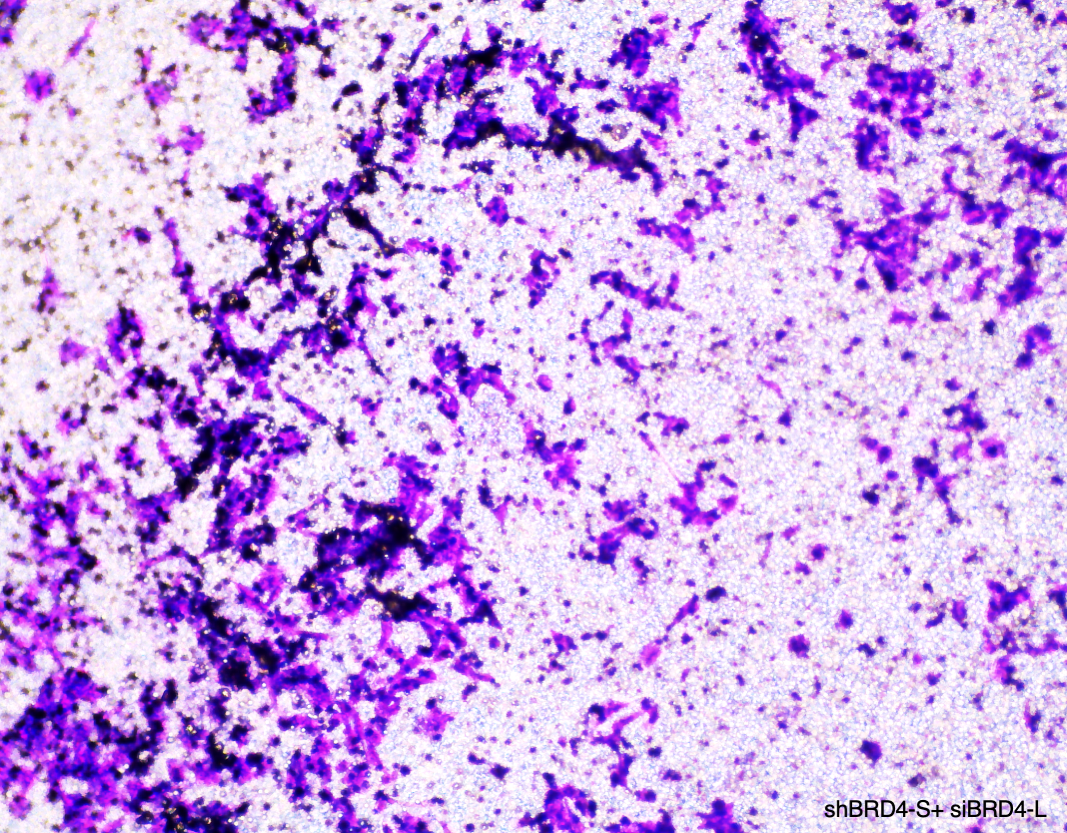

Supplement: Supplementary file 8 — Source Data Fig. 5 [file 44319_2023_33_MOESM8_ESM.zip › Fig.5/Fig. 5I/MIGRATION/shBRD4-S+siBRD4-L.tiff]

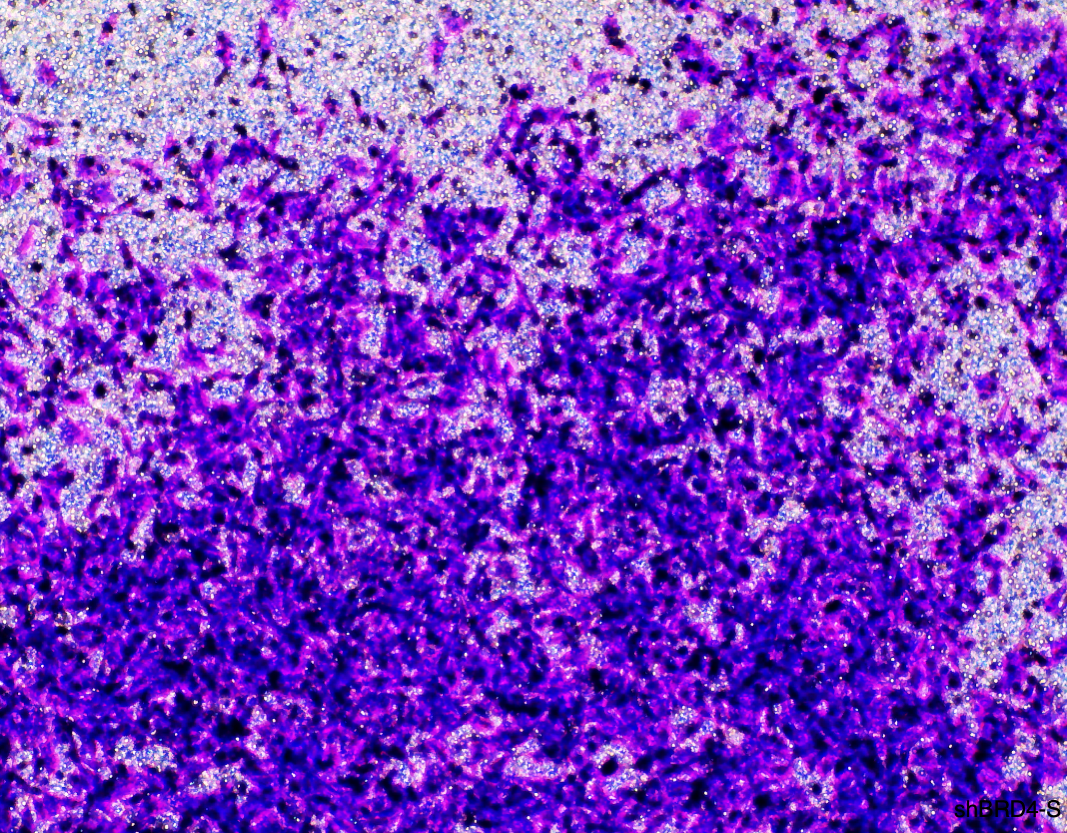

Supplement: Supplementary file 8 — Source Data Fig. 5 [file 44319_2023_33_MOESM8_ESM.zip › Fig.5/Fig. 5I/MIGRATION/shBRD4-S.tiff]

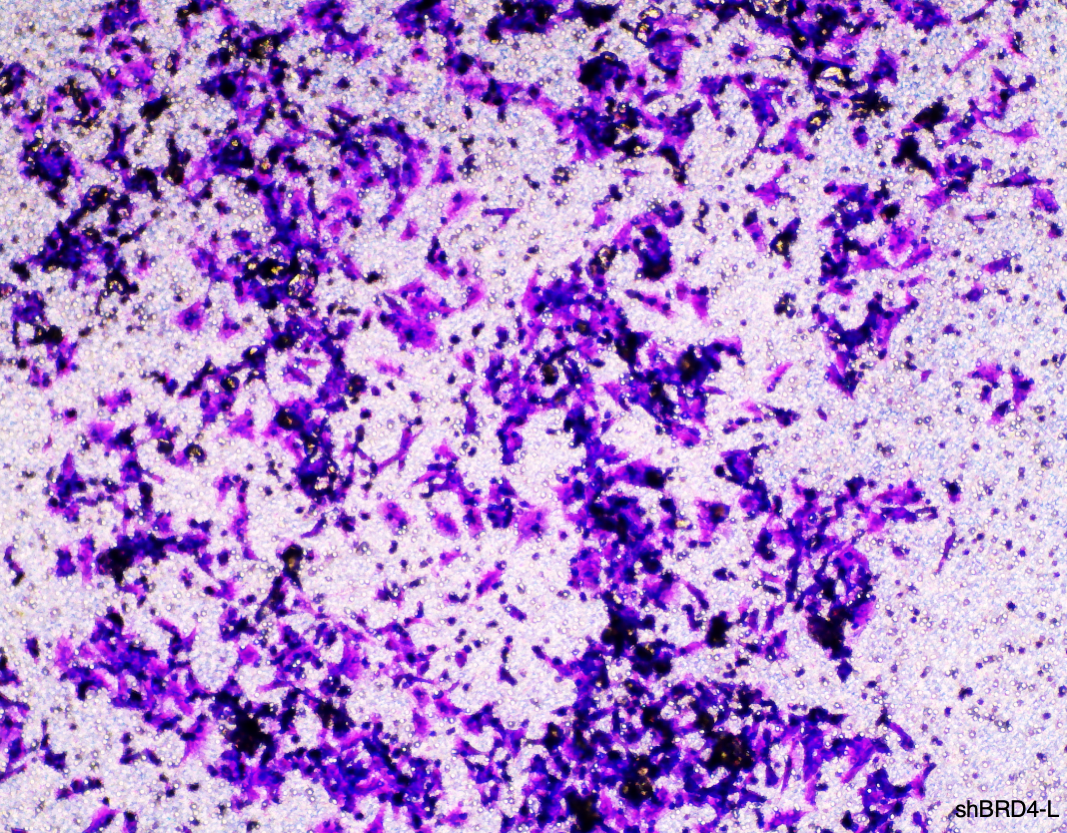

Supplement: Supplementary file 8 — Source Data Fig. 5 [file 44319_2023_33_MOESM8_ESM.zip › Fig.5/Fig. 5I/MIGRATION/shBRD4-L.tiff]

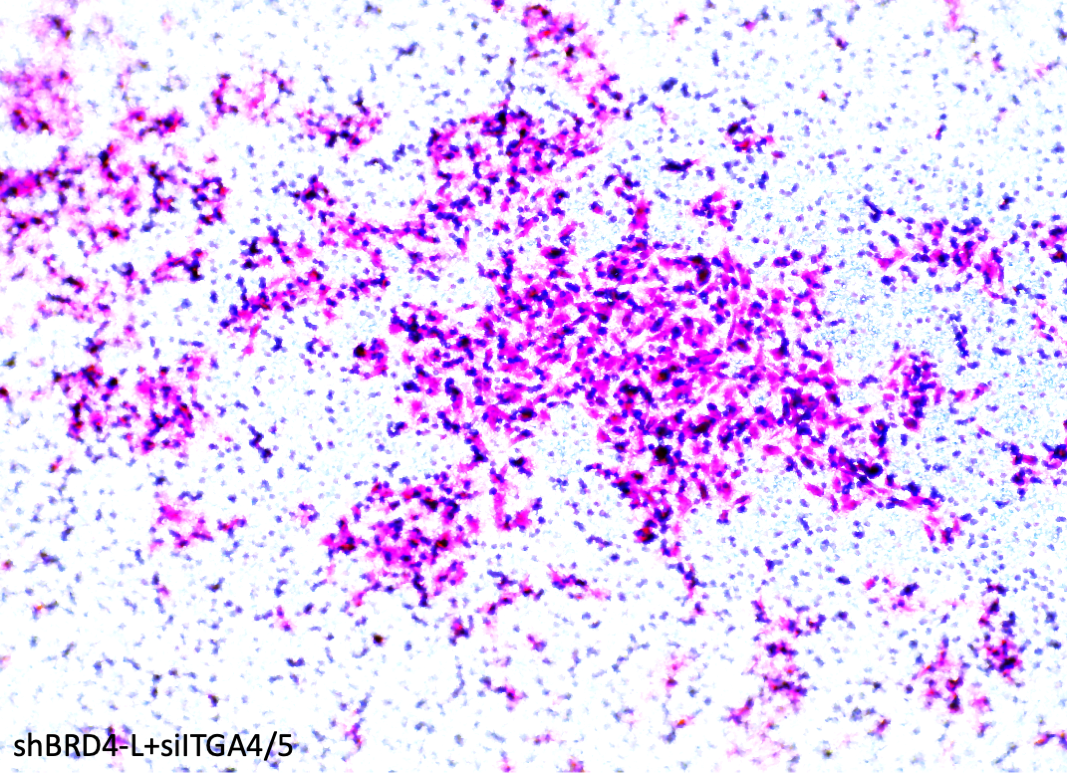

Supplement: Supplementary file 9 — Source Data Fig. EV5 [file 44319_2023_33_MOESM9_ESM.zip › Fig EV5/EV5A_MIGRATION/shBRD4-L+siITGA4:5.tiff]

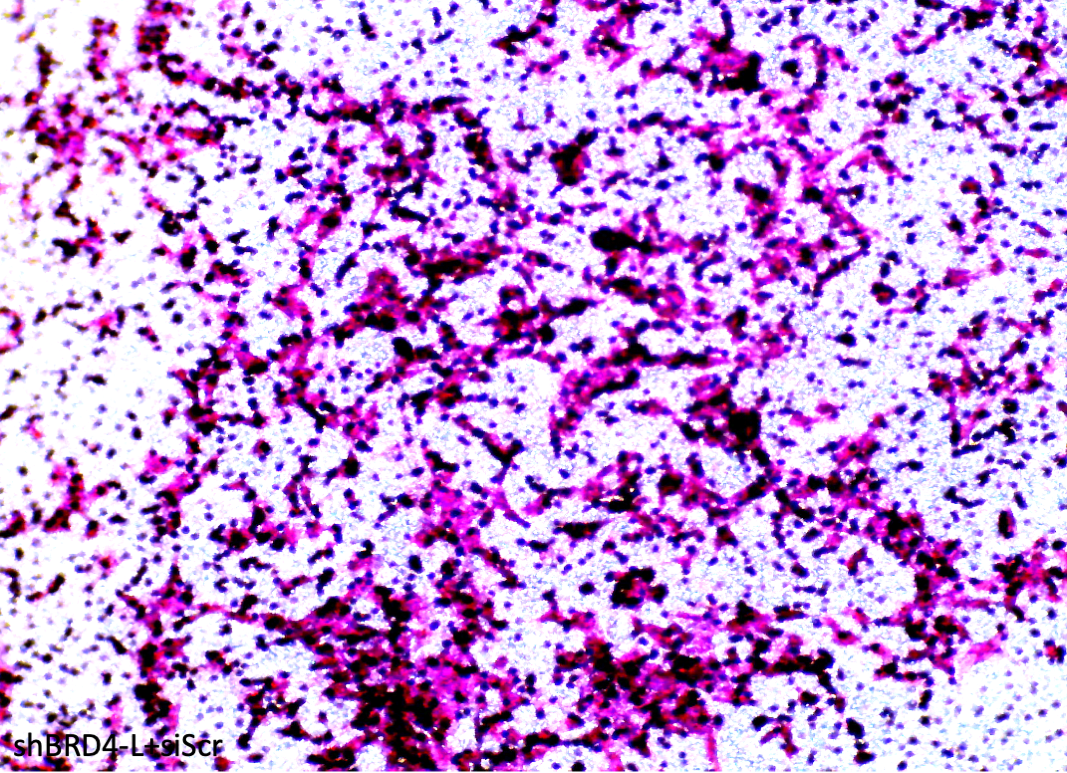

Supplement: Supplementary file 9 — Source Data Fig. EV5 [file 44319_2023_33_MOESM9_ESM.zip › Fig EV5/EV5A_MIGRATION/shBRD4-L+siScr.tiff]

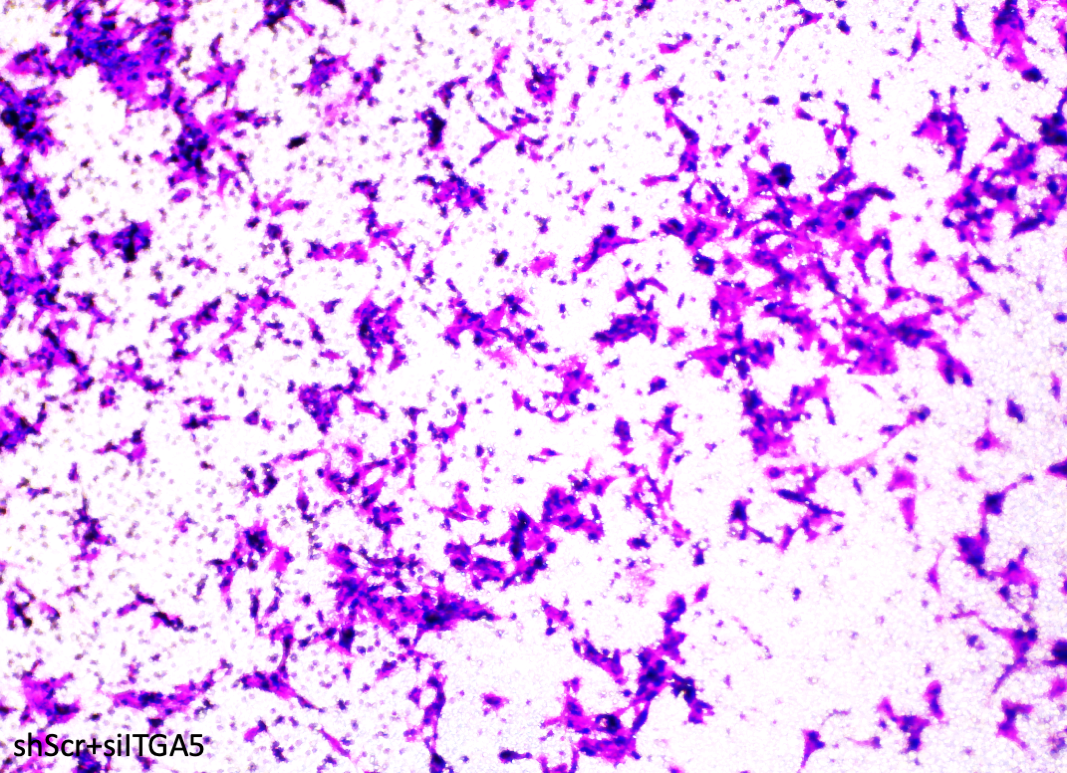

Supplement: Supplementary file 9 — Source Data Fig. EV5 [file 44319_2023_33_MOESM9_ESM.zip › Fig EV5/EV5A_MIGRATION/shScr+siITGA5.tiff]

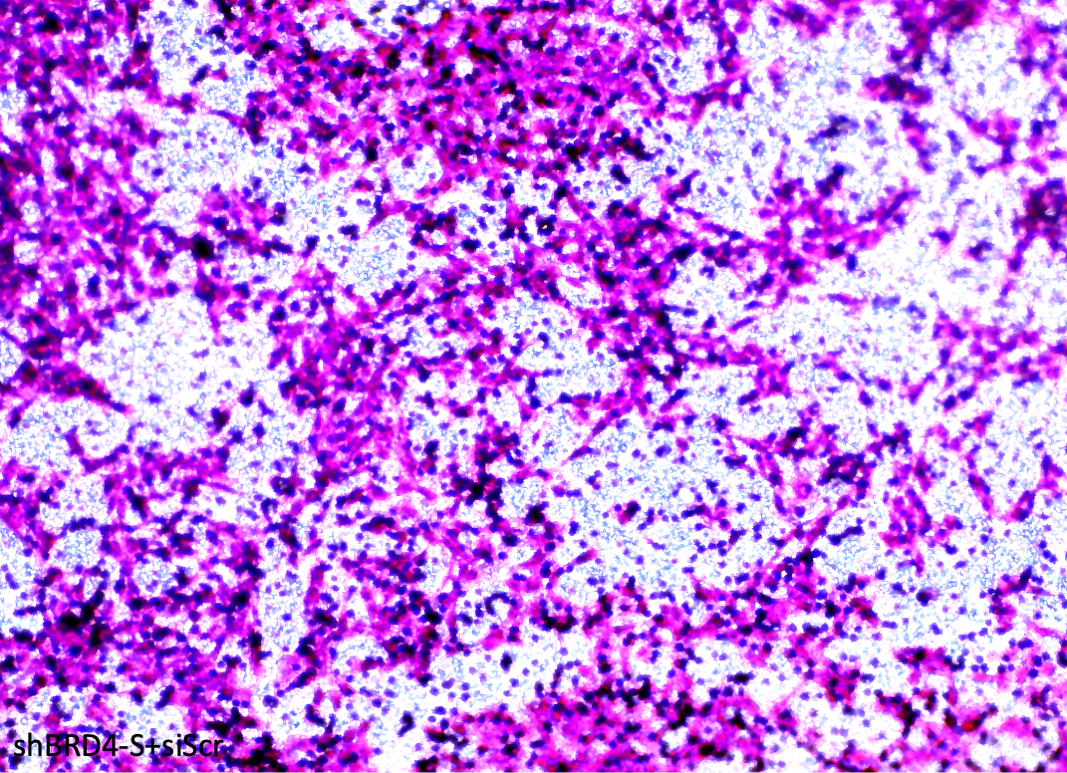

Supplement: Supplementary file 9 — Source Data Fig. EV5 [file 44319_2023_33_MOESM9_ESM.zip › Fig EV5/EV5A_MIGRATION/shBRD4-S+siScr.tiff]

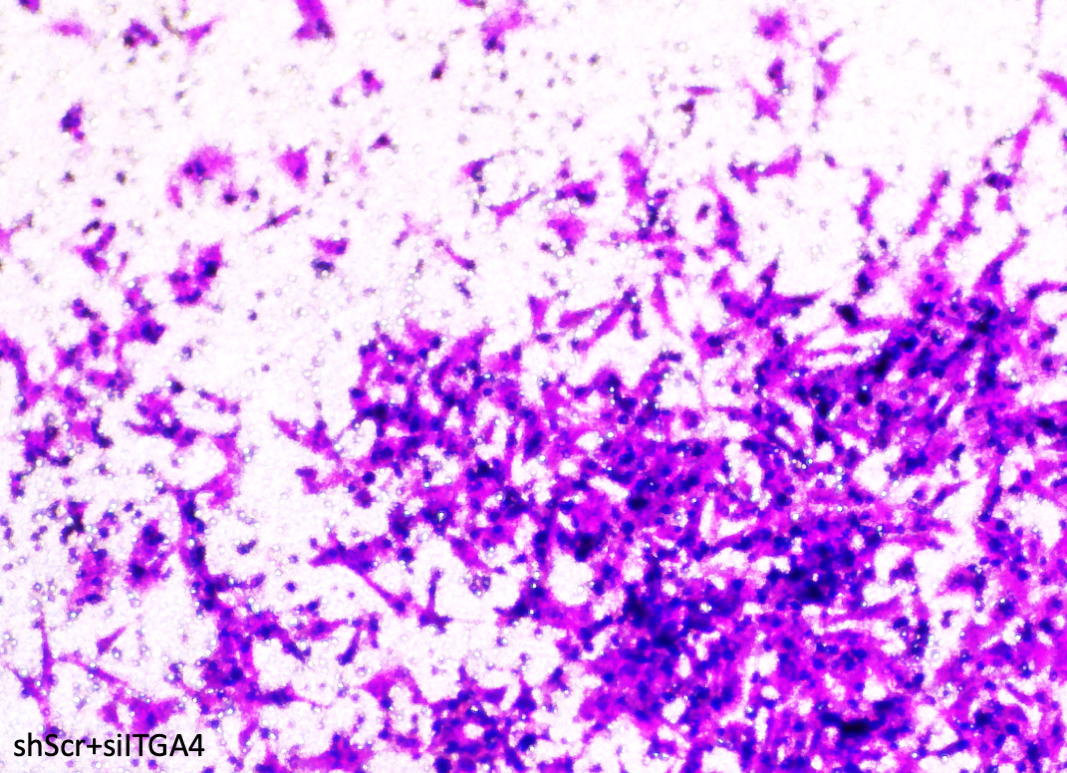

Supplement: Supplementary file 9 — Source Data Fig. EV5 [file 44319_2023_33_MOESM9_ESM.zip › Fig EV5/EV5A_MIGRATION/shScr+siITGA4.tiff]

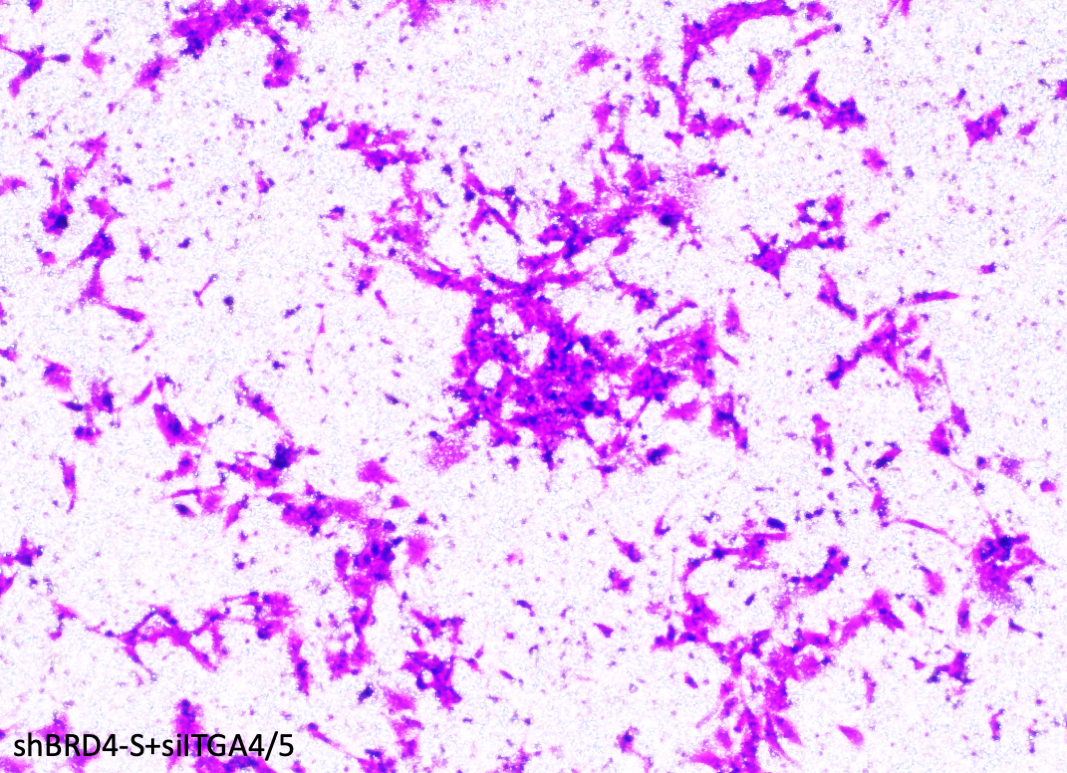

Supplement: Supplementary file 9 — Source Data Fig. EV5 [file 44319_2023_33_MOESM9_ESM.zip › Fig EV5/EV5A_MIGRATION/shBRD4-S+siITGA4:5.tiff]

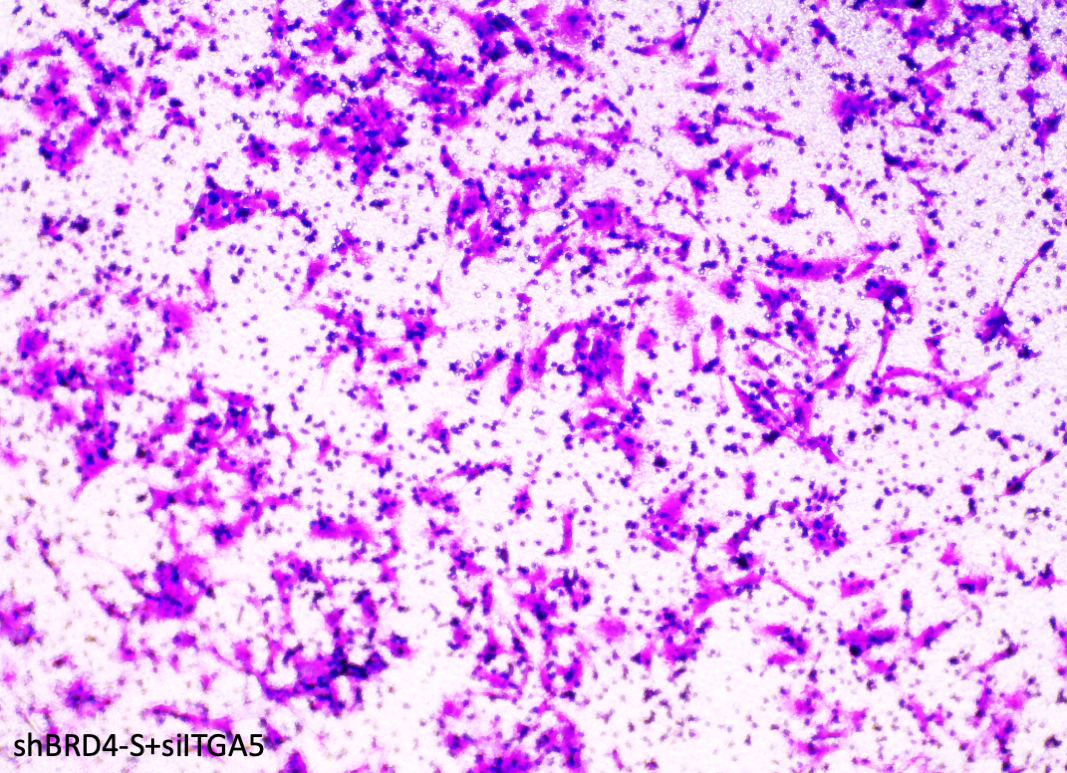

Supplement: Supplementary file 9 — Source Data Fig. EV5 [file 44319_2023_33_MOESM9_ESM.zip › Fig EV5/EV5A_MIGRATION/shBRD4-S+siITGA5.tiff]

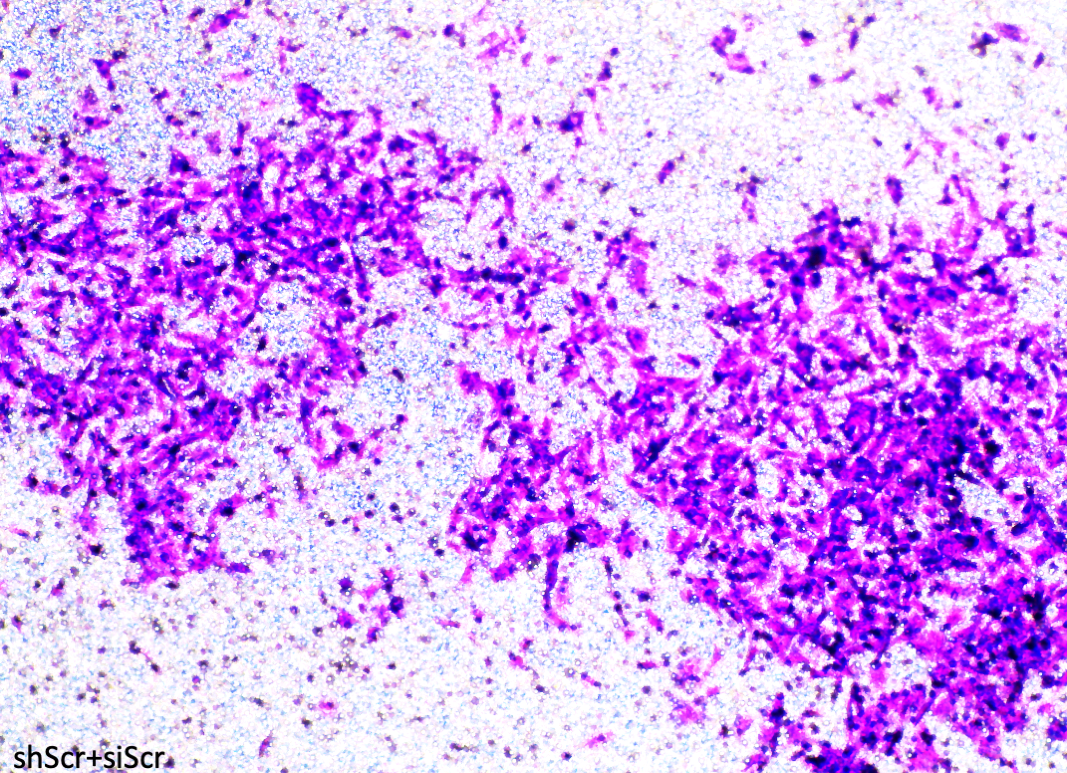

Supplement: Supplementary file 9 — Source Data Fig. EV5 [file 44319_2023_33_MOESM9_ESM.zip › Fig EV5/EV5A_MIGRATION/shScr+siScr.tiff]

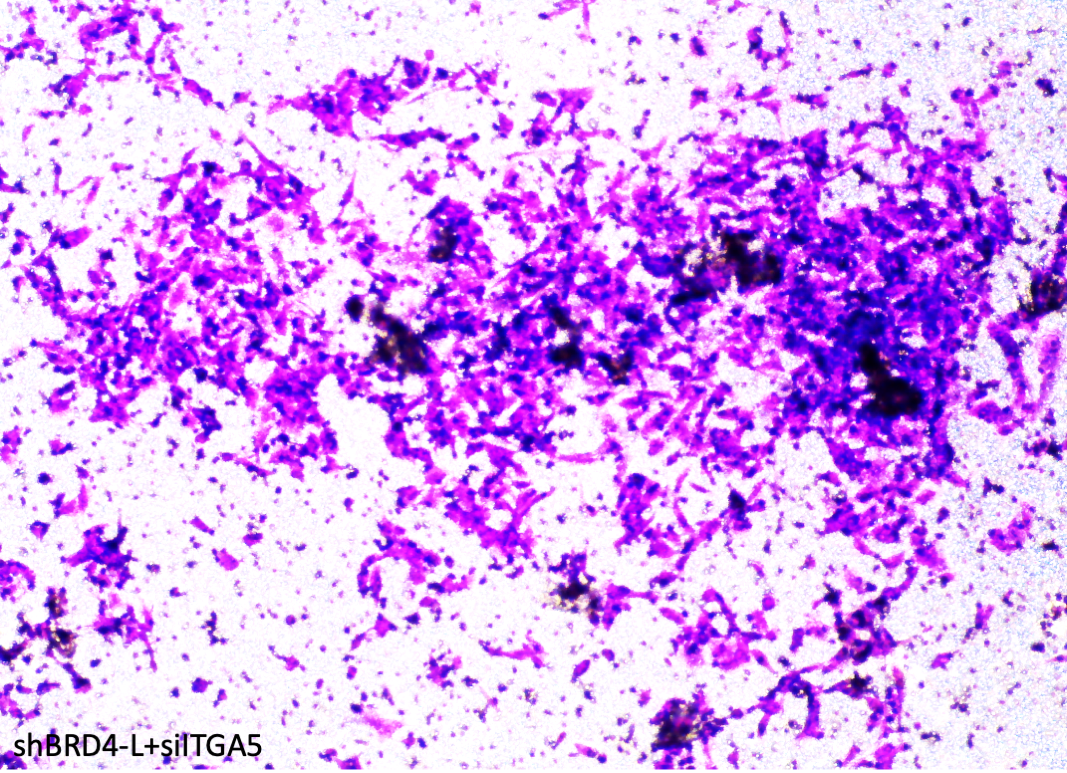

Supplement: Supplementary file 9 — Source Data Fig. EV5 [file 44319_2023_33_MOESM9_ESM.zip › Fig EV5/EV5A_MIGRATION/shBRD4-L+siITGA5.tiff]

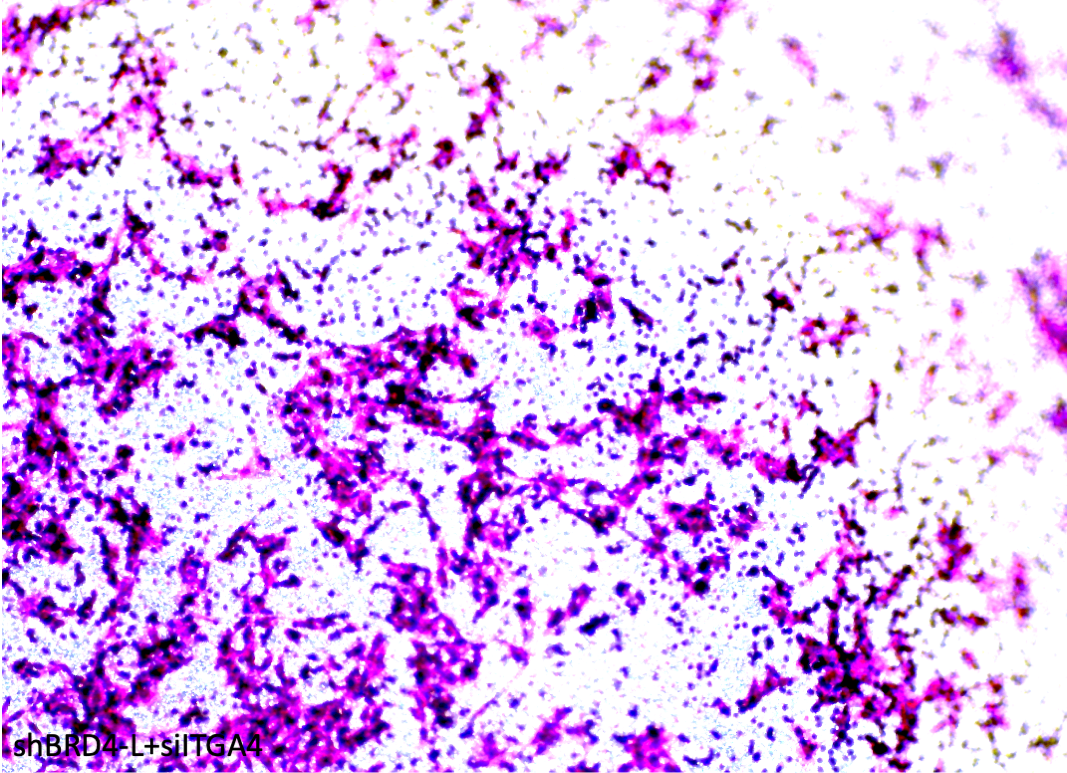

Supplement: Supplementary file 9 — Source Data Fig. EV5 [file 44319_2023_33_MOESM9_ESM.zip › Fig EV5/EV5A_MIGRATION/shBRD4-L+siITGA4.tiff]

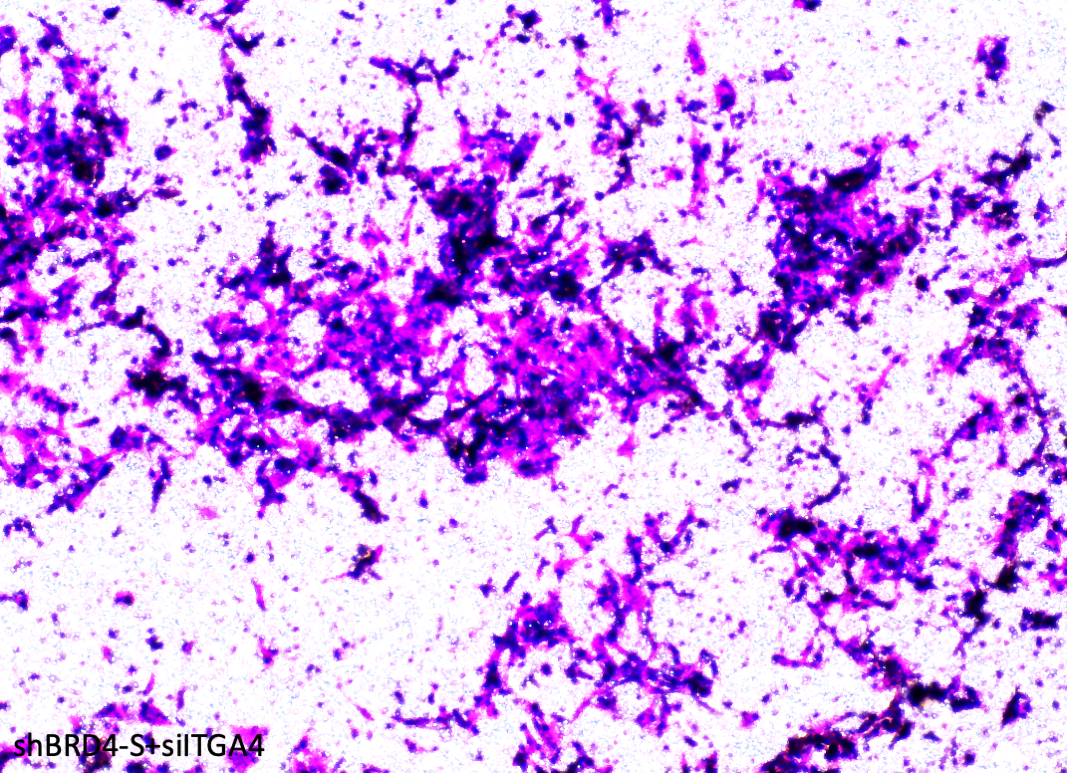

Supplement: Supplementary file 9 — Source Data Fig. EV5 [file 44319_2023_33_MOESM9_ESM.zip › Fig EV5/EV5A_MIGRATION/shBRD4-S+siITGA4.tiff]

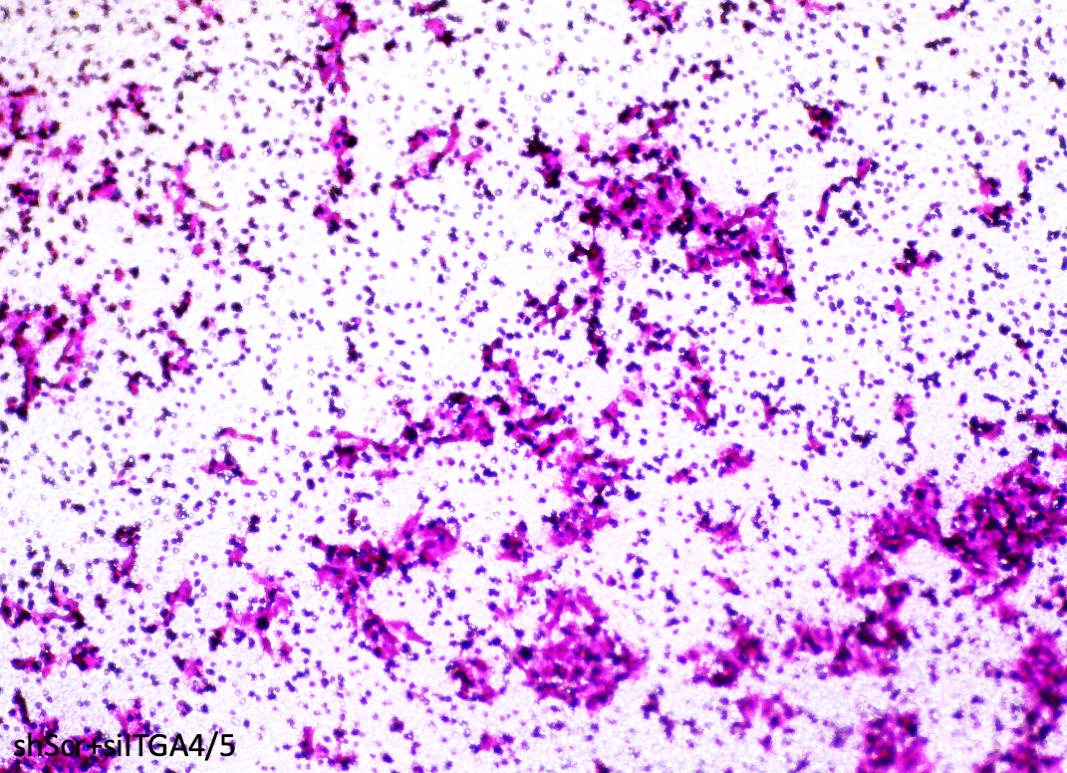

Supplement: Supplementary file 9 — Source Data Fig. EV5 [file 44319_2023_33_MOESM9_ESM.zip › Fig EV5/EV5A_MIGRATION/shScr+siITGA4:5.tiff]

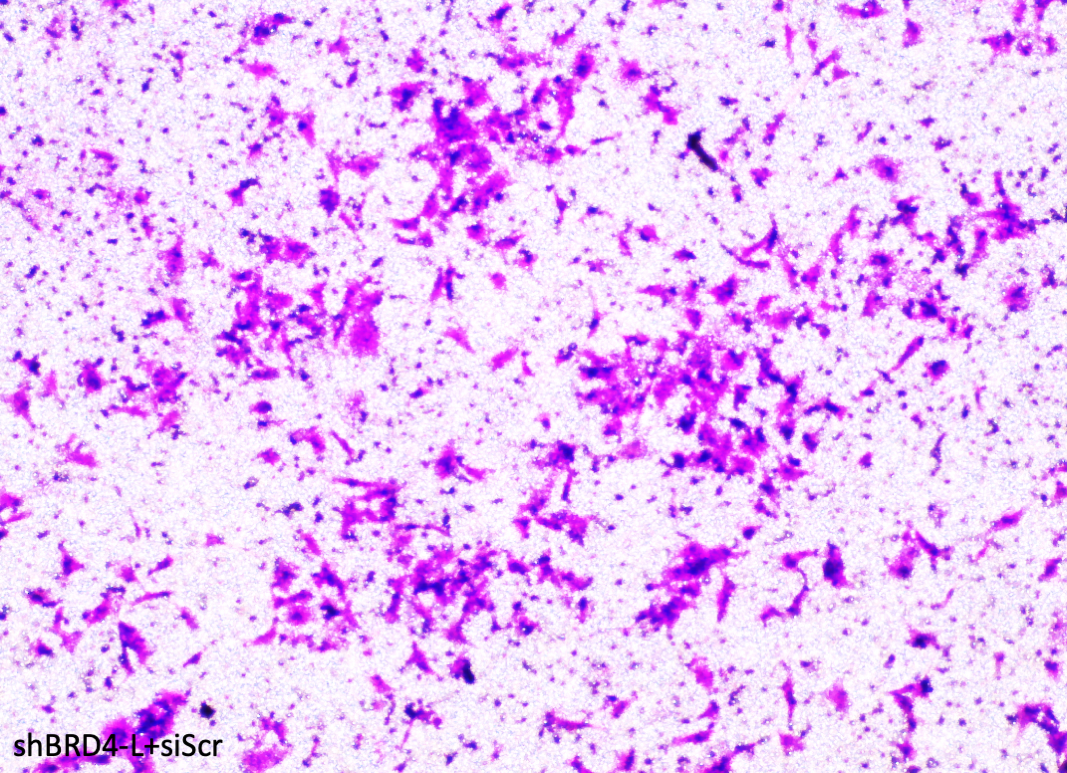

Supplement: Supplementary file 9 — Source Data Fig. EV5 [file 44319_2023_33_MOESM9_ESM.zip › Fig EV5/EV5B_INVASION/shBRD4-L+siScr.tiff]

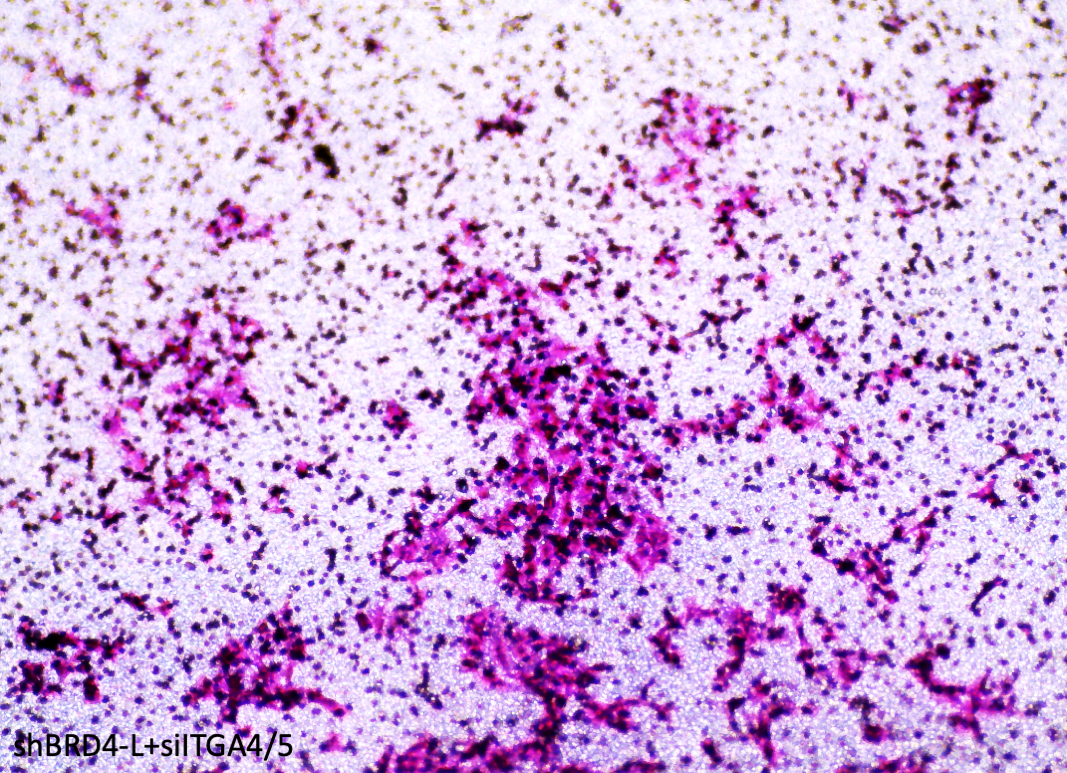

Supplement: Supplementary file 9 — Source Data Fig. EV5 [file 44319_2023_33_MOESM9_ESM.zip › Fig EV5/EV5B_INVASION/shBRD4-L+siITA4:5.tiff]

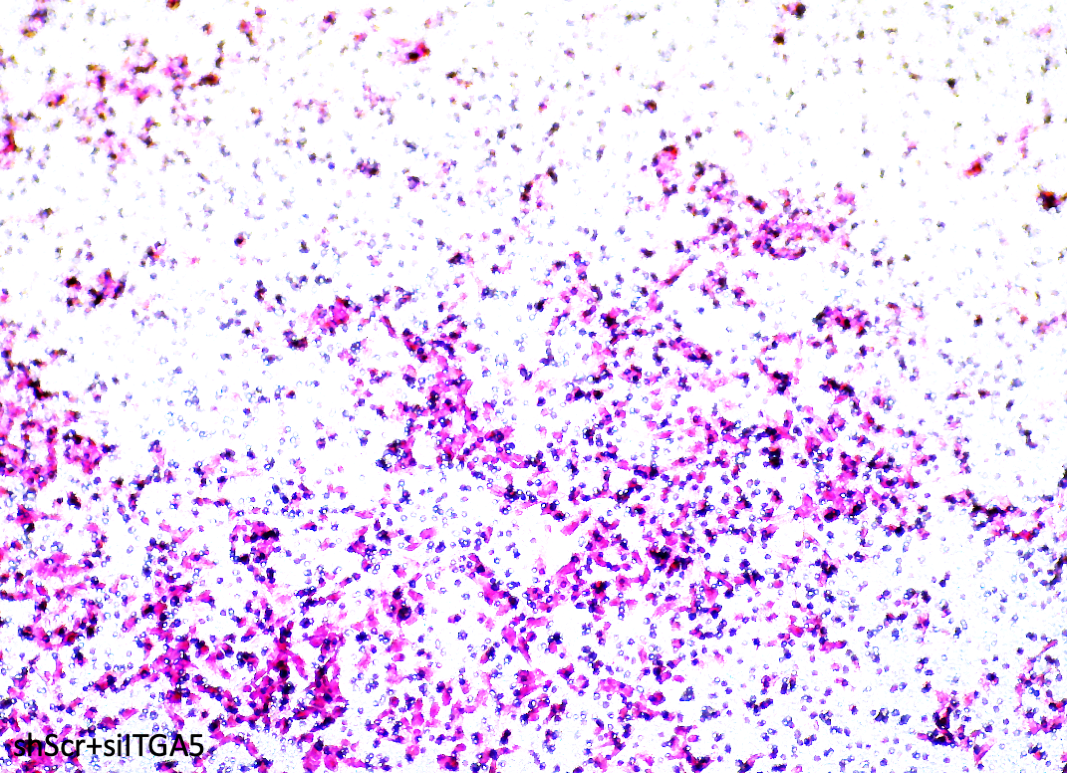

Supplement: Supplementary file 9 — Source Data Fig. EV5 [file 44319_2023_33_MOESM9_ESM.zip › Fig EV5/EV5B_INVASION/shScr+siITGA5.tiff]

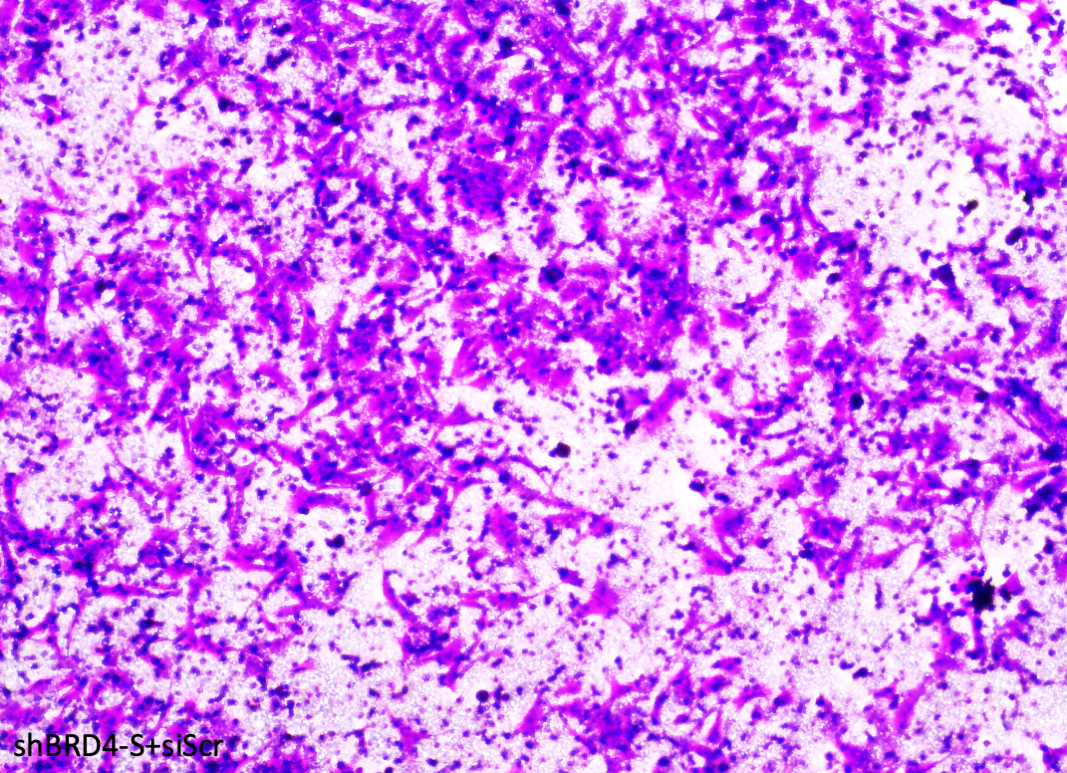

Supplement: Supplementary file 9 — Source Data Fig. EV5 [file 44319_2023_33_MOESM9_ESM.zip › Fig EV5/EV5B_INVASION/shBRD4-S+siScr.tiff]

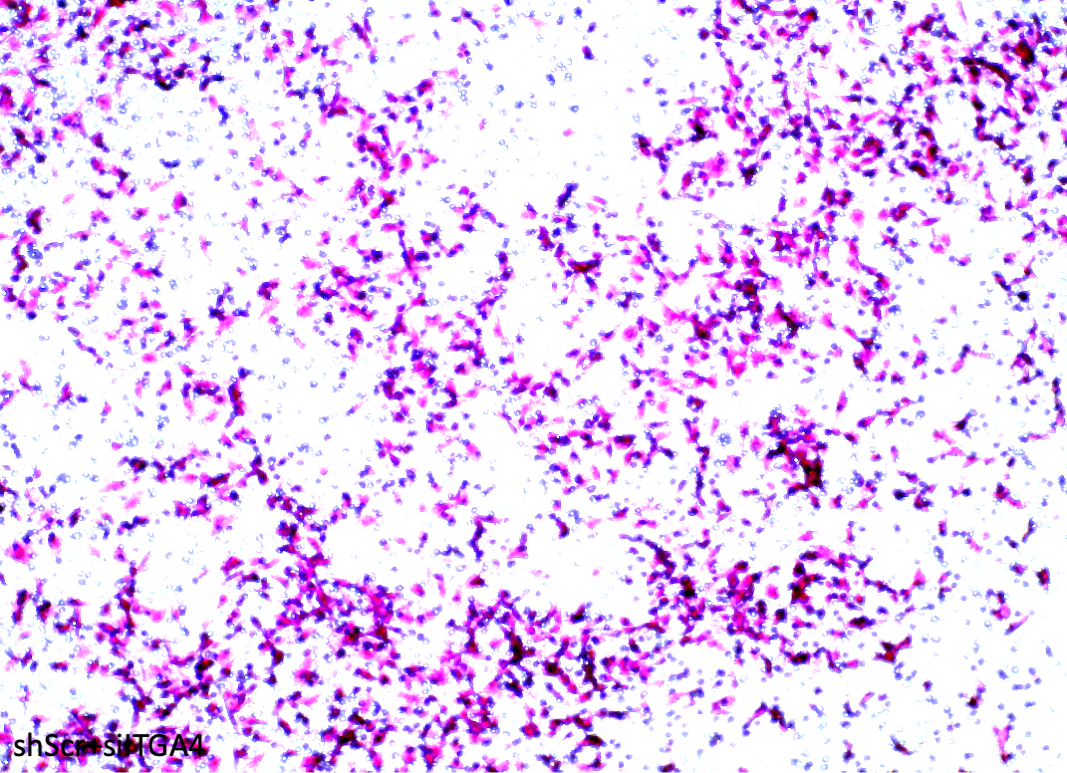

Supplement: Supplementary file 9 — Source Data Fig. EV5 [file 44319_2023_33_MOESM9_ESM.zip › Fig EV5/EV5B_INVASION/shScr+siITGA4.tiff]

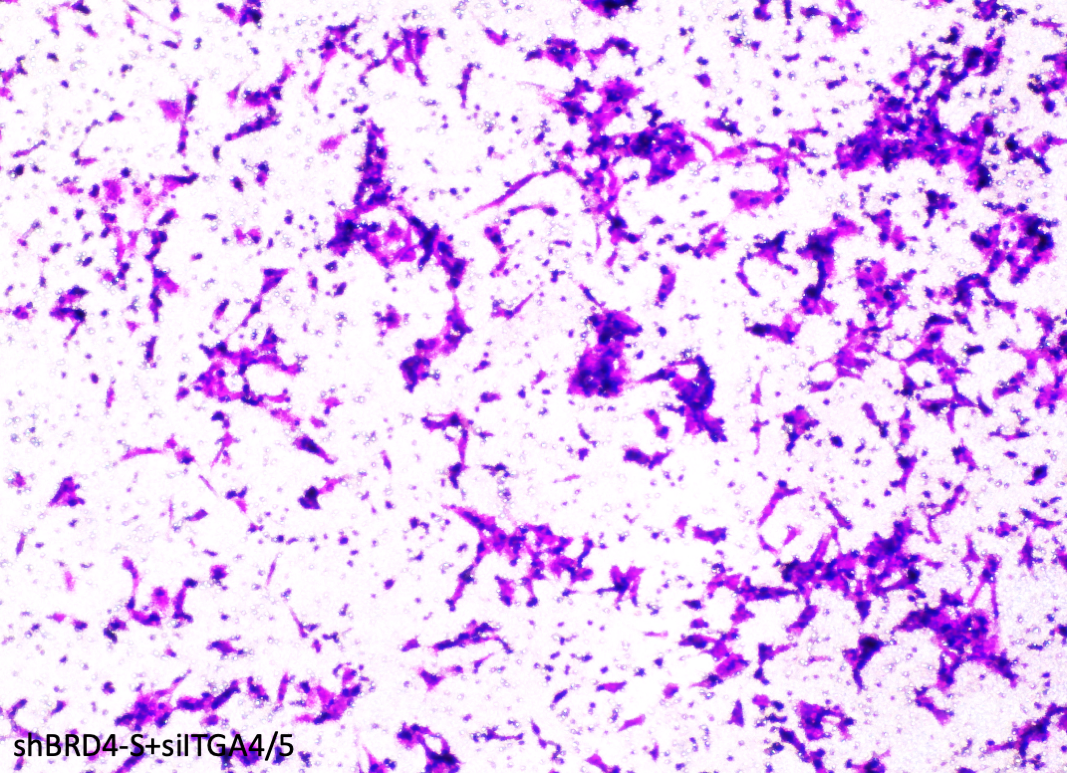

Supplement: Supplementary file 9 — Source Data Fig. EV5 [file 44319_2023_33_MOESM9_ESM.zip › Fig EV5/EV5B_INVASION/shBRD4-S+siITGA4:5.tiff]

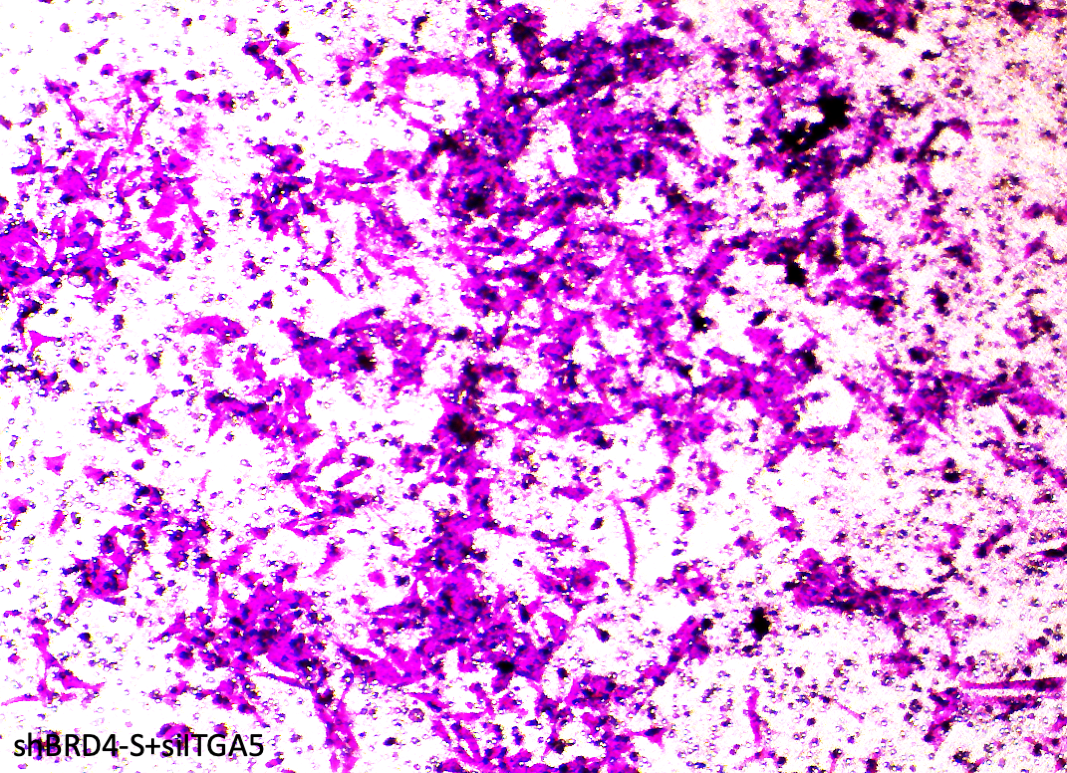

Supplement: Supplementary file 9 — Source Data Fig. EV5 [file 44319_2023_33_MOESM9_ESM.zip › Fig EV5/EV5B_INVASION/shBRD4-S+siITGA5.tiff]

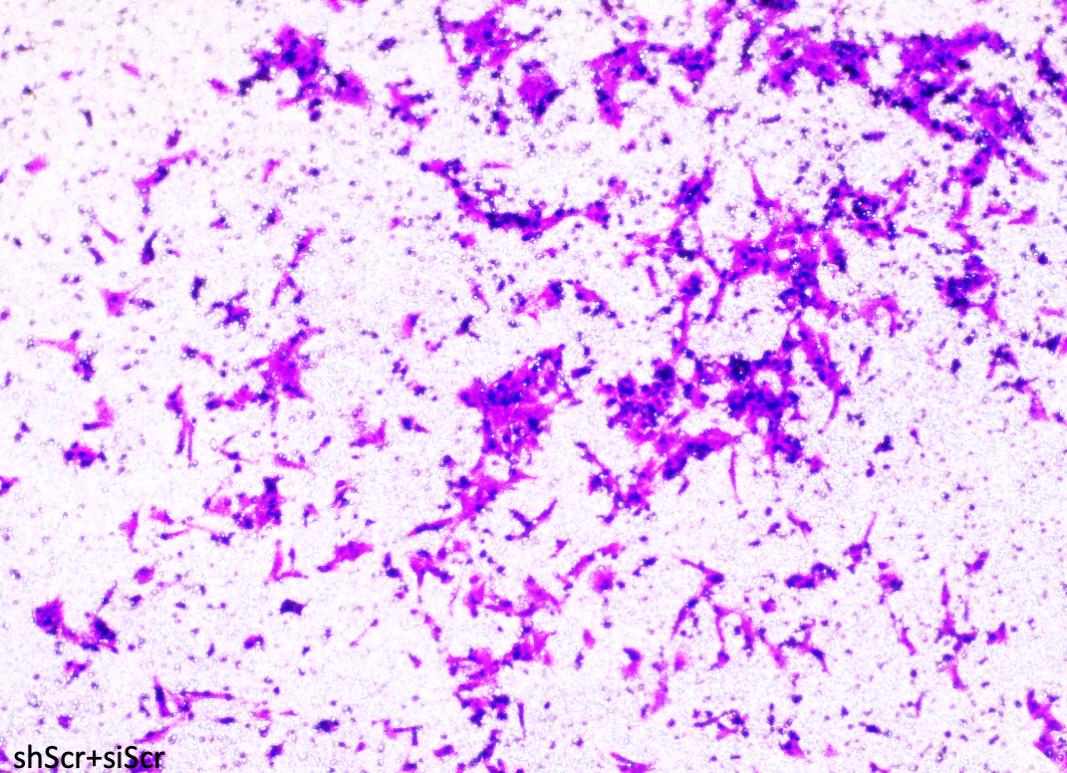

Supplement: Supplementary file 9 — Source Data Fig. EV5 [file 44319_2023_33_MOESM9_ESM.zip › Fig EV5/EV5B_INVASION/shScr+siScr.tiff]

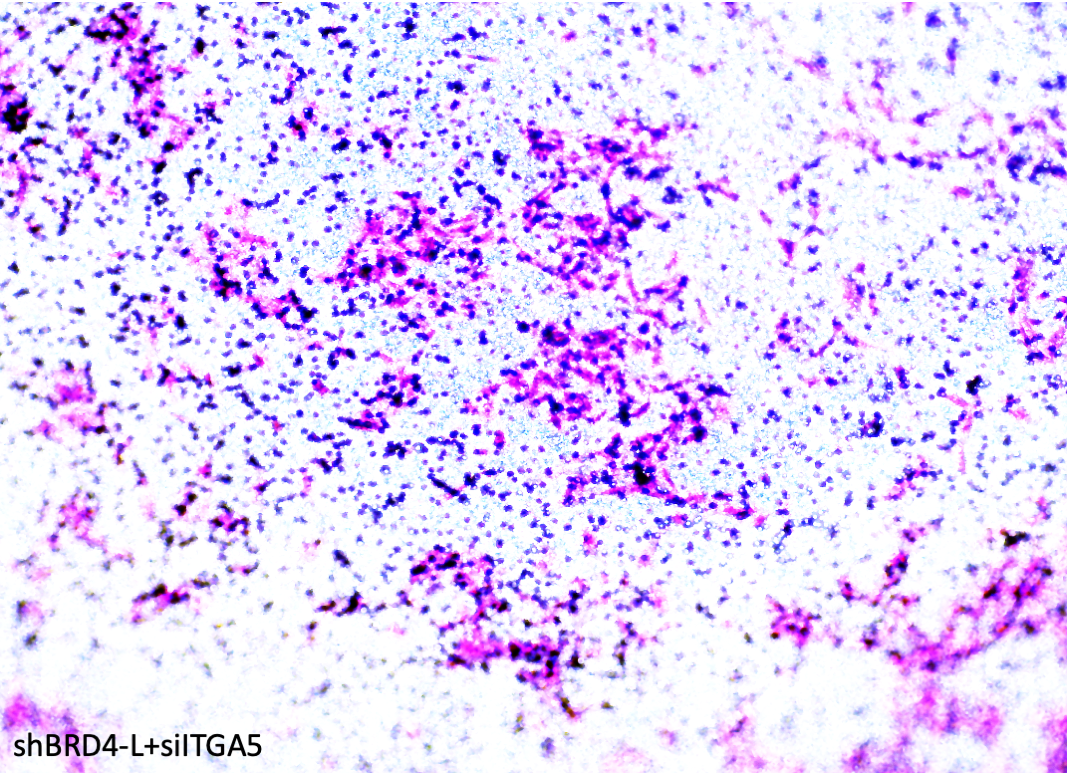

Supplement: Supplementary file 9 — Source Data Fig. EV5 [file 44319_2023_33_MOESM9_ESM.zip › Fig EV5/EV5B_INVASION/shBRD4-L+siITGA5.tiff]

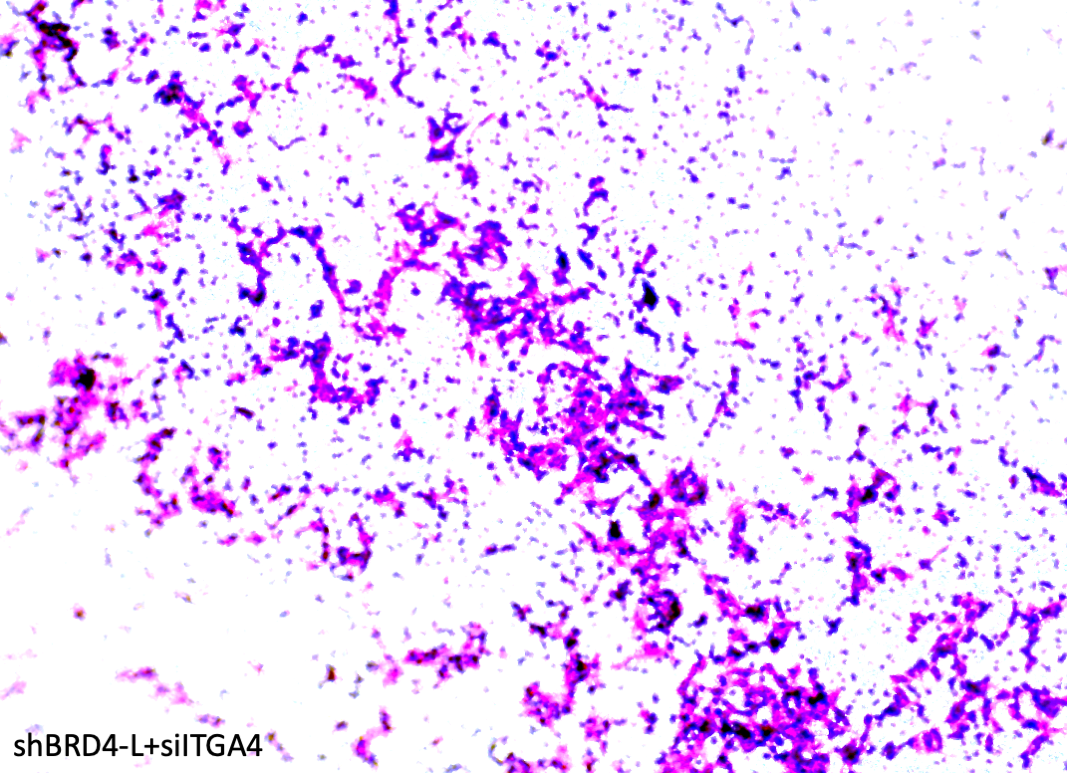

Supplement: Supplementary file 9 — Source Data Fig. EV5 [file 44319_2023_33_MOESM9_ESM.zip › Fig EV5/EV5B_INVASION/shBRD4-L+siITGA4.tiff]

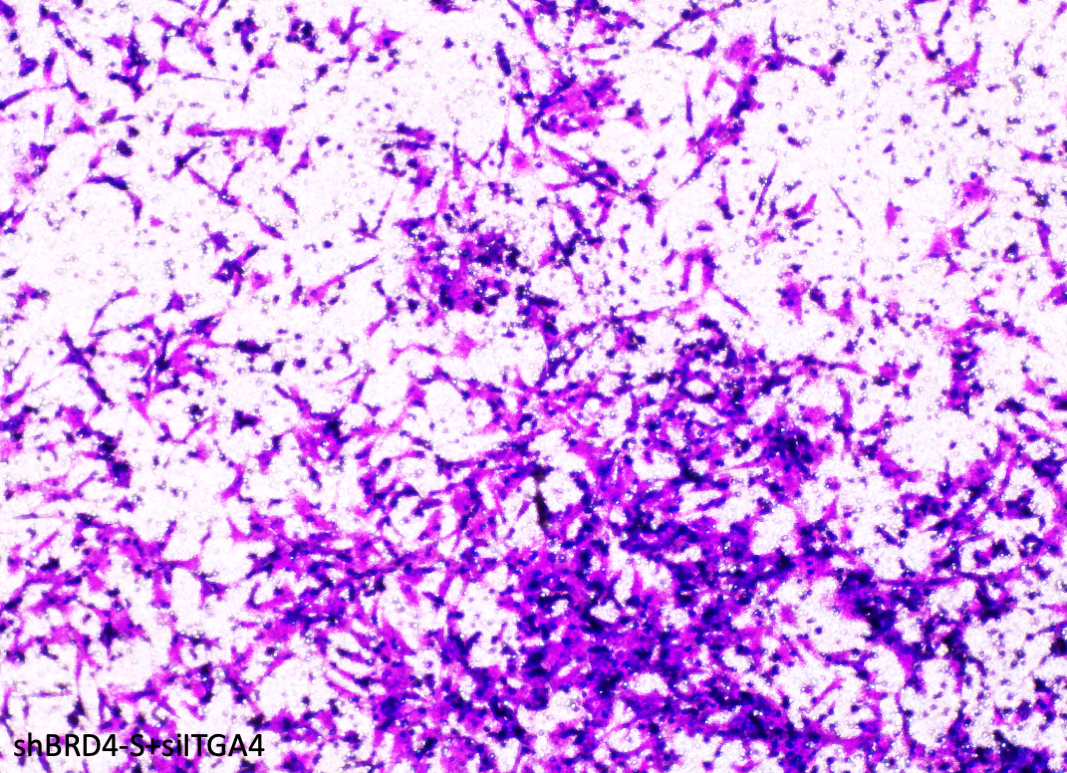

Supplement: Supplementary file 9 — Source Data Fig. EV5 [file 44319_2023_33_MOESM9_ESM.zip › Fig EV5/EV5B_INVASION/shBRD4-S+siITGA4.tiff]

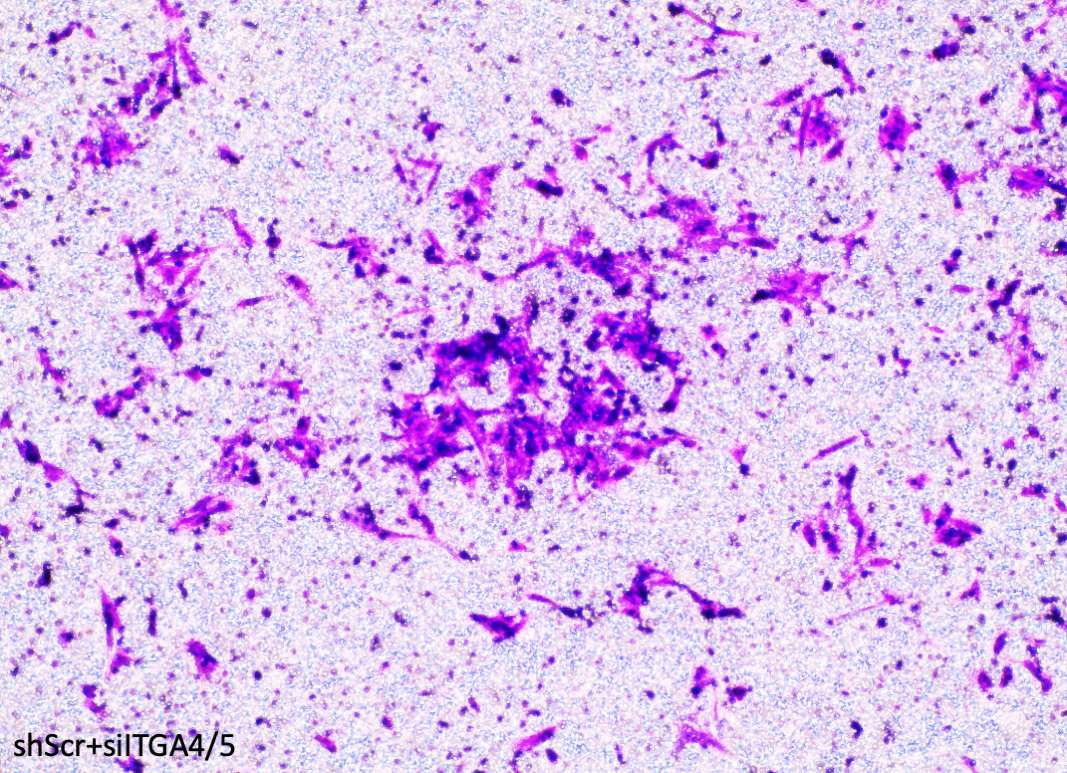

Supplement: Supplementary file 9 — Source Data Fig. EV5 [file 44319_2023_33_MOESM9_ESM.zip › Fig EV5/EV5B_INVASION/shScr+siITGA4:5.tiff]
