# Supplementary material for: BRD4 isoforms have distinct roles in tumour progression and metastasis in rhabdomyosarcoma
Source: EMBO Rep. 2024 Jan 8;25(2):832–52. doi: 10.1038/s44319-023-00033-1 (PMC10897194; doi:10.1038/s44319-023-00033-1)
Supplement: Supplementary file 10 — Expanded View Figures [file 44319_2023_33_MOESM10_ESM.pdf]

Expanded View Figures

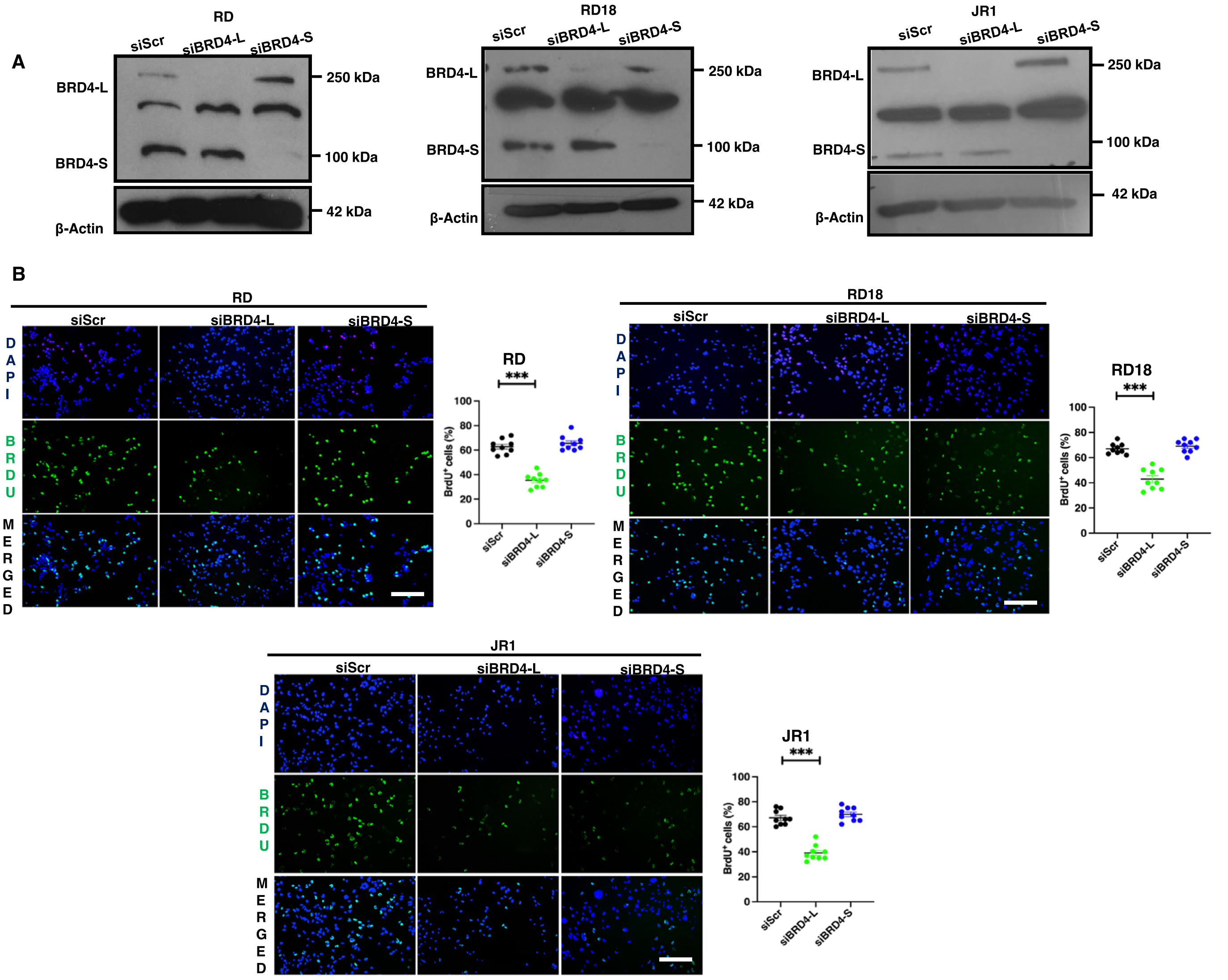

Figure EV1. BRD4-L knockdown represses proliferation.

(**A**) Western blot analysis showing transient BRD4 knockdown efficiency in siBRD4-L and siBRD4-S RD, RD18 and JR1 cells as indicated. β-Actin was used as the loading control. Images are representative of at least three biological replicates. (**B**) Proliferation was analysed by BrdU assay in control (siScr), siBRD4-L and siBRD4-S RD, RD18 and JR1 cells as indicated. Images are representative of at least three biological replicates. Scale bar: 100 μm. Scatter plot showing the percentage of $BrdU^{+}$ cells in siScr, siBRD4-L and siBRD4-S cells. Values correspond to the average ± SEM ($n = 3$ biological replicates with 3 technical replicates shown). Two-tailed non-parametric unpaired t test was performed for statistical analysis. $^{***}p \leq 0.001$.

 © The Author(s)

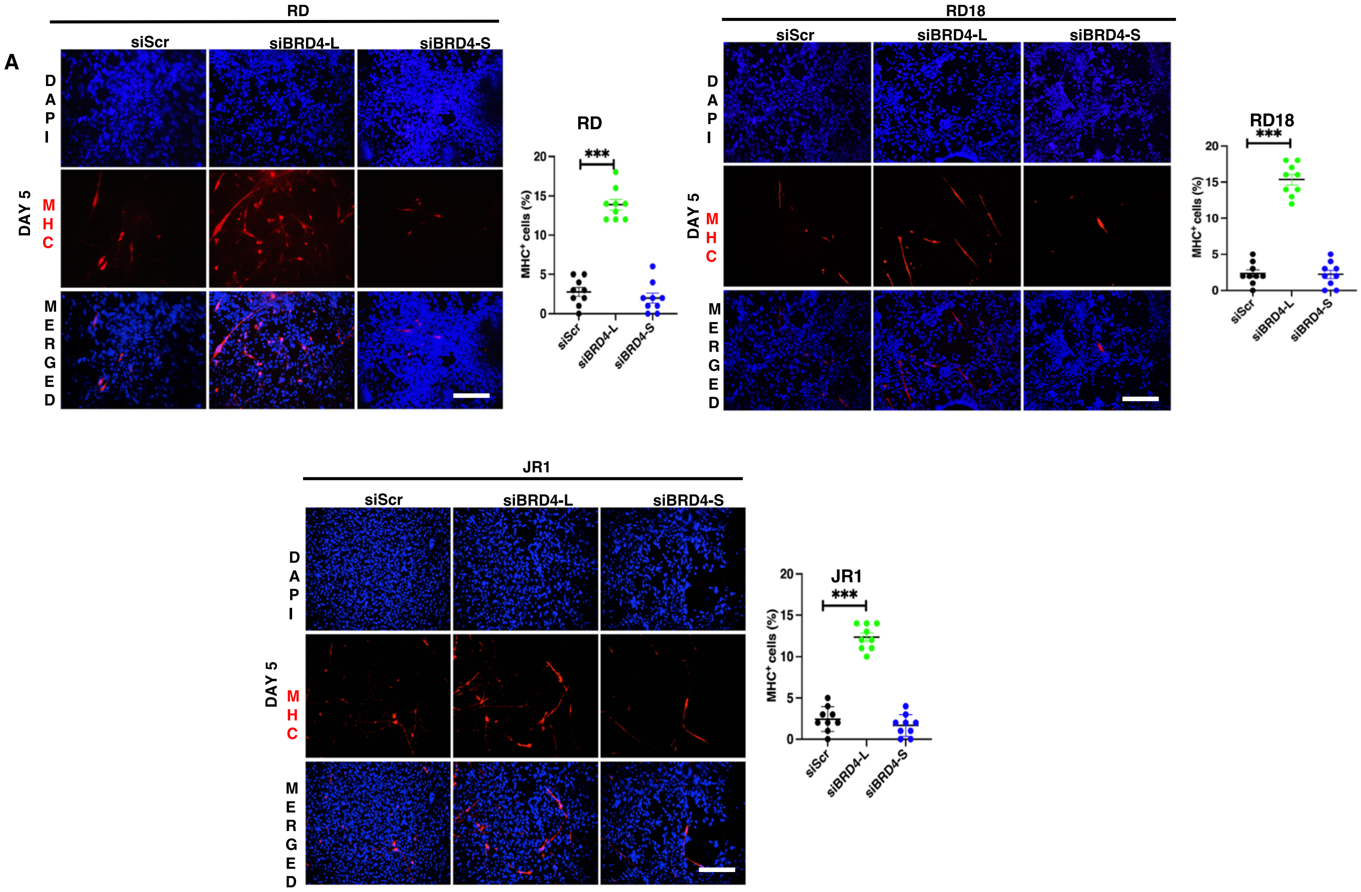

Figure EV2. BRD4-L knockdown enhances differentiation.

(**A**) Differentiation was analysed by immunofluorescence in siScr, siBRD4-L and siBRD4-S in RD, RD18 and JR1 cells as indicated. Forty-eight hours post-transfection, cells were cultured in differentiation media for 5 days and MHC^{+} cells were analysed using anti-MHC antibody. Images are representative of at least three biological replicates. Nuclei were stained with DAPI (blue). Scale bar: 100 μm. Scatter plots indicating the percentage of MHC^{+} cells in siScr, siBRD4-L and siBRD4-S cells. Values correspond to the average ± SEM ($n = 3$ biological replicates with 3 technical replicates shown). Two-tailed non-parametric unpaired t test was performed for statistical analysis. ***$p \leq 0.001$.

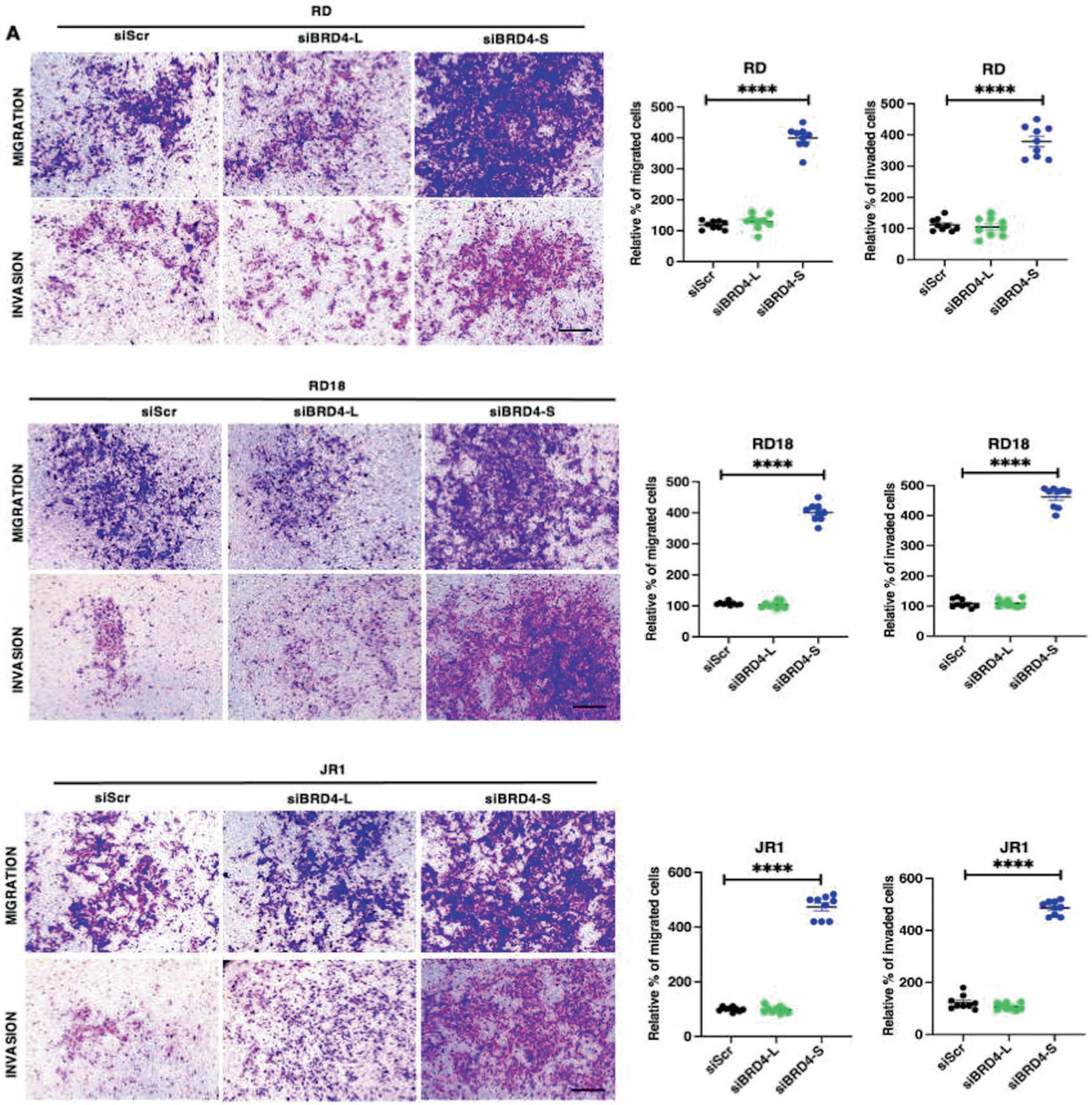

Figure EV3. BRD4-S knockdown increases cellular migration and invasion.

(**A**) Migration and invasion was analysed by Transwell assays in siScr, siBRD4-L and siBRD4-S RD, RD18 and JR1 cells as indicated. The inserts were stained with crystal violet. Images are representative of at least three biological replicates. Scale bar: 100 µm. Scatter plots indicating the percentage of migration and invasion is shown. Values correspond to the average ± SEM. Values correspond to the average ± SEM ($n = 3$ biological replicates with 3 technical replicates shown). Two-tailed non-parametric unpaired t test was performed for statistical analysis. ****$p \leq 0.0001$.

 © The Author(s)

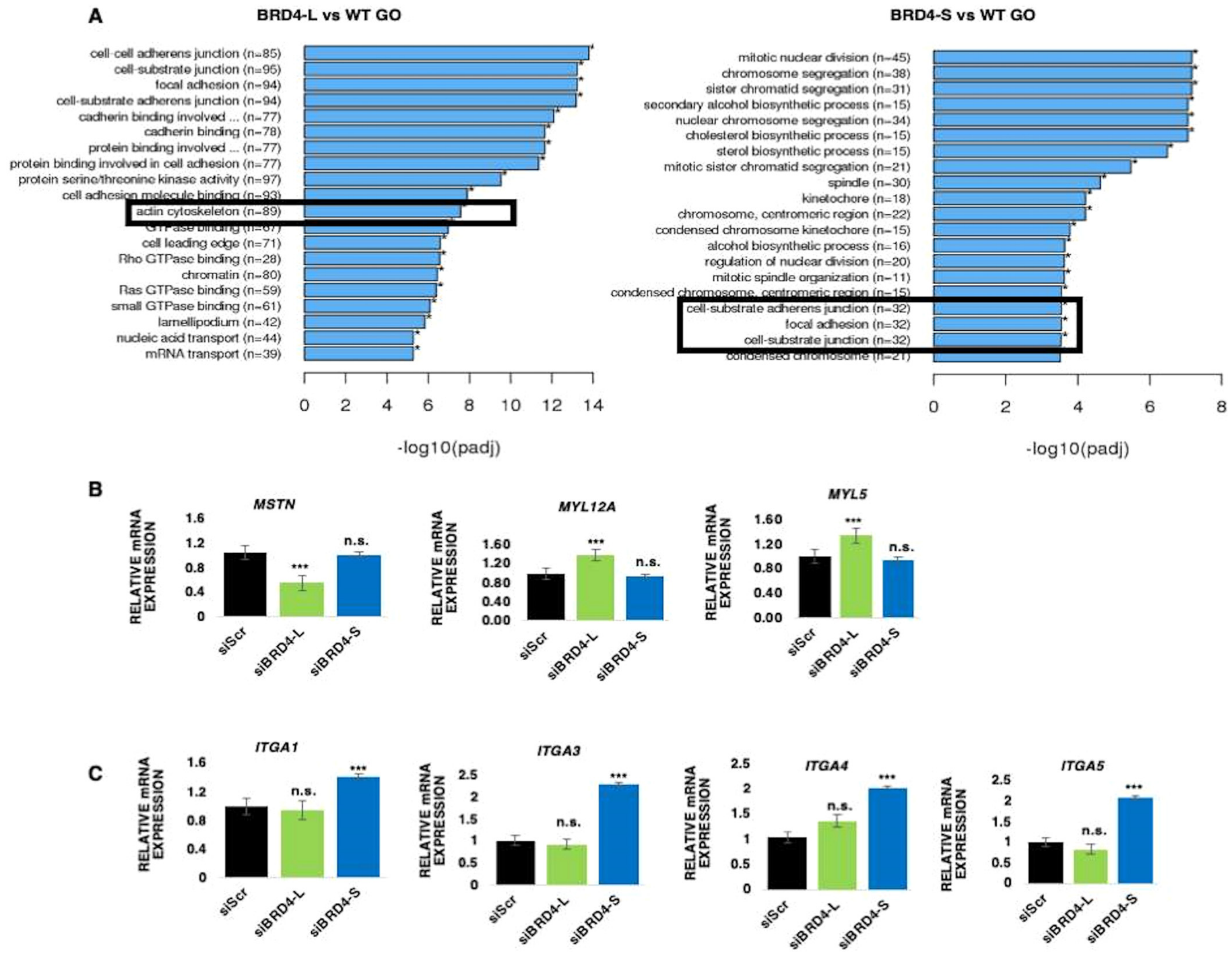

Figure EV4. Validation of BRD4-L and BRD4-S targets.

(**A**) GO enrichment histogram showing significantly enriched biological processes upon BRD4-L and BRD4-S knockdown based on the number of differentially expressed genes. (**B**) RT-qPCR analysis of *MSTN*, *MYL12A* and *MYL5* mRNA in siScr, siBRD4-L and siBRD4-S cells. The values correspond to average ± SEM ($n = 4$ biological replicates). n.s. = not significant, ***$p \leq 0.001$, n.s. not significant. (**C**) RT-qPCR analysis of the integrin (*ITGA1, ITGA3, ITGA4* and *ITGA5*) mRNA in siScr, siBRD4-L and siBRD4-S cells. The values correspond to average ± SEM ($n = 4$ biological replicates). Two-tailed non-parametric unpaired t test was performed for statistical analysis. n.s. not significant, ***$p \leq 0.001$, n.s. not significant.

© The Author(s)

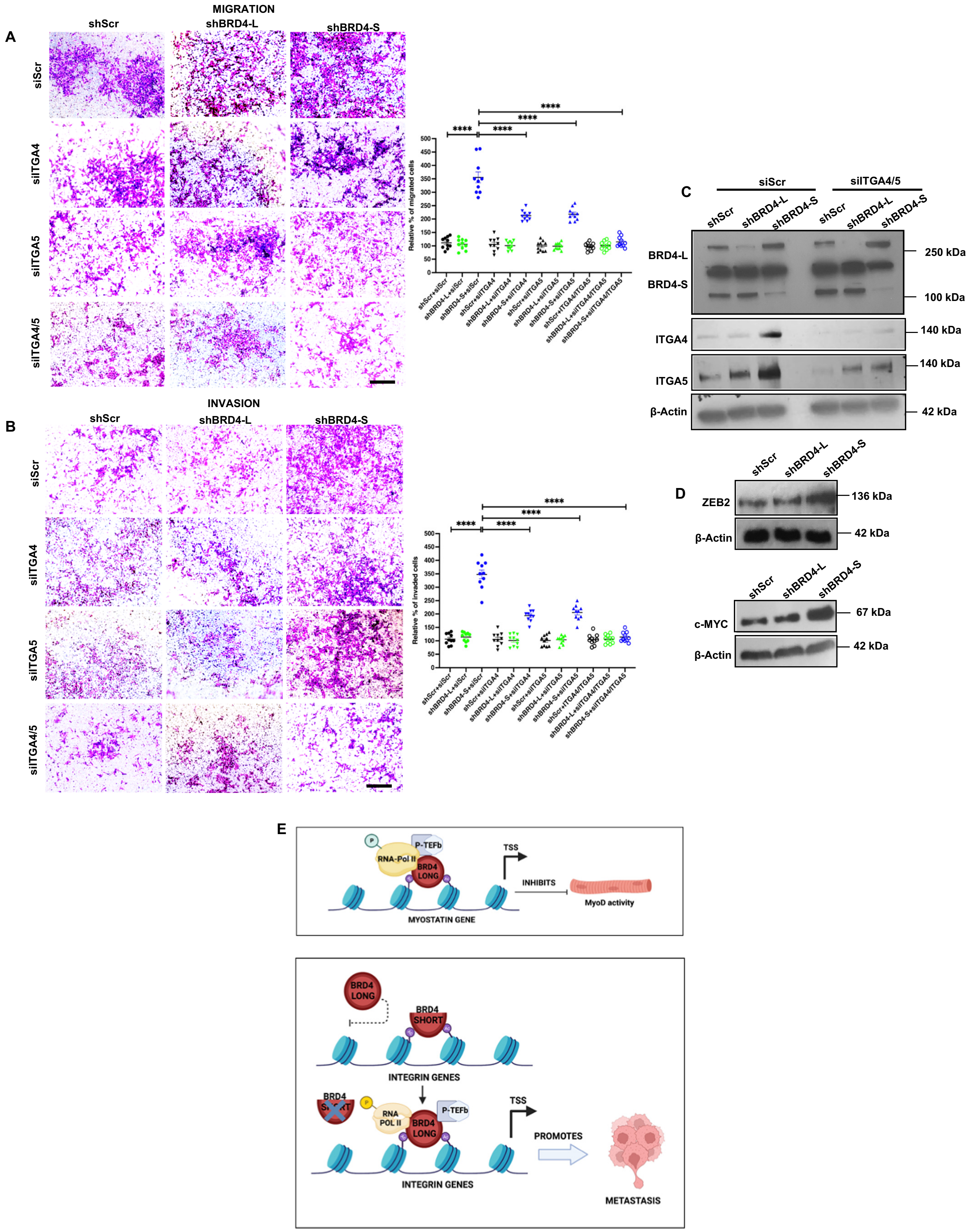
A
MIGRATION
shScr
shBRD4-L
shBRD4-S
siScr
siITGA4
siITGA5
siITGA4/5
Relative % of migrated cells
****
B
INVASION
Relative % of invaded cells
C
siScr
siITGA4/5
BRD4-L
BRD4-S
ITGA4
ITGA5
β-Actin
250 kDa
100 kDa
140 kDa
42 kDa
D
ZEB2
136 kDa
c-MYC
67 kDa
E
RNA-Pol II
P-TEFb
BRD4 LONG
TSS
INHIBITS
MyoD activity
MYOSTATIN GENE
BRD4 SHORT
INTEGRIN GENES
RNA POL II
PROMOTES
METASTASIS

 © The Author(s)

Figure EV5. ITGA4/5 knockdown rescues migration and invasion in shBRD4-S cells.

(**A**,**B**) Migration and invasion was analysed by Transwell assays in shScr, shBRD4-L and shBRD4-S by transfecting them with siScr, siITGA4, siITGA5 or siITGA4 and 5. The inserts were stained with crystal violet. Images are representative of at least three biological replicates. Scale bar: 100 μm. Scatter plots indicating the percentage of migration and invasion is shown. Error bars correspond to the average ± SEM ($n = 5$ biological replicates with 2 technical replicates shown). Two-tailed non-parametric unpaired t test was performed for statistical analysis. $^{***}p \le 0.001$, $^{****}p \le 0.0001$. (**C**) Western blot analysis showing transient ITGA4 and ITGA5 knockdown efficiency in shScr, shBRD4-L and shBRD4-S cells as indicated. β-Actin was used as the loading control. Images are representative of at least three biological replicates. (**D**) Western blot analysis showing ZEB2 and c-MYC expression in shScr, shBRD4-L and shBRD4-S cells as indicated. β-Actin was used as the loading control. Images are representative of at least two biological replicates. (**E**) Graphical model summarizing the function of the BRD4 isoforms in ERMS. Source data are available online for this figure.

© The Author(s)
